# Acute effect of dietary nitrate on forearm muscle oxygenation, blood volume and strength in older adults: A randomized clinical trial

**DOI:** 10.1371/journal.pone.0188893

**Published:** 2017-11-30

**Authors:** Gustavo Vieira de Oliveira, Marina Morgado, Carlos Adam Conte-Junior, Thiago Silveira Alvares

**Affiliations:** 1 Nutrition and Exercise Metabolism Research Group, Nutrition Institute, Universidade Federal do Rio de Janeiro, Macaé, Rio de Janeiro, Brazil; 2 Department of Food Technology, Universidade Federal Fluminense, Macaé, Brazil; 3 Department of Basic Nutrition and Dietetics, Nutrition Institute, Universidade Federal do Rio de Janeiro, Rio de Janeiro, Brazil; Norwegian University of Science and Technology, NORWAY

## Abstract

Both recovery time of post-exercise muscle oxygenation and muscle strength decline with aging. Although beetroot consumption has been shown to improve muscle oxygenation and exercise performance in adults, these effects in the elderly has not been addressed. The aim of the present study was to evaluate the effect of a beetroot-based gel (BG) on muscle O_2_ saturation, blood volume (tHb) and handgrip strength in the elderly in response to handgrip exercise. In a randomized crossover double-blind design, twelve older subjects consumed BG (100 g of beetroot-based gel containing ~ 12 mmol nitrate) or PLA (100 g of nitrate-depleted gel nitrate-depleted). The subjects performed a rhythmic handgrip exercise which consisted of a one 1-min set at 30% of the maximal voluntary contraction (MVC) of each subject, followed by a 1 min recovery. The muscle oxygenation parameters and tHb were continuously monitored by using near-infrared spectroscopy. MVC was evaluated at baseline, immediately after exercise, and 30 min afterwards. The muscle O_2_ resaturation rate during exercise recovery was greater in the BG when compared to PLA condition (1.43 ± 0.77 vs 1.02 ± 0.48%.s^-1^; *P* < 0.05). Significant increase was observed in tHb during exercise recovery (10.25 ± 5.47 vs 6.72 ± 4.55 μM; *P* < 0.05) and significant reduction of handgrip strength decline was observed 30 min after exercise in BG (- 0.24 ± 0.18 vs—0.39 ± 0.20 N; *P* < 0.05). In summary, a single dose of a beetroot-based gel speeds up muscle O_2_ resaturation, increases blood volume and improves recovery of handgrip strength after handgrip exercise in older adults.

## Introduction

Aging causes numerous changes to the cardiovascular system including increased total peripheral resistance and vascular endothelial dysfunction, which has been associated with the reduced bioavailability of nitric oxide (NO) [1]. Since NO may influence O_2_ utilization by the cells, this suggests that O_2_ delivery to the working muscle during exercise may be compromised in the elderly.

Muscle O_2_ saturation (SmO_2_) measured by near-infrared spectroscopy reflects the balance between muscle O_2_ delivery and muscle O_2_ utilization during exercise and/or during the recovery time after exercise [2]. Recovery time of muscle O_2_ resaturation following the completion of exercise has been used as an index of deficient muscle O_2_ delivery in relation to muscle O_2_ demand [3,4]. Kutsuzawa et al. [3] demonstrated that recovery rate of muscle O_2_ resaturation after submaximal handgrip exercise was delayed with increased age. Ichimura et al. [4] demonstrated that the half-recovery time of oxygenated hemoglobin/myoglobin was significantly related to age, since it was greater in the elderly subjects than in the middle-aged subjects. These data suggest that age-related prolongation in recovery time of muscle O_2_ resaturation reflects the age-related decline in muscle function, which is dependent on O_2_ delivery to working muscles.

Recently, the consumption of beetroot, a food rich in nitrate, has gained popularity in scientific literature due to the possible effect of the nitrate present in this food to promote NO bioconversion [5,6]. Furthermore, short-term beetroot consumption has been demonstrated to improve muscle oxygenation status during moderate to severe exercise in healthy adults [7,8,9,10]. Whether beetroot consumption has the ability to increase muscle O_2_ delivery and/or increase the capacity of muscle to extract or utilize O_2_ in older adults has not been determined. For this reason, the aim of the present work was to evaluate whether beetroot-based nutritional gel rich in nitrate promotes changes in the SmO_2_ parameters, blood volume and muscle strength of the forearm following the completion of a handgrip exercise protocol. Analysis of urinary nitrate and nitrite were also addressed. We hypothesized that a single dose of the beetroot gel rich in dietary nitrate would result in increased NO bioconversion and would, consequently, promote increases in the muscle oxygenation, blood volume and forearm strength after handgrip exercise in the elderly.

## Materials and methods

### Participants

**Fig 1:**
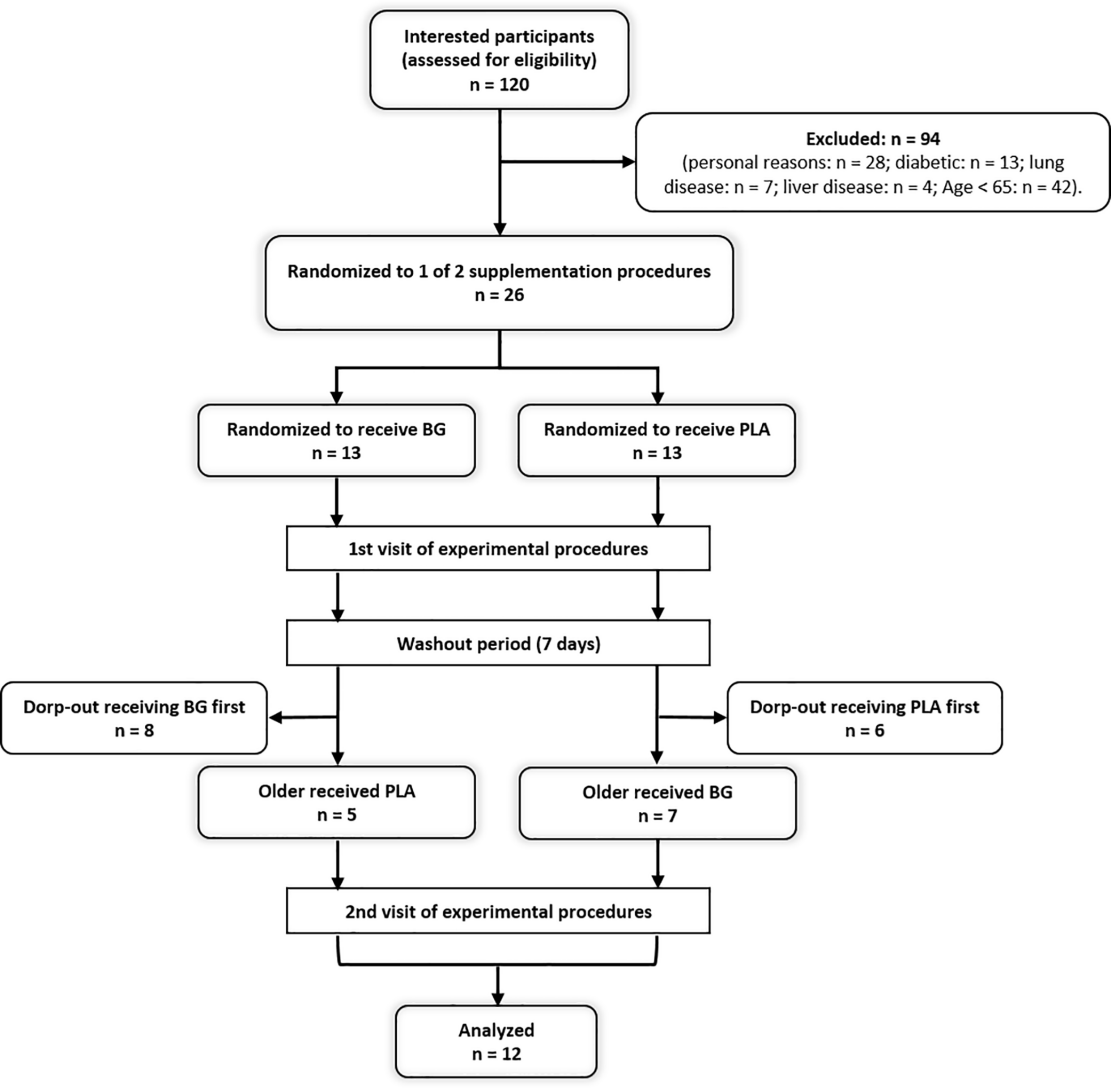
Flow diagram of the progress through the phases of the randomized, crossover and placebo-controlled study.

One hundred twenty individuals were recruited through announcements in flyers and advertisements during community events at Family Center of the Elderly in Macaé, Rio de Janeiro, Brazil. Twenty-six elderly individuals were eligible to participate in the study and twelve completed all the experimental procedures (Fig 1). An a priori power analysis was conducted (G*Power v. 3.0.1) for an F test (repeated measures, within-between interaction for two time points). On the basis of a statistical power (1 – β) of 0.80, a moderately large effect size (0.5), and an overall level of significance of 0.05, at least 12 participants were needed to detect a statistical difference. Exclusion criteria included people aging < 65 years, smoking, beetroot allergy, unwillingness to avoid beetroot products during the entire study, liver and kidney disease, diabetes, and/or acutely ill. All eligible participants were fully informed of the nature and purpose of the investigation and provided written consent to participate. All experimental procedures were performed in accordance with the ethical standards of the Declaration of Helsinki and approved by the institutional ethics committee and the trial was registered in the ClinicalTrials.gov (NCT02772900).

### Study design

**Fig 2:**
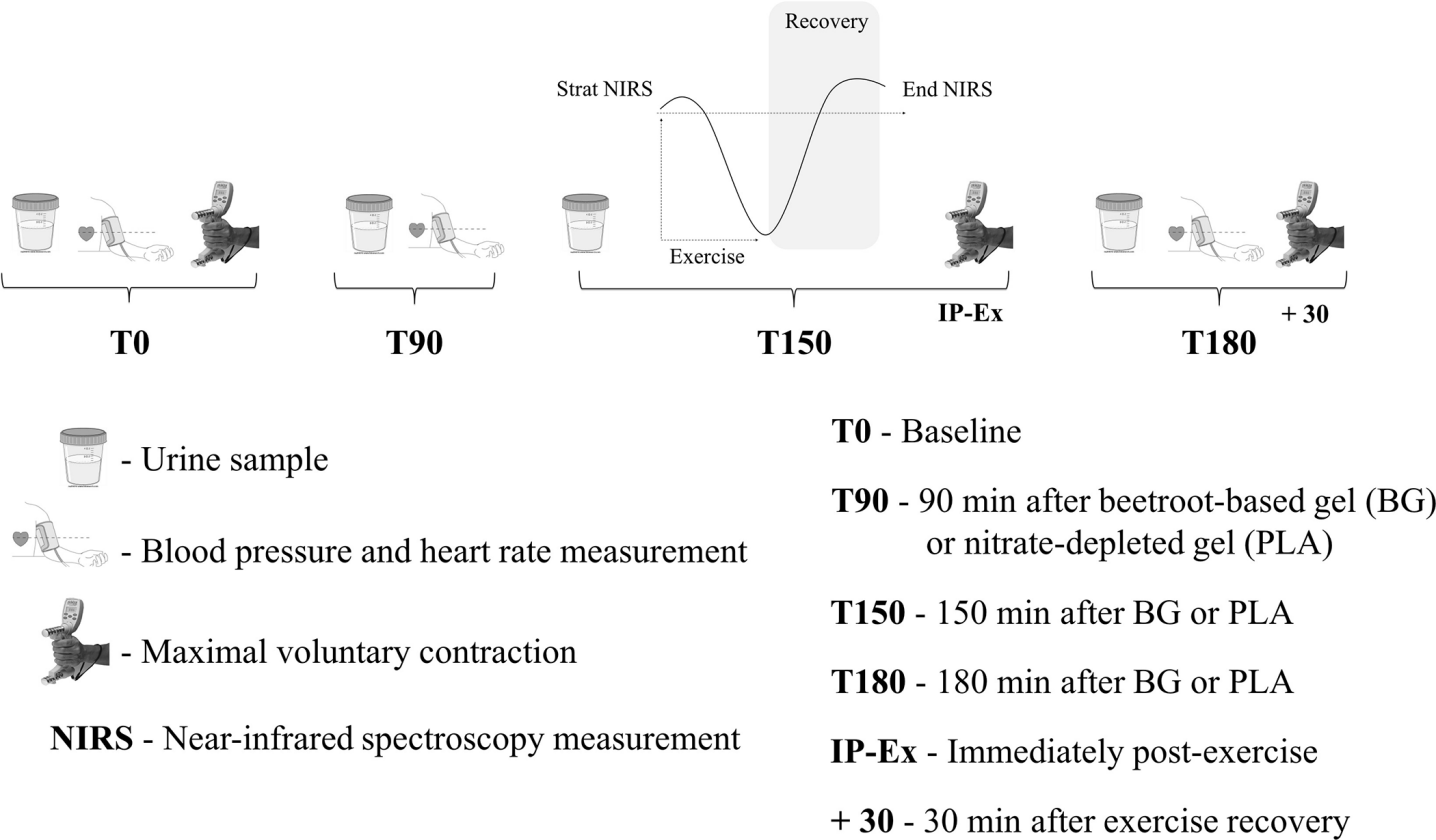
Experimental design of the study.

The study was conducted in a randomized, double-blind, crossover and placebo-controlled way conducted from April 2016 to January 2017. All participants reported to the laboratory on three occasions, with at least 1-week interval between visits. The first visit was used to explain the experimental procedures and collect clinical and anthropometric data, as well as to ensure familiarity with the handgrip exercise protocol. In the second and third visits urine samples were drawn at baseline (T0) after a 10-min period of quiet rest. Thereafter, subjects were randomly divided into either a beetroot-based nutritional gel (BG) or a control nitrate-depleted gel (PLA). NIRS measurements in response to the handgrip exercise protocol began approximately 150 min after the nutritional intervention (T150) and lasted for approximately 10 min. Urine samples were drawn again at T90, T150 and T180 after nutritional intervention (Fig 2). The three visits were held between 07:00 and 12:00 a.m. The participants were instructed to fast for at least 8 hours before each visit, restrict carbonated mineral water, avoid the intake of foods rich in nitrate and nitrite, and not to use any mouthwash.

### Nutritional intervention

All subjects were orally administered either 100 g of a beetroot-based nutritional gel (BG) or a nitrate-depleted gel (PLA) in identical forms in a double-blind and randomized manner. After 5 minutes of complete consumption of the gel, 400 mL of deionized water was provided to the subject in order to eliminate any residual gel in the mouth. The content of nitrate of the BG and PLA consumed by the subjects of the present study were 12.2 ± 0.18 and 0.02 ± 0.02 mmol/100g, respectively. The BG and PLA were prepared in our laboratory according to the procedures Oliveira et al. [11] and Morgado et al. [12]. Briefly, in order to produce 100 g of the beetroot-based gel, ~16.2 g of the beetroot powder was mixed with ~80 ml of BJ, ~2.8 g of carboxymethylcellulose and ~1ml of artificial orange flavor, and mixed until it obtained the consistency of a gel. These steps last approximately ~30 min. For nitrate-depleted gel production there was an additional three-hour preparation time for the removal of nitrate from beetroot. We have chosen to use a beetroot-based gel product in the present study since the consistency of the beetroot-based gel is likely to increase the amount of time and mouth contact associated with the intake of nitrate, which may increase the ability of the nitrate present in the beetroot to be reduced to nitrite in the oral cavity by the enzymatic action of nitrate reductase via oral commensal bacteria.

### Urinary nitrite and nitrate analysis

Nitrite and nitrate analyses in urine were performed as previously described by Croitoru [13], using a high-performance liquid chromatography (HPLC) system. Nitrite is a metabolic end-product derived from oxidation of NO and was used in the present study as an indicator of NO bioconversion/bioavailability [6]. Measurements were made at T0, T90, T150 and T180. Sodium nitrite and nitrate was used for quantification, and the results were expressed as mmol.mmol^-1^ creatinine for nitrate and μmol.mmol^-1^ creatinine for nitrite. On the day before each visit, the participants were instructed to avoid foods rich in nitrate and nitrite in their diet.

### Muscle oxygenation and blood volume measurements

Forearm SmO_2_ of the flexor muscle (flexor carpi radialis) of each subject’s dominant forearm was continuously monitored using a commercially available portable NIRS device (PortaMon, Artinis, Medical Systems). Portable NIRS had 3 transmitters and one receiver. For congruence in measurement depth, the inter-optode distance of 35 mm was used for further analysis of the concentration changes in oxyhaemoglobin (O_2_Hb) and deoxyhemoglobin (HHb). This device is a 2-wavelength, continuous wave system, which simultaneously uses the modified Beer-Lambert law and spatially resolved spectroscopy (SRS) methods [14]. The portable NIRS device was placed on the skin, over the dominant forearm muscle, exactly 5 cm distal of the medial epicondyle of the humerous. Forearm skin-fold thickness (9.0 ± 8.7 mm) of each subject was less than the penetration depth (approximately 15 mm) of the portable NIRS device that is half of the inter-optode distance (35 mm), ensuring that measurements were recorded from the forearm muscle tissue [15]. Measurement of the skin-fold thickness was grasped from proximal forearm, in anteromedial aspect of the flexor muscles of the forearm, approximately 5 cm distal of the medial epicondyle of the humerous. The skin-fold was raised vertically with the thumb and index finger of the left hand and the forearm skin-fold was measured using a Lange Skin-fold Caliper. To secure the probe on the skin and minimize movement during exercise, an elastic bandage was wrapped around the subject’s forearm. The wrap also helped to minimize the possibility that extraneous light could influence the signal. During all tests, the NIRS system was connected to a personal computer via Bluetooth for data acquisition (10Hz), analogue-to-digital conversion and subsequent analysis of the raw data (i.e. no filter was used) using native software (Oxysoft version 2.1.6; Artinis Medical Systems). The software calculates changes in light absorption at the different wavelengths (750 and 850 nm) and converts them to relative concentrations of O_2_Hb and HHb using the modified Lambert law to correct for light scattering within the tissue. A fixed value of 4 for the differential path-length factor (DPF) was used to calculate absolute difference changes [16,17]. Blood volume (tHb) was calculated as O_2_Hb + HHb and SmO_2_ was calculated as [O_2_Hb/tHb]*100 using the spatially resolved spectroscopy method [18,19].

The following NIRS parameters were considered for statistical analysis: 1) SmO_2_ and tHb at baseline (Pre), 1, 5, 10, 20, 30, 40, 50 and 60 seconds of exercise and exercise recovery. SmO_2_ and tHb were reported as changes from baseline (30 s prior to exercise) and each time point (i.e.: Pre, 1, 5, 10, 20, 30, 40, 50 and 60 seconds) was converted to seconds by calculating the average of their respective time in milliseconds during exercise and exercise recovery (e.g.: the first second was calculated by the average of the first 60 milliseconds of data acquisition during exercise and exercise recovery, the fiftieth second was calculated by the average of the first 300 milliseconds of data acquisition during exercise and exercise recovery, and so on…); 2) Minimum SmO_2_ (SmO_2min_), which corresponds to the minimum SmO_2_ values within 60 s the exercise; 3) ΔSmO_2_ half-desaturation time (ΔSmO_2½DT_), which corresponds to the period of time between the start of the exercise until SmO_2_ reaches 50% of the difference between baseline SmO_2_ and the minimum SmO_2_ during the exercise; 4) SmO_2_ desaturation rate (SmO_2DR_), which corresponds to the downslope of SmO_2_ during the exercise; 5) SmO_2_ resaturation rate (SmO_2RR_), which corresponds to the upslope of SmO_2_ during the exercise recovery; and 6) Exercise and exercise recovery tHb amplitude (ΔtHb_Ex_ and ΔtHb_Rec_, respectively) was identified as the difference between the maximum and minimum tHb values within exercise and exercise recovery period.

### Forearm muscle strength

Dominant handgrip strength was measured by using an adjustable, hydraulic handgrip dynamometer (JAMAR, Model 5030J1, Sammons Preston Roylan, Bolingbrook, IL, USA). The procedures of the handgrip strength measurement were performed as described by Beam and Adams [20]. Three repetitions were performed with 30 s of rest between repetitions at baseline, immediately after the handgrip exercise protocol (IP-Ex), and approximately 30 min after exercise (+30). Each attempt was terminated when force showed a clear stagnation or a decrease. The maximal voluntary contraction (MVC) force was recorded in kg and converted to newtons by multiplying the kg value by 9.8067. The strength values were expressed by the difference with baseline values (in newtons) and adjusted to each subject’s body weight.

### Exercise protocol

The exercise consisted of rhythmic contractions of their forearm muscles at a controlled rate (60 contractions per minute, 0.5-s contraction/0.5-s relaxation) and range of motion (10-cm excursion of the pulley wire with each contraction). Each subject completed one 1-min bout of exercise at 30% of the MVC of each subject, with 1 min of quiet recovery. Subjects were in a supine position and used the dominant arm to perform the exercise.

### Statistical analysis

The normality, homogeneity of variances and sphericity of the data were examined with the Shapiro-Wilk, Levene and Mauchly tests, respectively. To identify differences in the muscle O_2_ saturation parameters (SmO_2DR_ and SmO_2RR_) and blood volume (ΔtHb_Ex_ and ΔtHb_Rec_) between BG and PLA interventions, a Paired t-test was used. When the assumption of normality and homogeneity of variances were violated, a Wilcoxon test was used. To identify differences in SmO_2_, tHb, forearm muscle strength, urinary nitrite and nitrate between BG and PLA, a two-way ANOVA with repeated measures on one factor (time) was used. In addition, to verify whether the order of BG or PLA condition affected the results, ANOVA with repeated measurement was also performed in this study. When a significant *F* was found, additional post-hoc tests with Bonferroni adjustment were performed. When the assumption of normality and homogeneity of variances were violated, a Friedman Test followed by post hoc analysis with Wilcoxon signed-rank tests and a Bonferroni adjustment were used. For all variables, when sphericity was violated, a Greenhouse-Geisser correction was used. Statistical significance was set at the 0.05 level of confidence. All analyses were performed using a commercially available statistical package (IBM SPSS Statistics version 23 for Mac, Chicago, IL), and the results were expressed as means ± standard deviation (SD).

## Results

The assumption of normality, homogeneity of variance and sphericity were violated for SmO_2_ and tHb during exercise. In addition, for SmO_2DR_ the assumption of normality was violated. Therefore, a non-parametric test was used for statistical analysis of these variables. Sphericity was violated for urinary nitrate and nitrate. For all other variables, the assumptions of normality, homogeneity of variances and sphericity were not violated. The plot of each residuals analysis of SmO_2_ and tHb during exercise and recovery period for both BG and PLA conditions was included as Supplementary Materials (S1 File).

Of the twenty-six eligible participants (100%) in the randomization phase of the study, twelve (three men and nine women) (46%) completed the study. Among the 14 participants that declined (54%), eleven participants withdrew for personal reasons and three participants withdrew due to illness unrelated to the study. Among the personal reasons, the discomfort of the exercise protocol and the duration of each experimental procedure visit (which lasted approximately 4 hours each) were the most stated by the participants who declined. The baseline volunteers’ characteristics are shown in Table 1.

**Table 1:** Baseline characteristics of the subjects completing the study.

n (female)	12 (9)
Age (years)	68.8 ± 3.5
Body mass (kg)	70.4 ± 8.7
BMI (kg/m^2^)	29.3 ± 3.0
Waist circumference (cm)	97.5 ± 9.0
MVC (N)	26.5 ± 5.79

The values are mean ± SD. BMI = Body mass index, MVC = maximal voluntary contraction.

### Urinary nitrite and nitrate analysis

The urinary concentrations of nitrite and nitrate before and after BG and PLA interventions are shown in Table 2. There was no difference in urinary nitrate (*P* = 0.813) and nitrite (*P* = 0.654) between BG and PLA interventions at T0. There was a significant main effect regarding time for nitrate (*P =* 0.006) and nitrite (*P =* 0.007). Post hoc tests revealed that urinary concentrations for nitrate increased more from T0 to T90 (*P =* 0.028), T150 (*P =* 0.004) and T180 (*P =* 0.000) and for nitrite increased more from T0 to T90 (*P =* 0.030), T150 (*P =* 0.028) and T180 (*P =* 0.037) in BG than in PLA. Furthermore, there was a significant interaction effect regarding time per treatment for urinary concentrations of both nitrate (*P =* 0.029) and nitrite (*P =* 0.038). Post hoc tests revealed that urinary concentrations for nitrate increased at T150 (*P =* 0.020) and T180 (*P =* 0.001) and for nitrite increased at T150 (*P =* 0.042) and T180 (*P =* 0.032) in BG compared with PLA intervention. In addition, there was no effect of the supplementation order (BG → PLA vs. PLA → BG) for nitrate (*P* = 0.564) and nitrite (*P* = 0.601).

**Table 2:** Values of nitrate and nitrite in both beetroot-based gel and nitrated-depleted gel conditions.

Variable	Condition	T0	T90	T150	T180
**Nitrate (mmol.mmol creatinine**^**-1**^**)**	BG	0.01 ± 0.01	0.12 ± 0.11^a^	0.21 ± 0.16^a^^,^*	0.21 ± 0.12^a^^,^*
	PLA	0.01 ± 0.00	0.04 ± 0.02	0.05 ± 0.04	0.02 ± 0.02
**Nitrite (mmol.mmol creatinine**^**-1**^**)**	BG	0.98 ± 1.01	2.13 ± 1.45^a^	5.33 ± 6.64^a^^,^*	4.66 ± 5.81^a^^,^*
	PLA	0.83 ± 0.52	1.36 ± 0.71	1.55 ± 0.89	1.10 ± 0.79

The values are mean ± SD. BG = beetroot-based gel, PLA = nitrate-depleted gel.

* Significantly different from PLA.

^a^ Significantly different from T0. Statistical significance was set at the 0.05 level of confidence based on post hoc test with Bonferroni adjustment.

### Muscle oxygenation and blood volume

**Fig 3:**
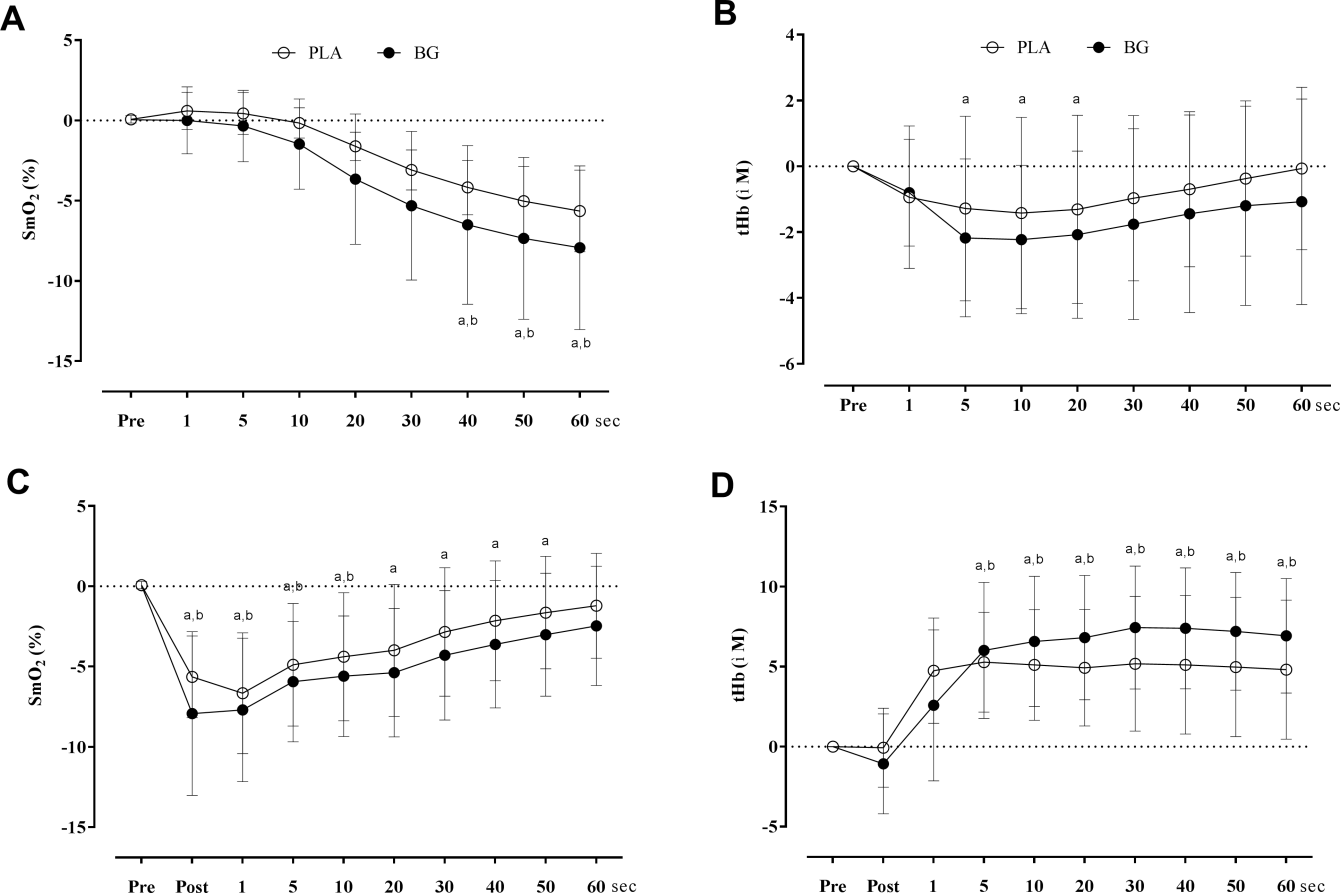
Values of muscle oxygenation (ΔSmO_2_, %) and blood volume (ΔtHb, μM) during (**A** and **B,** respectively) and following (**C** and **D**, respectively) the rhythmic handgrip exercise after beetroot-based nutritional gel (BG) and nitrate-depleted gel (PLA) intervention. Pre = baseline; Post = post-exercise. ^a^ significantly different from Pre for BG and ^b^ significantly different from Pre for PLA.

Changes in SmO_2_ and tHb during the exercise and exercise recovery are depicted in Fig 3. Values of the SmO_2min_, ΔSmO_2½DT_, SmO_2DR_ and SmO_2RR_, ΔtHb_Ex_ and ΔtHb_Rec_ are presented in the Table 3. SmO_2RR_ was greater (*P* = 0.043) in BG as compared to PLA condition and ΔtHb_Rec_ was significantly higher (*P* = 0.050) in BG as compared to PLA. Furthermore, there was a significant decrease in SmO_2min_ (*P* = 0.043) in BG as compared to PLA condition. There was a significant main effect regarding time for SmO_2_ (*P* = 0.000) during exercise. Post hoc tests revealed that SmO_2_ decreased more from T0 to 20 (*P* = 0.019), 30 (*P* = 0.002) and 40 (*P* = 0.001) seconds of exercise in BG than T0 to 20 (*P* = 1.000), 30 (*P* = 0.237) and 40 (*P* = 0.052) in PLA, and decreased more from T0 to 50 and 60 seconds of exercise in both BG (*P* = 0.000 and *P* = 0.000, respectively) and PLA (*P* = 0.017 and *P* = 0.008, respectively). Significant increase in tHb at 5, 10, 20, 30, 40, 50 and 60 seconds of exercise recovery was observed in both BG and PLA as compared to baseline values (main effect for time, *P* = 0.000). No significant differences were observed in tHb (interaction effect for time by treatment, *P* = 0.123) and SmO_2_ (interaction effect for time by treatment, *P* = 0.650) at any time point during exercise recovery, and ΔSmO_2½DT_ (*P* = 0.299) between both BG and PLA conditions. There was no effect of the supplementation order (BG → PLA vs. PLA → BG) for SmO_2_ during exercise (*P* = 0.759), SmO_2_ during recovery (*P* = 0.130), SmO_2min_ (*P* = 0.286), ΔSmO_2½DT_ (*P* = 0.337), ΔSmO_2½RT_ (P = 0.440), ΔtHb_Ex_ (*P* = 0.598) and ΔtHb_Rec_ (*P* = 0.060). The relative changes in oxyhaemoglobin (O_2_Hb) and deoxyhemoglobin (HHb) during exercise and recovery period for both BG and PLA conditions was included as Supplementary Materials (S1 and S2 Tables).

**Table 3:** Changes in muscle oxygenation and blood volume parameters.

	BG	PLA
**SmO**_**2min**_ **(%)**	- 13.68 ± 6.77*	- 10.70 ± 5.02
Δ**SmO**_**2½DT**_ **(s)**	15.92 ± 7.25	18.42 ± 7.48
**SmO**_**2DR**_ **(%.s**^**-1**^**)**	-1.90 ± 0.94	-1.72 ± 0.88
**SmO**_**2RR**_ **(%.s**^**-1**^**)**	1.43 ± 0.77*	1.02 ± 0.48
Δ**tHb**_**Ex**_ **(μM)**	10.48 ± 5.83	9.99 ± 3.39
Δ**tHb**_**Rec**_ **(μM)**	10.25 ± 5.47*	6.72 ± 4.55

Values are expressed as means ± SD. BG = beetroot-based nutritional gel, PLA = nitrate-depleted gel, SmO_2_ = muscle O_2_ saturation, tHb = blood volume.

* Significantly different from PLA.

### Forearm muscle strength

**Fig 4:**
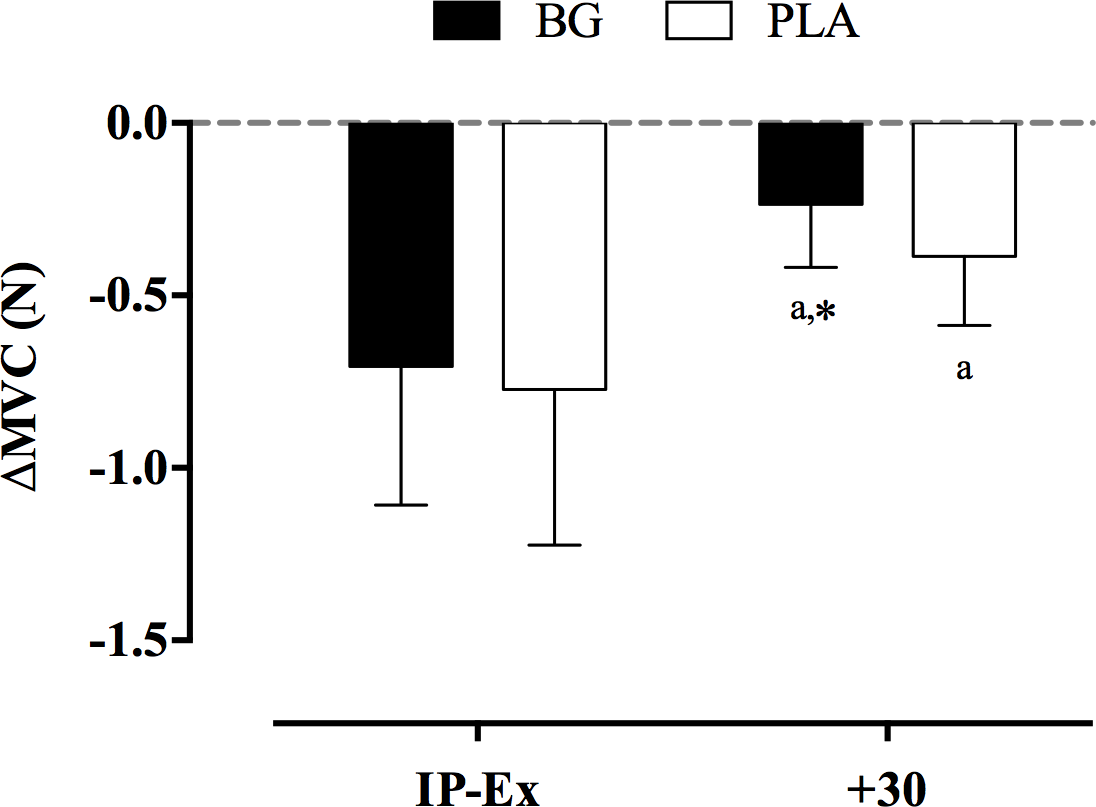
Changes from baseline in maximal voluntary contraction (ΔMVC, N) of the forearm muscle immediately after exercise (IP-Ex) and 30 minutes of recovery (+30) after beetroot-based nutritional gel (BG) and nitrate-depleted gel (PLA) intervention. The symbol*(P < 0.05) denotes significantly different from PLA and ^a^ significantly different from T0.

Changes from baseline in maximal voluntary contraction (ΔMVC) of the forearm muscle immediately after exercise (IP-Ex) and 30 minutes of recovery (+30) in the BG and PLA conditions are presented in Fig 4 and Table 4. No significant difference between BG and PLA conditions were observed for MVC at T0 (*P* = 0.982). There was a significant decrease in MVC from T0 to IP-Ex in both BG and PLA condition (main effect for time, both *P* = 0.000). There was a significant reduction in ΔMVC at +30 in the BG when compared to PLA condition (interaction effect for time by treatment, *P* = 0.050). No effect of the supplementation order (BG → PLA vs. PLA → BG) for MVC was observed (*P* = 0.116).

**Table 4:** Absolute values of maximal voluntary contraction (MVC) and difference (ΔMVC) from baseline (T0) immediately after exercise (IP-Ex) and 30 minutes of recovery (+30) after beetroot-based nutritional gel (BG) and nitrate-depleted gel (PLA) intervention.

Variable	Condition	T0	IP-Ex	+30
**MVC (N)**	BG	3.69±0.90	2.99±0.71^a^	3.46±0.80*
	PLA	3.69±0.81	2.91±0.59^a^	3.30±0.68
**ΔMVC (N)**	BG	-	-0.71±0.40	-0.24±0.18^b,^*
	PLA	-	-0.77±0.45	-0.39±0.20^b^

The values are mean ± SD.

The symbol*(P < 0.05) denotes significantly different from PLA.

The letters ^a^ denotes significantly different from T0 and

^b^ denotes significantly different from IP-Ex.

## Discussion

The aim of this study was to examine the effects of a single dose of a beetroot-based nutritional gel, which is rich in nitrate, on forearm muscle O_2_ saturation, on blood volume and forearm strength following the completion of a handgrip exercise protocol in the elderly. This is the first investigation into the effects of the single dose of beetroot-based nutritional gel on SmO_2_ parameters (forearm muscle O_2_ desaturation [ΔSmO_2min_, SmO_2DR_ and ΔSmO_2DT_] and O_2_ resaturation rate (SmO_2RR_) and blood volume. The major findings of this study are that a single dose of a beetroot-based nutritional gel rich in nitrate increase muscle O_2_ extraction (SmO_2min_), speeds up muscle O_2_ resaturation rate (SmO_2RR_) during exercise recovery and speeds up the force recovery of handgrip strength after 30 minutes of exercise recovery. These results were associated with an increase in the muscle blood volume (ΔtHb_Rec_) and urinary nitrite and nitrate, which increased ~3.5-fold and ~4.0-fold, respectively after 150 minutes and ~4.0-fold and ~10.0-fold after 180 minutes, respectively in BG as compared to PLA. Others studies have observed similar increases in plasma nitrite and nitrate 180 minutes after beetroot juice consumption [21,22], demonstrating that plasma nitrate and nitrite have a similar kinetic effect as observed in the urine nitrite and nitrate of the present study.

Collectively, these findings suggest that the beetroot gel promotes NO bioconversion, which may increase blood and oxygen delivery to active muscle and thereby speed up the force recovery of handgrip strength.

NO bioavailability is compromised in the systemic circulation and in the musculature of sedentary ageing humans due to increased oxidative stress [23]. In the current study, significant increases in urinary nitrate and nitrite concentrations were observed after approximately 2.5 h after BG when compared to PLA condition. These finding support the results of other study where significant increases were also observed in older people after a single dose of BG [11] and may suggest that there was absorption and a possible bioconversion of the dietary nitrate present in the BG into nitrite and, subsequently NO.

An association between ageing and reduced blood flow and O_2_ delivery to the active muscle in aged subjects has been documented [24,25]. Although a myriad of factors (*e*.*g*.: prostacyglin and endotelin-1) that act regulating smooth vascular muscle tonus are impaired in elderly [26], the mechanisms underlying the attenuated local vasodilatory response can be explained in part by impaired NO availability [25]. NO may influence O_2_ utilization by the cells, suggesting that O_2_ delivery to the working muscle during exercise may be compromised in the elderly. In this scenario, improved NO bioconversion would likely increase blood flow and O_2_ delivery to the contracting muscles. Ichimura et al. [4] observed prolongation of muscle oxygenation in aging people, suggesting a decline in muscle oxidative capacity and impairs peripheral endothelial function in this population. In the present study, SmO_2RR_ was faster (*P* = 0.043) in BG as compared to PLA, along with a more pronounced blood volume increase during recovery (ΔtHb_Rec_), which indicates a greater O_2_ delivery relative to O_2_ consumption. However, no significant difference in SmO_2_ was observed at the end of the 60 seconds of exercise recovery, which suggests that BG may improve O_2_ delivery only in the initial period of exercise recovery (~10 sec). Previous studies have also demonstrated improvements in muscle oxygenation status during moderate to severe exercise in healthy adults after beetroot consumption [7,8,9,10]. This beneficial effect is probably at least partly explained by the improved bioconversion of the dietary nitrate present in the BG into NO, which have an important role in modulating vascular tone and blood perfusion. We recently demonstrated that there was an improved endothelium-dependent vasodilation after a single dose of beetroot based gel in the elderly with cardiovascular risk factors [11]. Endothelium-dependent vasodilation has been used as a physiological measurement for evaluating NO production [27]. Therefore, this finding corroborates a possible effect of beetroot on bioconversion of NO, as encountered in the present study.

Ageing has been associated with a reduced muscle O_2_ extraction during exercise [23], which may limit maximal oxygen consumption and contribute to reduced muscle energy capacity [28]. Although no significant difference was observed in ΔSmO_2½DT_ (*P* = 0.299) and SmO_2DR_ (*P* = 0.374) in the present study, there was a significant decrease in SmO_2min_ (*P* = 0.043) and SmO_2_ during the handgrip exercise in BG as compared to PLA condition. The lesser SmO_2min_ observed in BG, together with no significant change in ΔtHb during exercise, may indicate a greater muscle O_2_ consumption of exercising muscle. This finding suggest that beetroot consumption may improve mitochondrial function in older people. Previous *in vitro* study has demonstrated that beetroot induce mitochondrial biogenesis and modestly increases basal cellular respiration without affecting respiratory capacity and proton leak [29]. Larsen et al. [30] demonstrated that nitrate supplementation increased mitochondrial respiratory control ratio and P/O ratio during submaximal ADP stimulation in isolated mitochondria of muscle tissue of healthy adults, indicating a better coupling between respiration and oxidative phosphorylation, and an improved mitochondrial efficiency, respectively. The effect of beetroot on the mitochondria may be mediated partially by the bioconversion of the nitrate present in this food into nitrite and NO, which has been demonstrated to be a key messenger to regulate mitochondrial number and function in various cell types [31,32]. The ΔSmO_2½DT_ and SmO_2DR_ represents a O_2_ consumption rate immediately after beginning the exercise. The absence of the effect observed in these variables may be related to muscle metabolic condition (i.e. changes in pH and O_2_ pressure). For example, nitrite-NO bioconversion is favored in fatiguing muscle during exercise and perhaps no differences were shown in ΔSmO_2½DT_ and SmO_2DR_ because muscle fatigue in the beginning of the exercise was not sufficient to generate adequate muscle metabolic condition for nitrite-NO bioconversion.

A previous study in healthy, moderately active subjects observed that a higher magnitude of deoxygenation during isometric exercise (oxidative capacity) shows slower time for muscle reoxygenation during exercise recovery, since O_2_ supply is not fully increased despite the increased availability of O_2_ [33]. Although improved muscle oxidative capacity during handgrip exercise after BG consumption (lesser SmO_2min_) has been observed, there was a faster O_2_ resaturation rate during the exercise recovery (greater SmO_2RR_), which suggests that BG consumption improves muscle oxidative capacity and O_2_ supply in the elderly. In sedentary healthy subjects, muscle reoxygenation rate during bicycle ergometer exercise recovery has been shown to be approximately 22% faster in middle-age subjects when compared with the elderly [4]. Additionally, the muscle reoxygenation rate during exercise recovery was approximately 35% faster in physically active when compared with sedentary elderly, and 37% faster in physically active when compared to sedentary middle-age subjects [4]. Our results have demonstrated that SmO_2RR_ during the handgrip exercise recovery was 40% faster after BG when compared with PLA condition in the sedentary elderly. Hence, it is important to point out that habitual physical activity and BG condition, albeit single dose consumption, seems speed up of muscle O_2_ resaturation rate during exercise recovery.

Another important finding from this study is that BG promoted faster recovery from the decrease in maximal isometric strength observed after handgrip exercise. Good handgrip strength in older people is important for successful performance in activities of daily living and occupational activities. The energy pathway predominantly involved in the MVC is the phosphagen system. Because the need for large amounts of adenosine triphosphate (ATP) is so urgent, the ATP and phosphocreatine (PCr) must be immediately available for the interacting muscle filaments–actin and myosin. A possible explanation for the effect of BG in speeding up force recovery of handgrip strength could be increased resynthesis of PCr and ATP. Previous studies have reported a positive association between faster muscle O_2_ resaturation measured by NIRS and faster PCr resynthesis after exercise [34,35,36,37]. McCully et al. [38] simultaneously measured and PCr recovery after submaximal exercise in normal subjects using NIRS and phosphorus magnetic resonance spectroscopy, respectively, and found that the time constants of these indices were similar. The authors suggested that the muscle O_2_ resaturation is rate limiting for ATP synthesis, when evaluated as the rate of PCr recovery after submaximal exercise. Furthermore, nitrate increases the rate of human skeletal muscle PCr recovery after exercise in hypoxia, suggesting an augmented maximum rate of oxidative ATP synthesis [39], and it lowers the ATP cost of contractile force production [40]. Thus, the greater SmO_2RR_ observed in the present study suggests that beetroot consumption may have improved PCr resynthesis and, consequently, speed up muscle strength recovery. On the other hand, Siervo et al. (2016) [41] did not observe any significant effect on MVC after 7 days of beetroot juice consumption (~12 mmol/140mL). It is worth mentioning that the authors did not evaluate MVC in response to a high-intensity rhythmic handgrip exercise as has been done in the present study.

In summary, we investigated the effects of a single dose of a beetroot-based nutritional gel, rich in dietary nitrate, on SmO_2_, blood volume, and forearm muscle strength in older adults. The results of the present study suggest that the dietary nitrate improves SmO_2_ status of the forearm in older adults by increasing muscle O_2_ extraction during handgrip exercise and by speeding up muscle O_2_ resaturation during exercise recovery, thereby preventing the age-related prolongation in post-exercise recovery time of muscle O_2_ resaturation and improving the muscle function.

## Supporting information

S1 File Analysis of residuals for SmO_2_ and tHb during exercise and exercise recovery. (PDF)

S1 Table Changes in muscle O_2_Hb and HHb during handgrip exercise and during exercise recovery. Values are expressed as means ± SD. BG = beetroot-based nutritional gel, PLA = nitrate-depleted gel, O_2_Hb = muscle oxyhaemoglobin, HHb = muscle deoxyhaemoglobin. * Significantly different from PLA. ^a^ significantly different from Pre.
(DOCX)

S2 Table Changes in muscle O_2_Hb parameters during handgrip exercise and during exercise recovery. Values are expressed as means ± SD. BG = beetroot-based nutritional gel, PLA = nitrate-depleted gel, O_2_Hb = muscle oxyhaemoglobin. * Significantly different from PLA.
(DOCX)
