# Supplementary material for: Acute effect of dietary nitrate on forearm muscle oxygenation, blood volume and strength in older adults: A randomized clinical trial
Source: PLoS One. 2017 Nov 30;12(11):e0188893. doi: 10.1371/journal.pone.0188893 (PMC5708833; doi:10.1371/journal.pone.0188893)

Normal Q-Q Plots of Standardized Residual for SmO_2 During Exercise

Normal Q-Q Plot of Standardized Residual for Pre

Treatment= BG

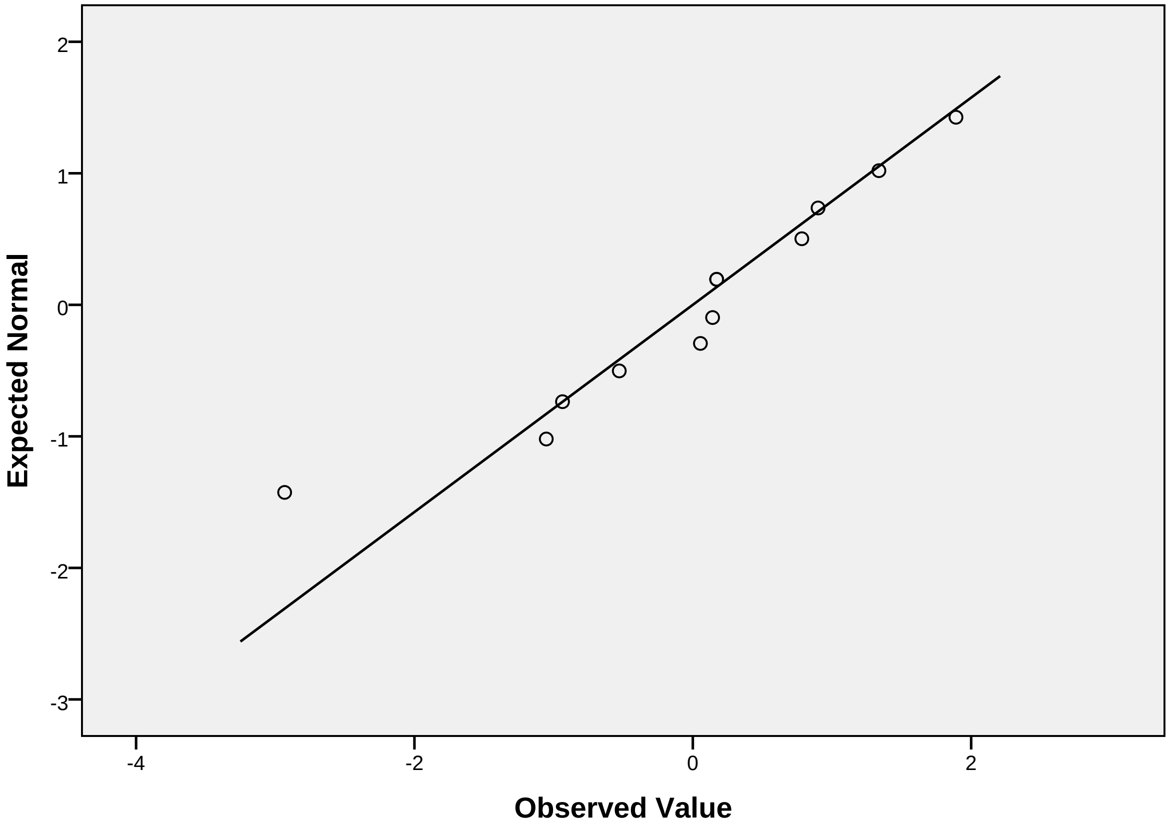

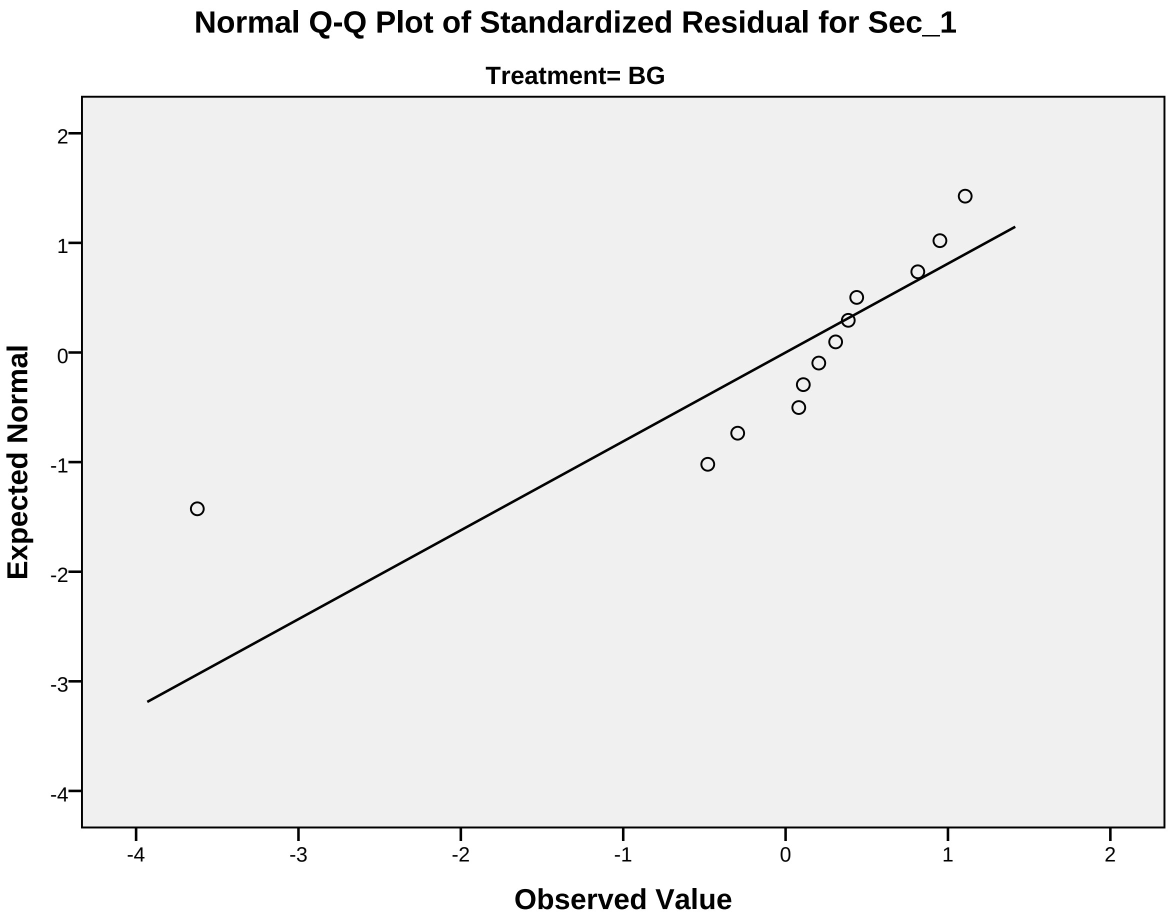
Normal Q-Q Plot of Standardized Residual for Sec_1
Treatment= BG
Expected Normal
2
1
0
-1
-2
-3
-4
-4
-3
-2
-1
0
1
2
Observed Value

Normal Q-Q Plot of Standardized Residual for Sec_5

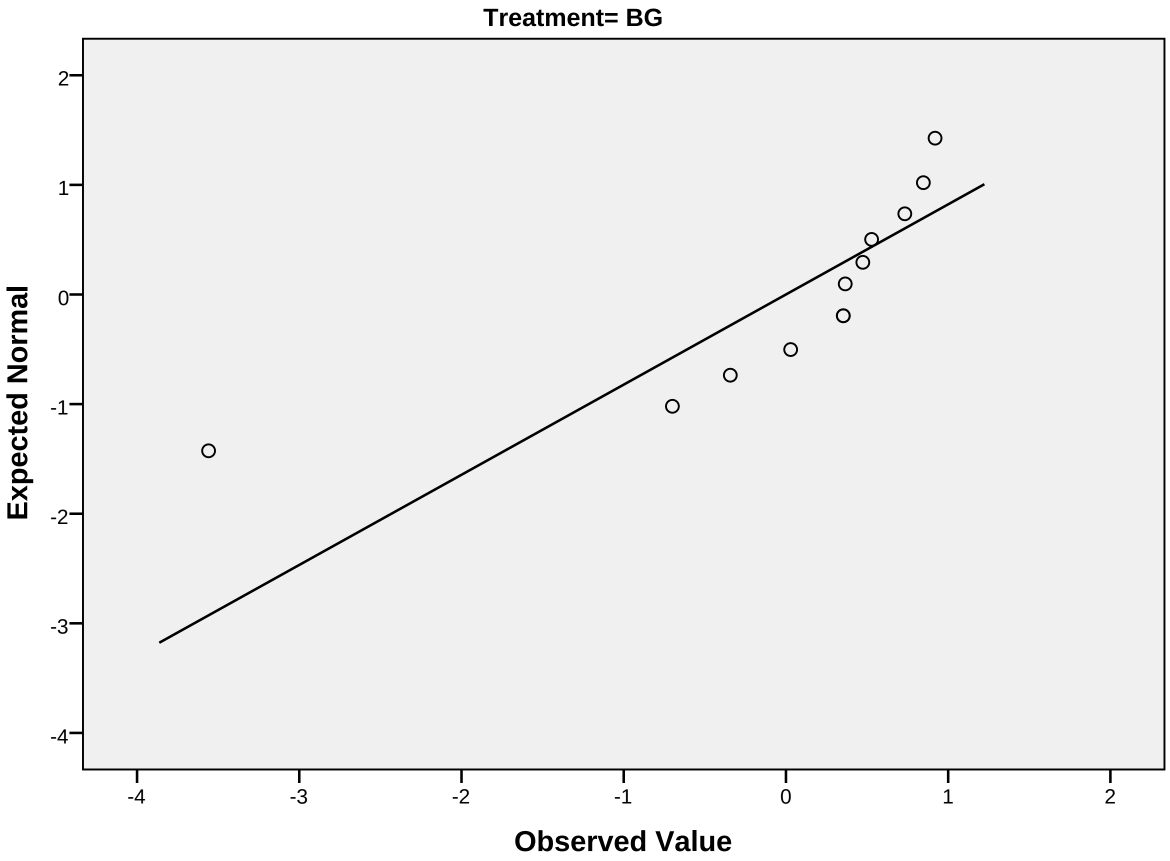

Normal Q-Q Plot of Standardized Residual for Sec_10

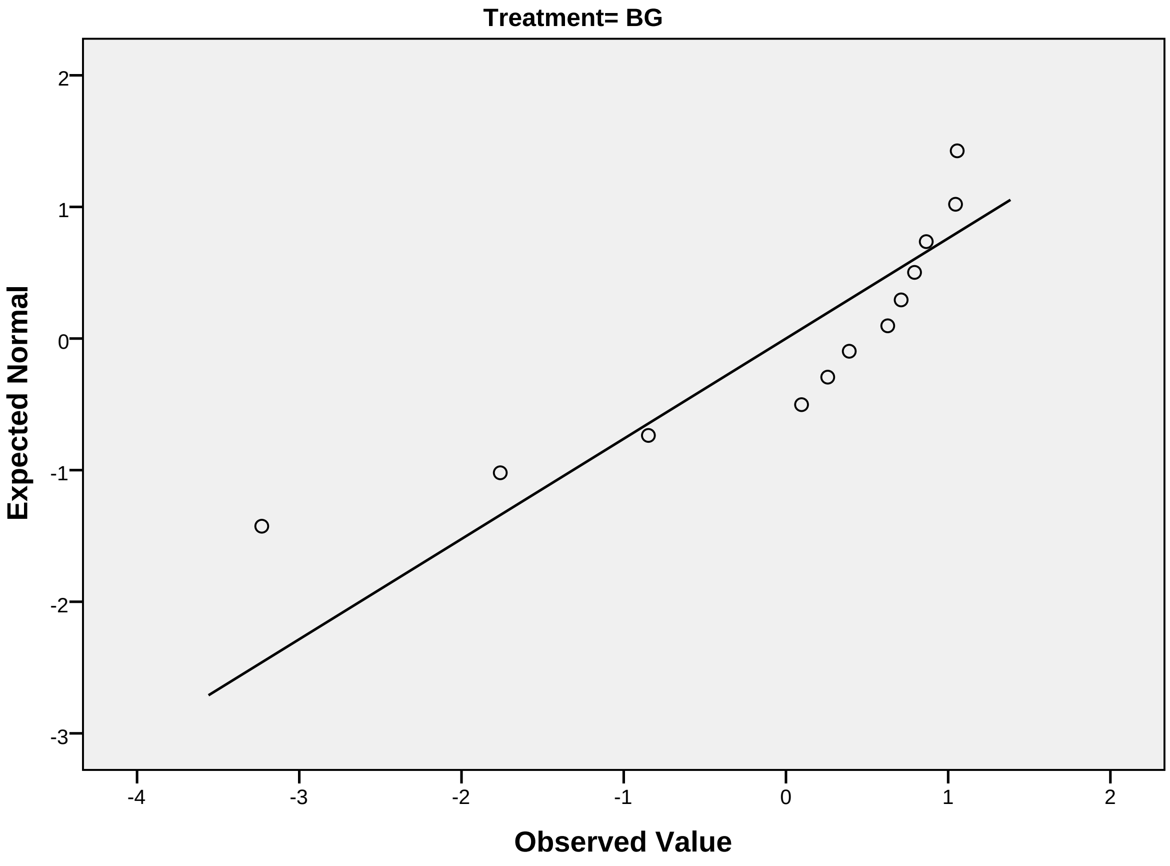

Normal Q-Q Plot of Standardized Residual for Sec_20

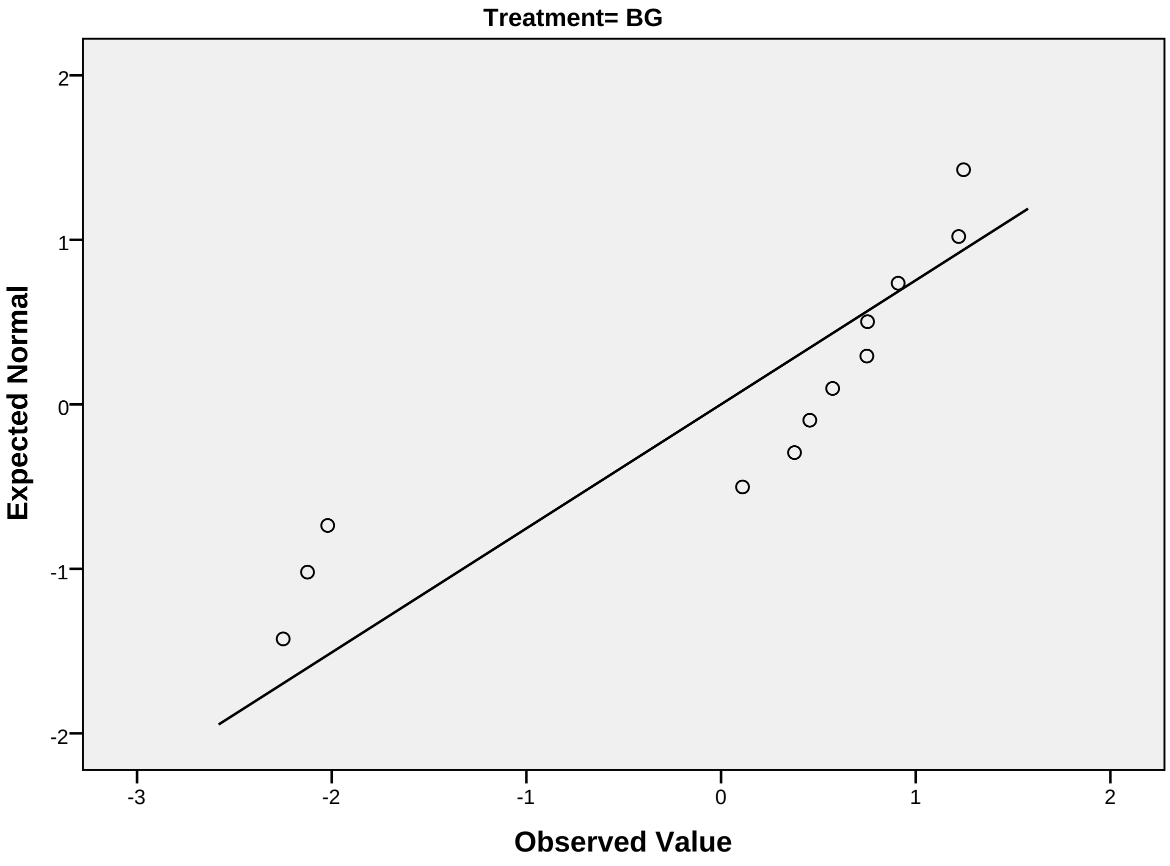

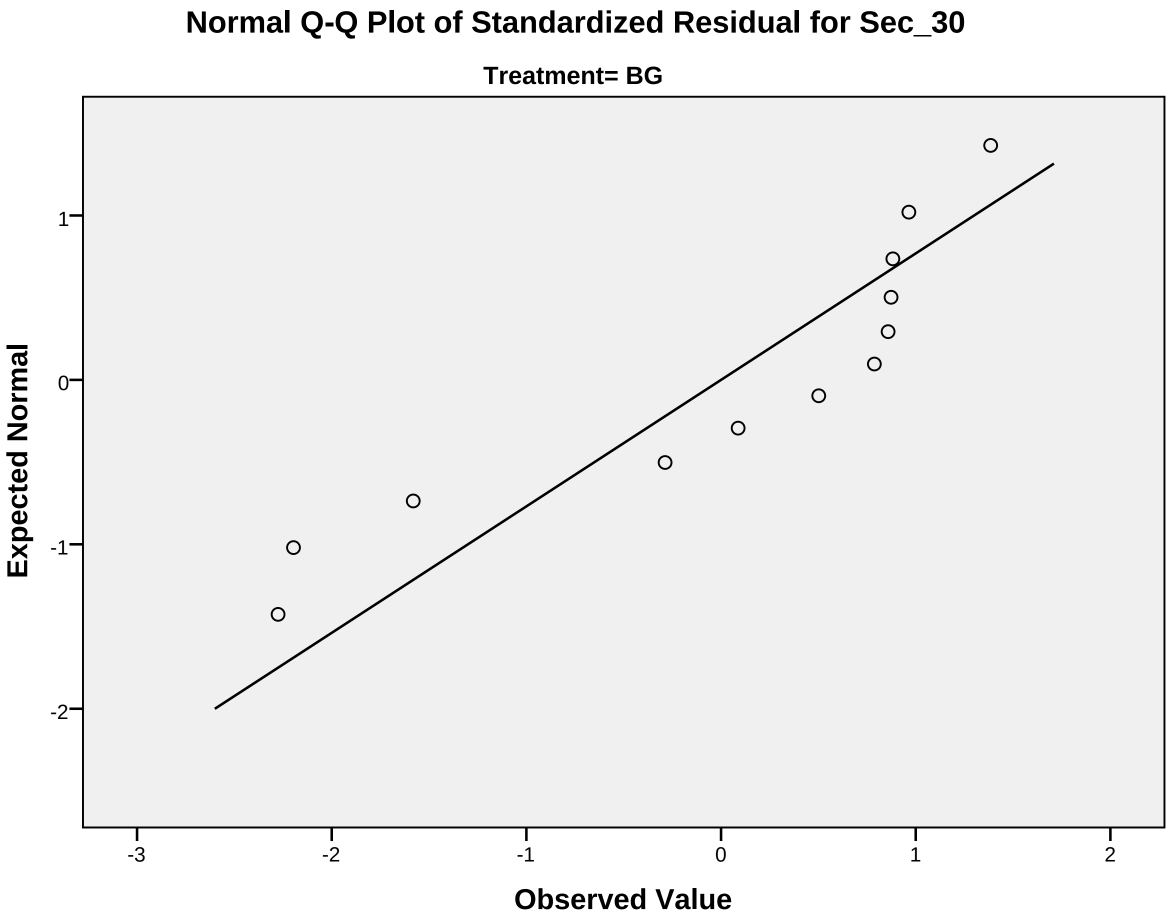
Normal Q-Q Plot of Standardized Residual for Sec_30
Treatment= BG
Expected Normal
1
0
-1
-2
-3
-2
-1
0
1
2
Observed Value

Normal Q-Q Plot of Standardized Residual for Sec_40

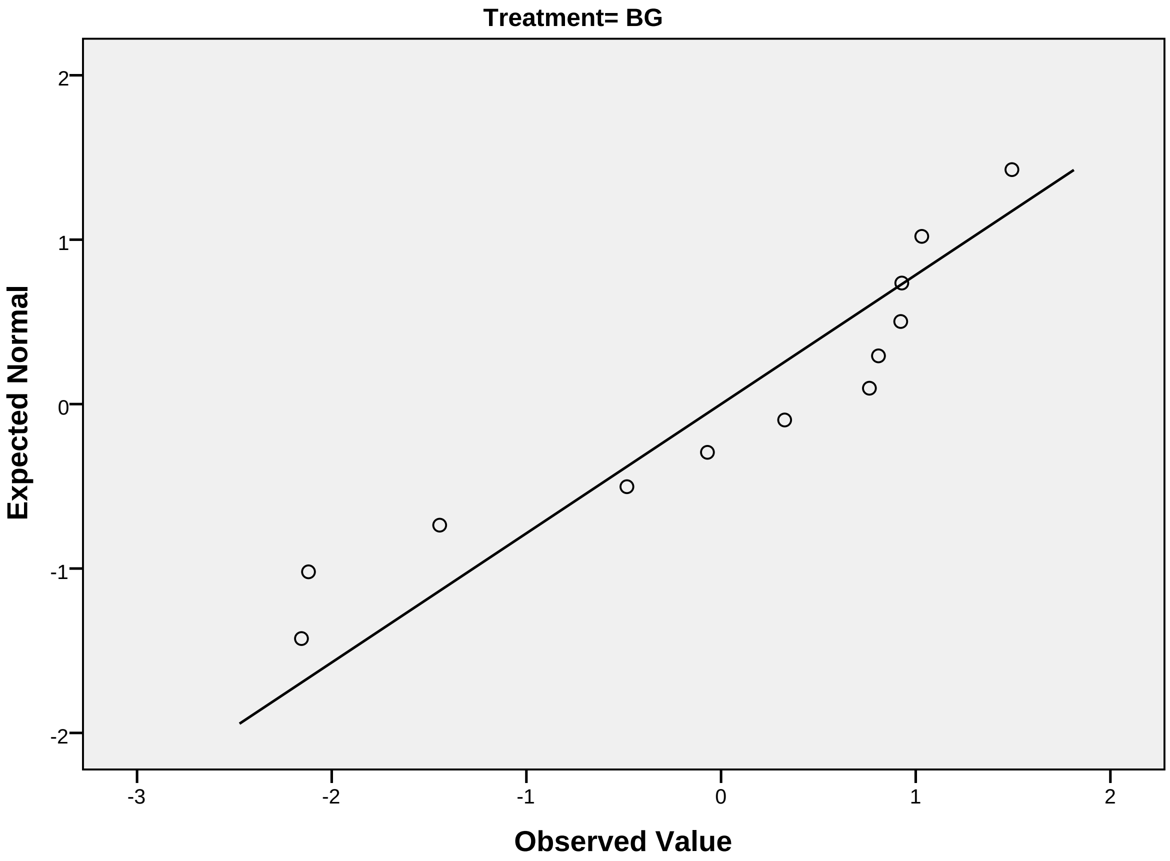

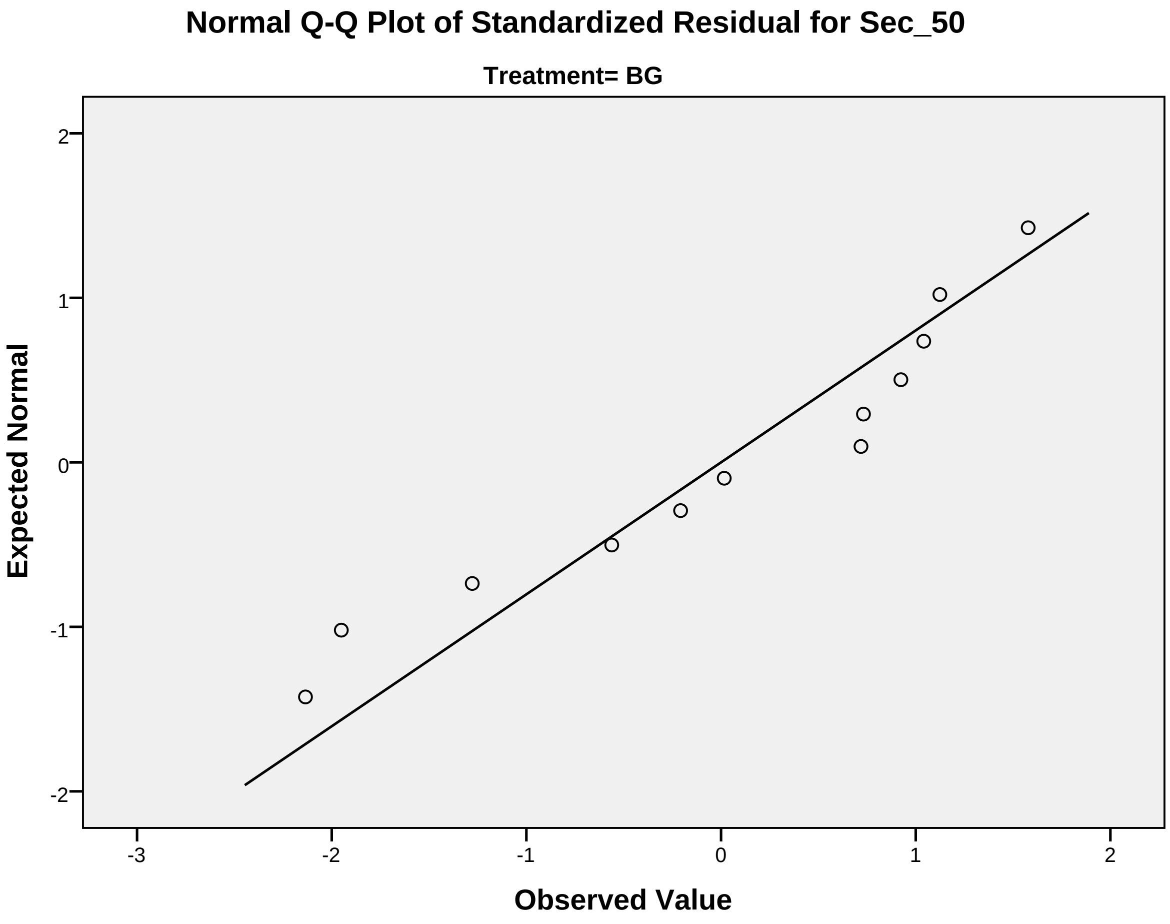
Normal Q-Q Plot of Standardized Residual for Sec_50
Treatment= BG
Expected Normal
Observed Value
2
1
0
-1
-2
-3
-2
-1
0
1
2

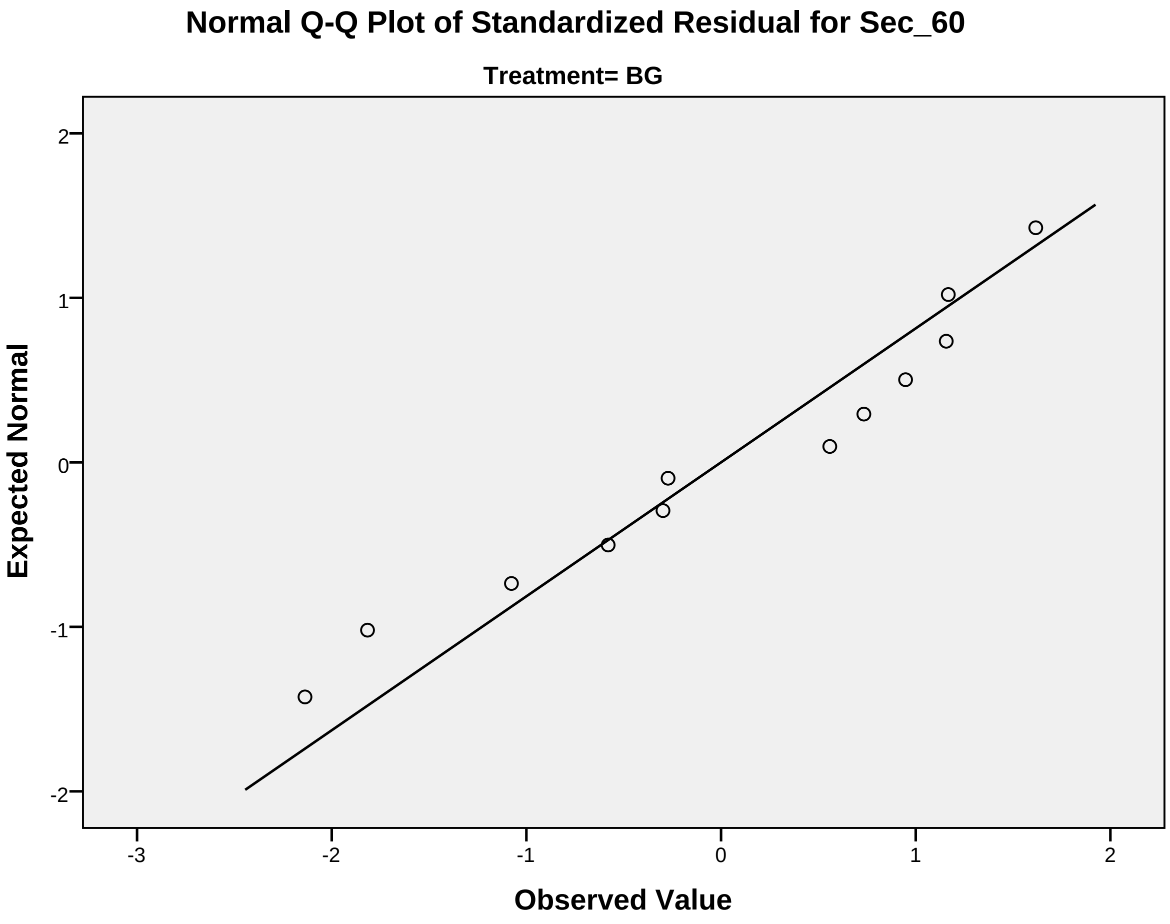

Detrended Normal Q-Q Plots for SmO_2 During Exercise

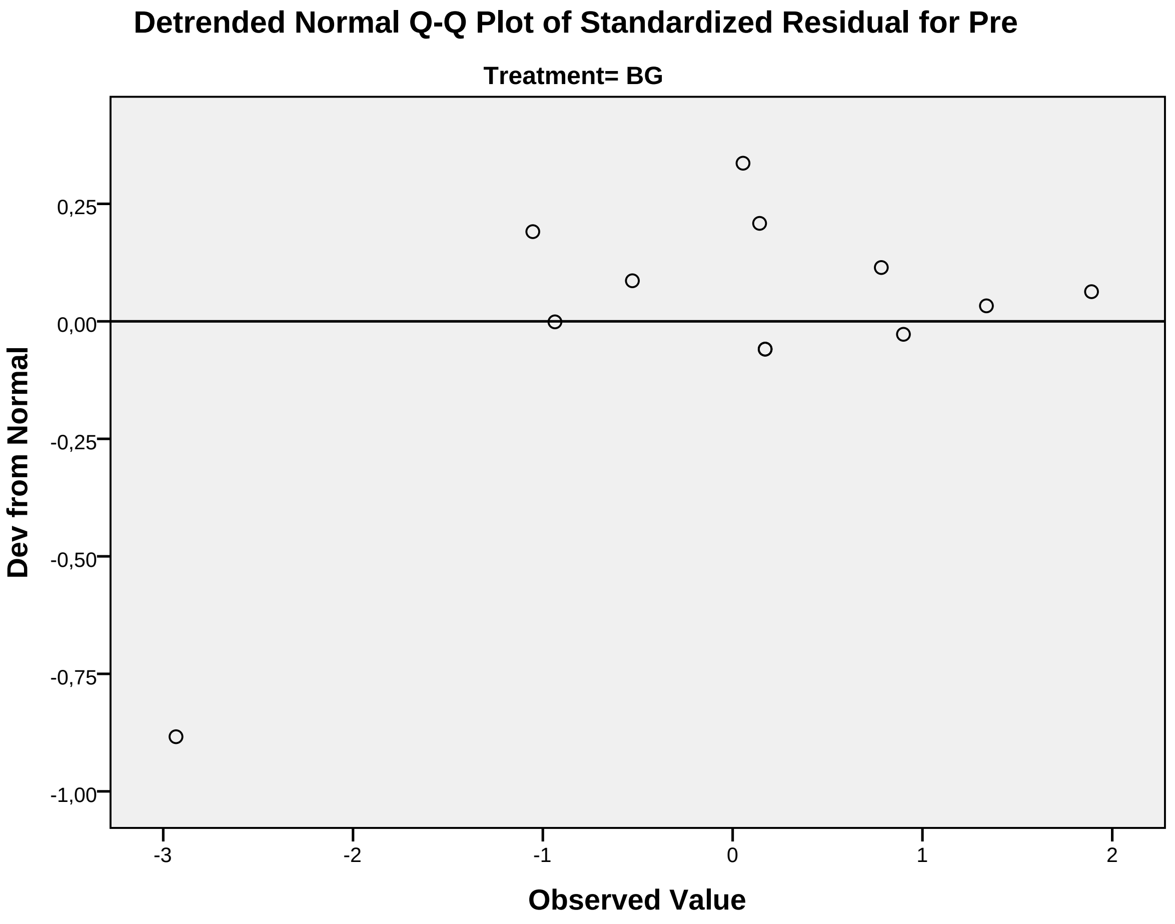

Detrended Normal Q-Q Plot of Standardized Residual for Pre
Treatment= BG
Dev from Normal
0,25
0,00
-0,25
-0,50
-0,75
-1,00
-3
-2
-1
0
1
2
Observed Value

Detrended Normal Q-Q Plot of Standardized Residual for Sec_1

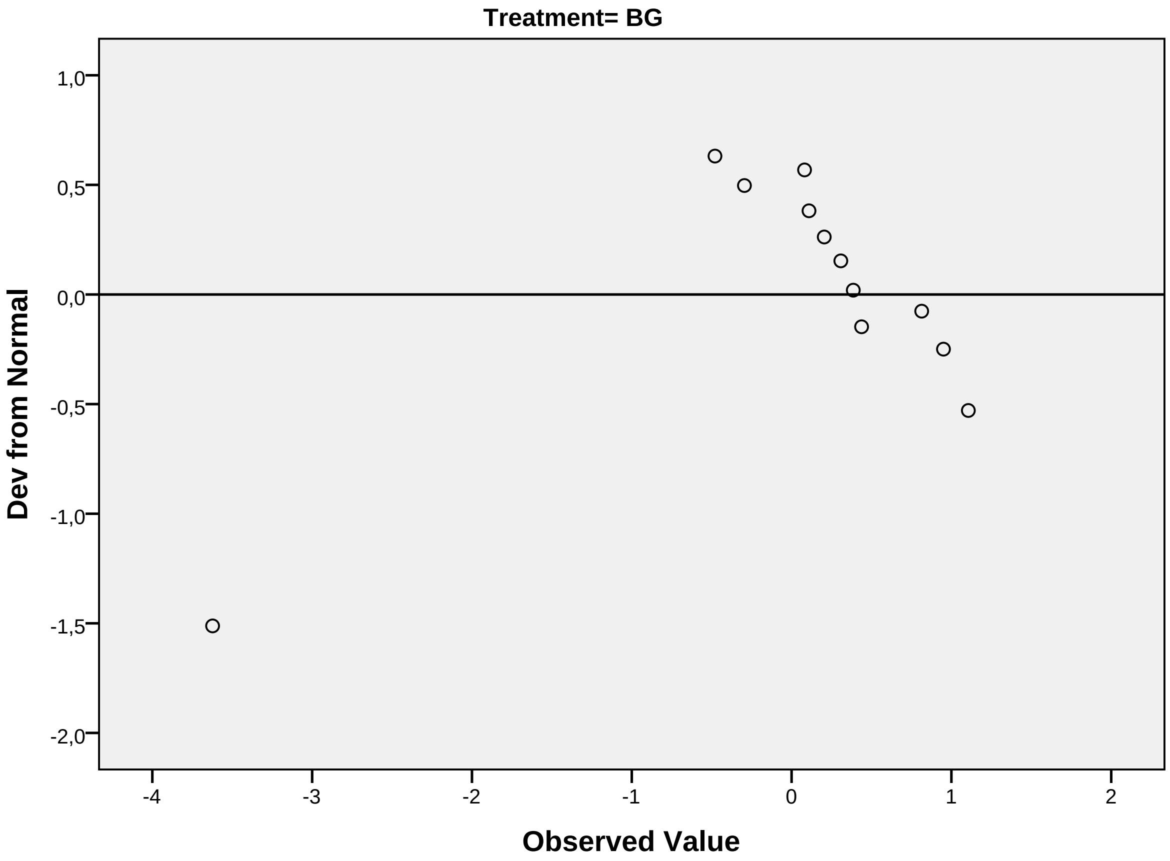

Detrended Normal Q-Q Plot of Standardized Residual for Sec_5

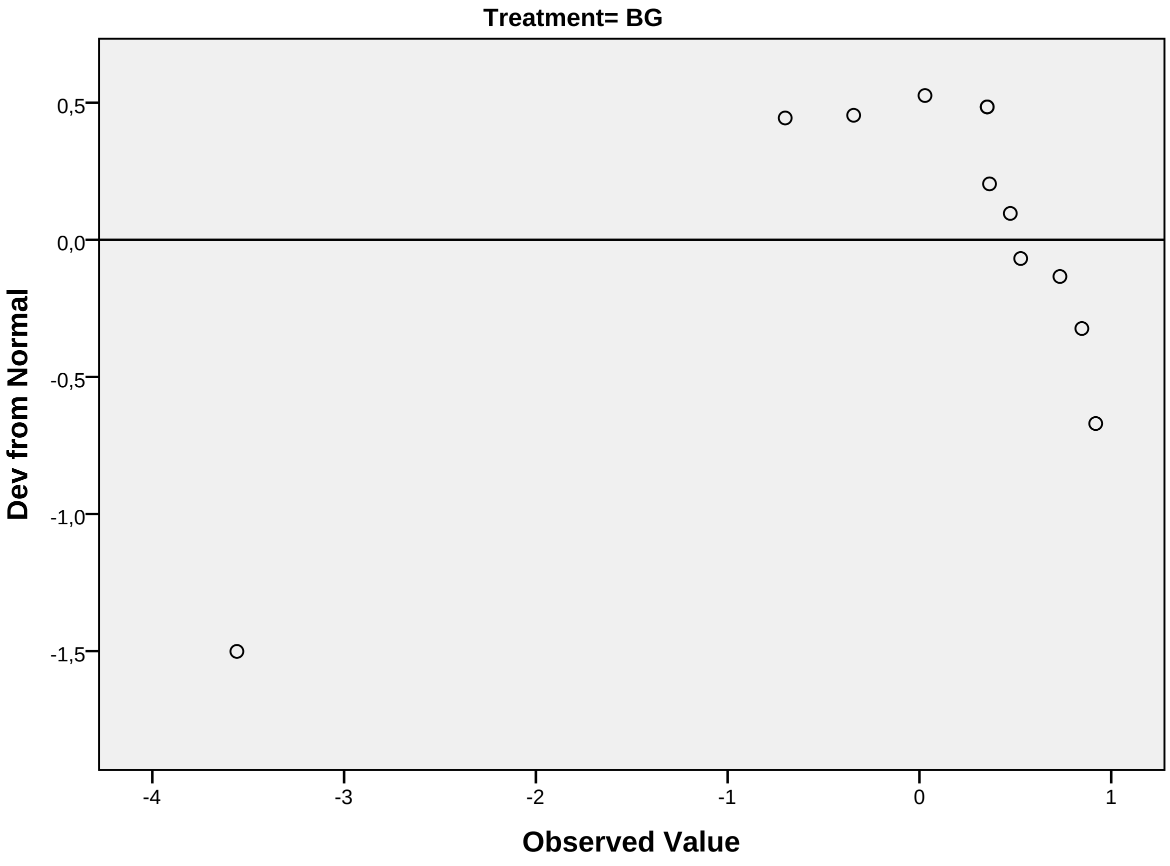

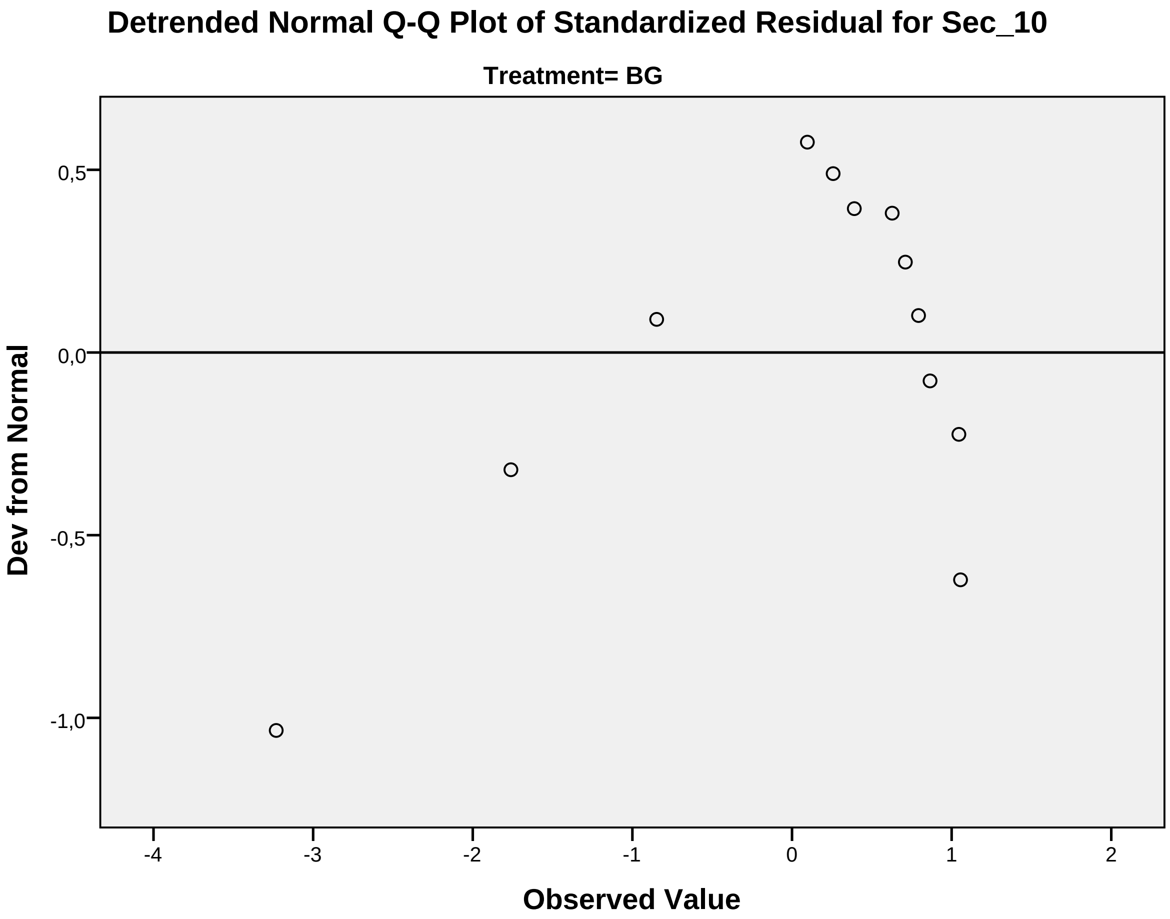
Detrended Normal Q-Q Plot of Standardized Residual for Sec_10
Treatment= BG
Dev from Normal
0,5
0,0
-0,5
-1,0
-4
-3
-2
-1
0
1
2
Observed Value

Detrended Normal Q-Q Plot of Standardized Residual for Sec_20

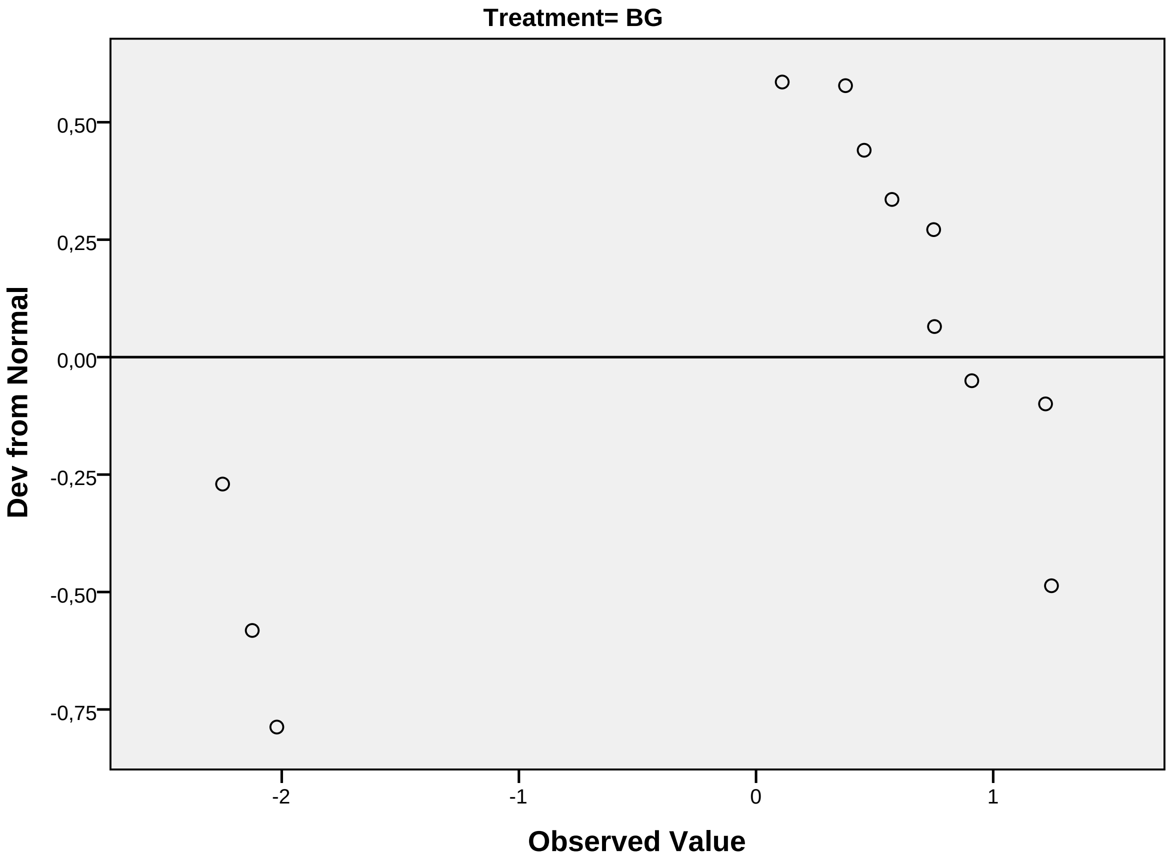

Detrended Normal Q-Q Plot of Standardized Residual for Sec_30

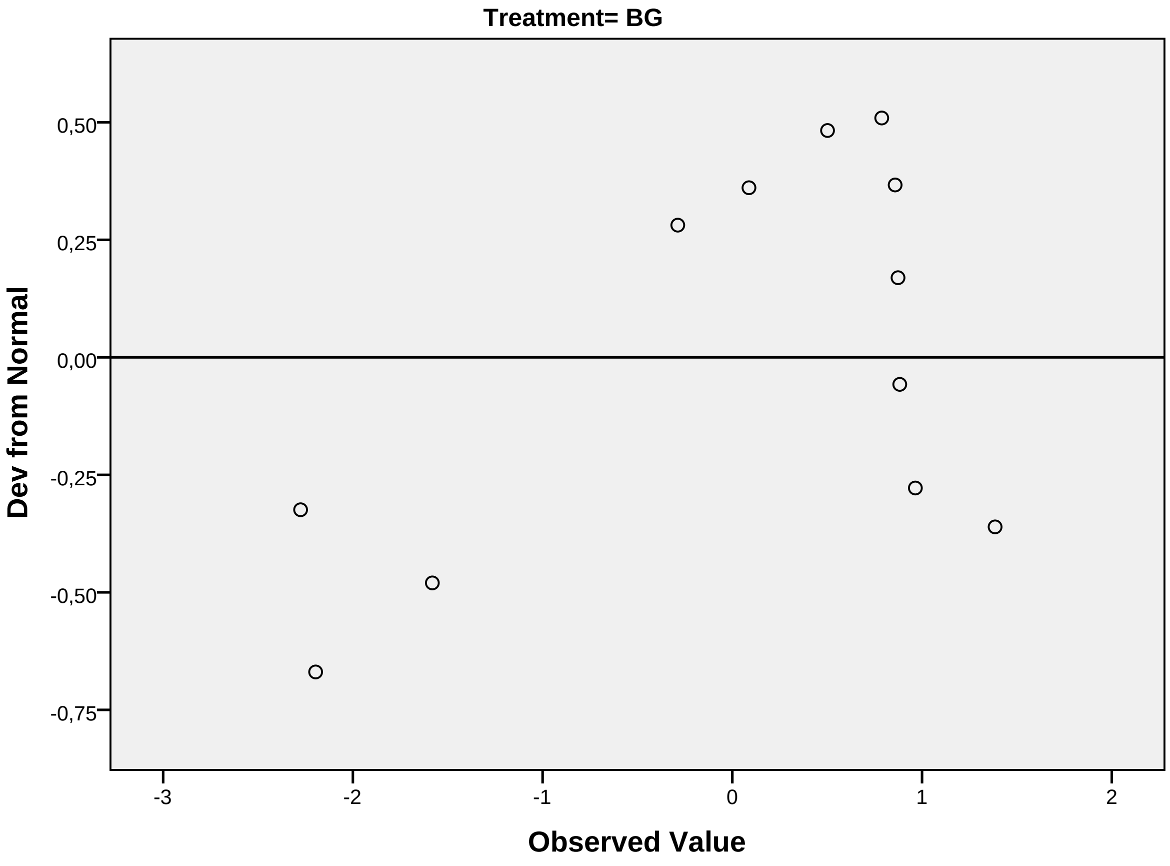

Detrended Normal Q-Q Plot of Standardized Residual for Sec_40

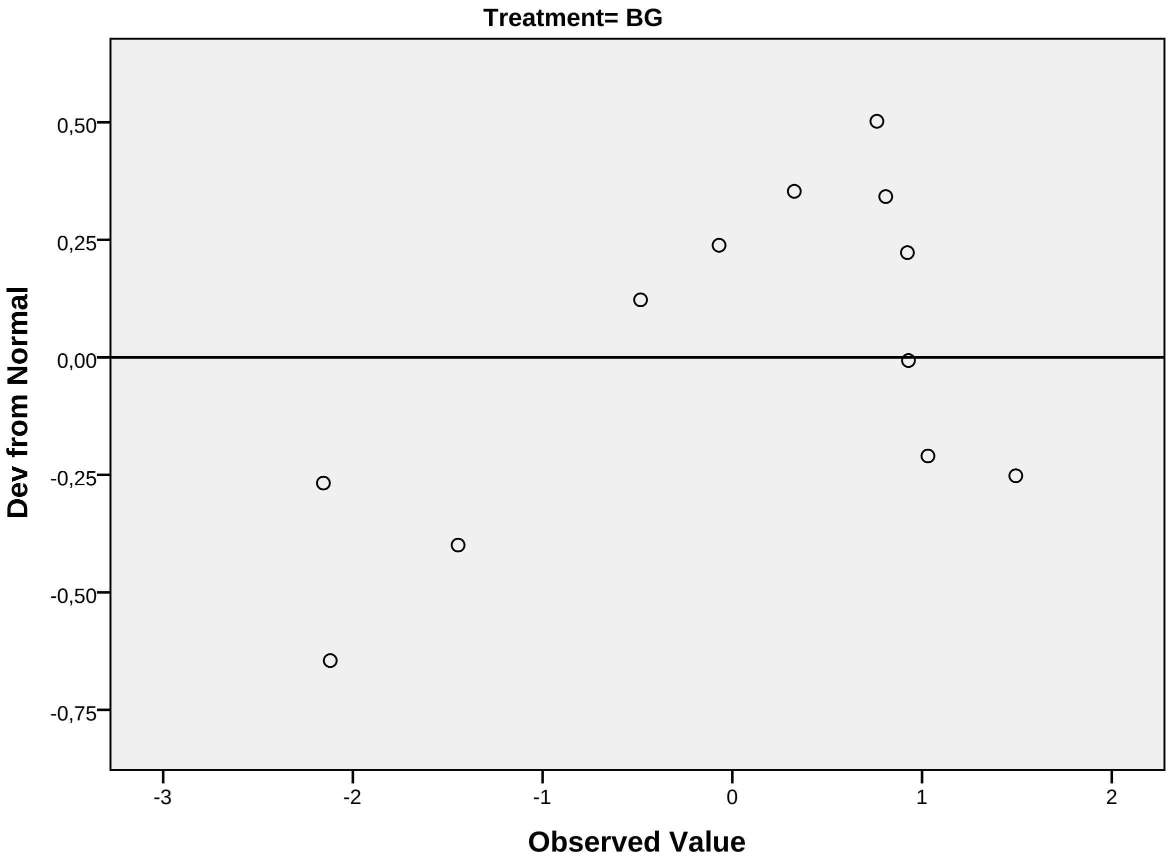

Detrended Normal Q-Q Plot of Standardized Residual for Sec_50

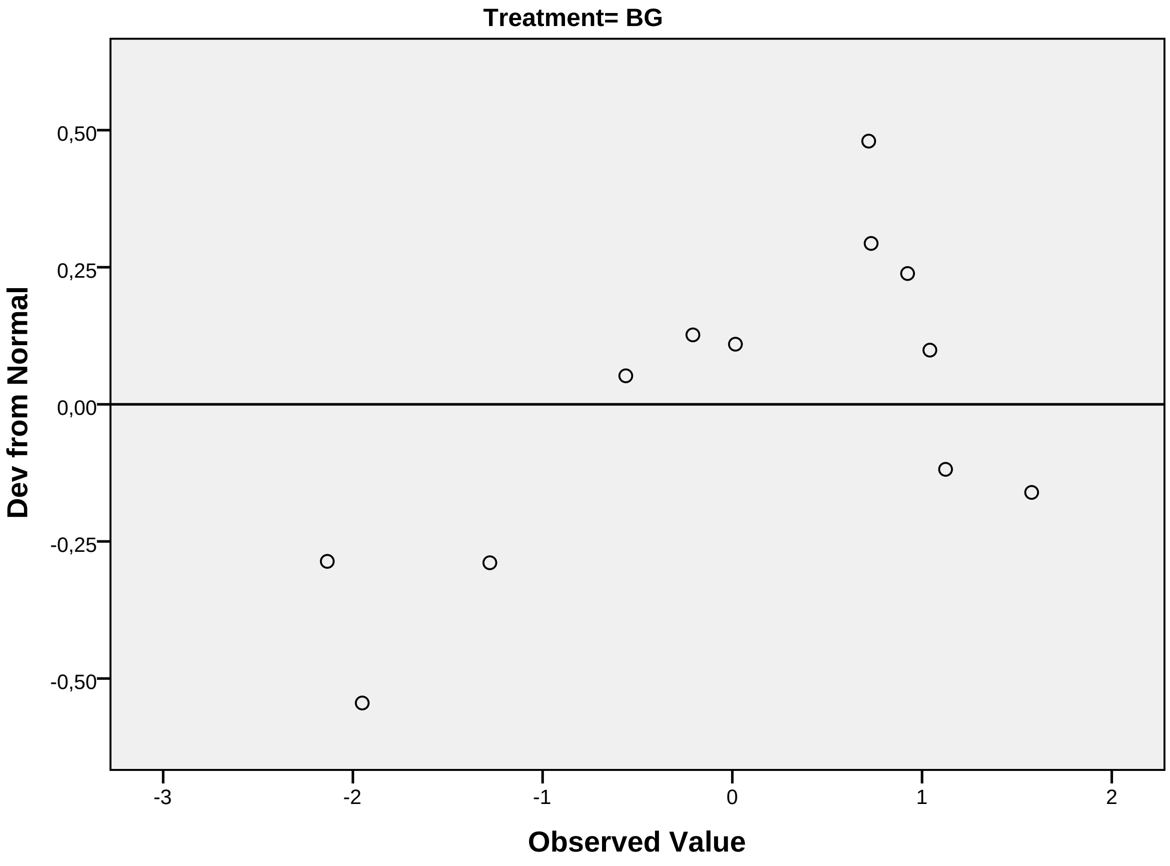

Detrended Normal Q-Q Plot of Standardized Residual for Sec_60

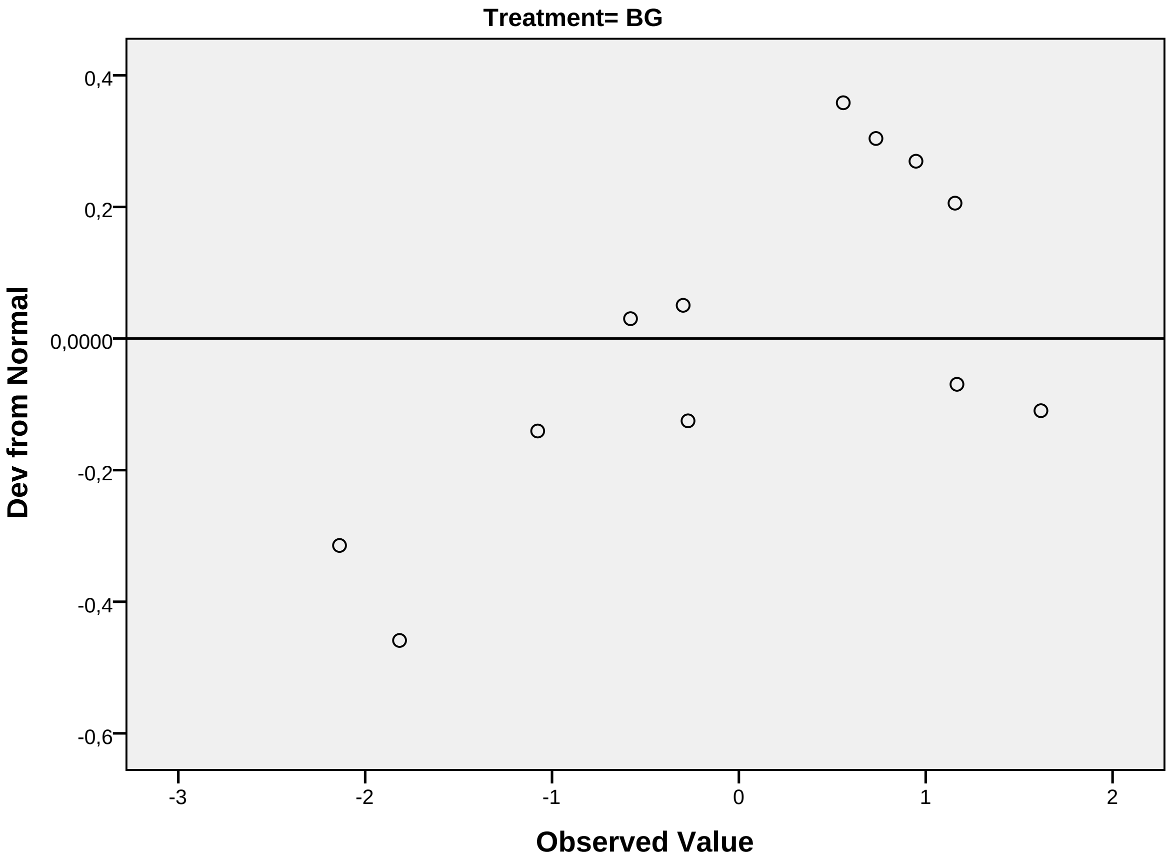

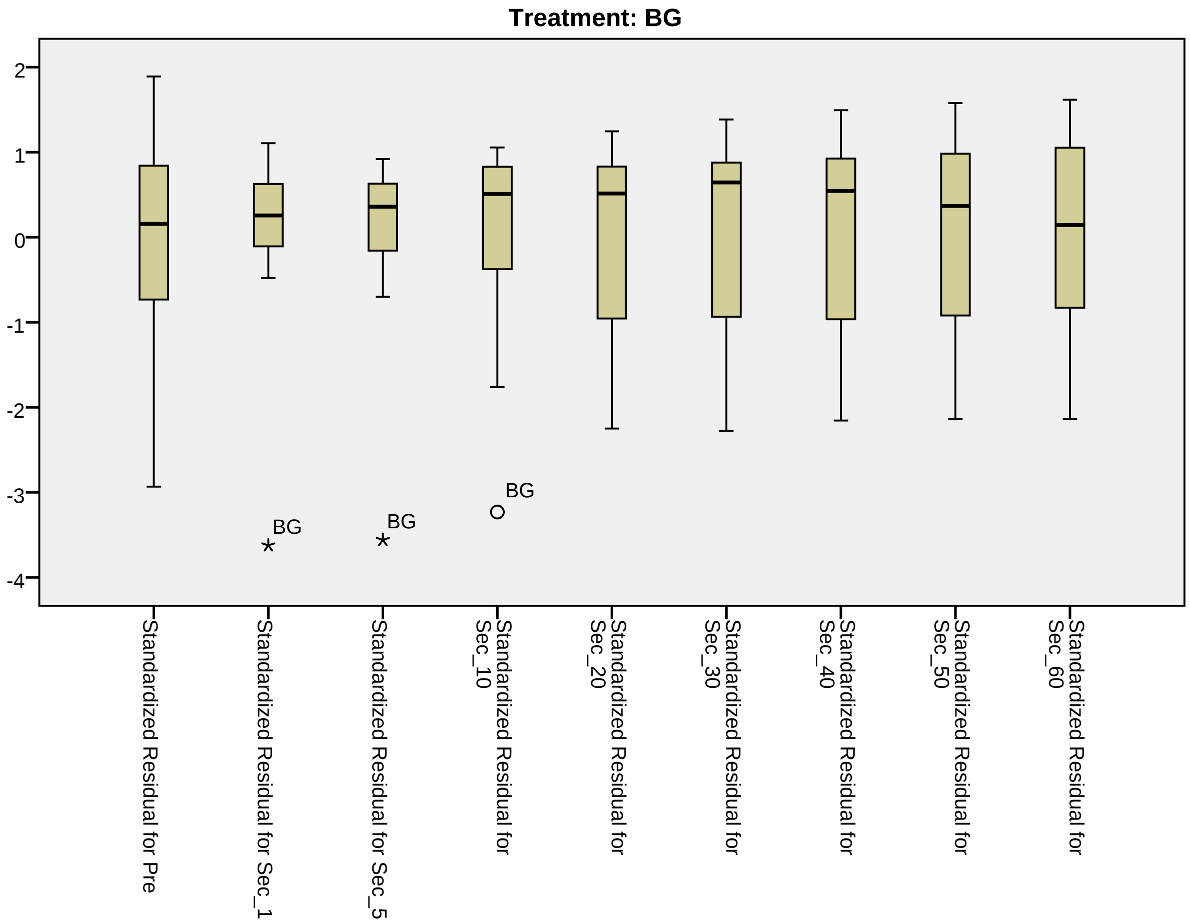

Normal Q-Q Plots of Standardized Residual for SmO$_2$ During Exercise

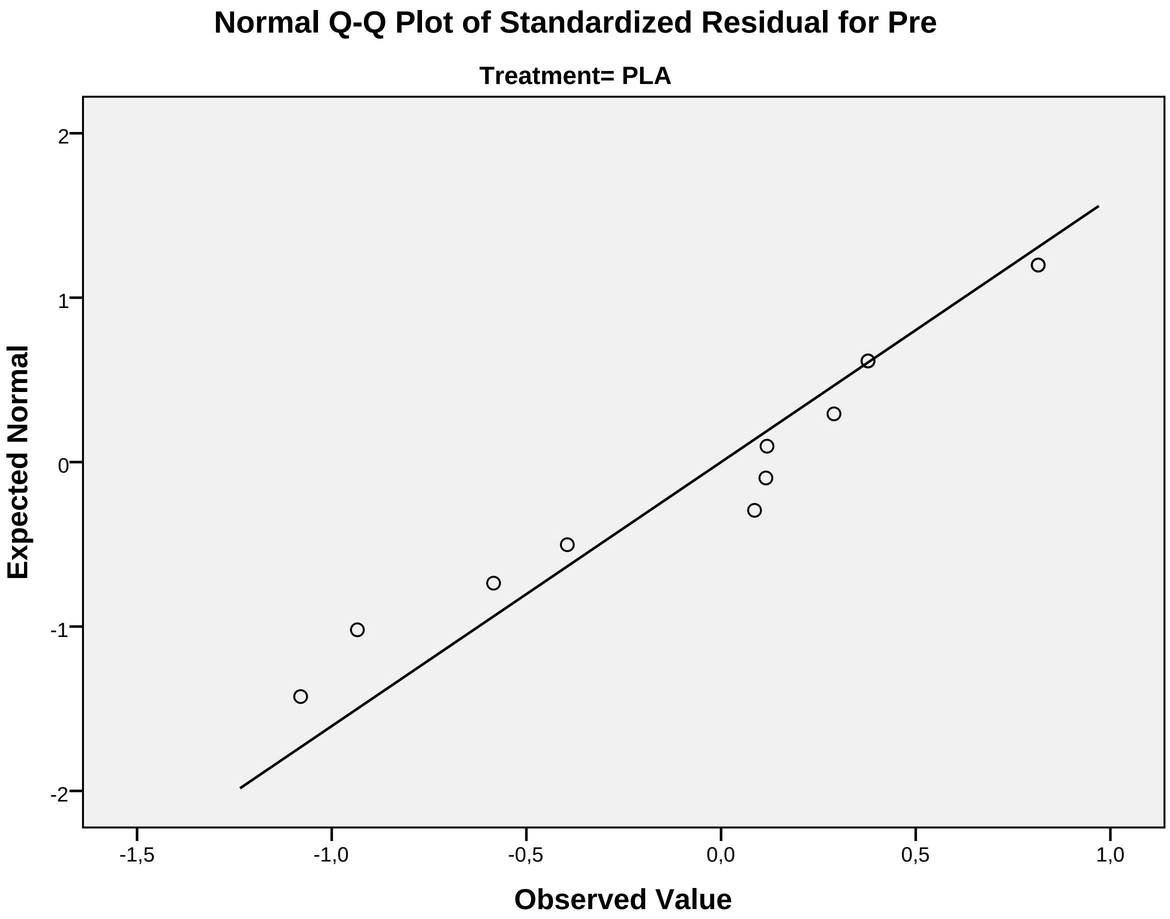
Normal Q-Q Plot of Standardized Residual for Pre
Treatment= PLA
Expected Normal
2
1
0
-1
-2
-1,5
-1,0
-0,5
0,0
0,5
1,0
Observed Value

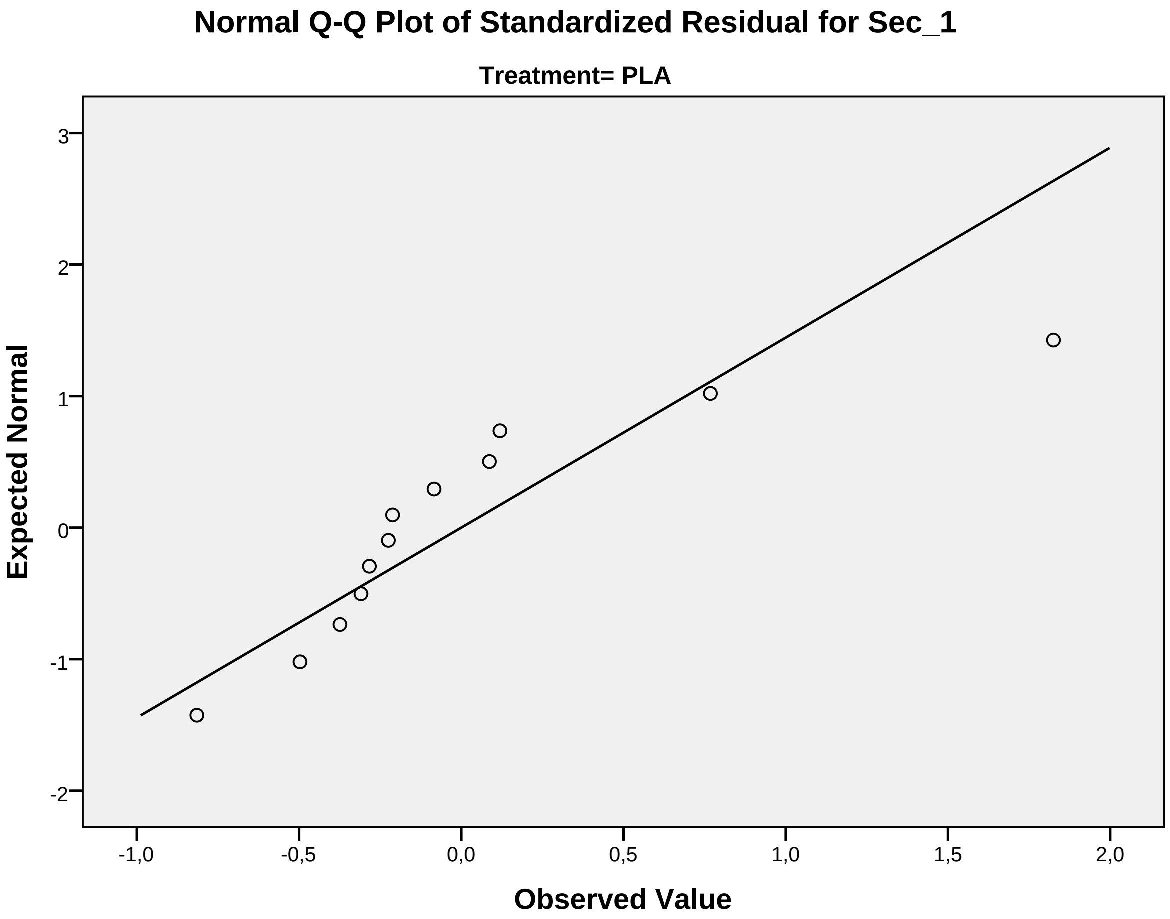
Normal Q-Q Plot of Standardized Residual for Sec_1
Treatment= PLA
Expected Normal
3
2
1
0
-1
-2
-1,0
-0,5
0,0
0,5
1,0
1,5
2,0
Observed Value

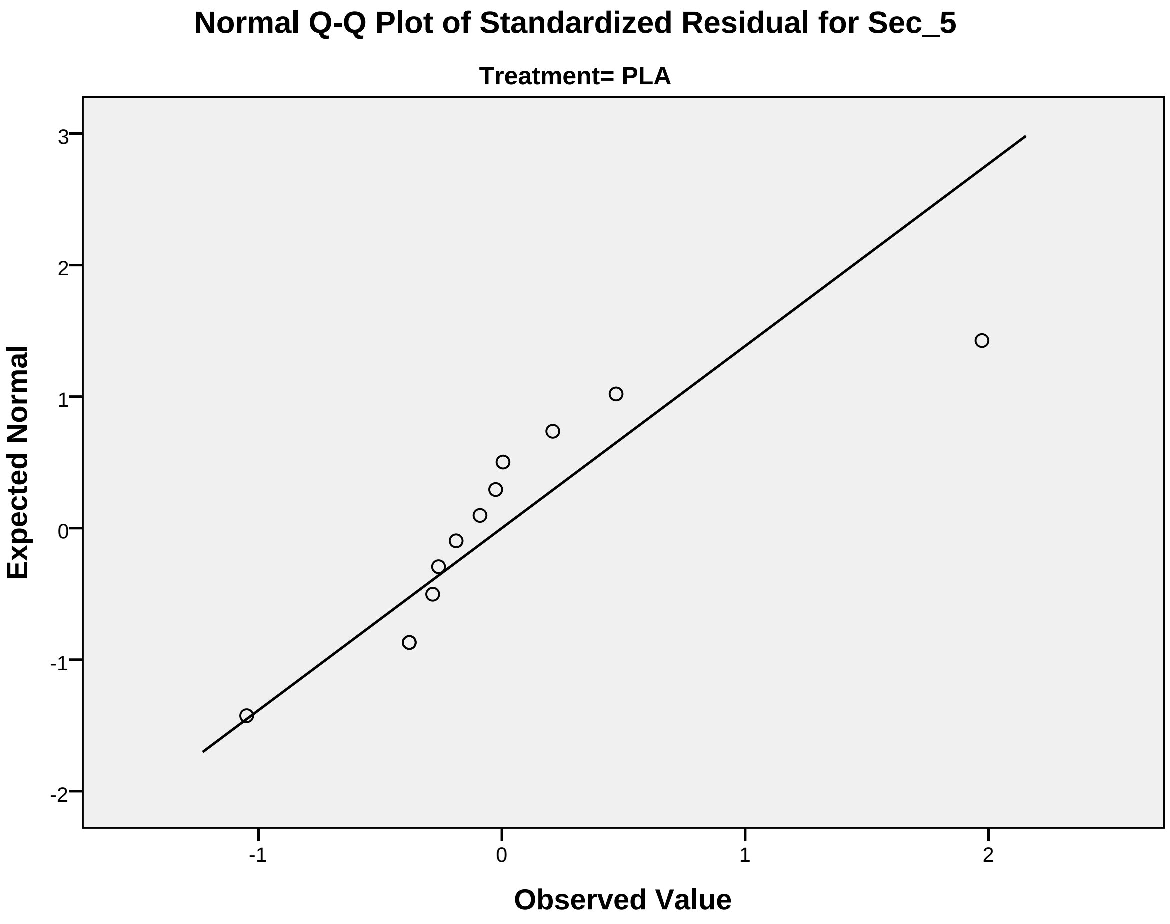
Normal Q-Q Plot of Standardized Residual for Sec_5
Treatment= PLA
Expected Normal
Observed Value
3
2
1
0
-1
-2
-1
0
1
2

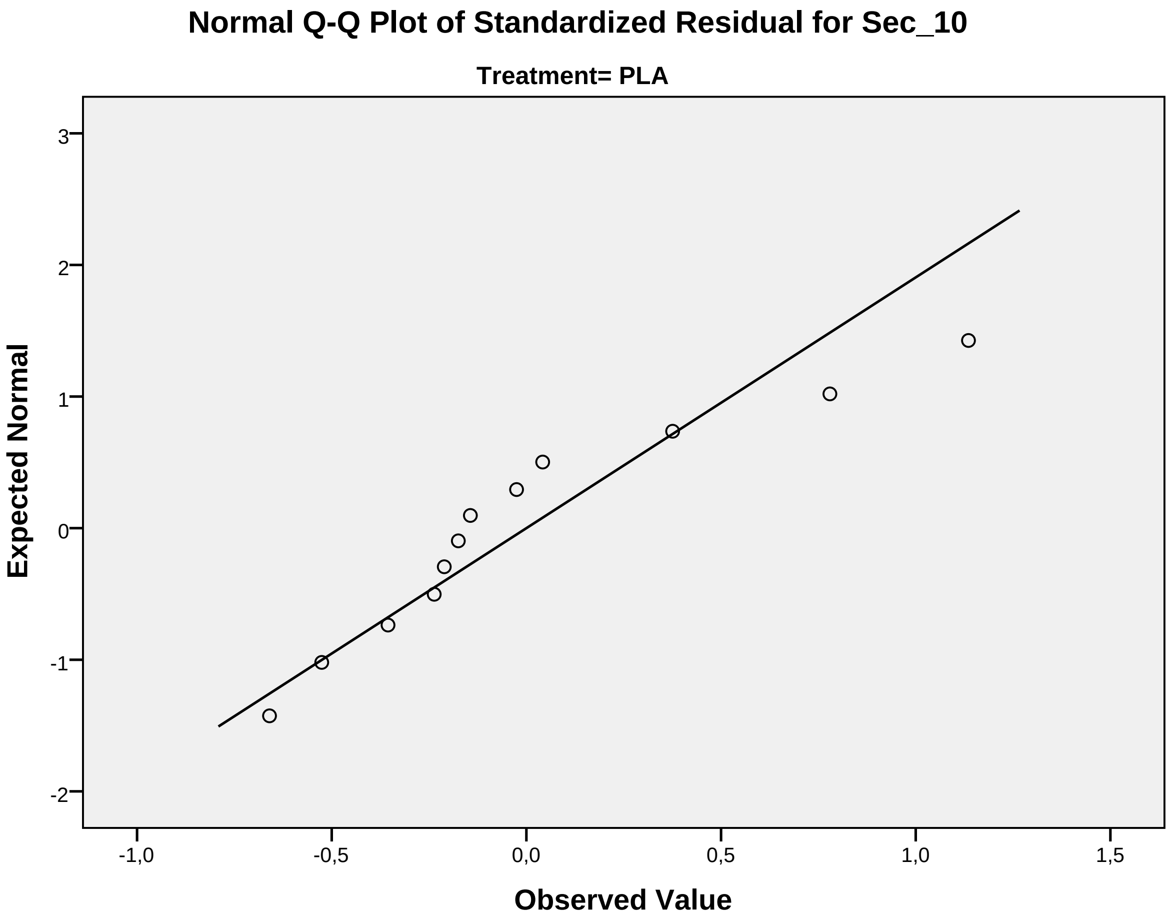

Normal Q-Q Plot of Standardized Residual for Sec_10
Treatment= PLA
Expected Normal
3
2
1
0
-1
-2
-1,0
-0,5
0,0
0,5
1,0
1,5
Observed Value

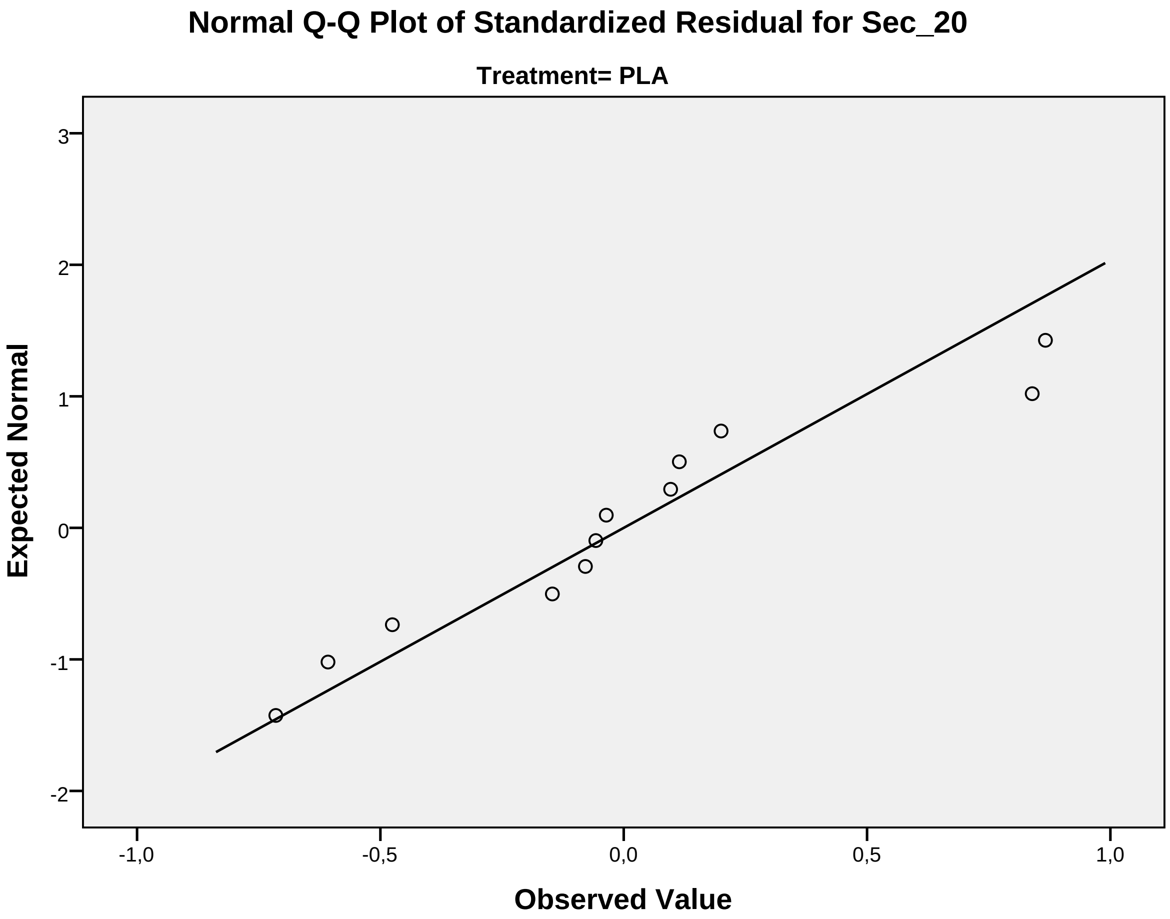
Normal Q-Q Plot of Standardized Residual for Sec_20
Treatment= PLA
Expected Normal
3
2
1
0
-1
-2
-1,0
-0,5
0,0
0,5
1,0
Observed Value

Normal Q-Q Plot of Standardized Residual for Sec_30

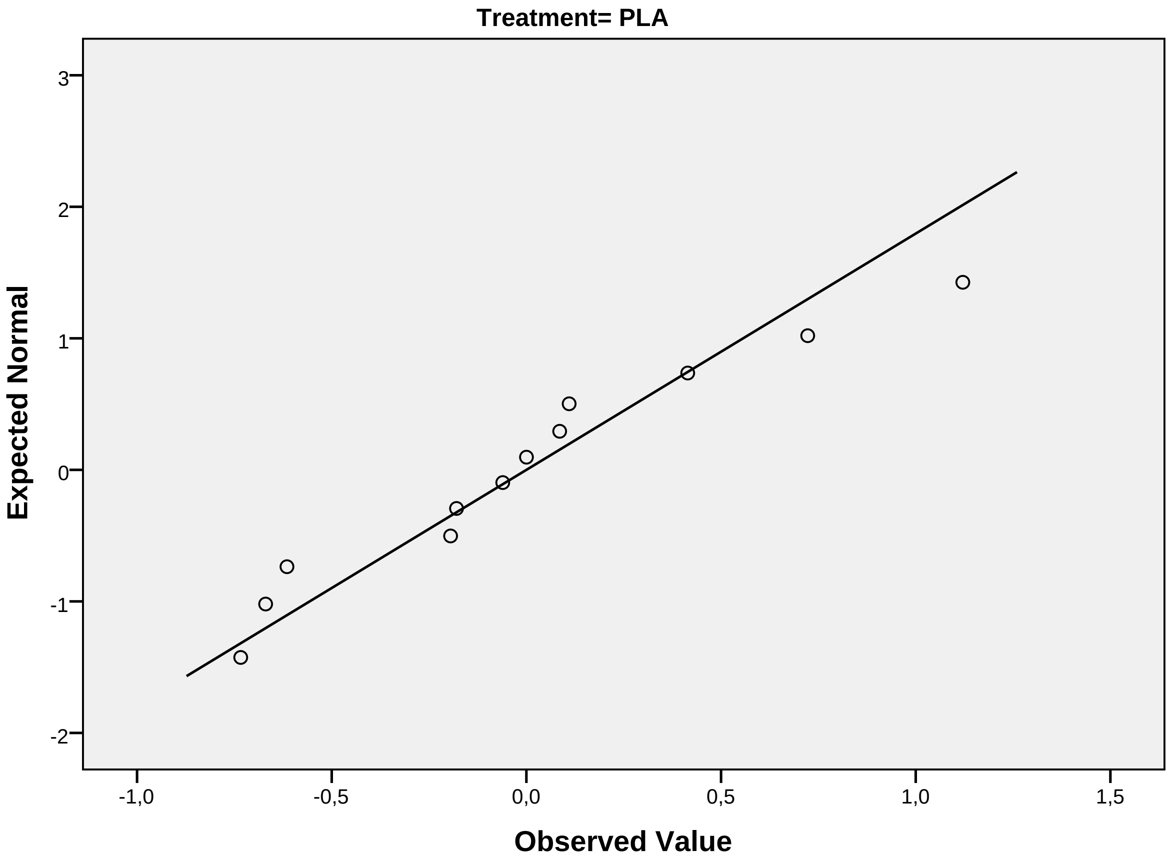

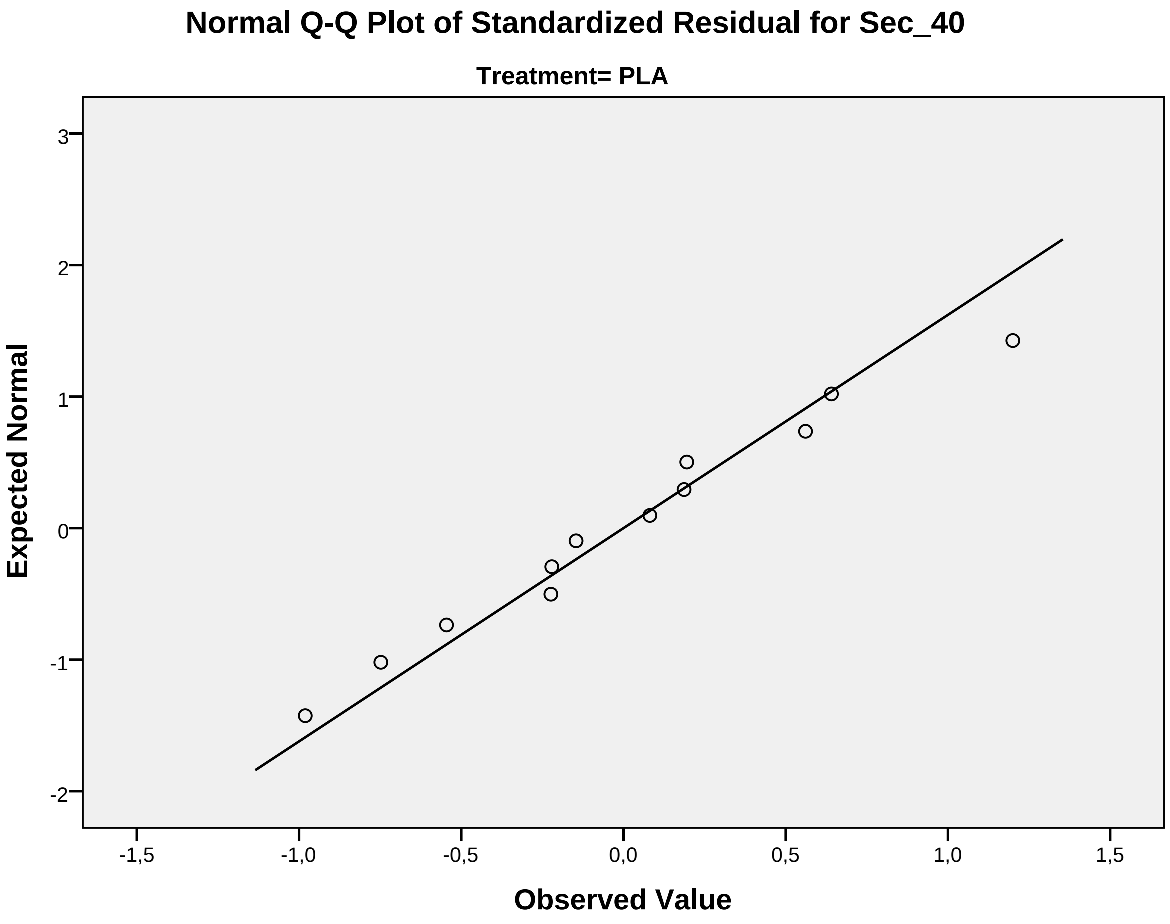
Normal Q-Q Plot of Standardized Residual for Sec_40
Treatment= PLA
Expected Normal
3
2
1
0
-1
-2
-1,5
-1,0
-0,5
0,0
0,5
1,0
1,5
Observed Value

Normal Q-Q Plot of Standardized Residual for Sec_50

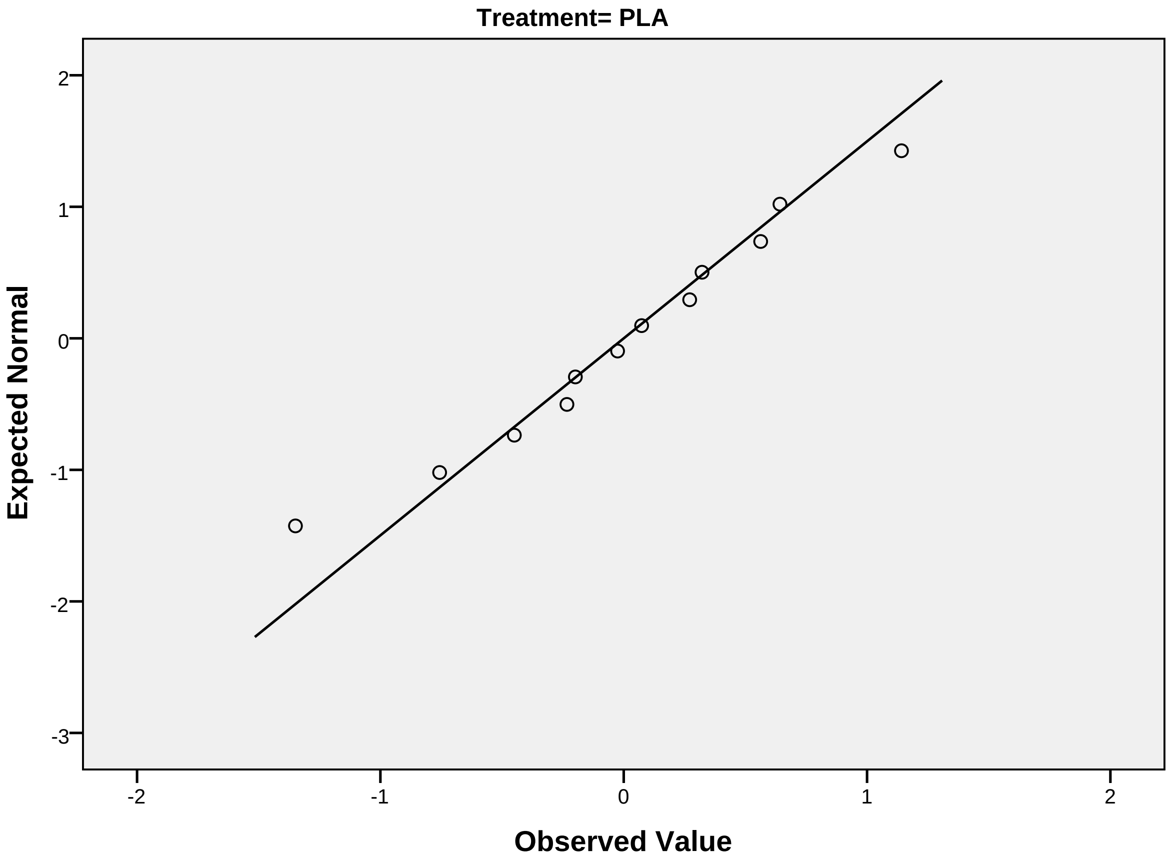

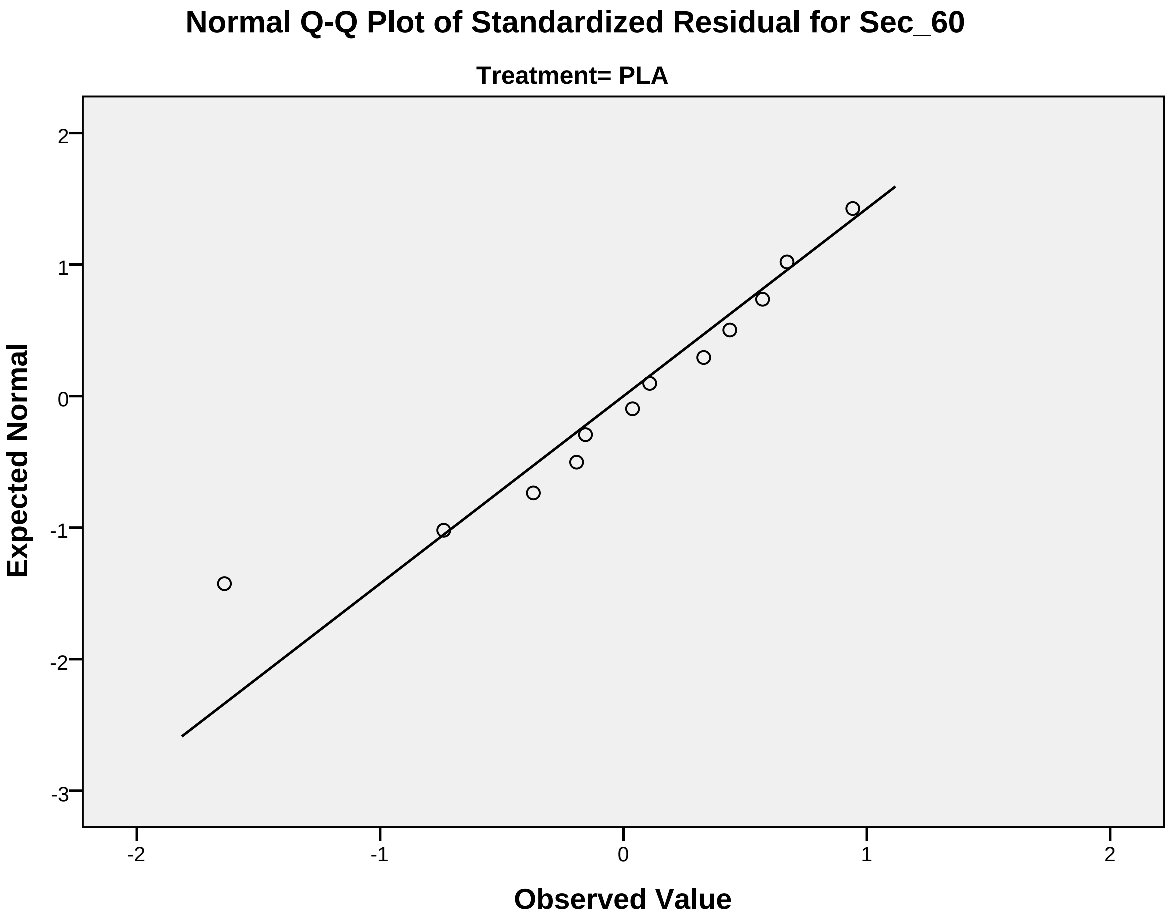

Detrended Normal Q-Q Plots of Standardized Residual for SmO_2 During Exercise

Detrended Normal Q-Q Plot of Standardized Residual for Pre

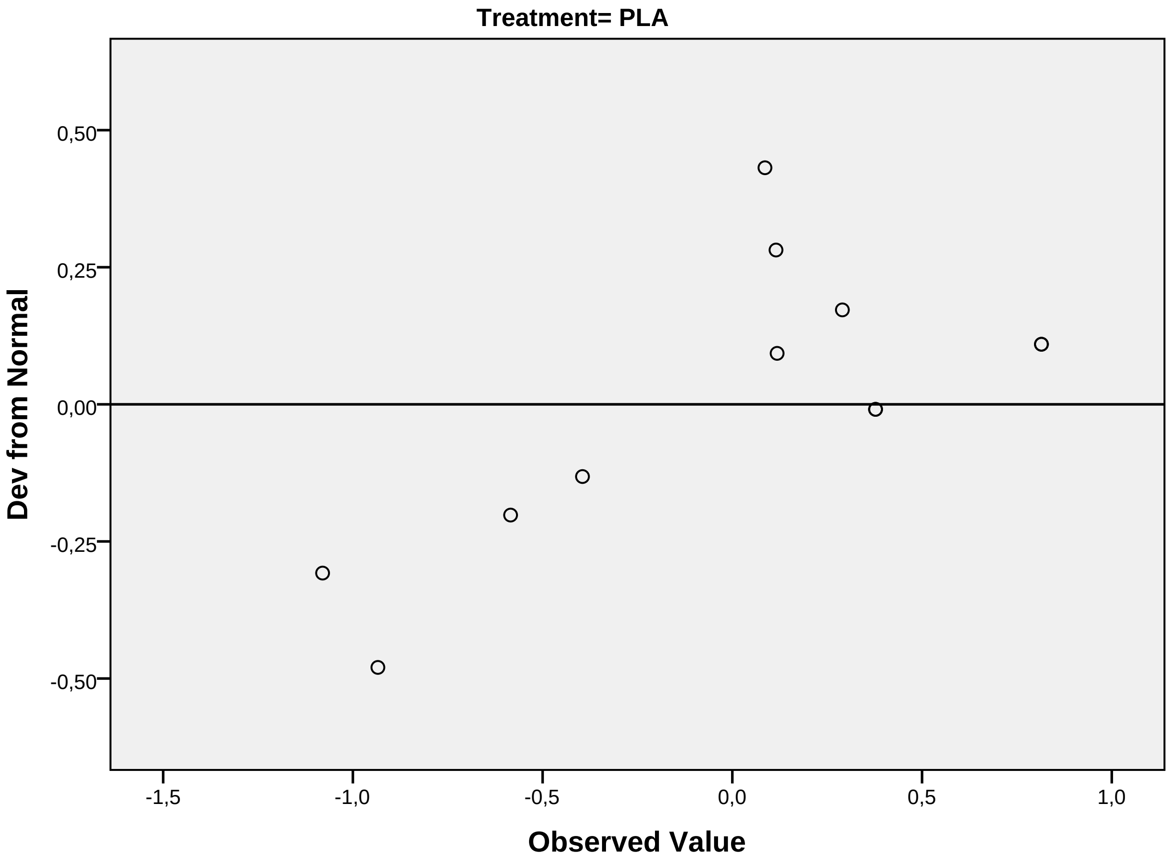

Detrended Normal Q-Q Plot of Standardized Residual for Sec_1

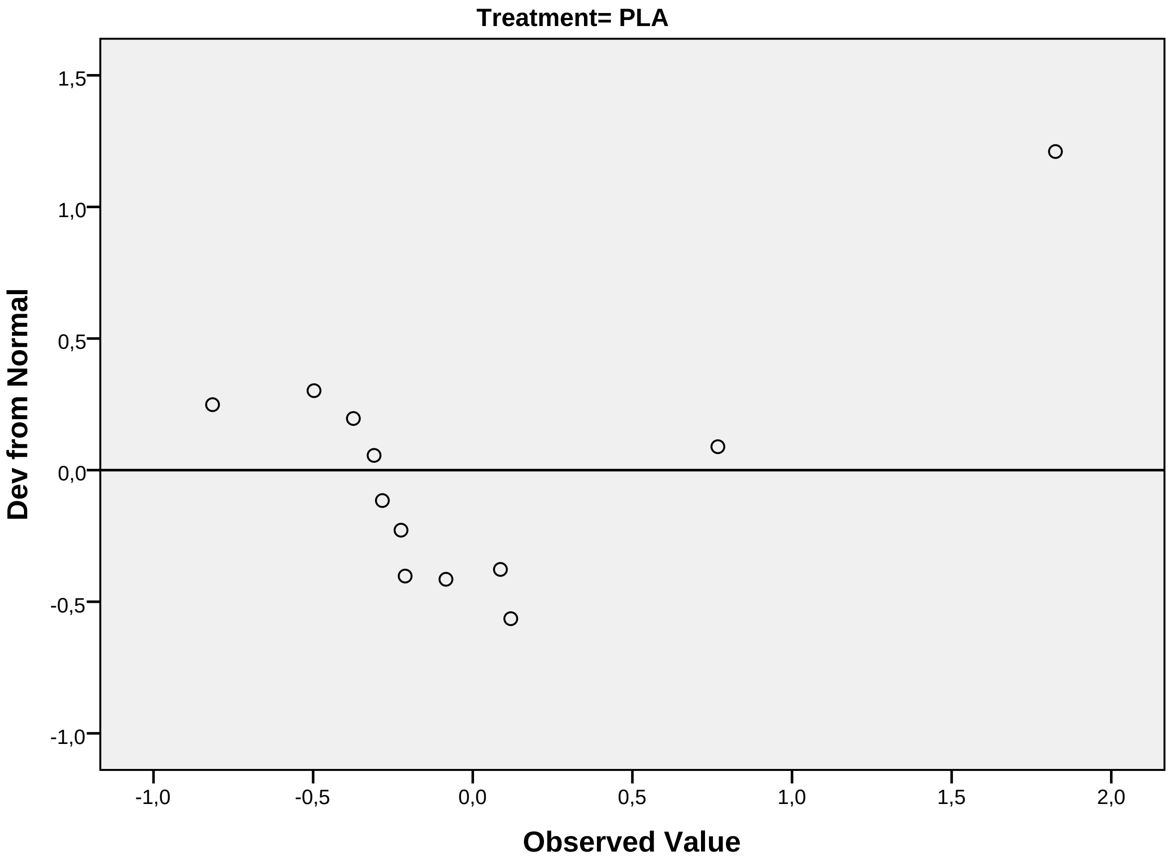

Detrended Normal Q-Q Plot of Standardized Residual for Sec_5

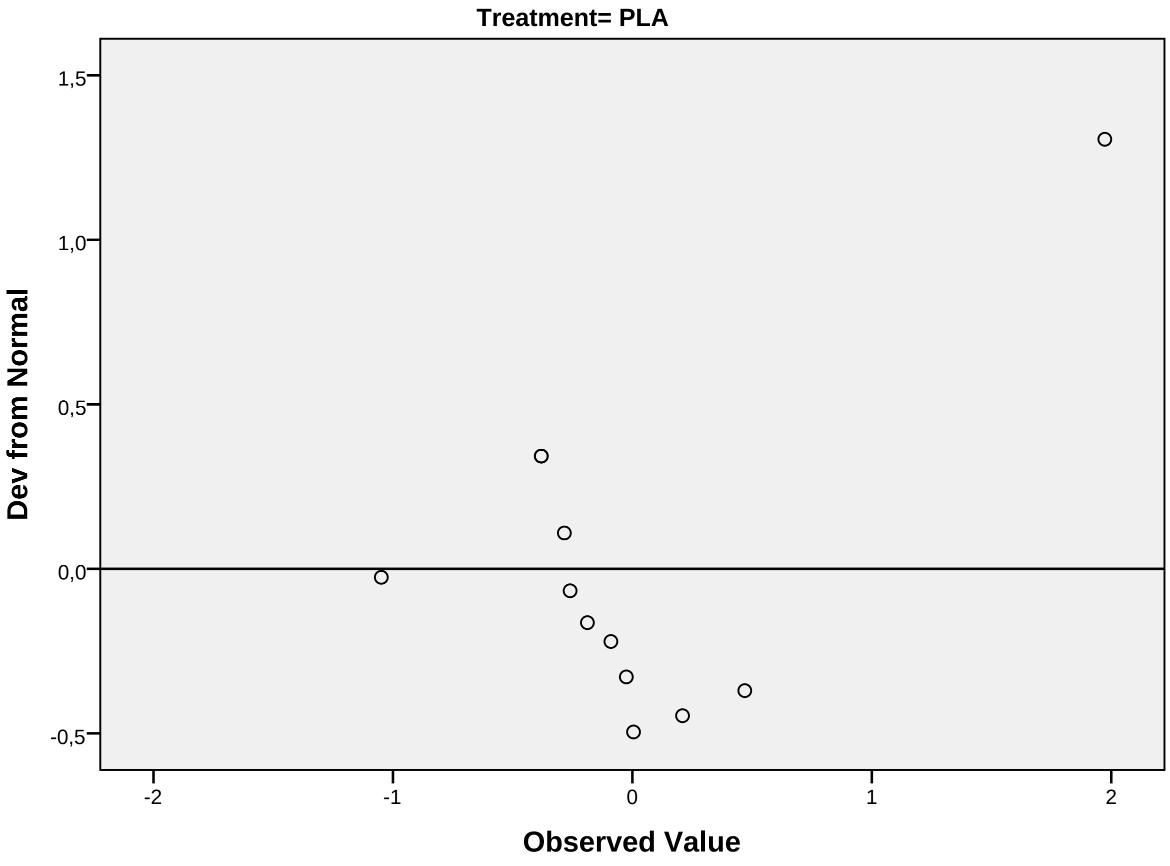

Detrended Normal Q-Q Plot of Standardized Residual for Sec_10

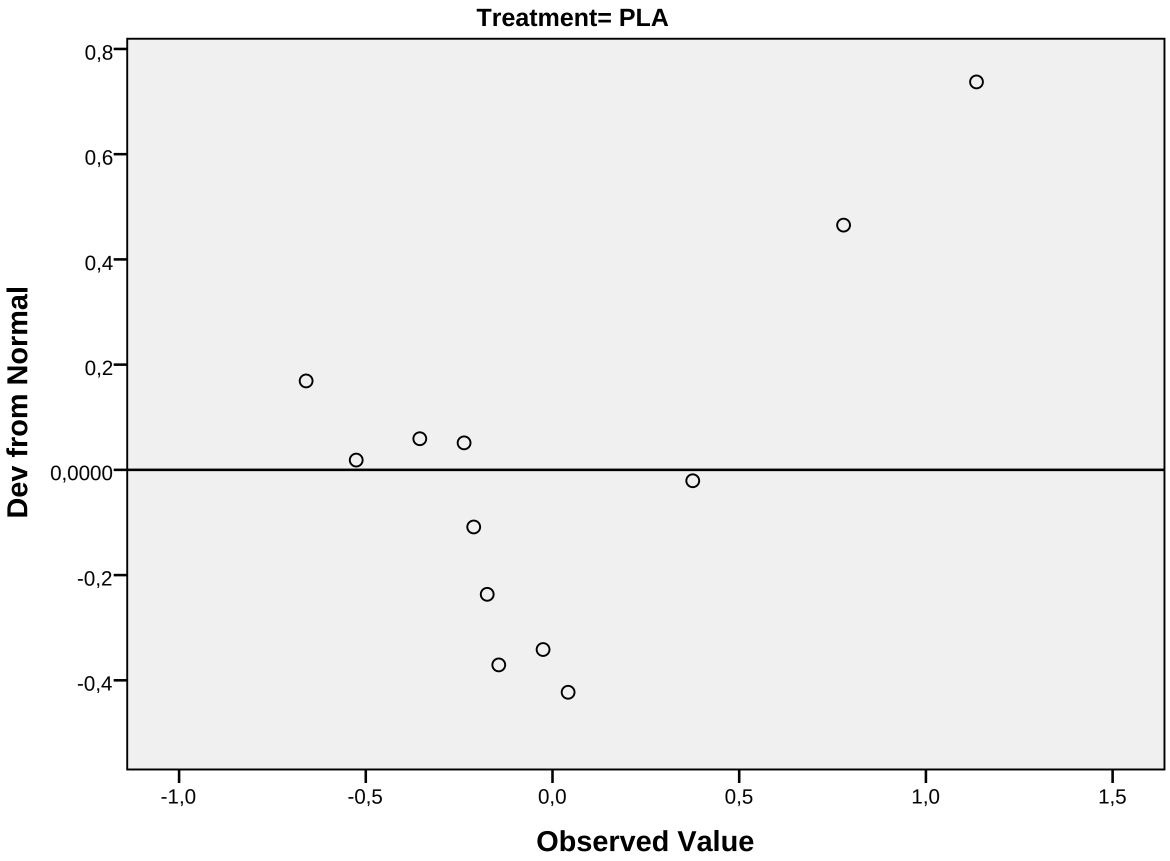

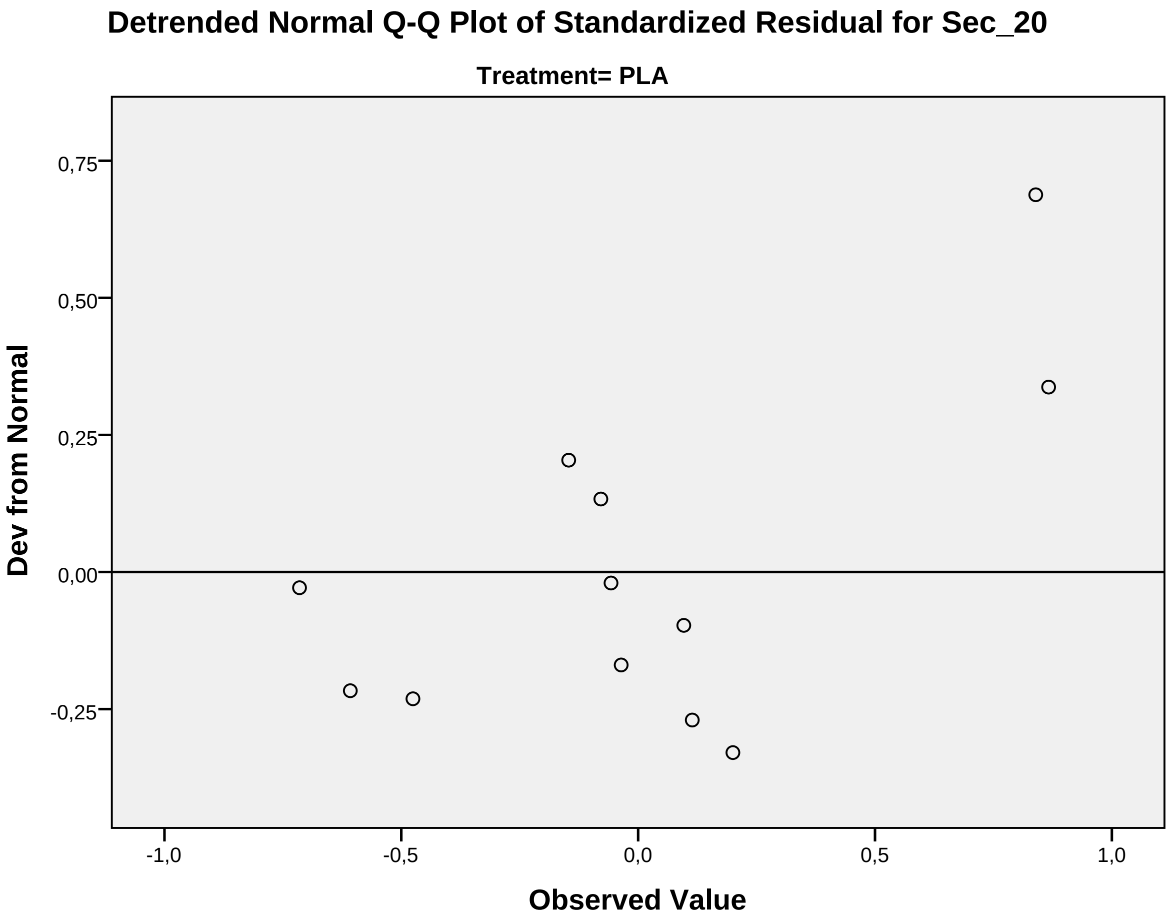

Detrended Normal Q-Q Plot of Standardized Residual for Sec_20
Treatment= PLA
Dev from Normal
0,75
0,50
0,25
0,00
-0,25
-1,0
-0,5
0,0
0,5
1,0
Observed Value

Detrended Normal Q-Q Plot of Standardized Residual for Sec_30

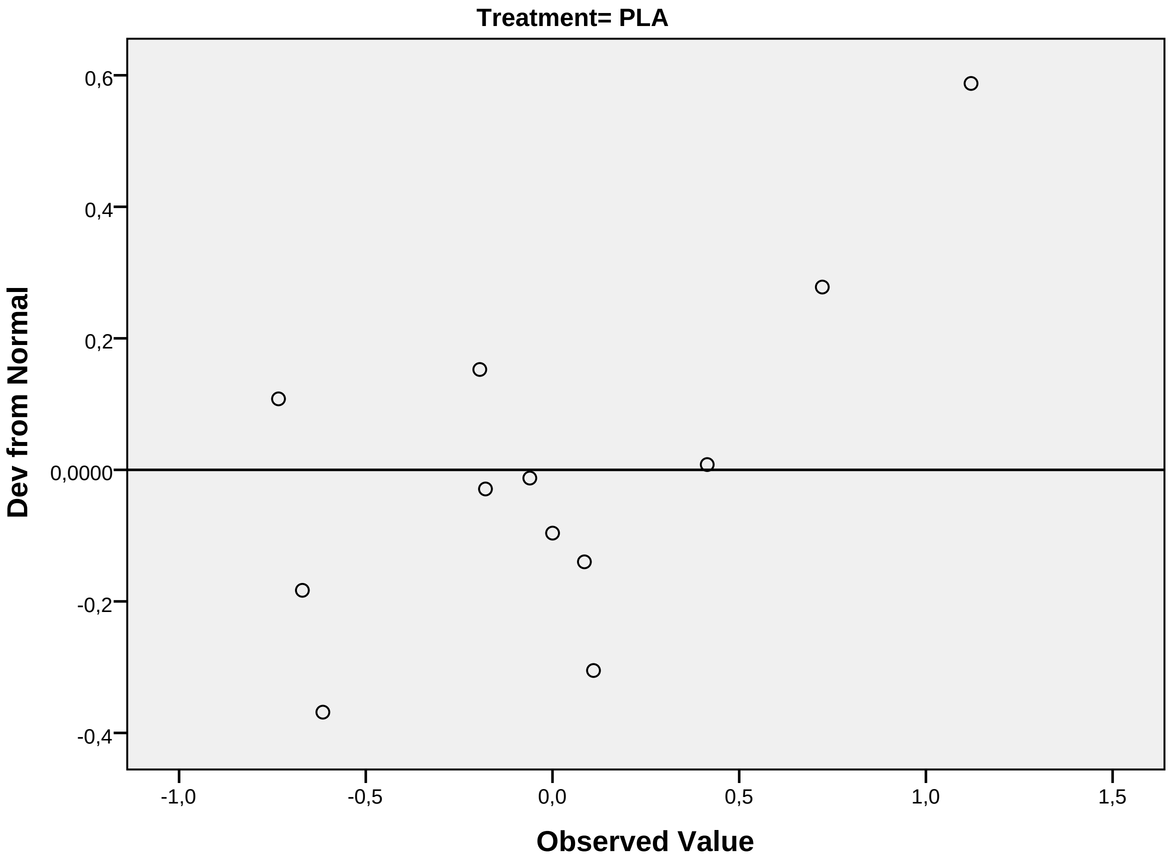

Detrended Normal Q-Q Plot of Standardized Residual for Sec_40

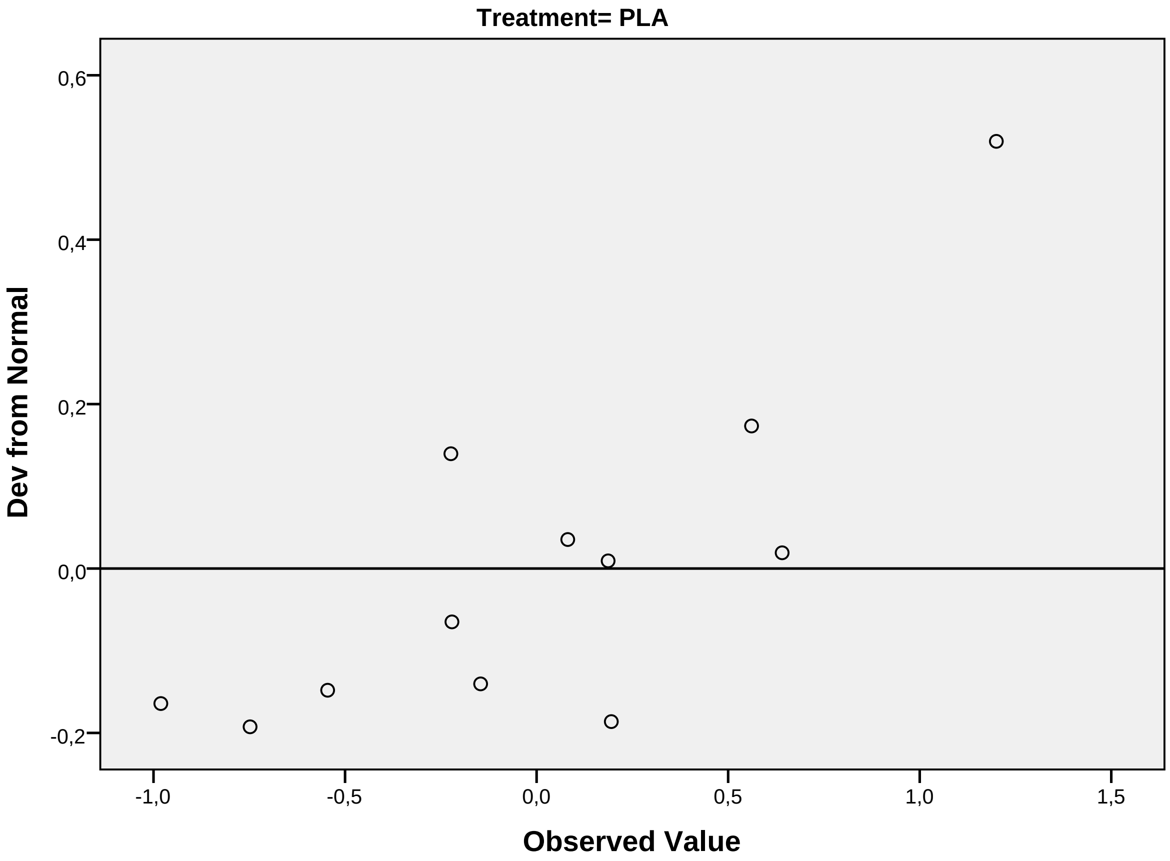

Detrended Normal Q-Q Plot of Standardized Residual for Sec_50

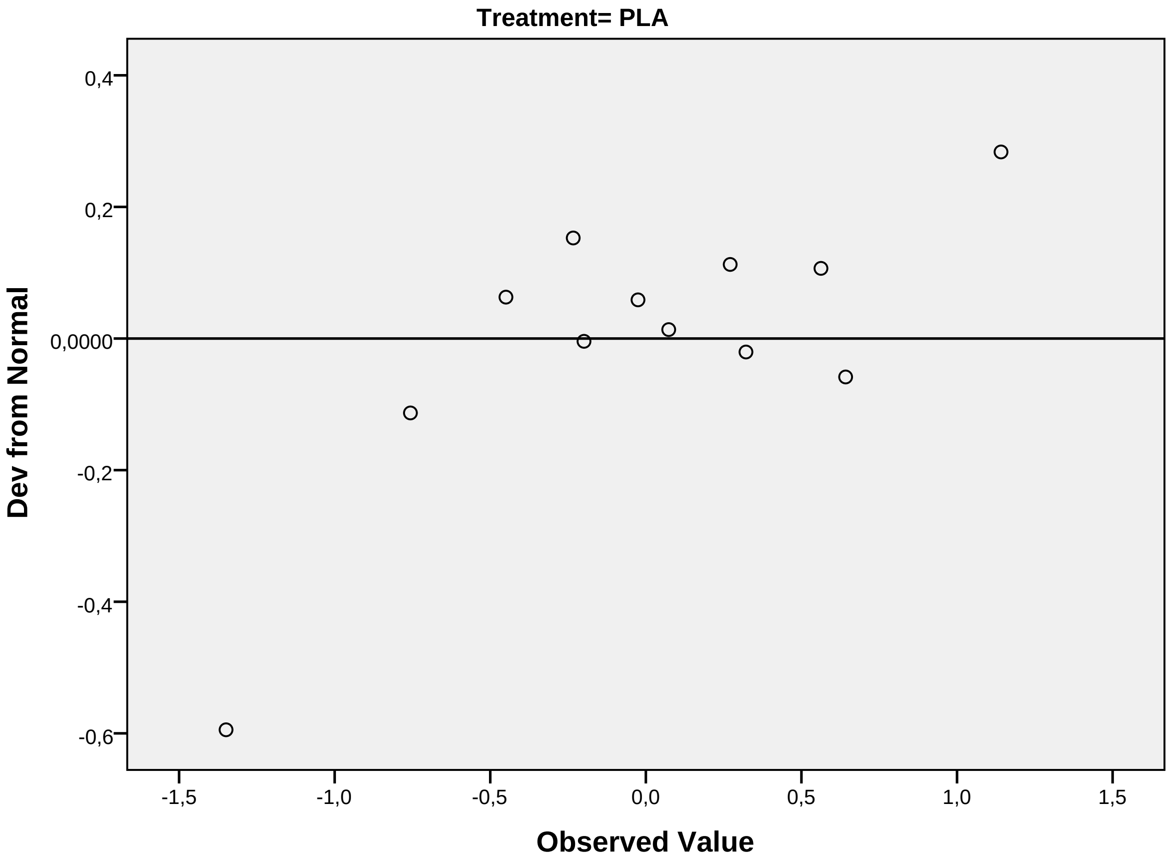

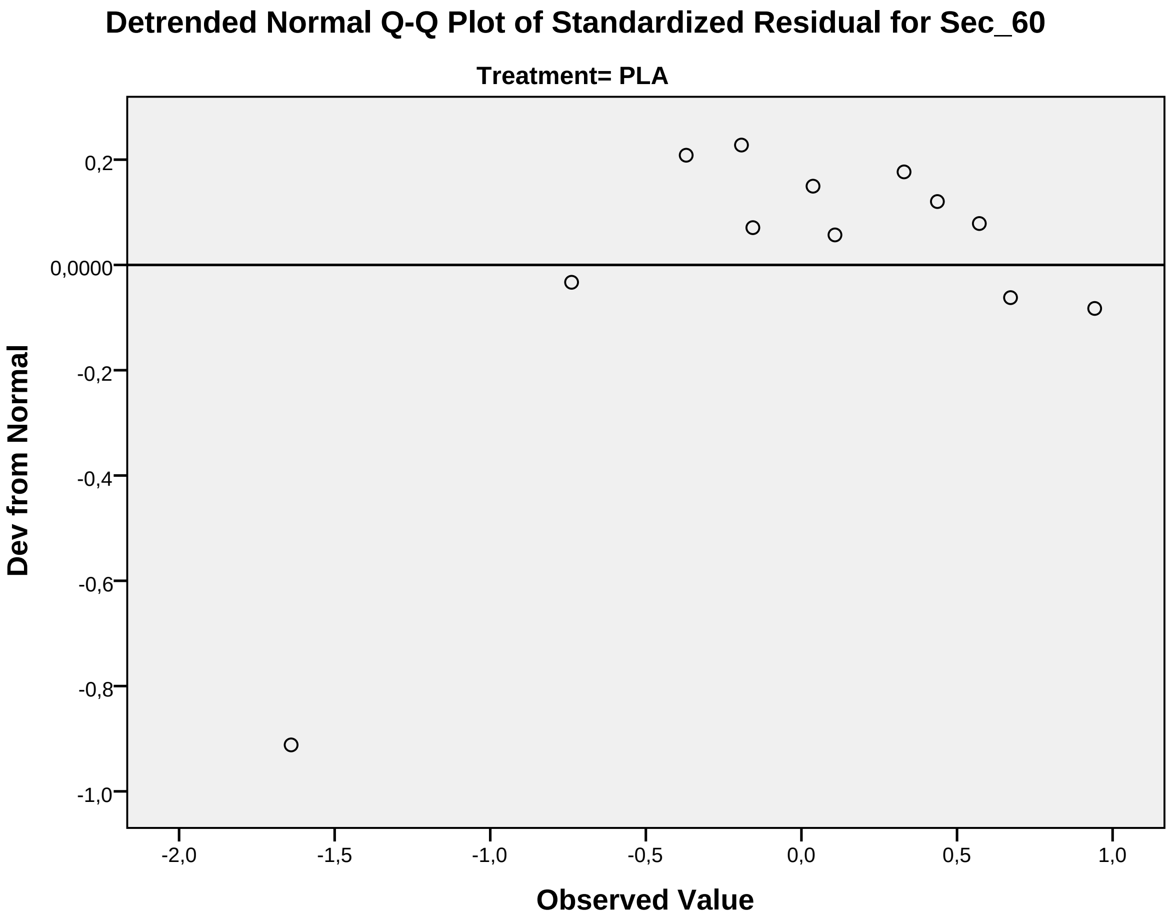
Detrended Normal Q-Q Plot of Standardized Residual for Sec_60
Treatment= PLA
Dev from Normal
0,2
0,0000
-0,2
-0,4
-0,6
-0,8
-1,0
-2,0
-1,5
-1,0
-0,5
0,0
0,5
1,0
Observed Value

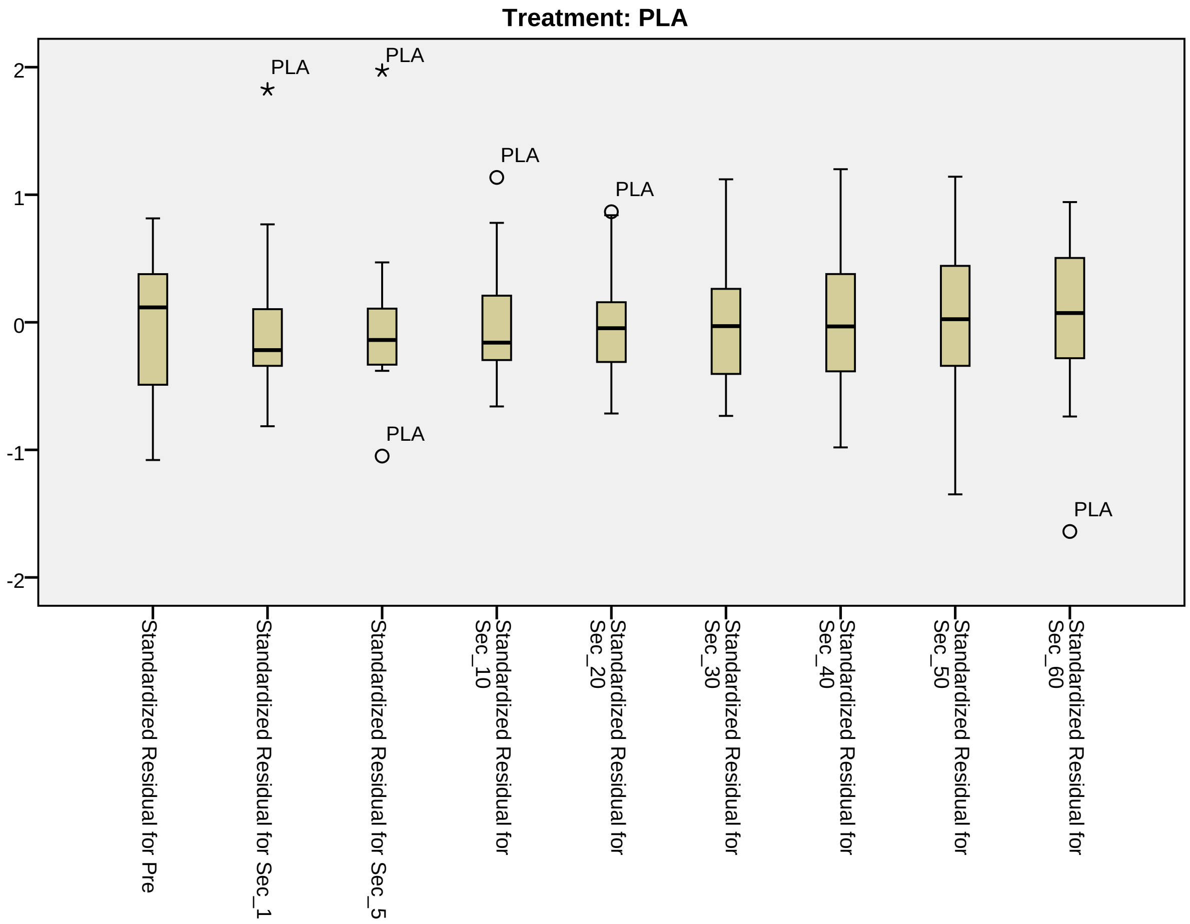
Treatment: PLA
2
1
0
-1
-2
PLA
PLA
PLA
PLA
PLA
PLA
Standardized Residual for Pre
Standardized Residual for Sec_1
Standardized Residual for Sec_5
Standardized Residual for Sec_10
Standardized Residual for Sec_20
Standardized Residual for Sec_30
Standardized Residual for Sec_40
Standardized Residual for Sec_50
Standardized Residual for Sec_60

Normal Q-Q Plots of Standardized Residual for SmO_2 During Recovery

Normal Q-Q Plot of Standardized Residual for Pre

Treatment= BG

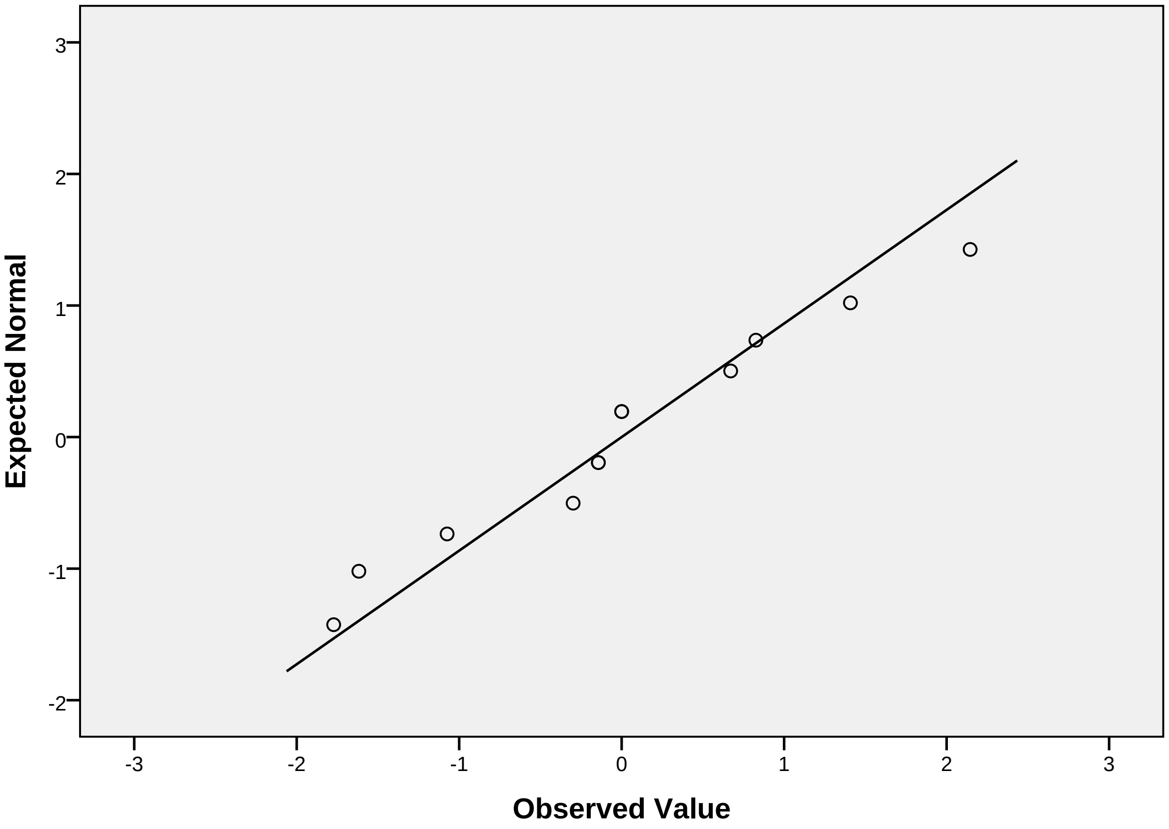

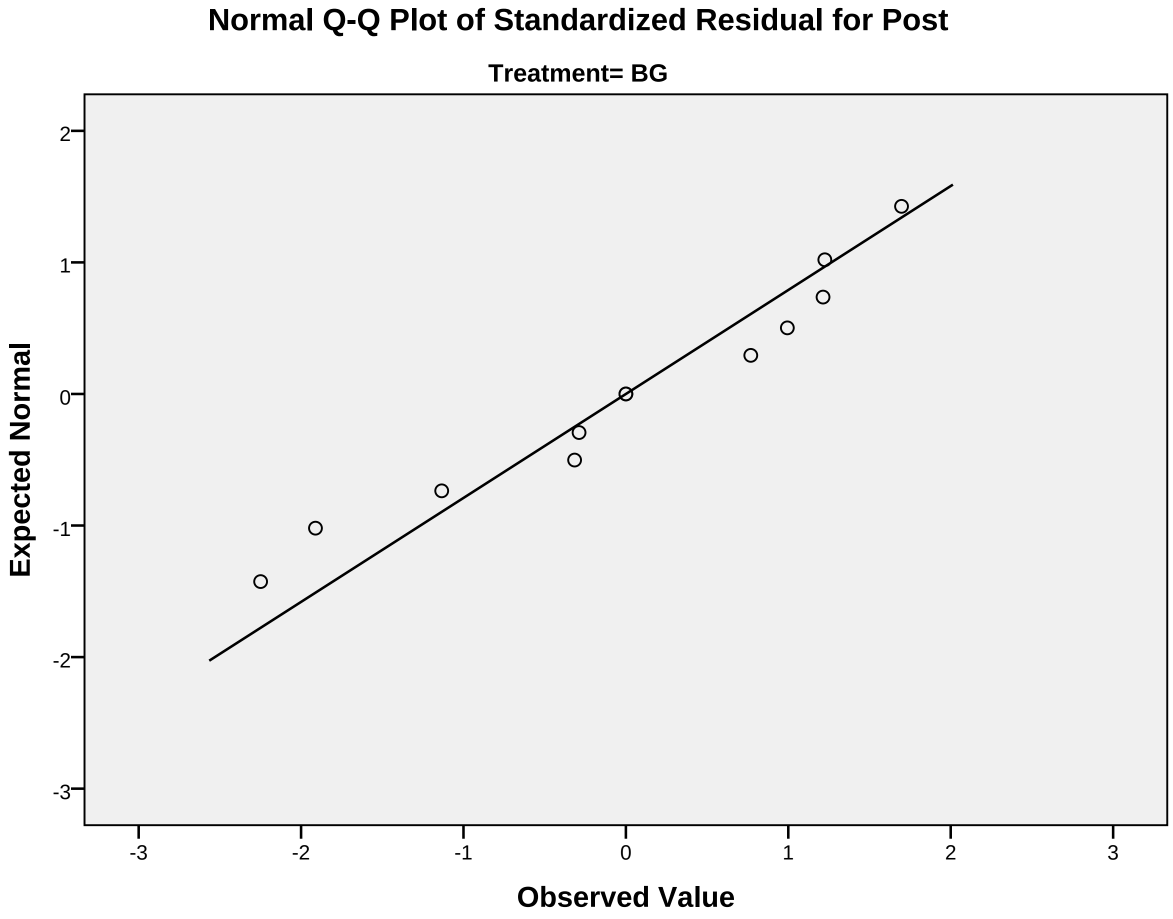
Normal Q-Q Plot of Standardized Residual for Post
Treatment= BG
Expected Normal
2
1
0
-1
-2
-3
-3
-2
-1
0
1
2
3
Observed Value

Normal Q-Q Plot of Standardized Residual for Sec_1

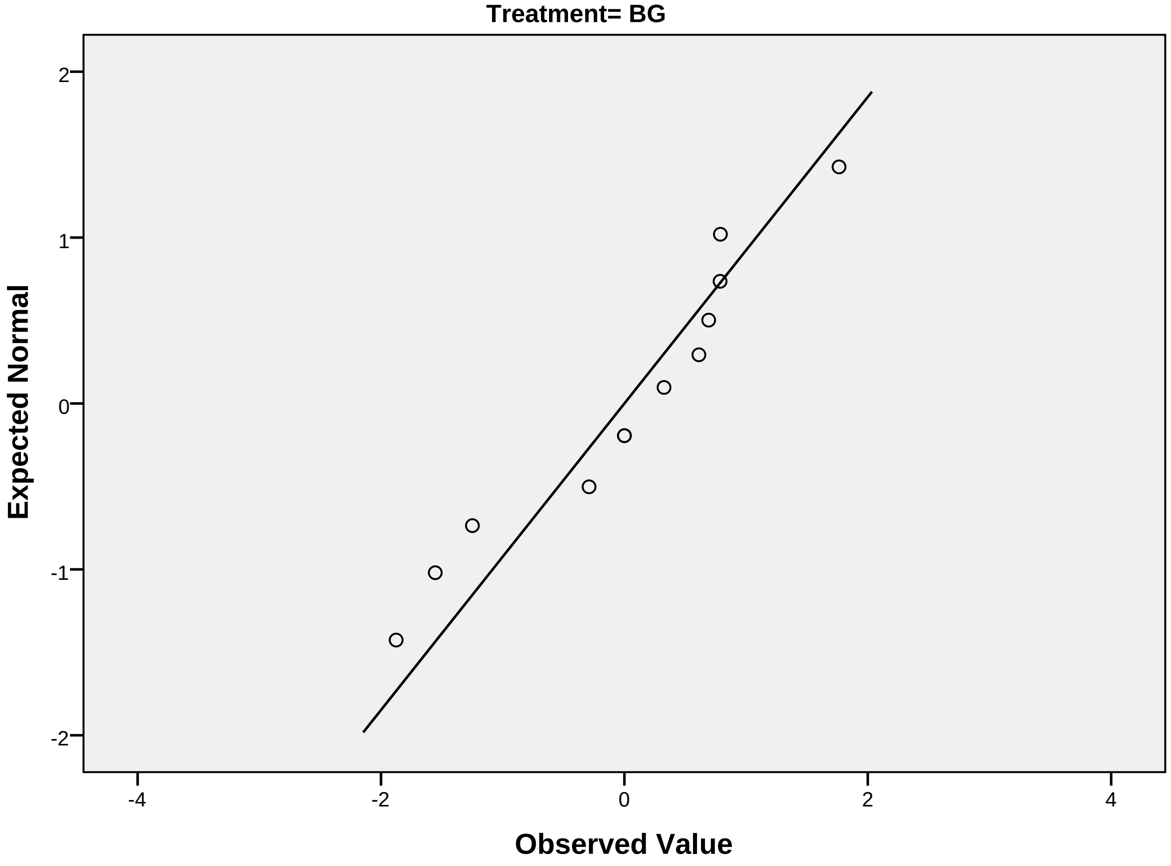

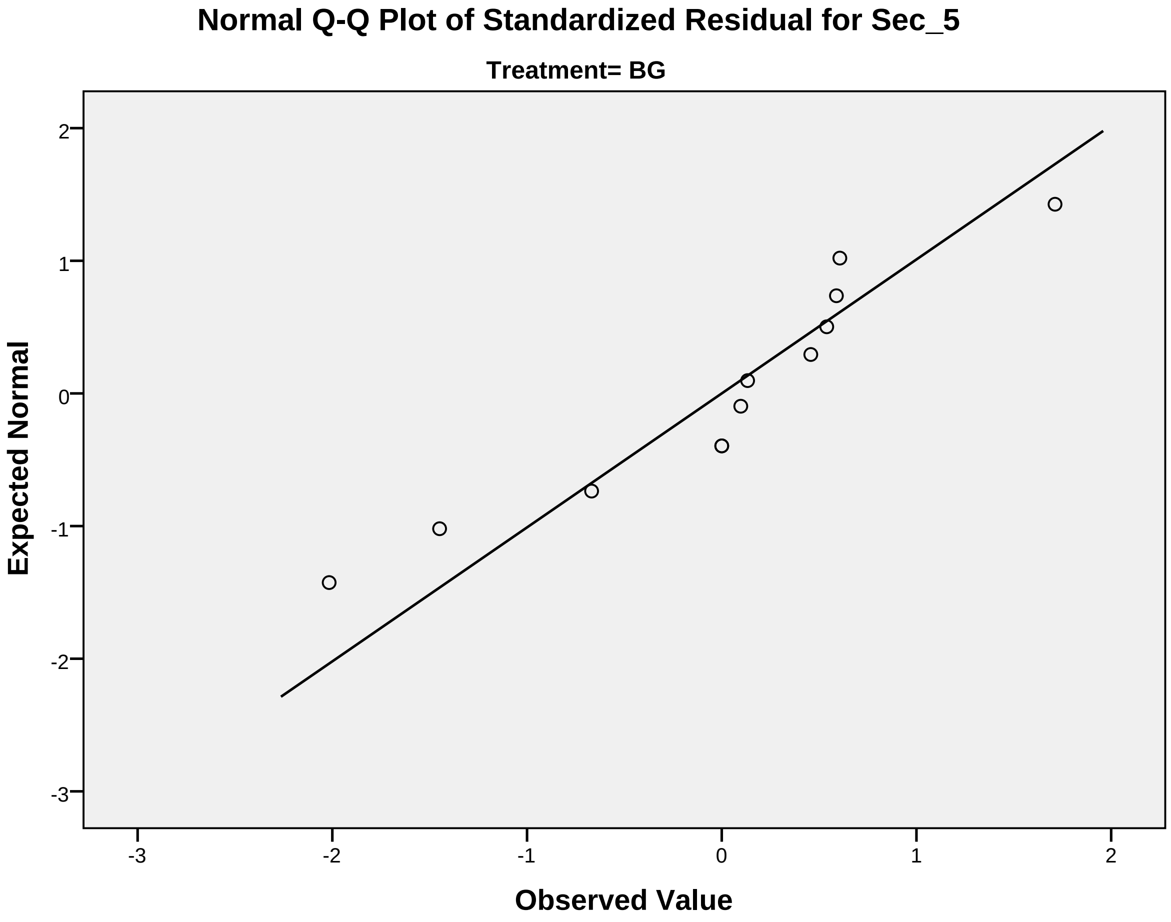
Normal Q-Q Plot of Standardized Residual for Sec_5
Treatment= BG
Expected Normal
Observed Value
2
1
0
-1
-2
-3
-3
-2
-1
0
1
2

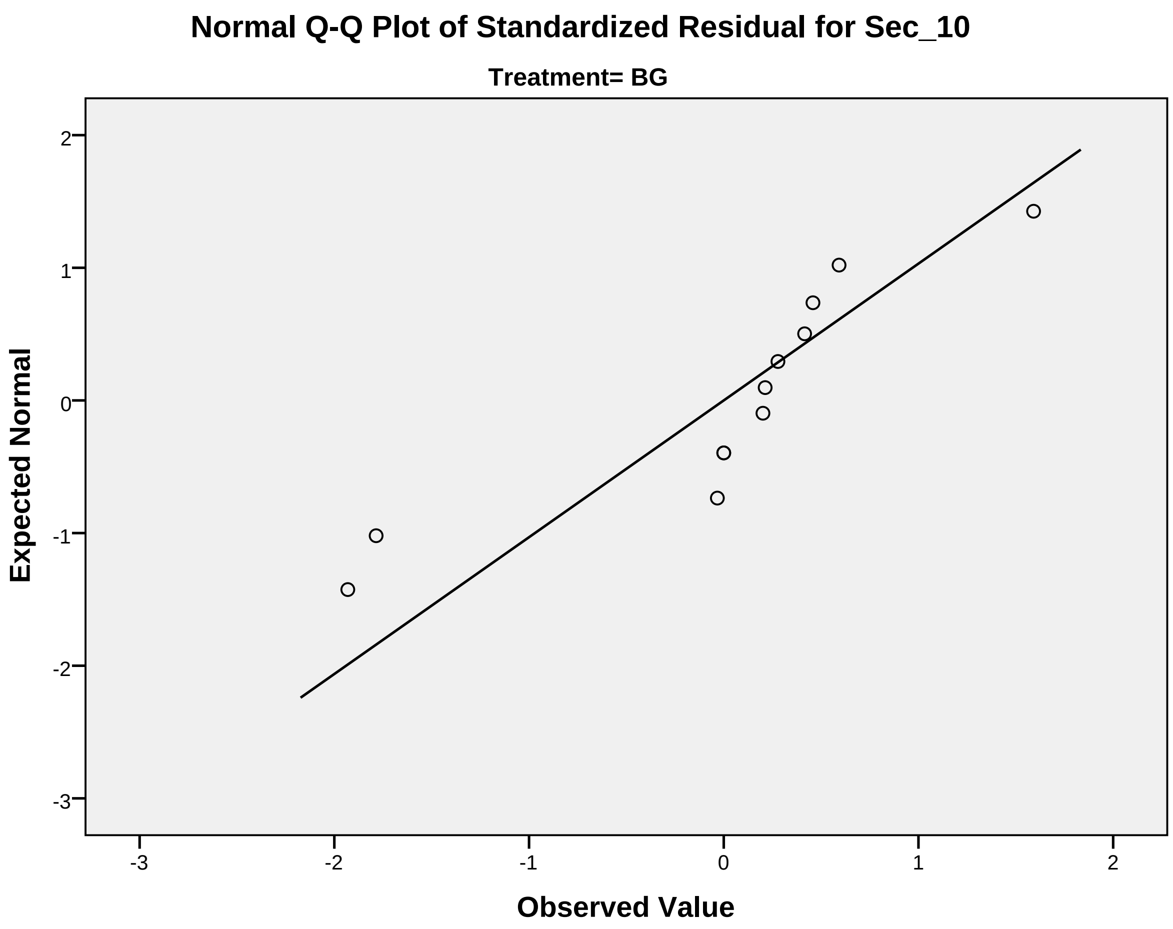
Normal Q-Q Plot of Standardized Residual for Sec_10
Treatment= BG
Expected Normal
2
1
0
-1
-2
-3
-3
-2
-1
0
1
2
Observed Value

Normal Q-Q Plot of Standardized Residual for Sec_20

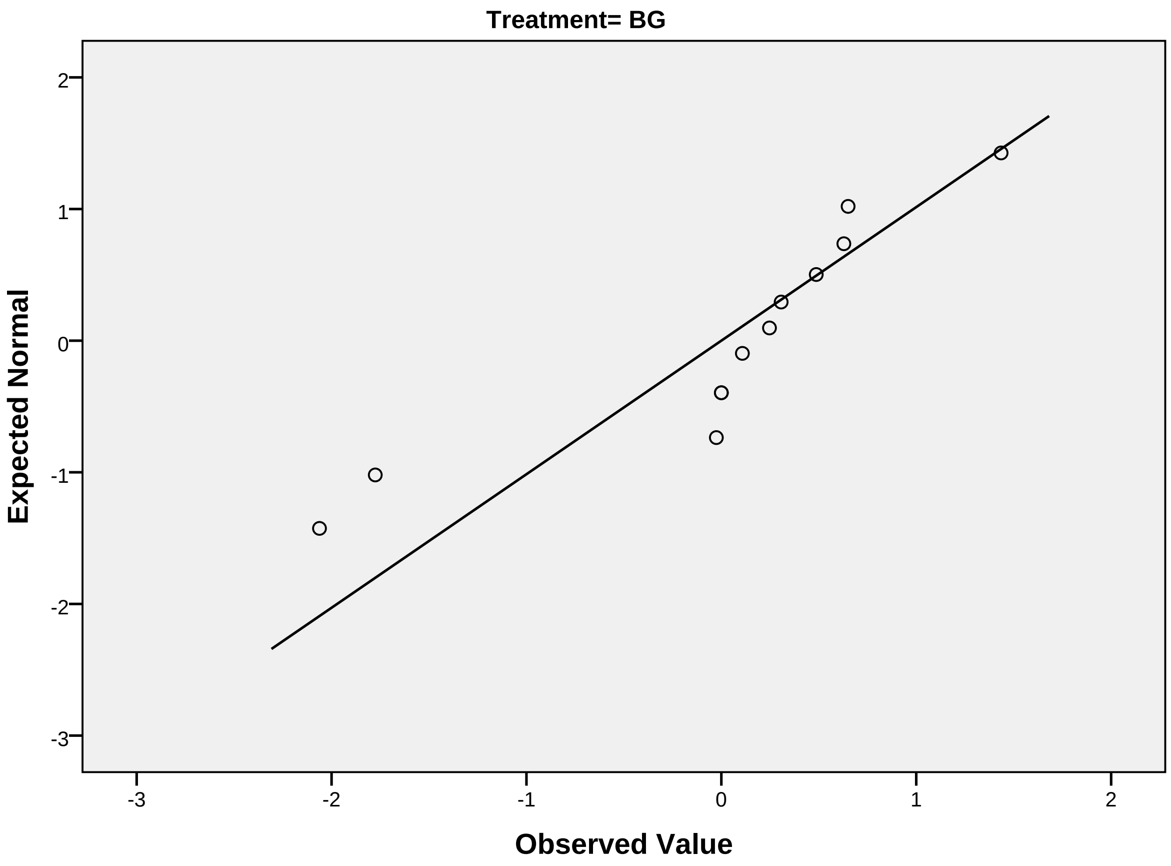

Normal Q-Q Plot of Standardized Residual for Sec_30

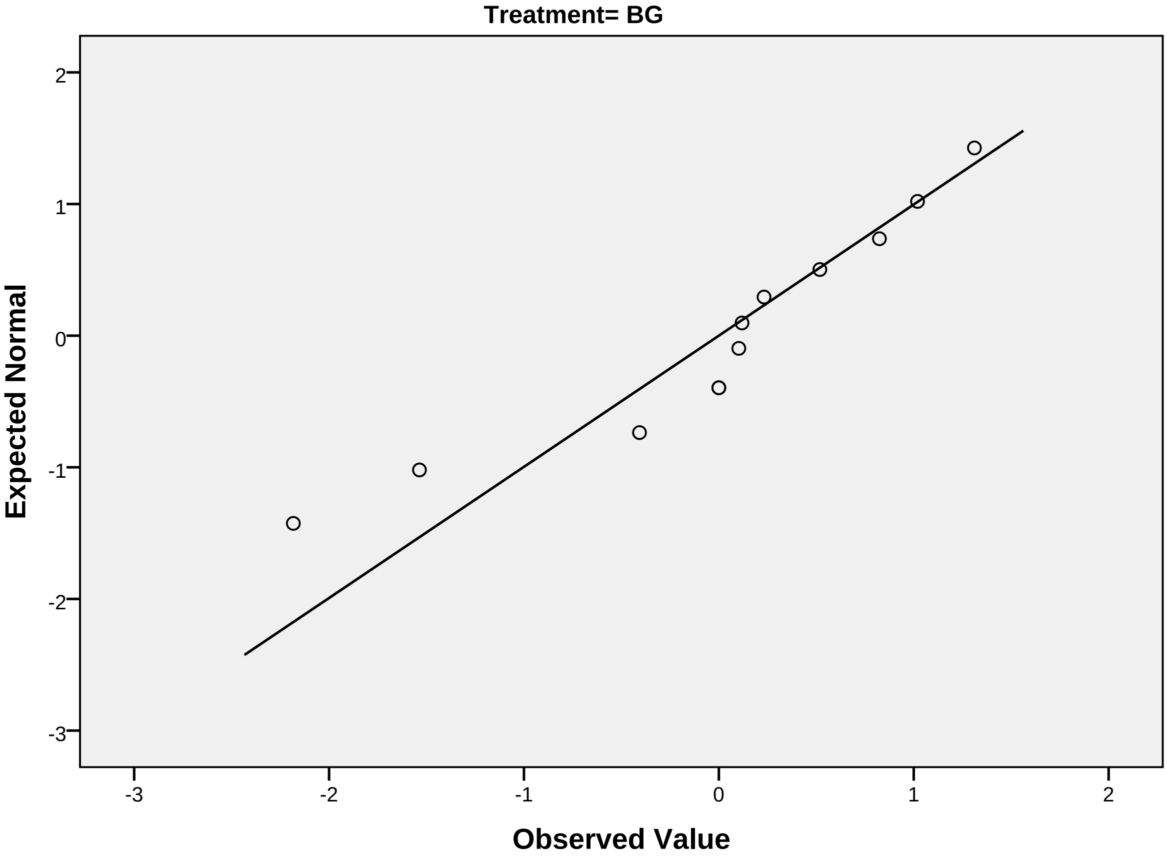

Normal Q-Q Plot of Standardized Residual for Sec_40

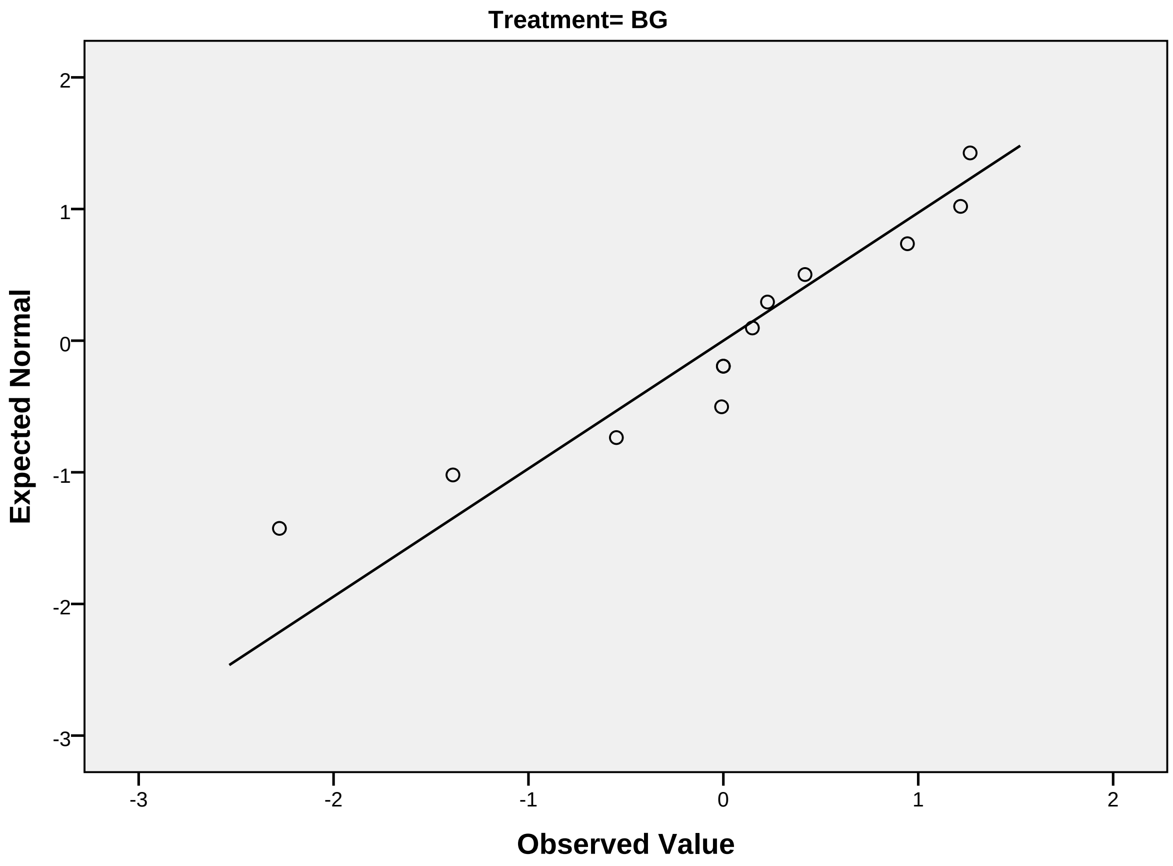

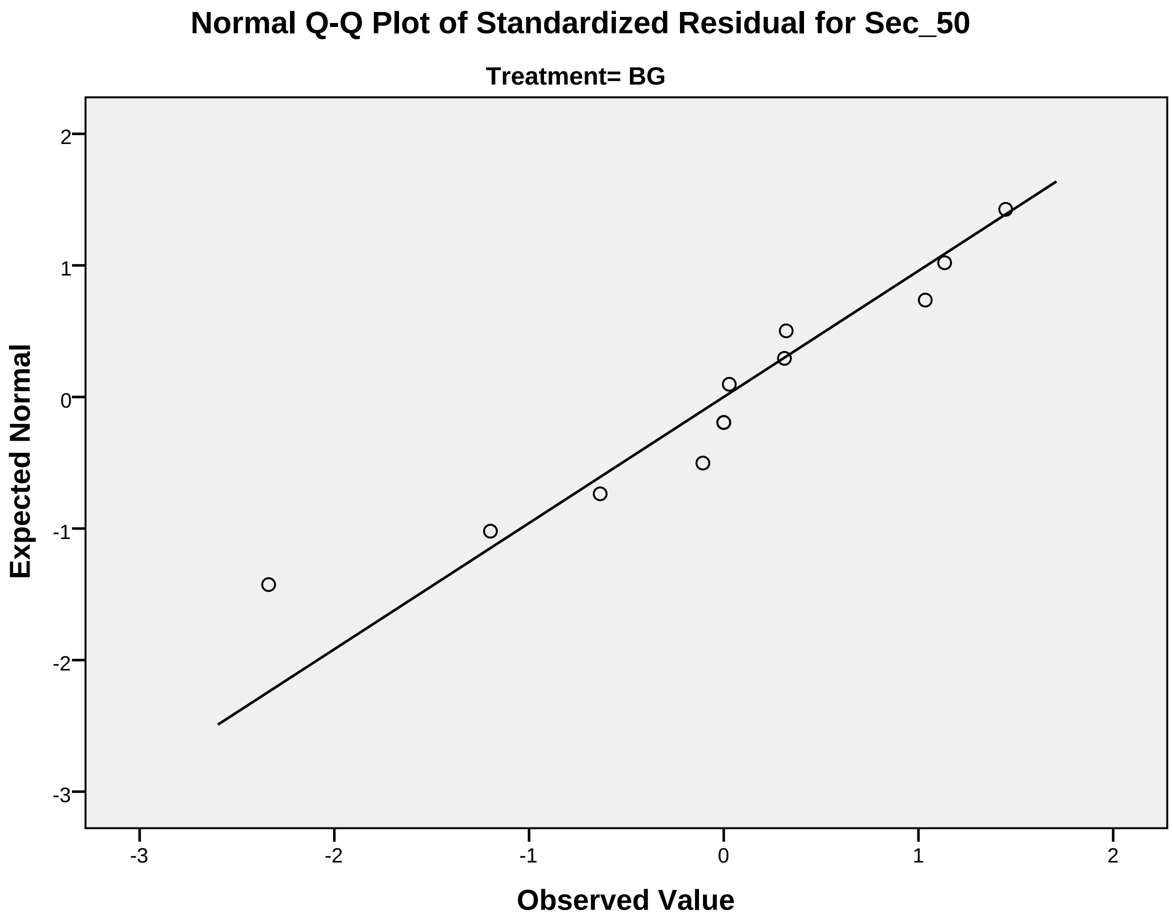
Normal Q-Q Plot of Standardized Residual for Sec_50
Treatment= BG
Expected Normal
Observed Value
2
1
0
-1
-2
-3
-3
-2
-1
0
1
2

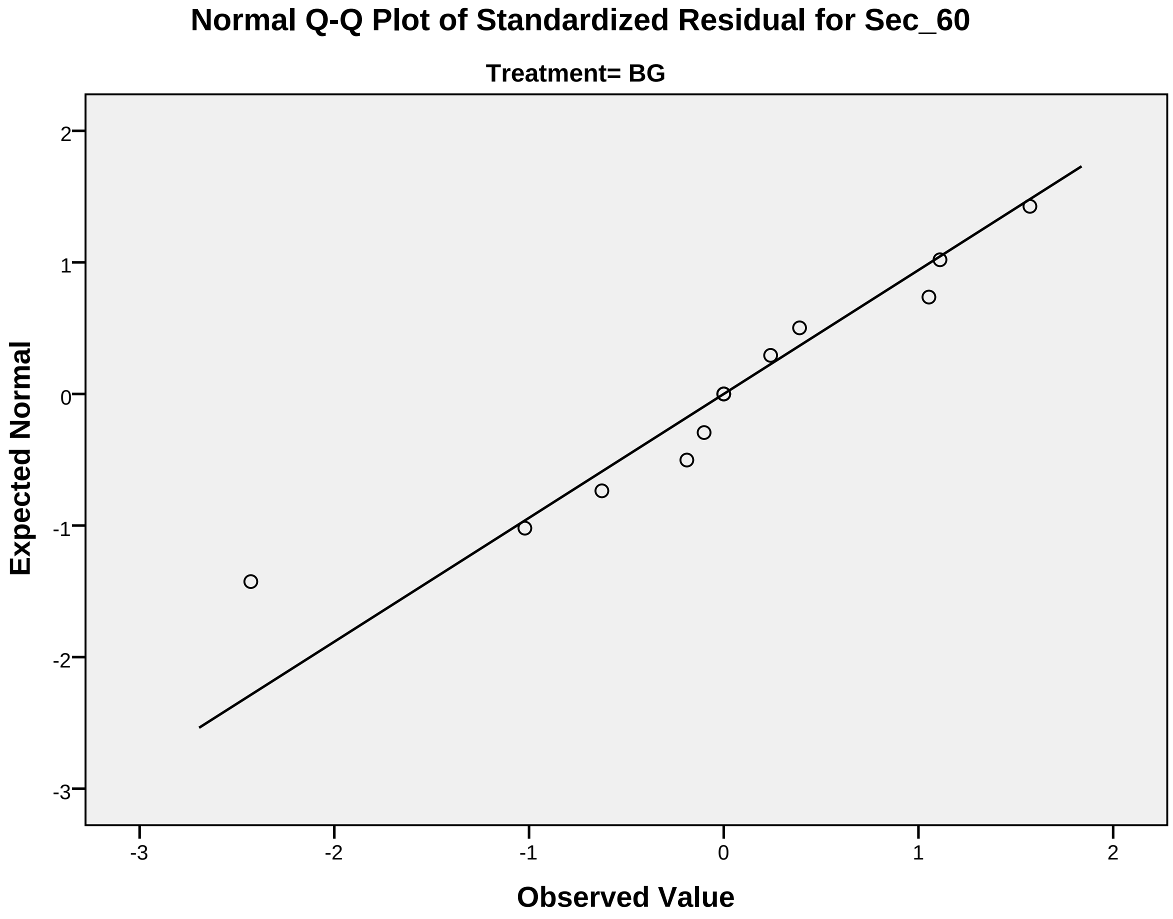
Normal Q-Q Plot of Standardized Residual for Sec_60
Treatment= BG
Expected Normal
2
1
0
-1
-2
-3
-3
-2
-1
0
1
2
Observed Value

Detrended Normal Q-Q Plot of Standardized Residual for Pre

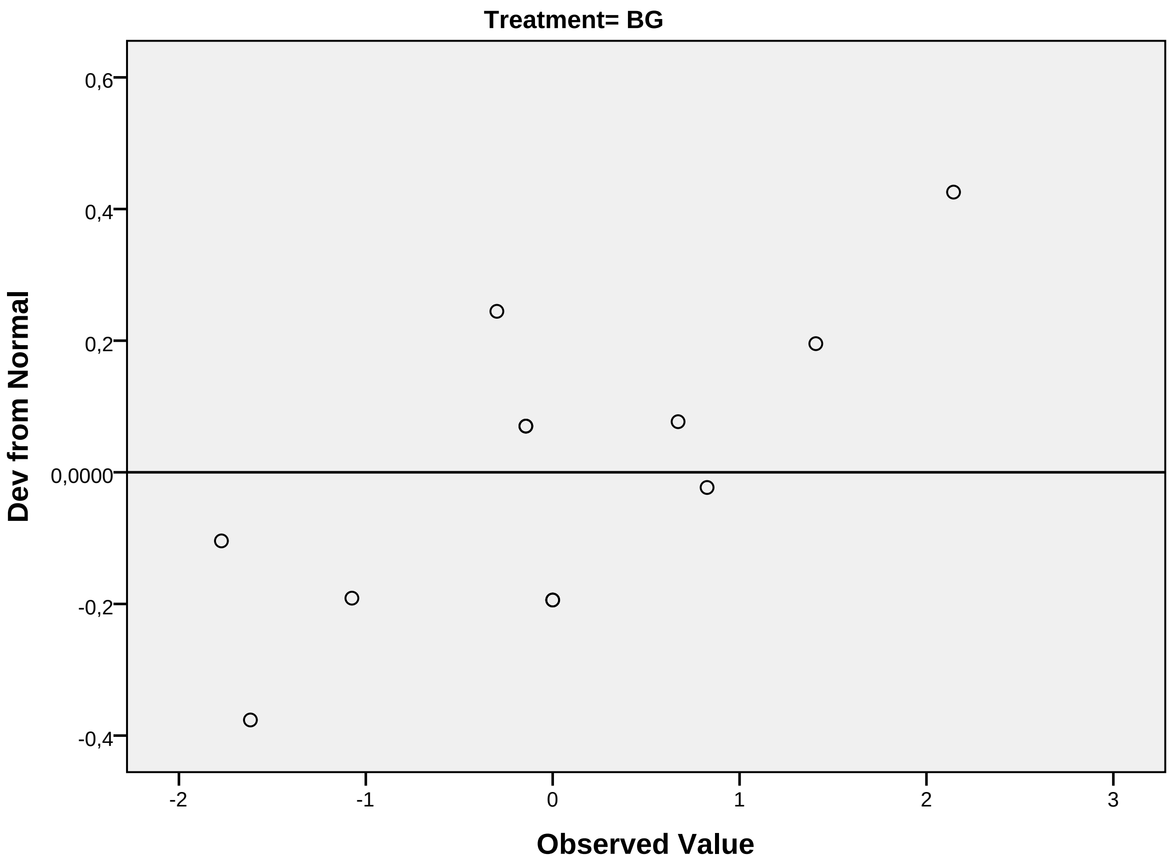

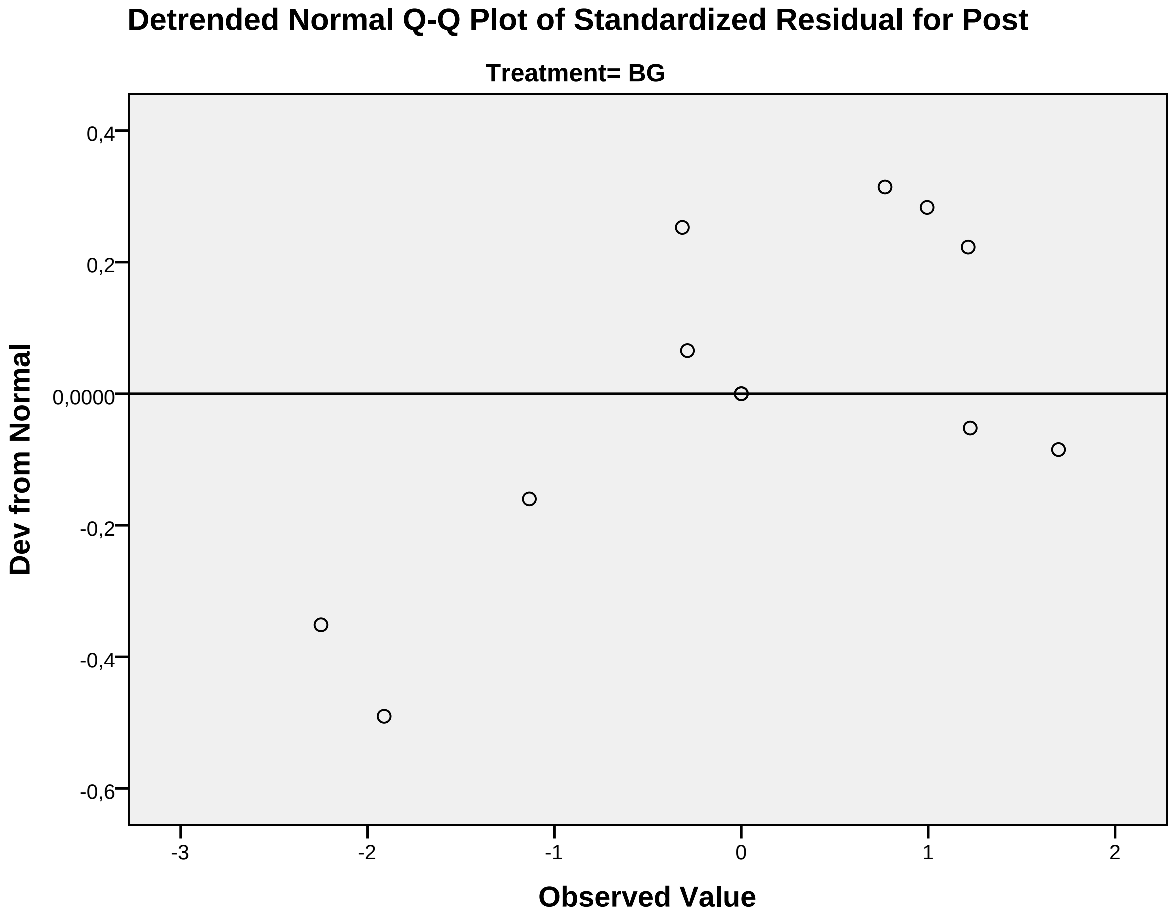

Detrended Normal Q-Q Plot of Standardized Residual for Post
Treatment= BG
Dev from Normal
0,4
0,2
0,0000
-0,2
-0,4
-0,6
-3
-2
-1
0
1
2
Observed Value

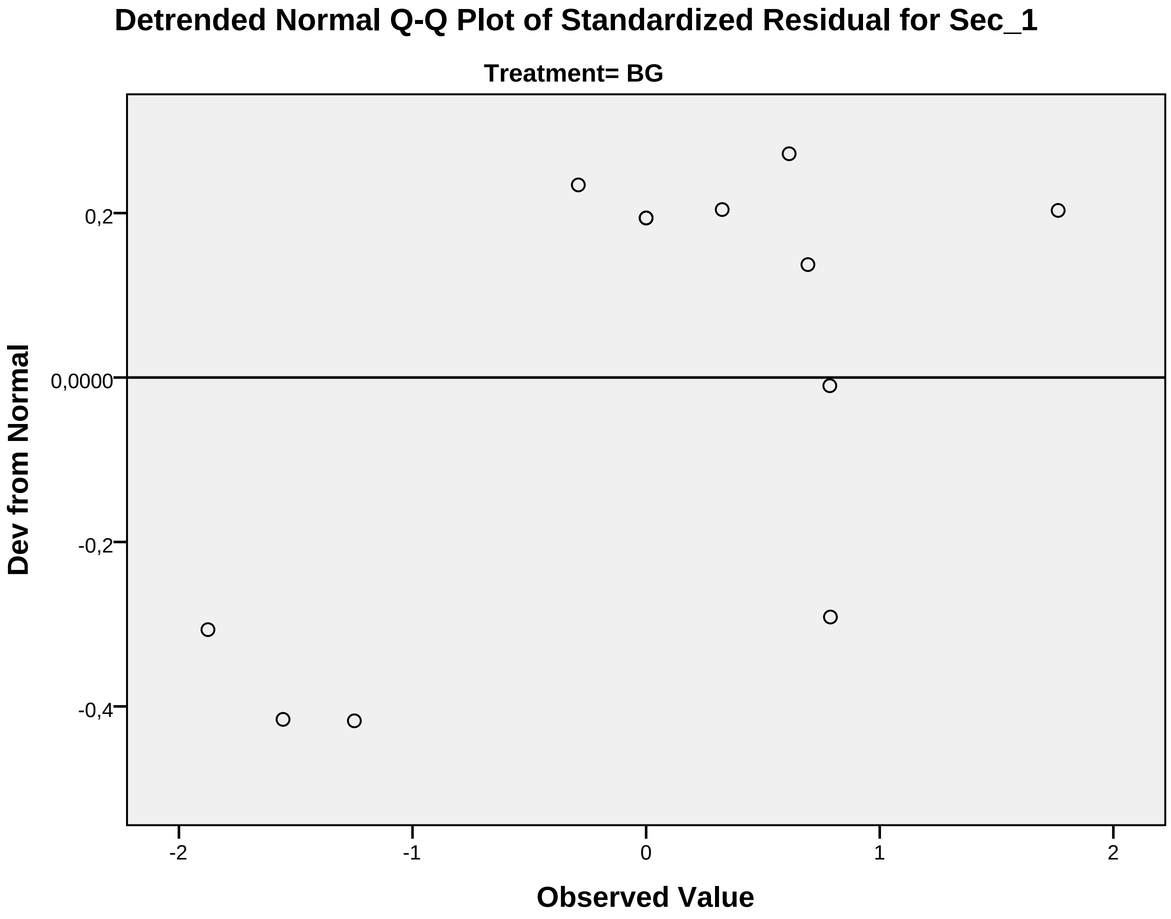

Detrended Normal Q-Q Plot of Standardized Residual for Sec_1
Treatment= BG
Dev from Normal
0,2
0,0000
-0,2
-0,4
-2
-1
0
1
2
Observed Value

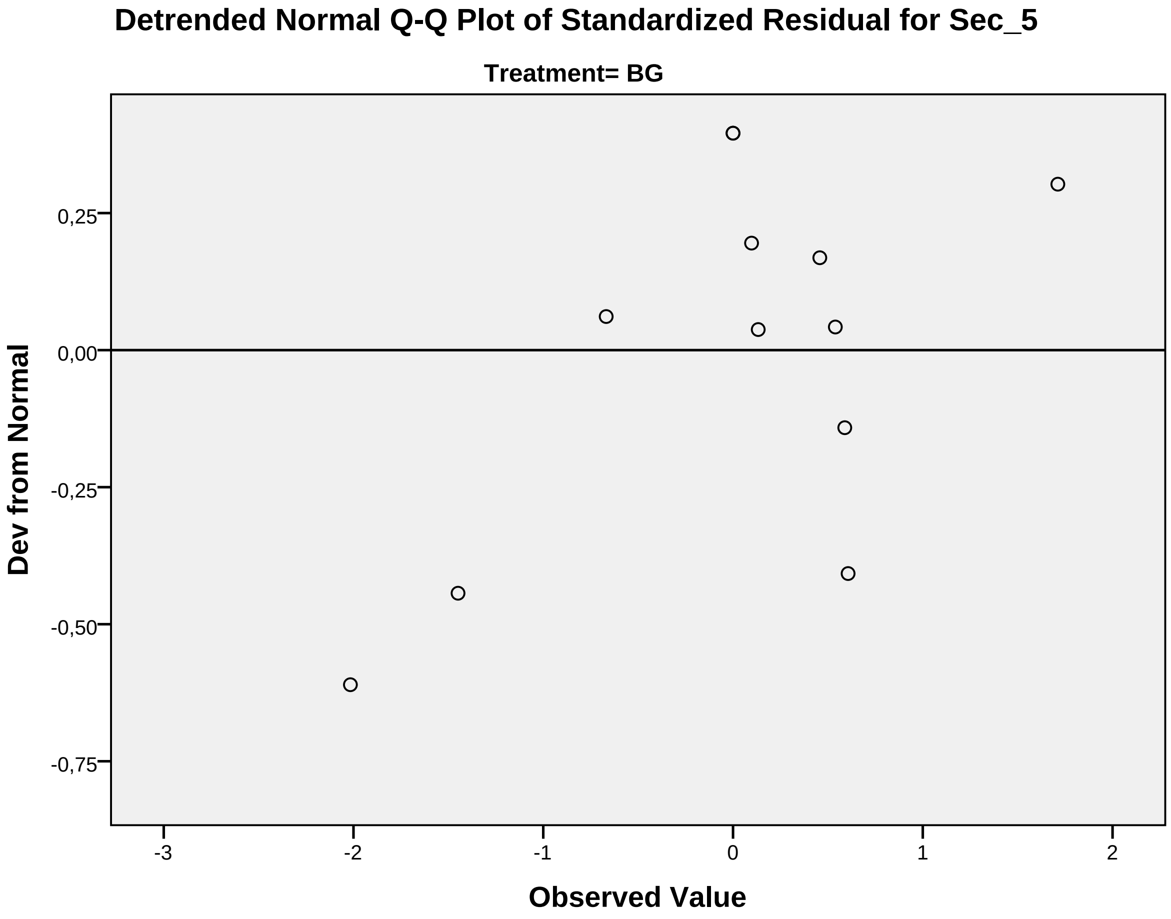
Detrended Normal Q-Q Plot of Standardized Residual for Sec_5
Treatment= BG
Dev from Normal
0,25
0,00
-0,25
-0,50
-0,75
-3
-2
-1
0
1
2
Observed Value

Detrended Normal Q-Q Plot of Standardized Residual for Sec_10

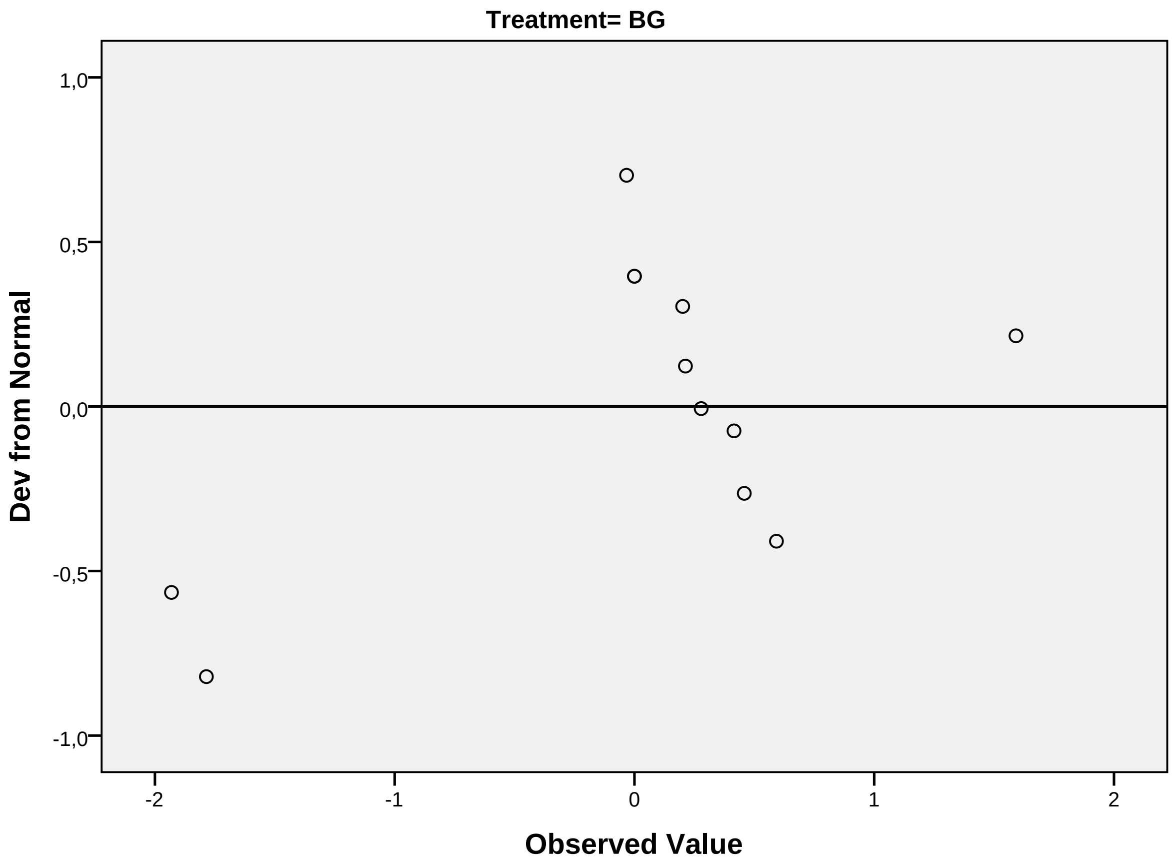

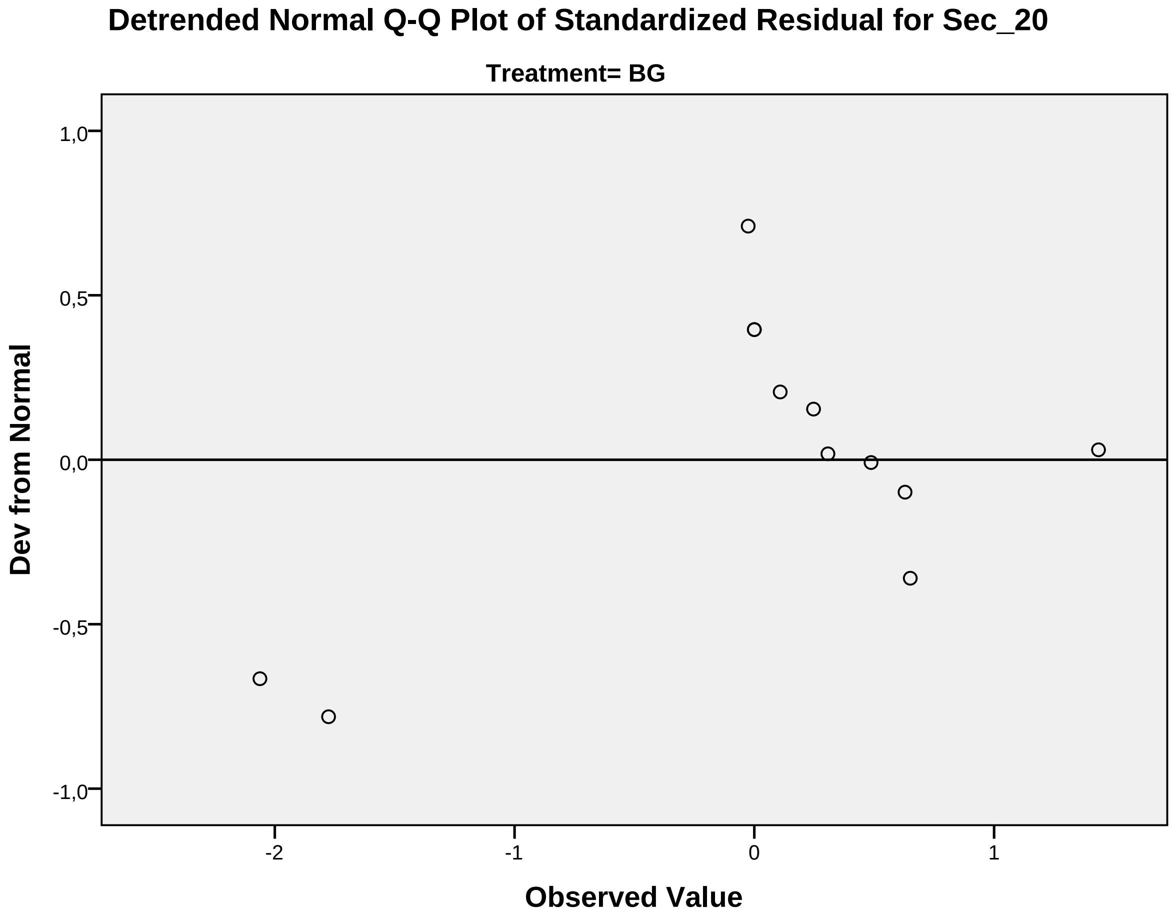

Detrended Normal Q-Q Plot of Standardized Residual for Sec_20
Treatment= BG
Dev from Normal
1,0
0,5
0,0
-0,5
-1,0
-2
-1
0
1
Observed Value

Detrended Normal Q-Q Plot of Standardized Residual for Sec_30

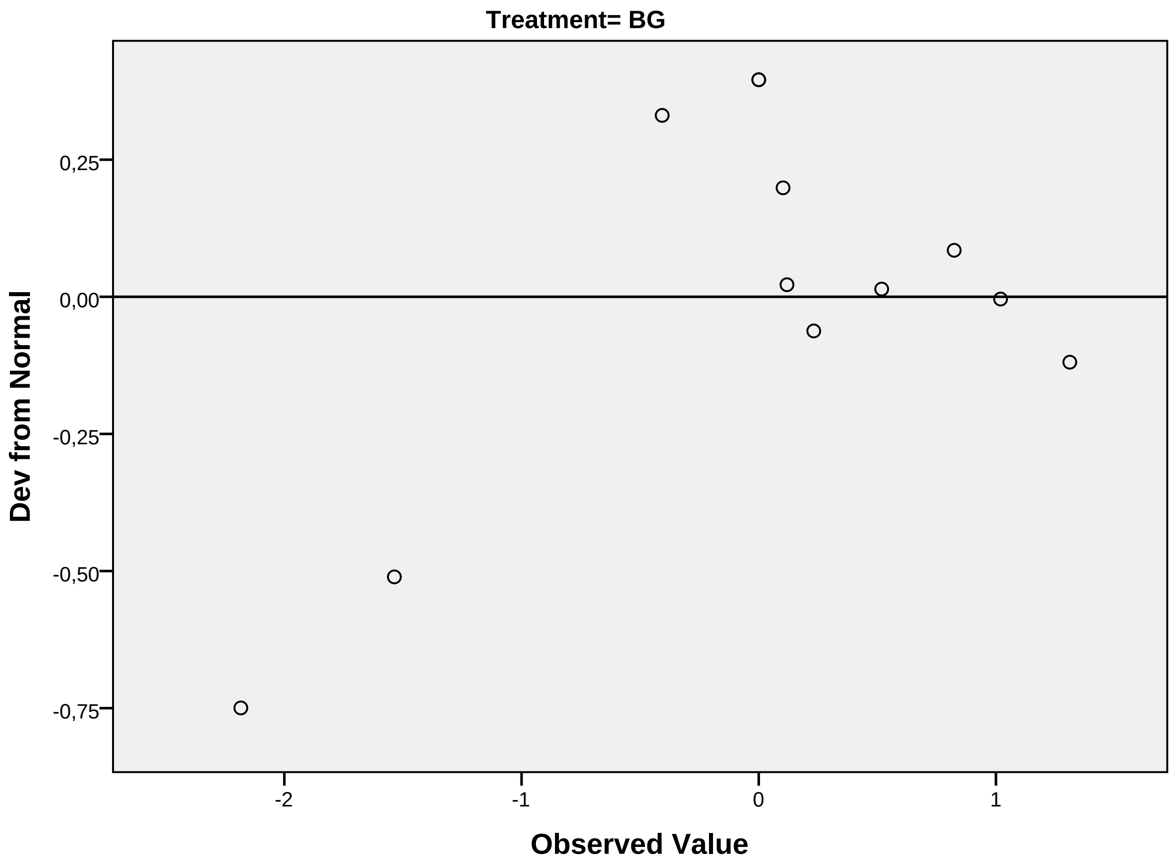

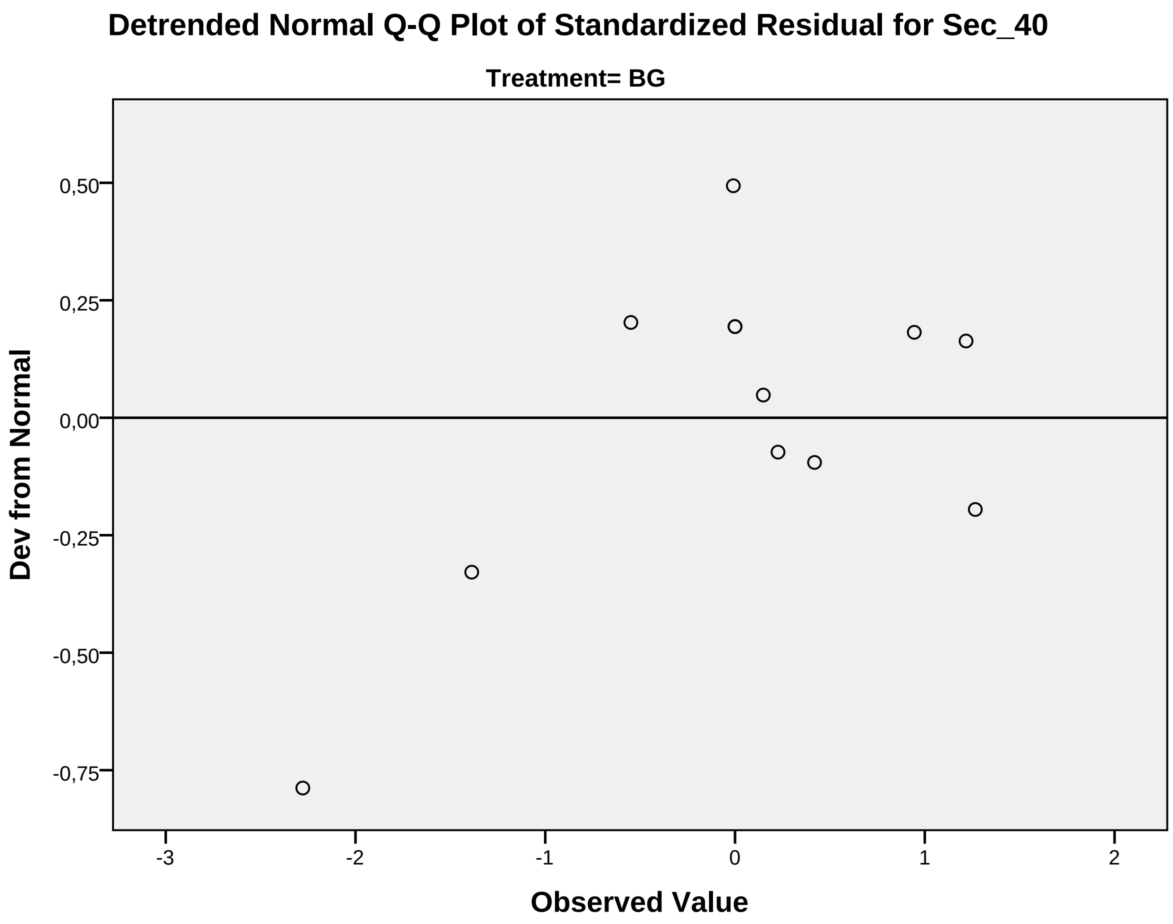
Detrended Normal Q-Q Plot of Standardized Residual for Sec_40
Treatment= BG
Dev from Normal
0,50
0,25
0,00
-0,25
-0,50
-0,75
-3
-2
-1
0
1
2
Observed Value

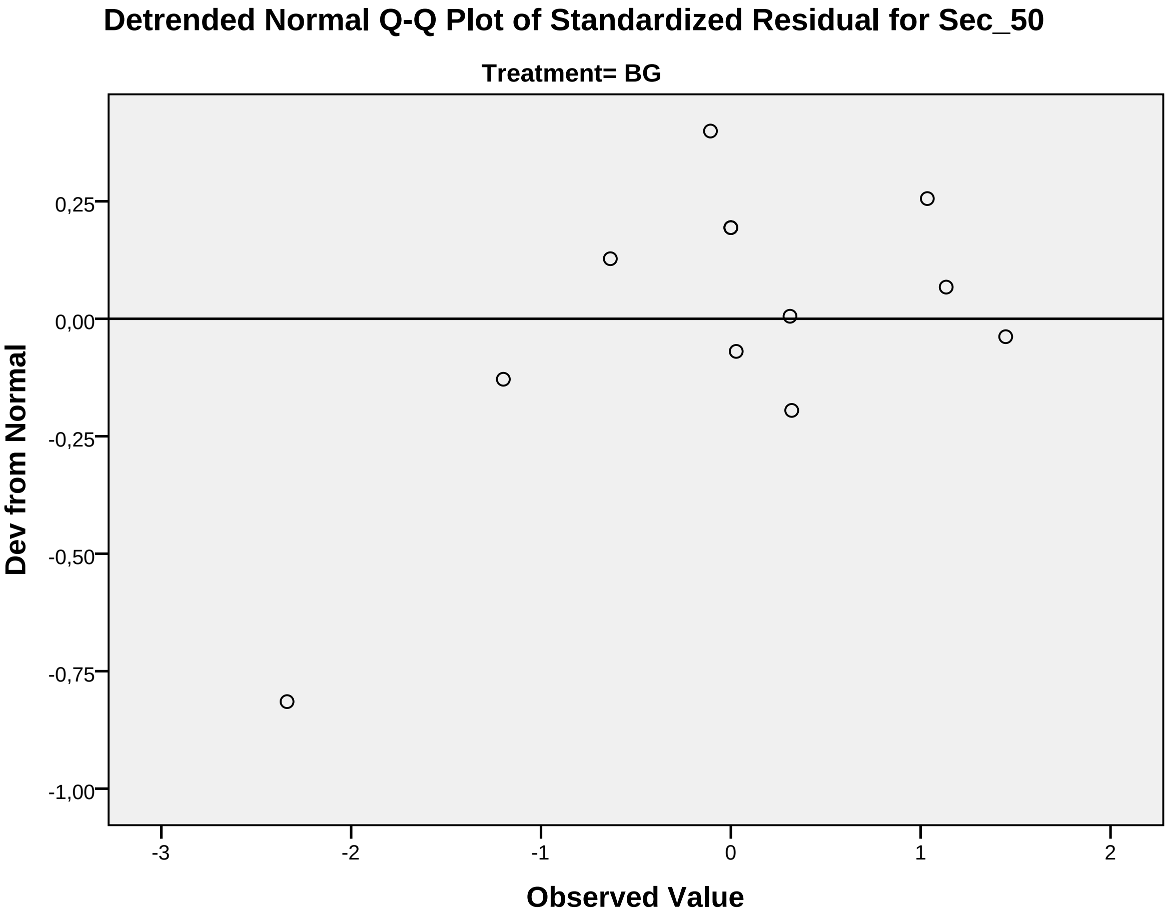
Detrended Normal Q-Q Plot of Standardized Residual for Sec_50
Treatment= BG
Dev from Normal
0,25
0,00
-0,25
-0,50
-0,75
-1,00
-3
-2
-1
0
1
2
Observed Value

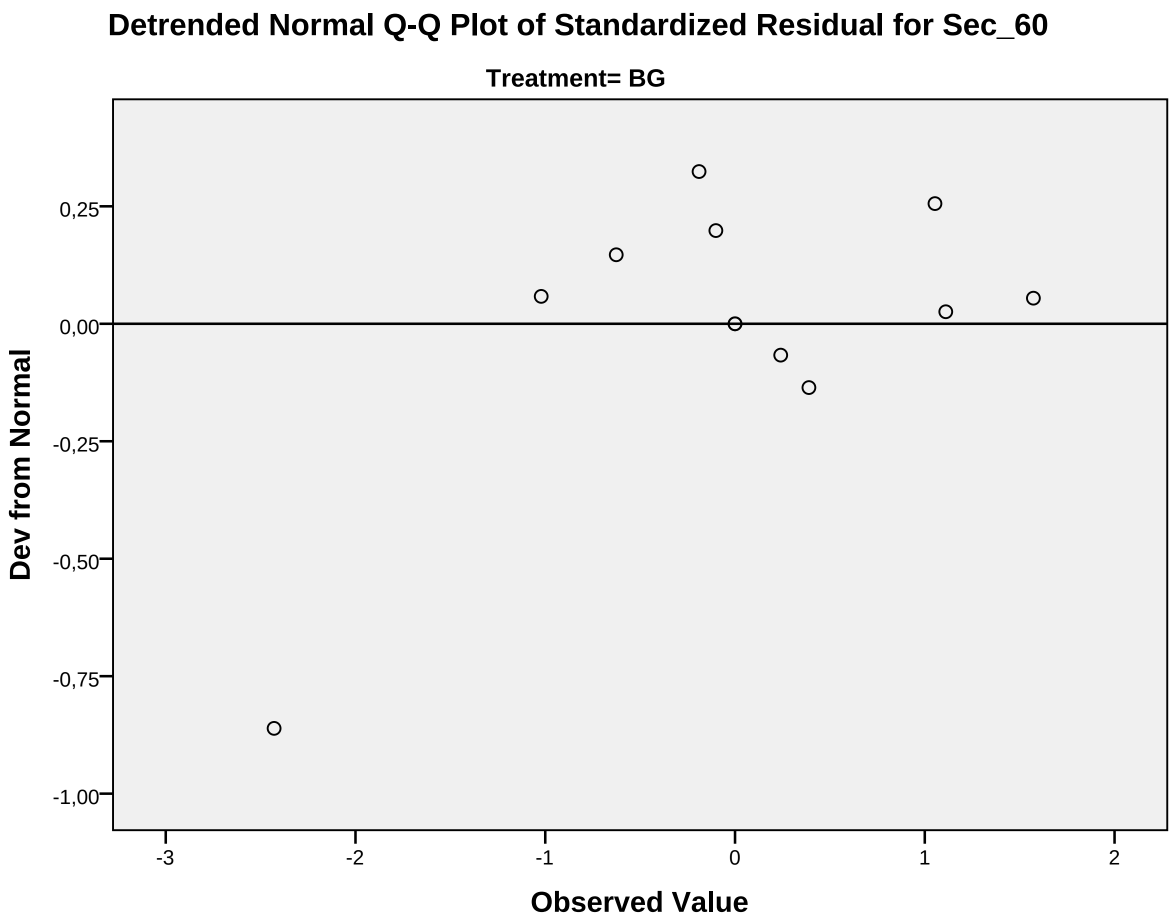
Detrended Normal Q-Q Plot of Standardized Residual for Sec_60
Treatment= BG
Dev from Normal
0,25
0,00
-0,25
-0,50
-0,75
-1,00
-3
-2
-1
0
1
2
Observed Value

Treatment: BG

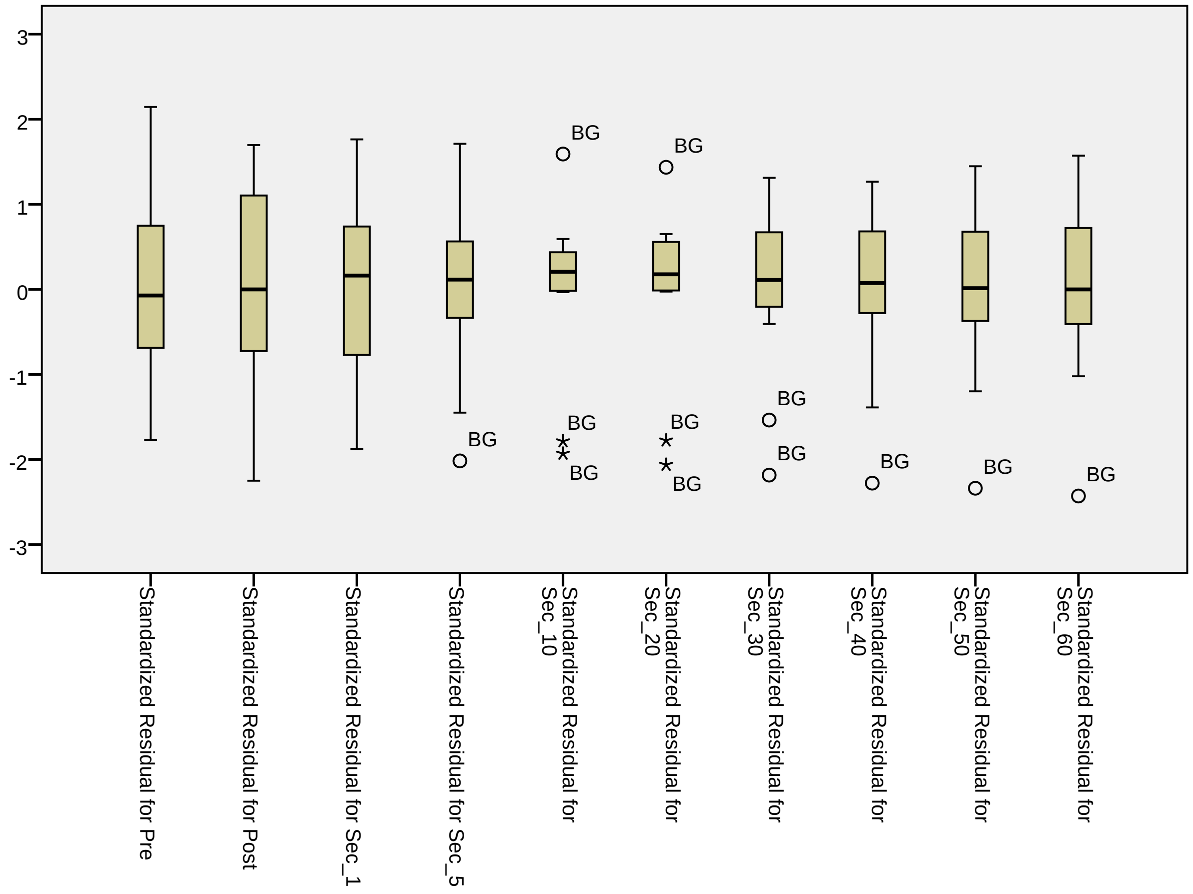

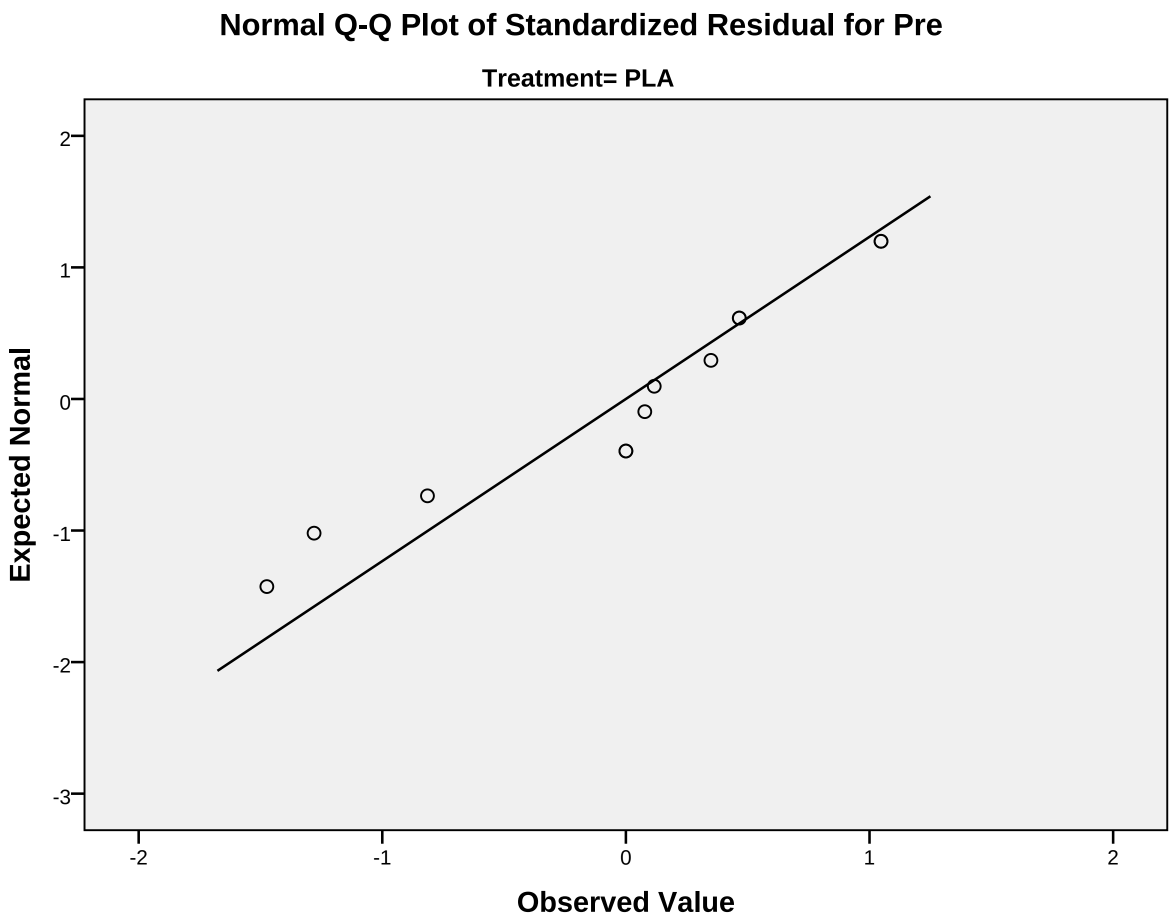
Normal Q-Q Plot of Standardized Residual for Pre
Treatment= PLA
Expected Normal
2
1
0
-1
-2
-3
-2
-1
0
1
2
Observed Value

Normal Q-Q Plot of Standardized Residual for Post

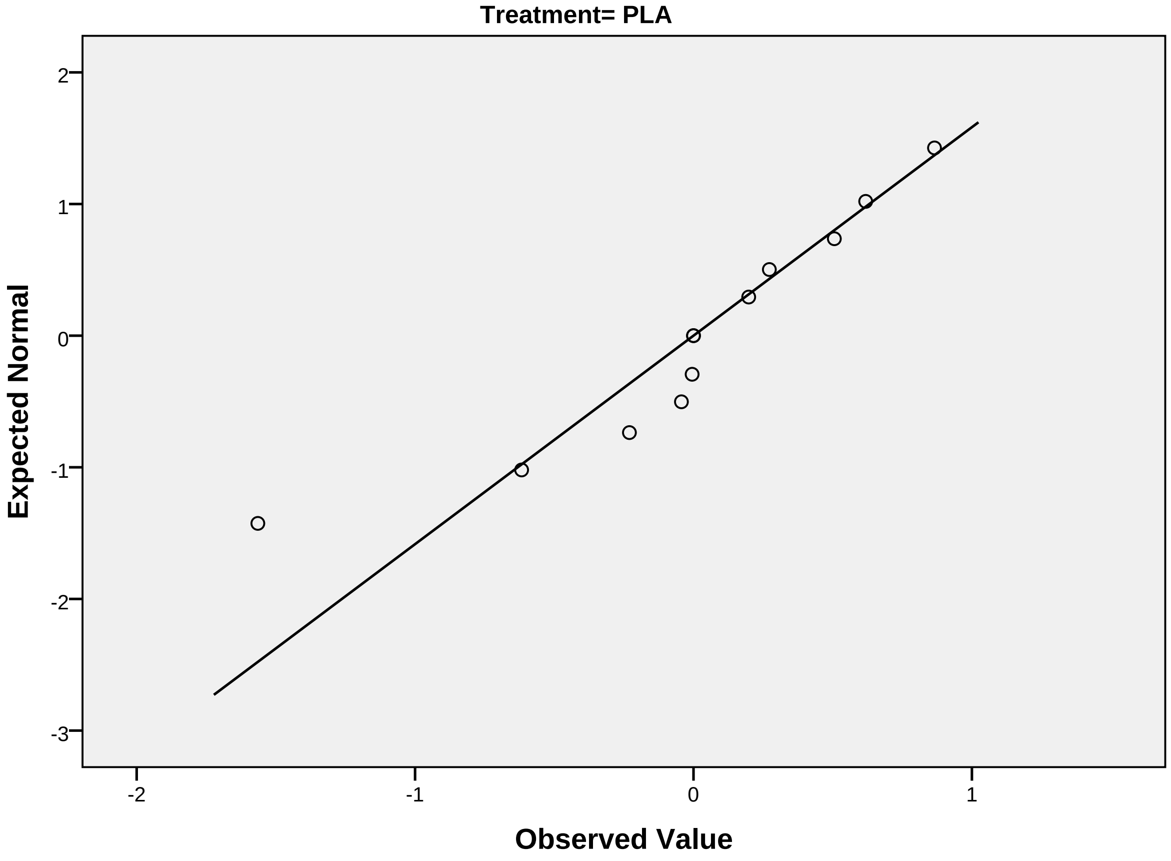

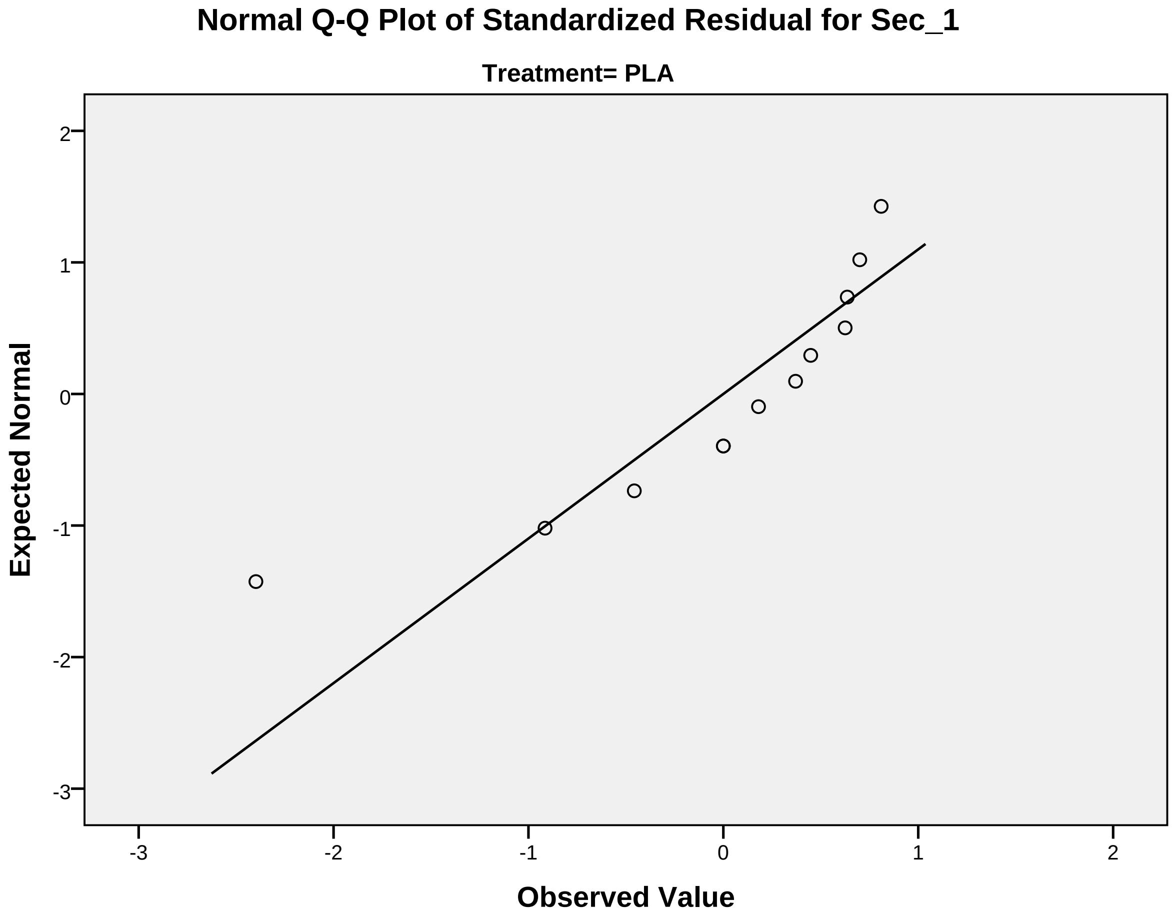

Normal Q-Q Plot of Standardized Residual for Sec_1
Treatment= PLA
Expected Normal
2
1
0
-1
-2
-3
-3
-2
-1
0
1
2
Observed Value

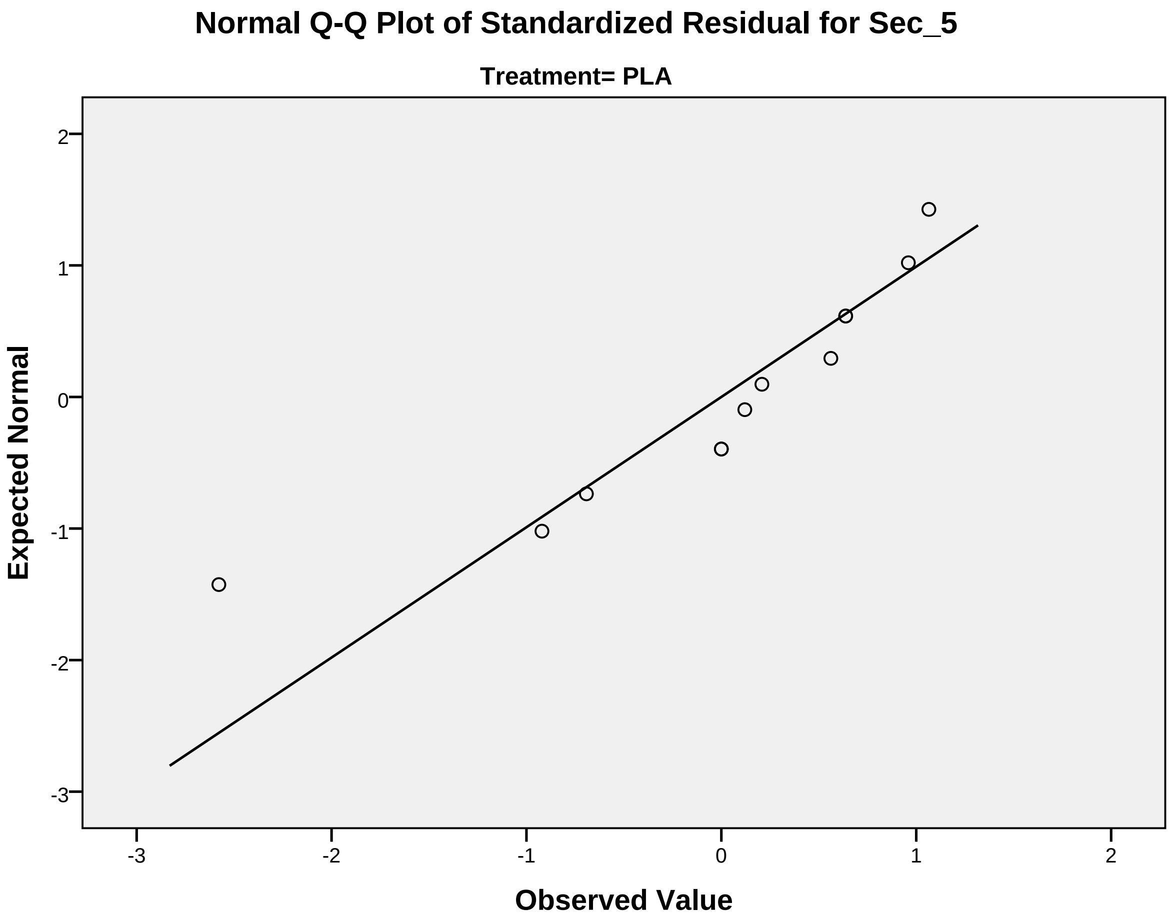
Normal Q-Q Plot of Standardized Residual for Sec_5
Treatment= PLA
Expected Normal
2
1
0
-1
-2
-3
-3
-2
-1
0
1
2
Observed Value

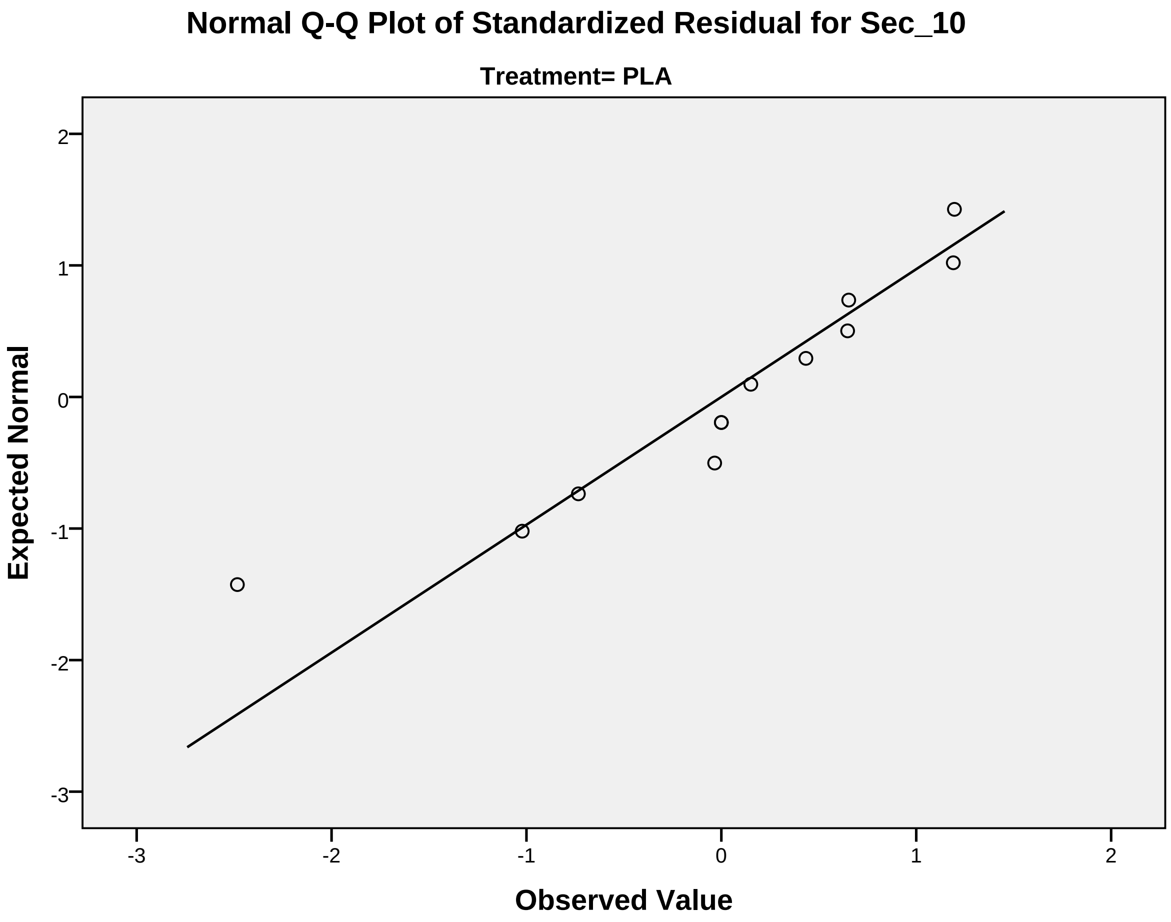
Normal Q-Q Plot of Standardized Residual for Sec_10
Treatment= PLA
Expected Normal
Observed Value
2
1
0
-1
-2
-3
-3
-2
-1
0
1
2

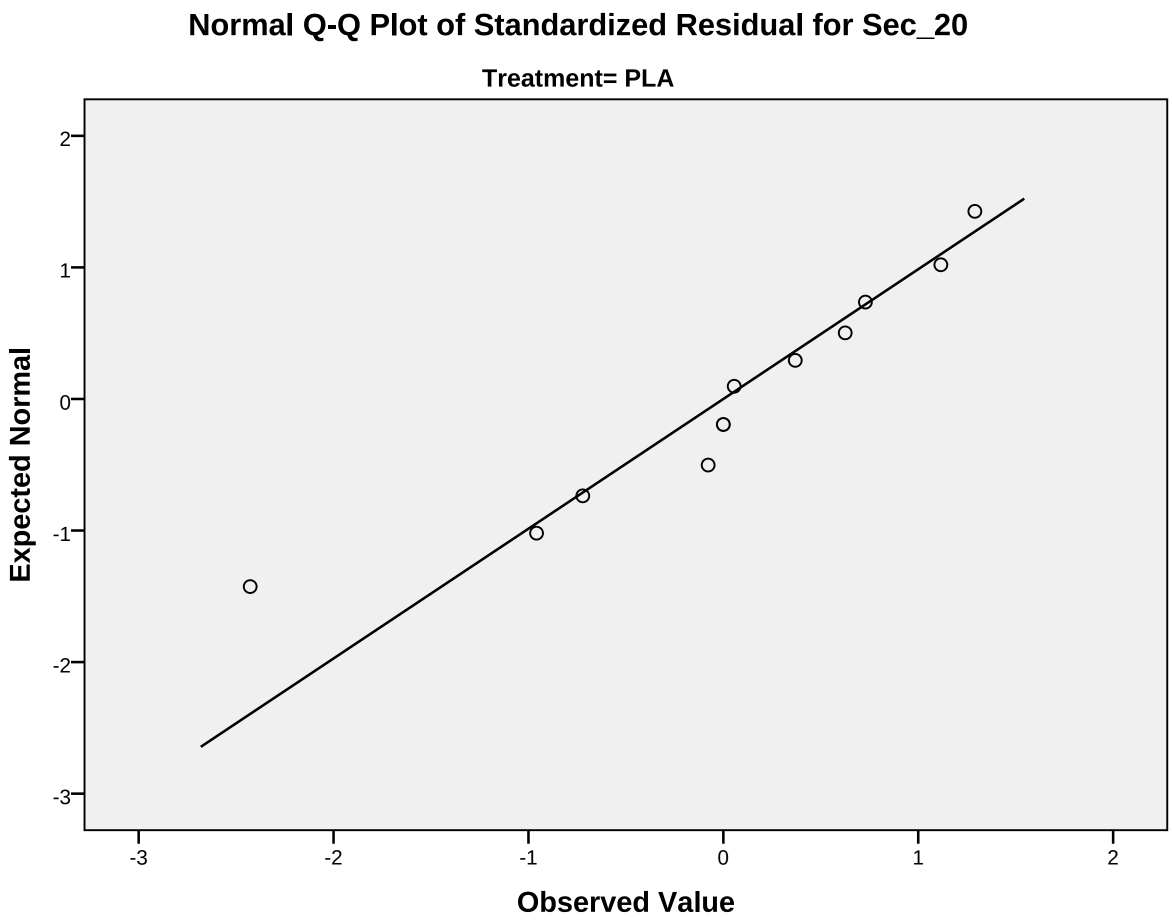
Normal Q-Q Plot of Standardized Residual for Sec_20
Treatment= PLA
Expected Normal
2
1
0
-1
-2
-3
-3
-2
-1
0
1
2
Observed Value

Normal Q-Q Plot of Standardized Residual for Sec_30

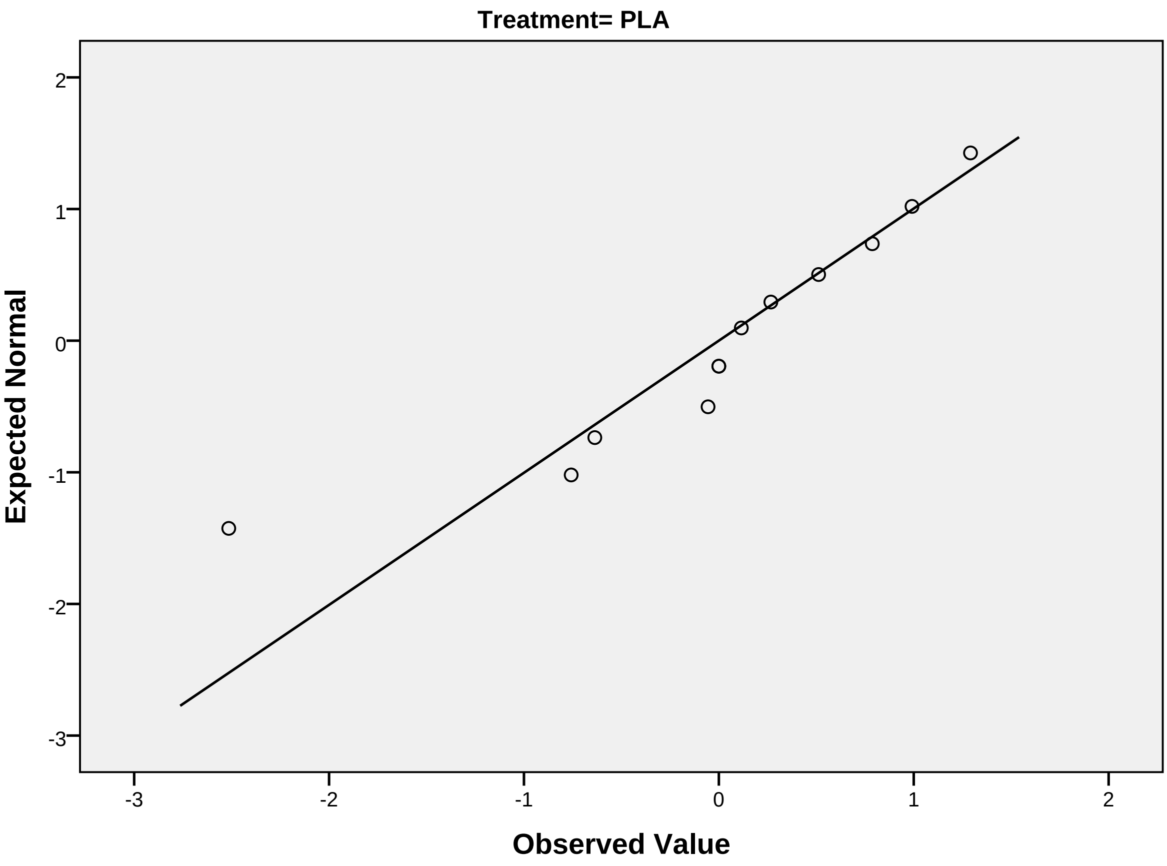

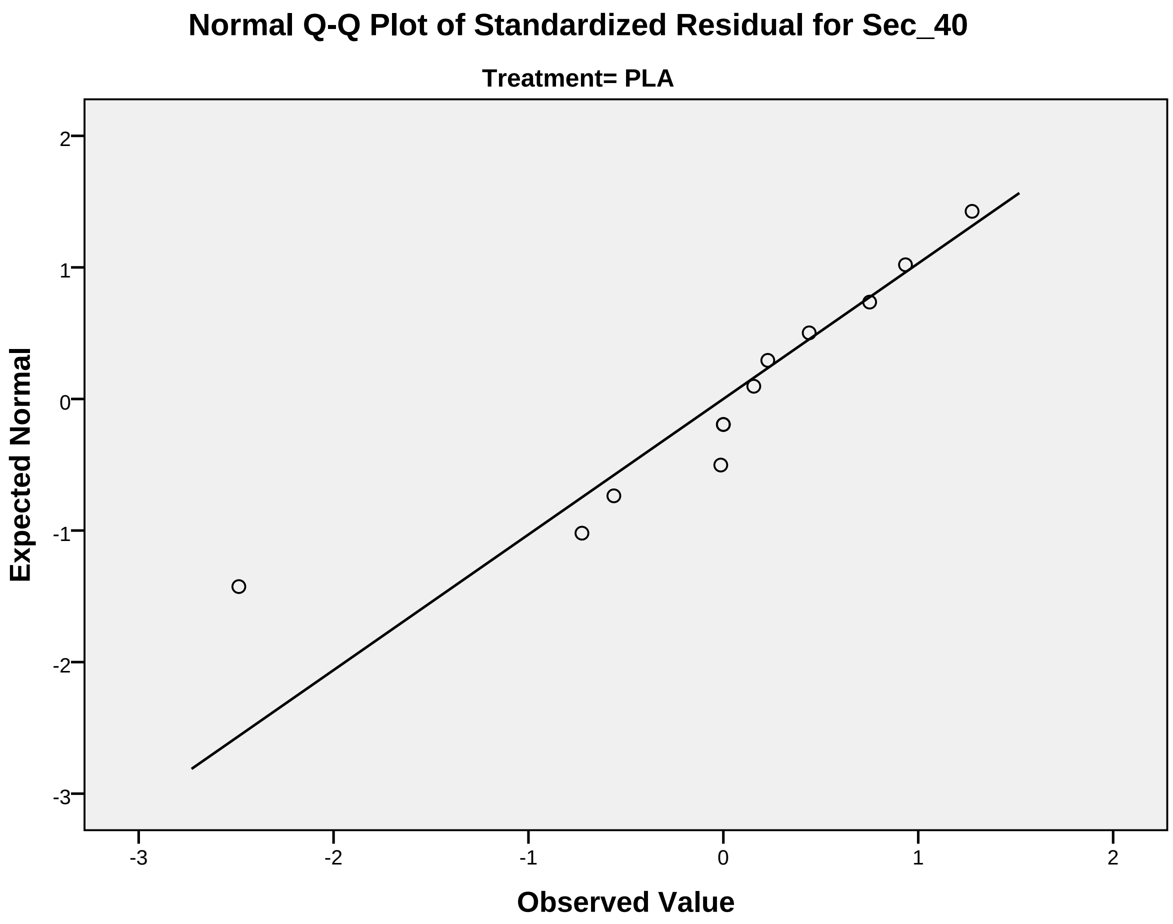
Normal Q-Q Plot of Standardized Residual for Sec_40
Treatment= PLA
Expected Normal
2
1
0
-1
-2
-3
-3
-2
-1
0
1
2
Observed Value

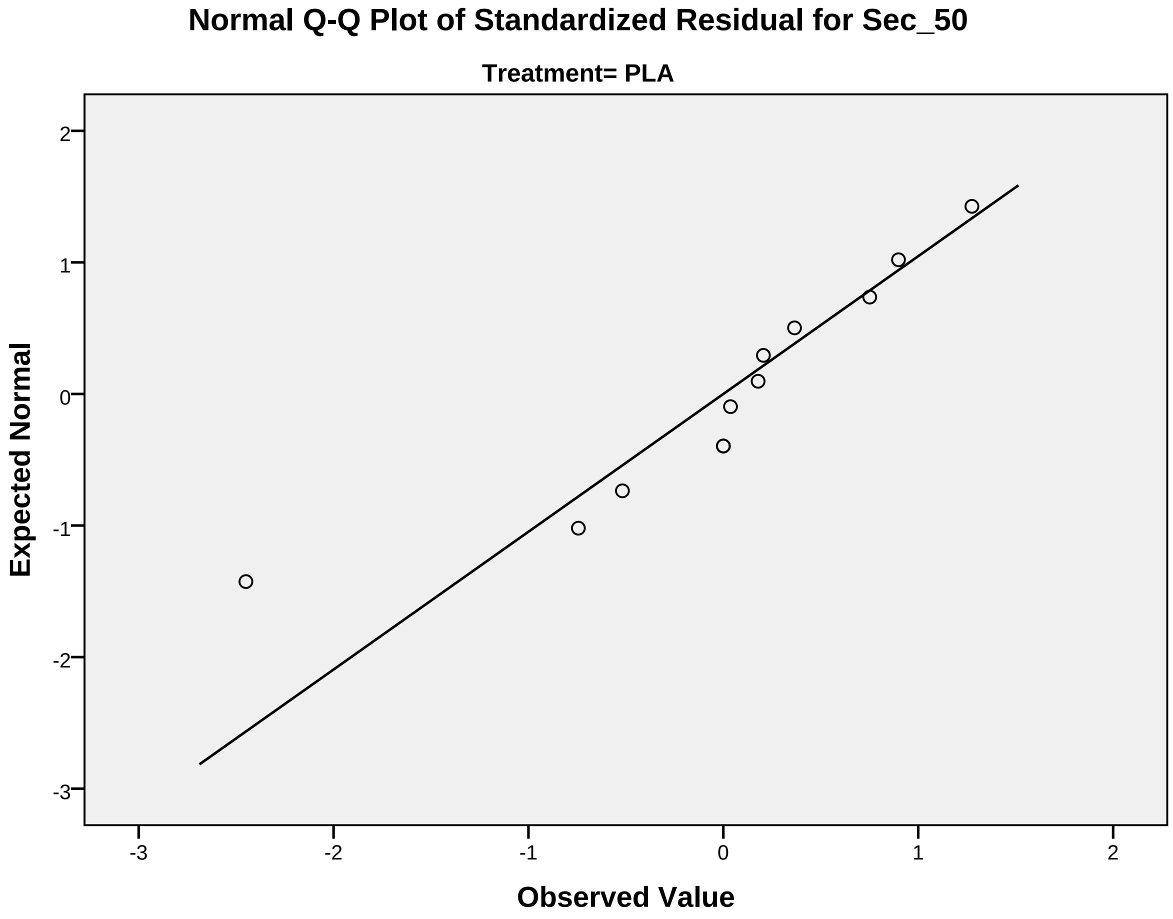
Normal Q-Q Plot of Standardized Residual for Sec_50
Treatment= PLA
Expected Normal
2
1
0
-1
-2
-3
-3
-2
-1
0
1
2
Observed Value

Normal Q-Q Plot of Standardized Residual for Sec_60

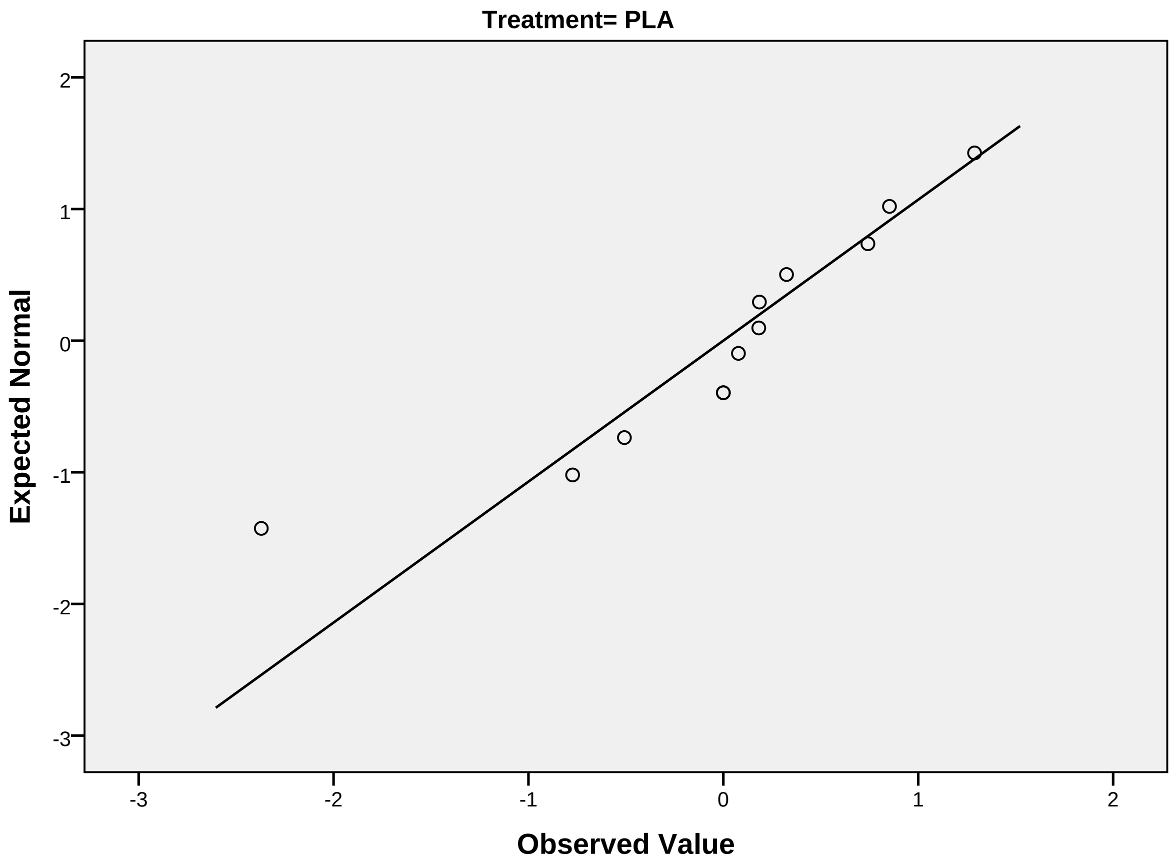

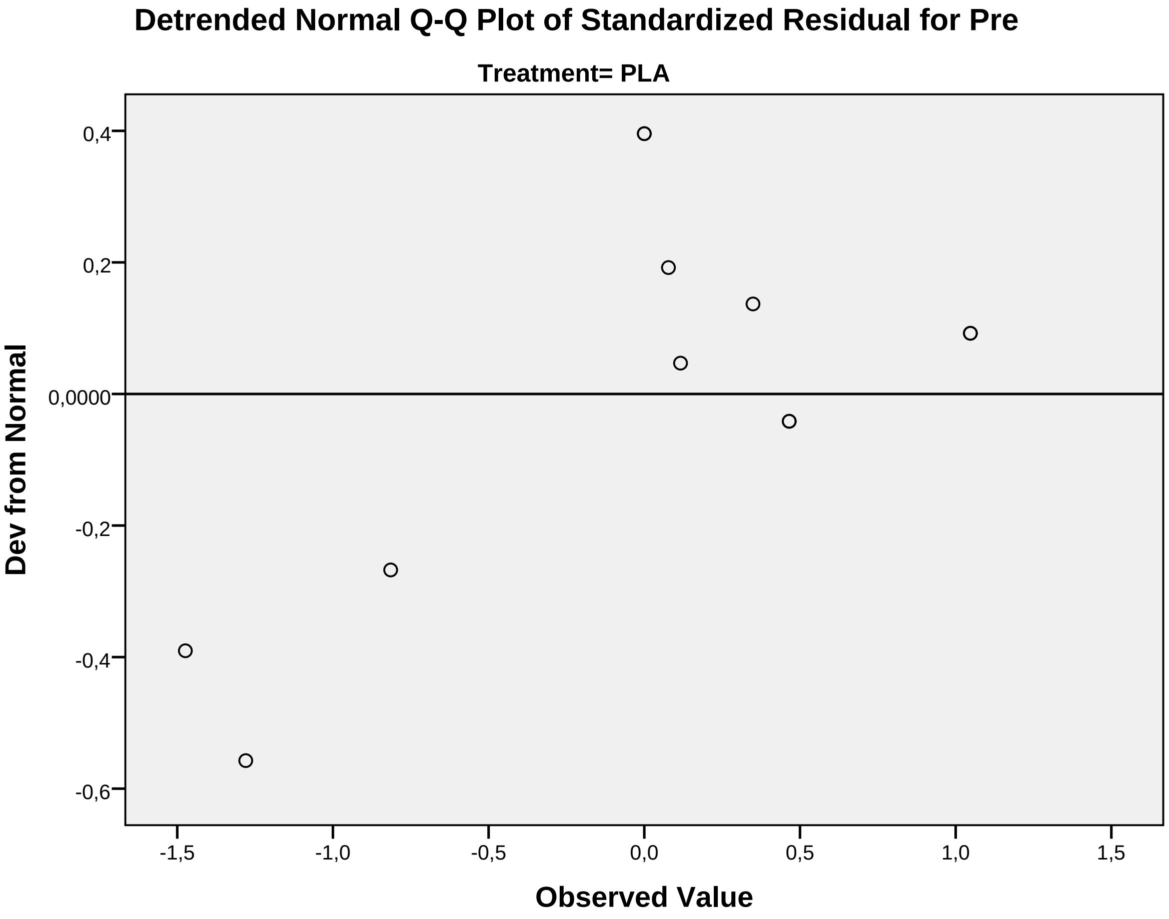
Detrended Normal Q-Q Plot of Standardized Residual for Pre
Treatment= PLA
Dev from Normal
0,4
0,2
0,0000
-0,2
-0,4
-0,6
-1,5
-1,0
-0,5
0,0
0,5
1,0
1,5
Observed Value

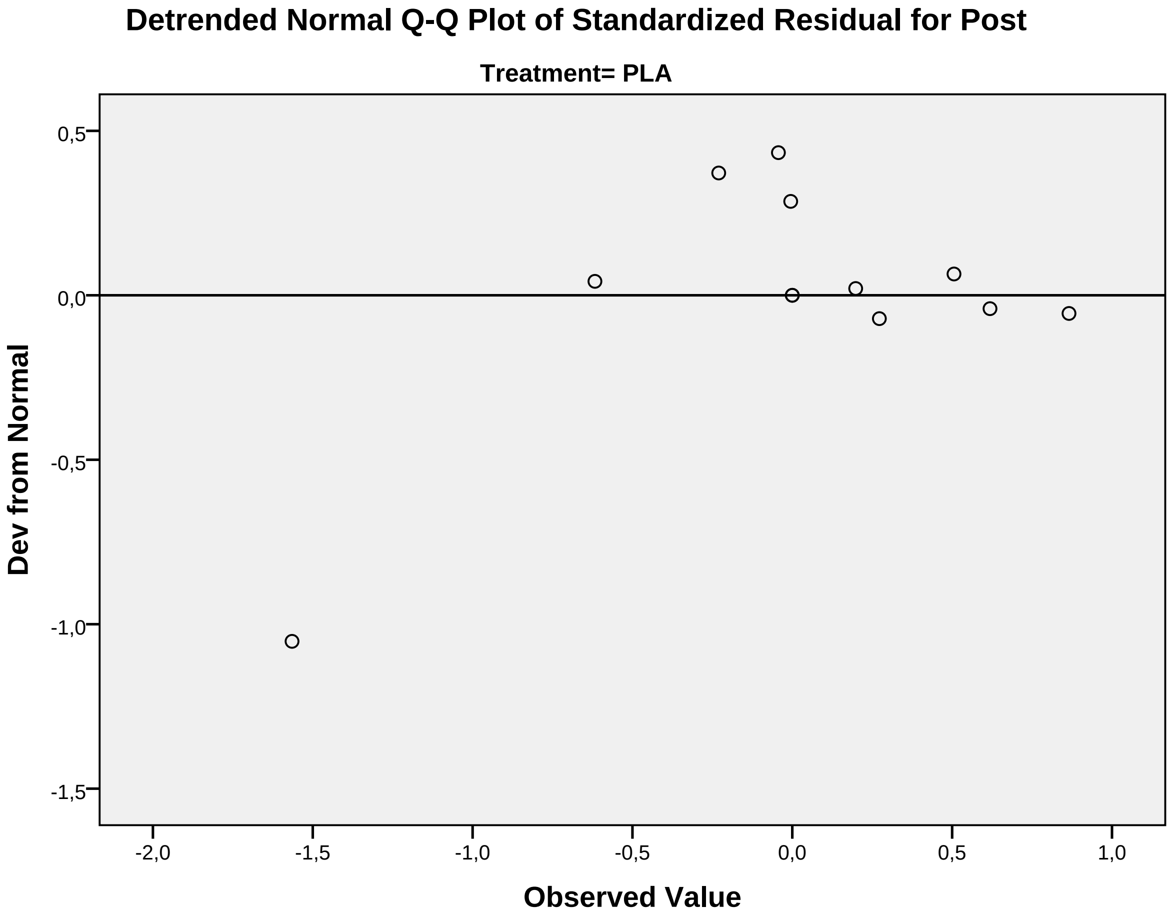
Detrended Normal Q-Q Plot of Standardized Residual for Post
Treatment= PLA
Dev from Normal
0,5
0,0
-0,5
-1,0
-1,5
-2,0
-1,5
-1,0
-0,5
0,0
0,5
1,0
Observed Value

Detrended Normal Q-Q Plot of Standardized Residual for Sec_1

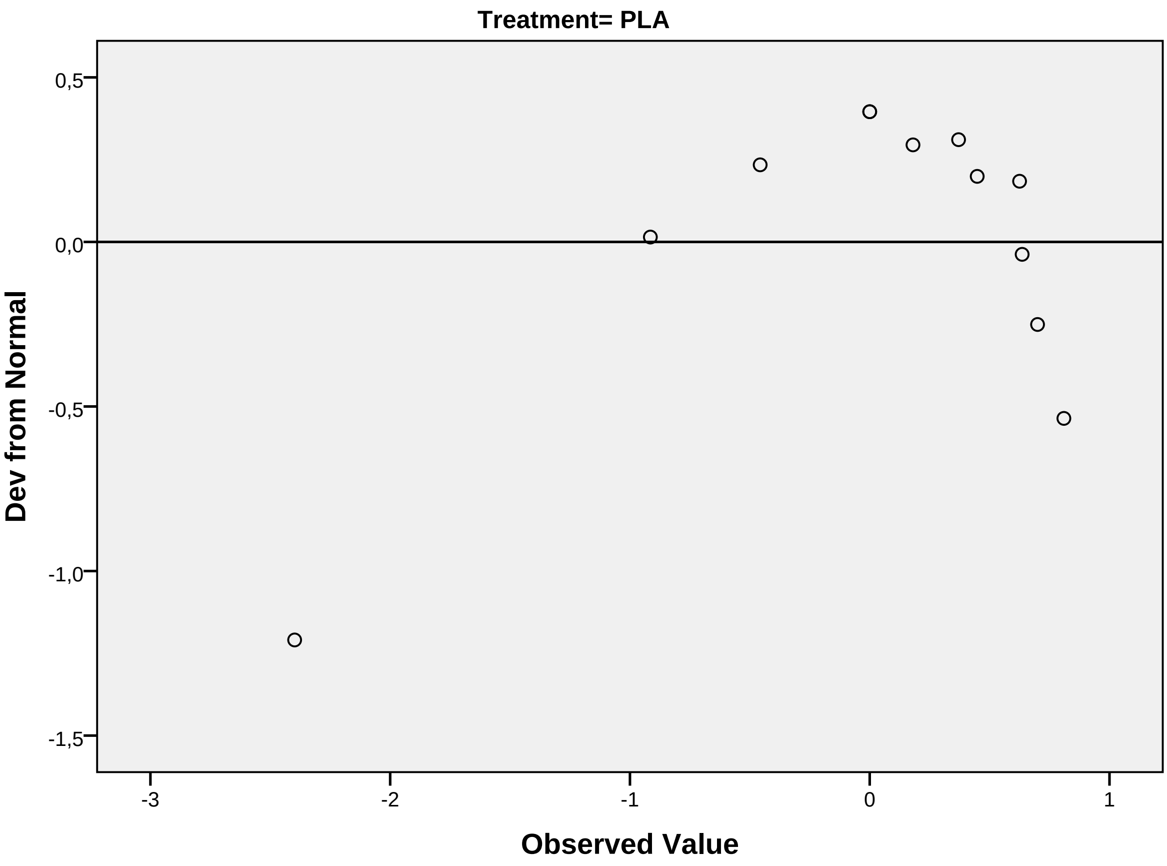

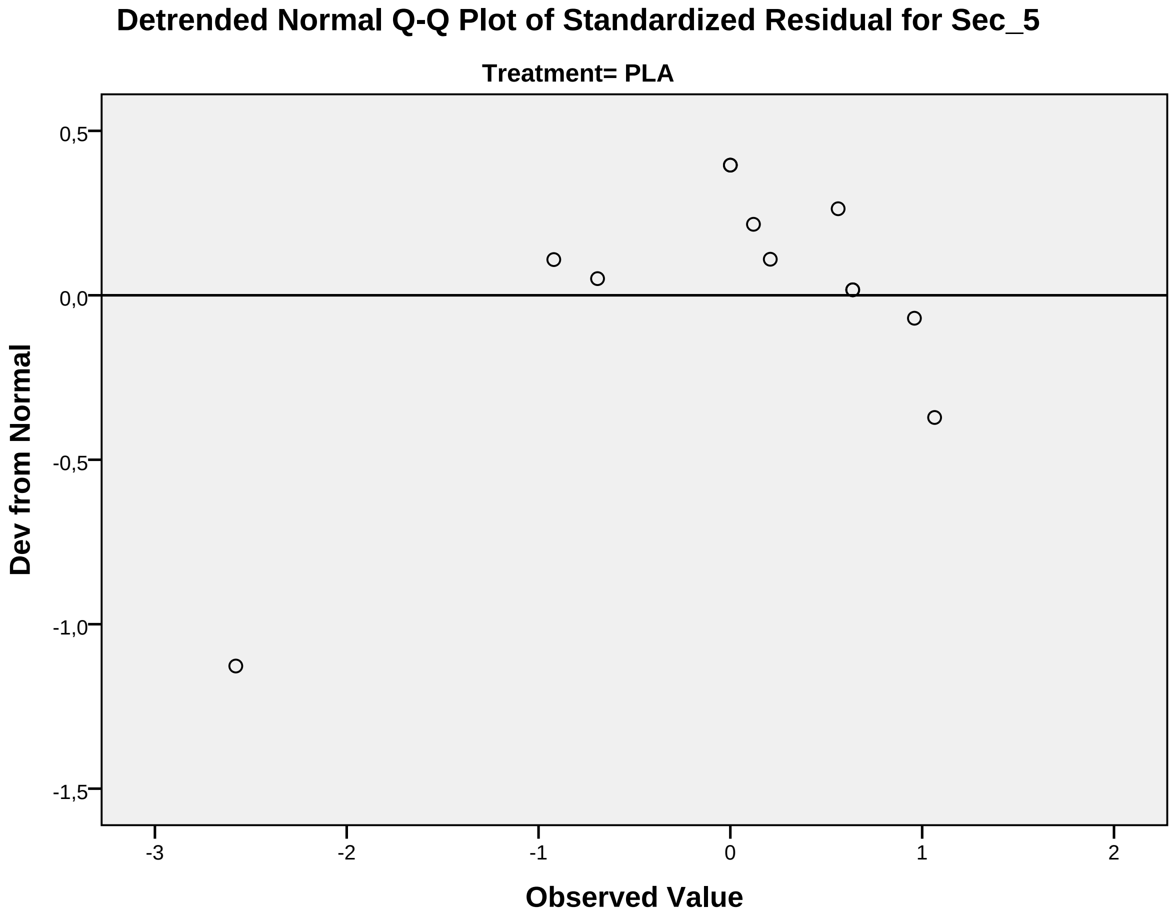

Detrended Normal Q-Q Plot of Standardized Residual for Sec_5
Treatment= PLA
Dev from Normal
0,5
0,0
-0,5
-1,0
-1,5
-3
-2
-1
0
1
2
Observed Value

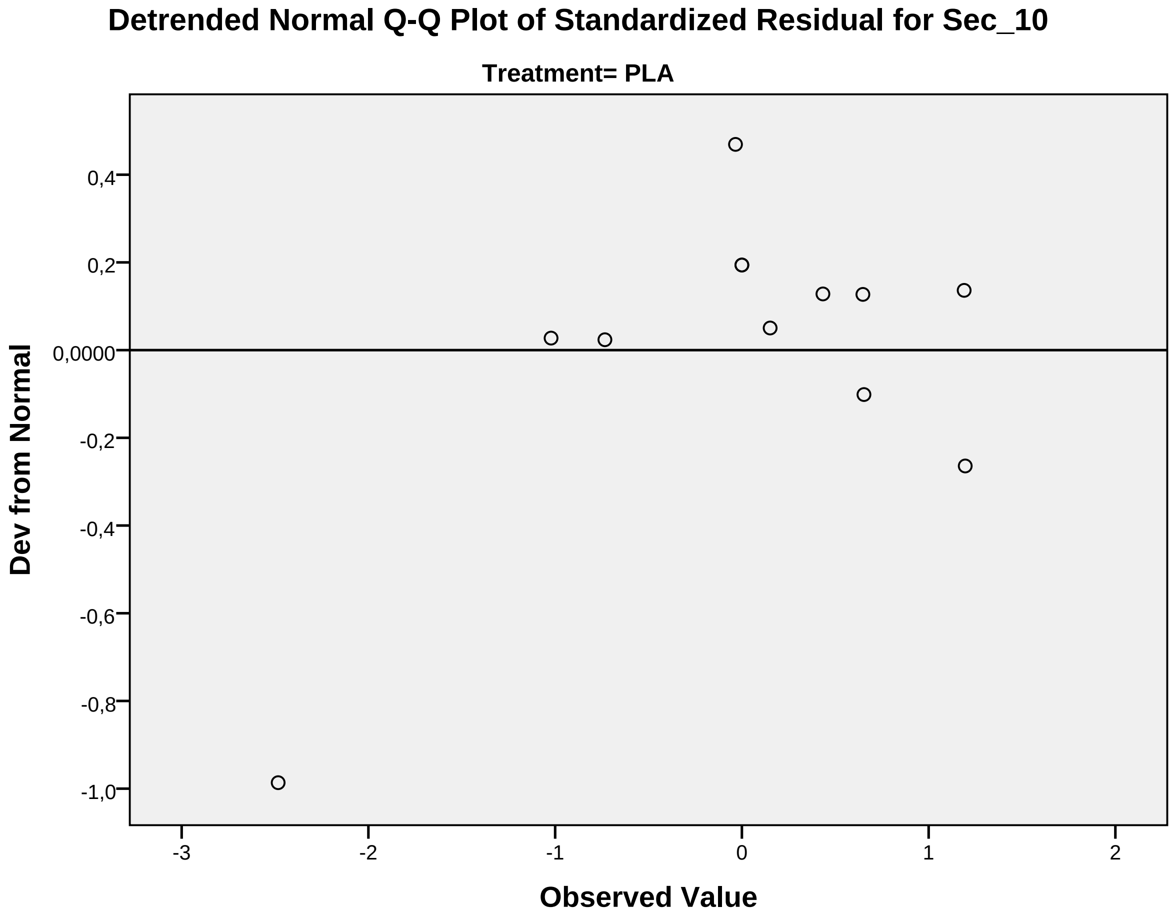
Detrended Normal Q-Q Plot of Standardized Residual for Sec_10
Treatment= PLA
Dev from Normal
0,4
0,2
0,0000
-0,2
-0,4
-0,6
-0,8
-1,0
-3
-2
-1
0
1
2
Observed Value

Detrended Normal Q-Q Plot of Standardized Residual for Sec_20

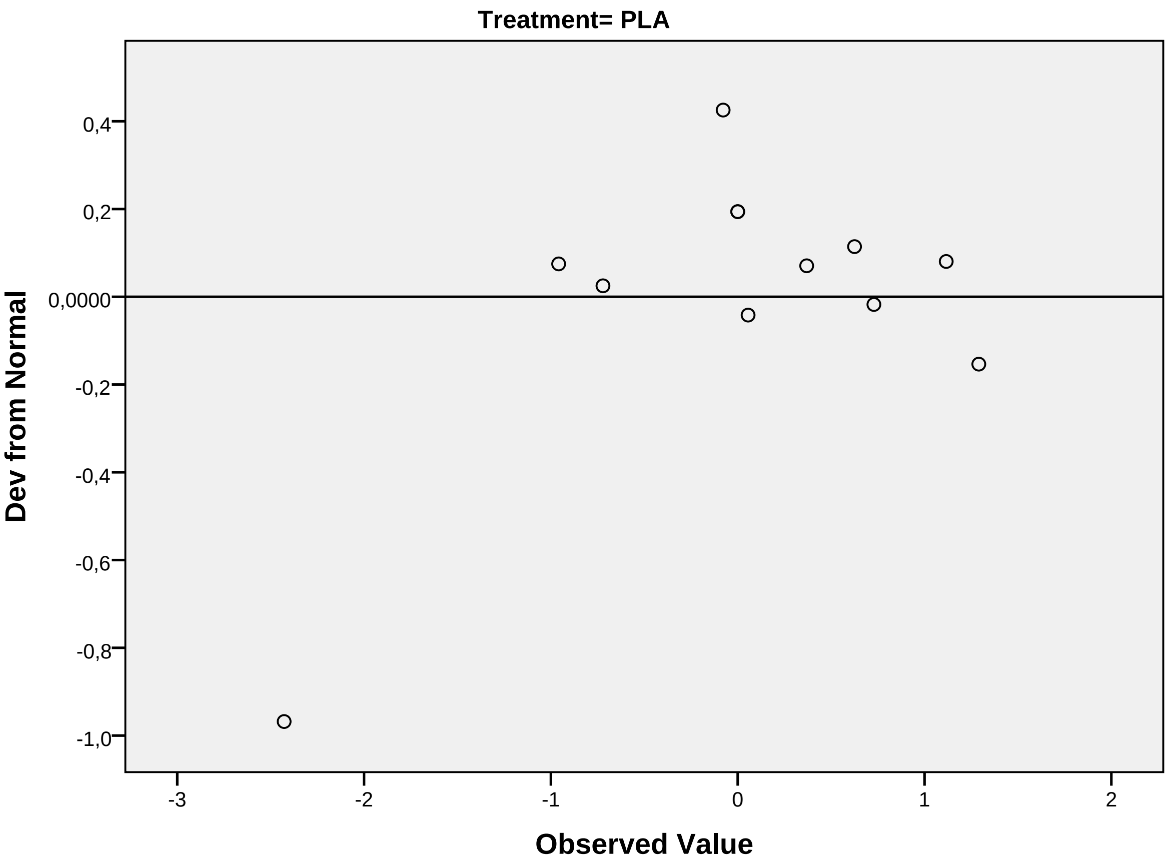

Detrended Normal Q-Q Plot of Standardized Residual for Sec_30

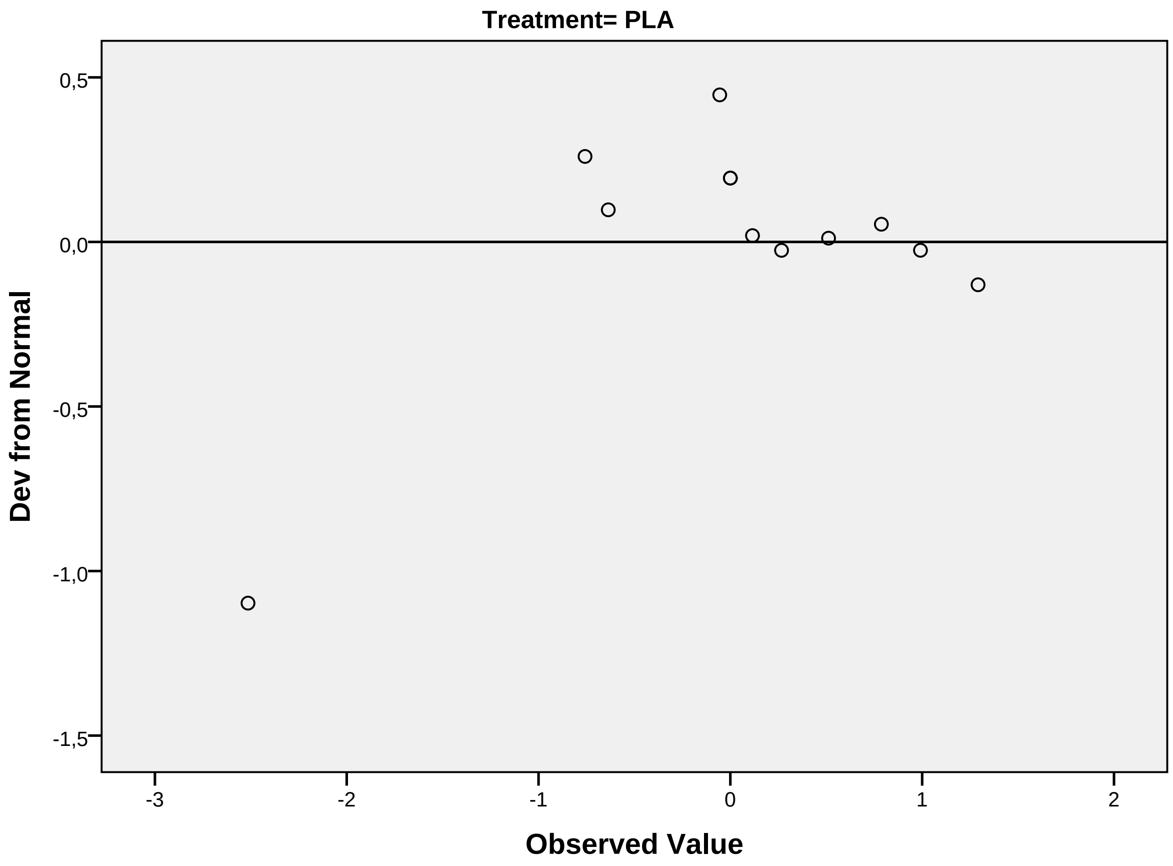

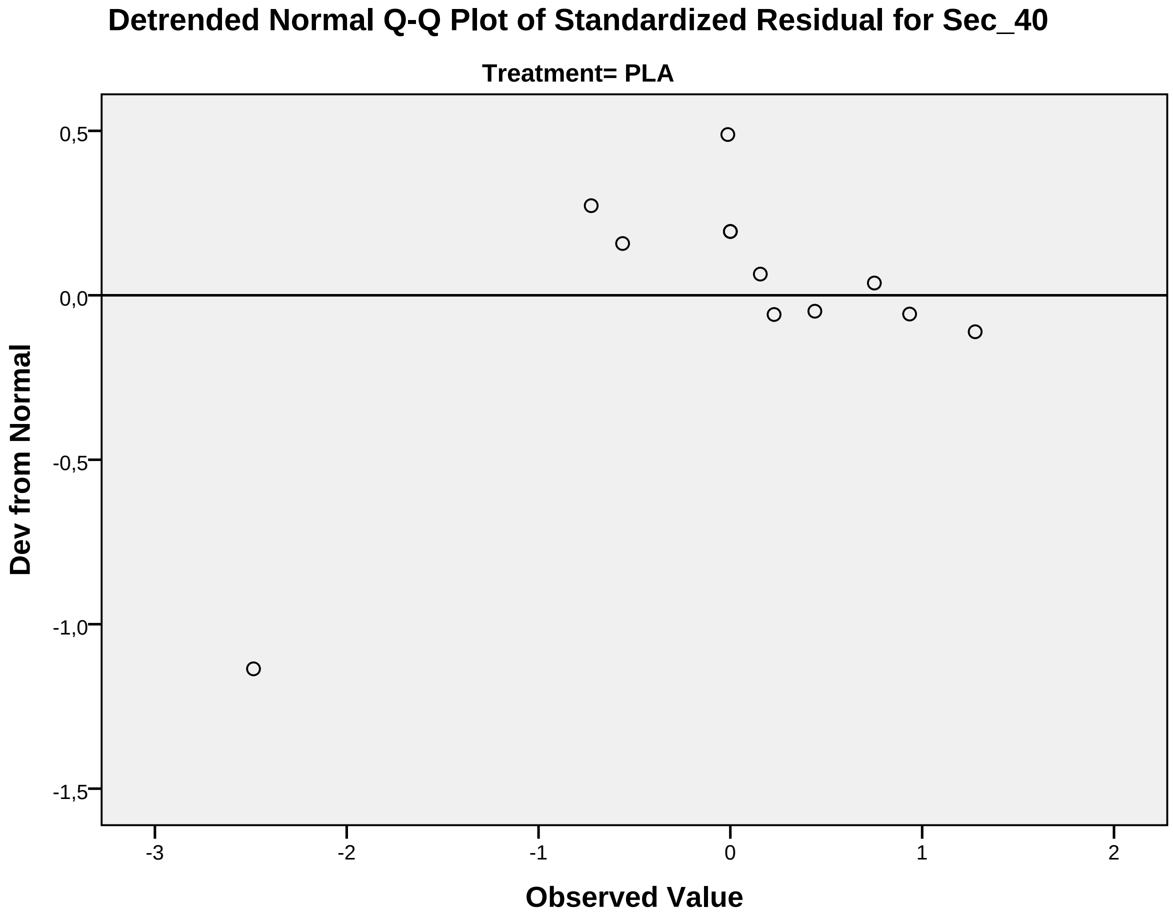

Detrended Normal Q-Q Plot of Standardized Residual for Sec_40
Treatment= PLA
Dev from Normal
0,5
0,0
-0,5
-1,0
-1,5
-3
-2
-1
0
1
2
Observed Value

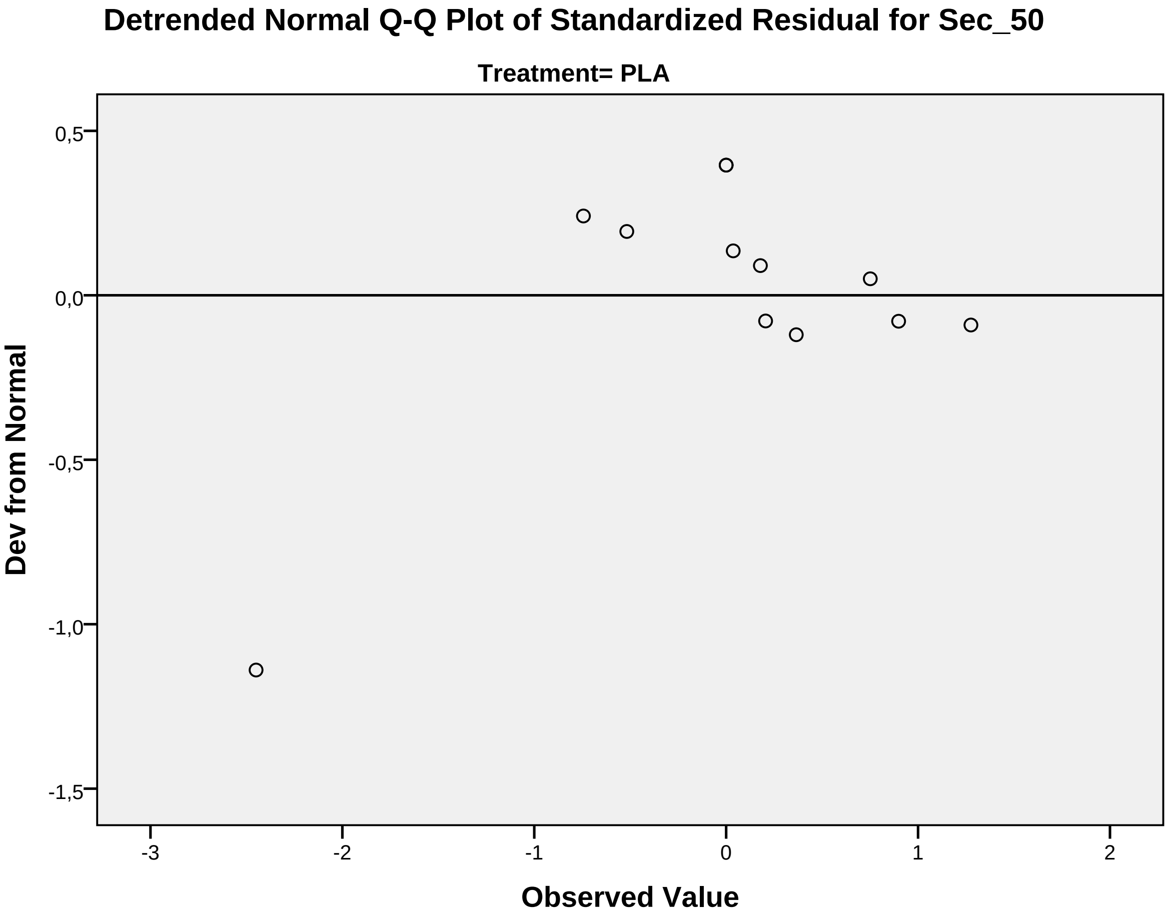
Detrended Normal Q-Q Plot of Standardized Residual for Sec_50
Treatment= PLA
0,5
0,0
-0,5
-1,0
-1,5
Dev from Normal
-3
-2
-1
0
1
2
Observed Value

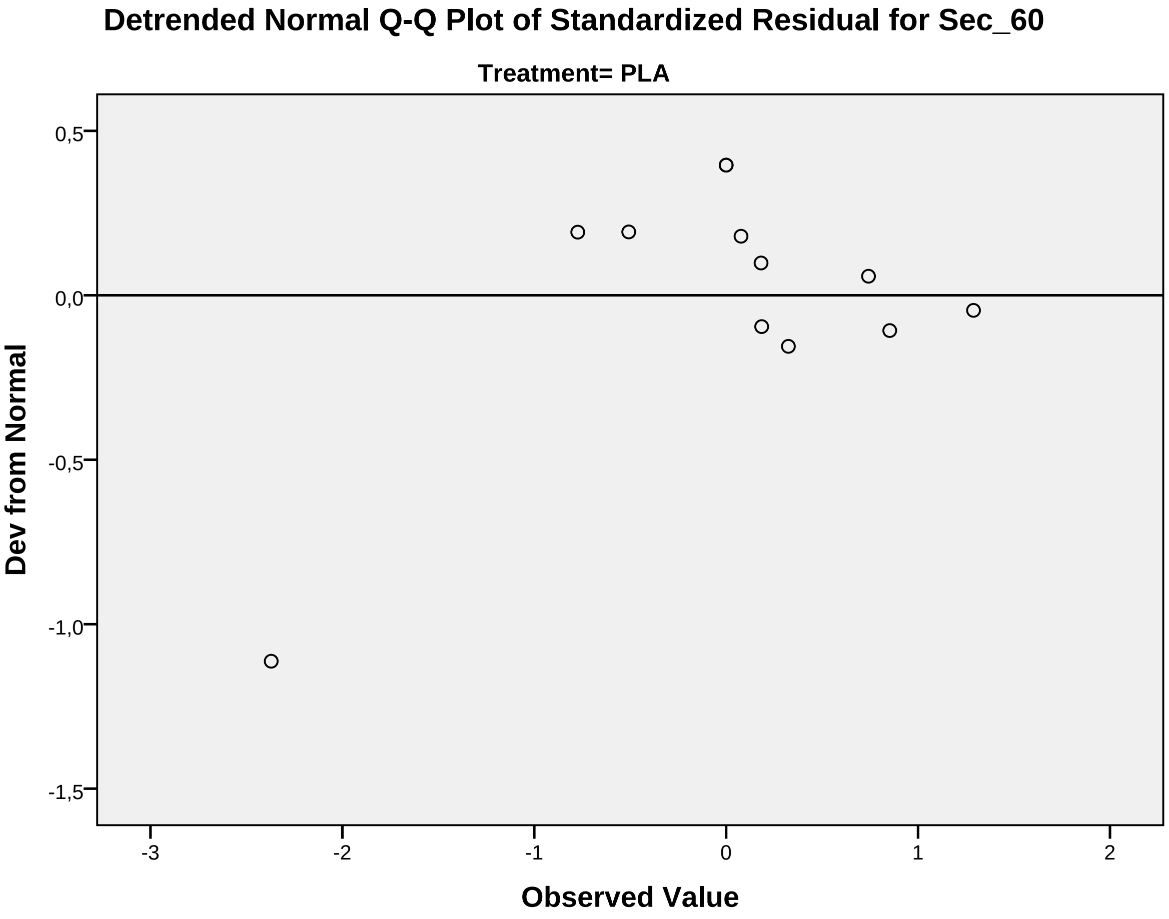
Detrended Normal Q-Q Plot of Standardized Residual for Sec_60
Treatment= PLA
Dev from Normal
0,5
0,0
-0,5
-1,0
-1,5
-3
-2
-1
0
1
2
Observed Value

Treatment: PLA

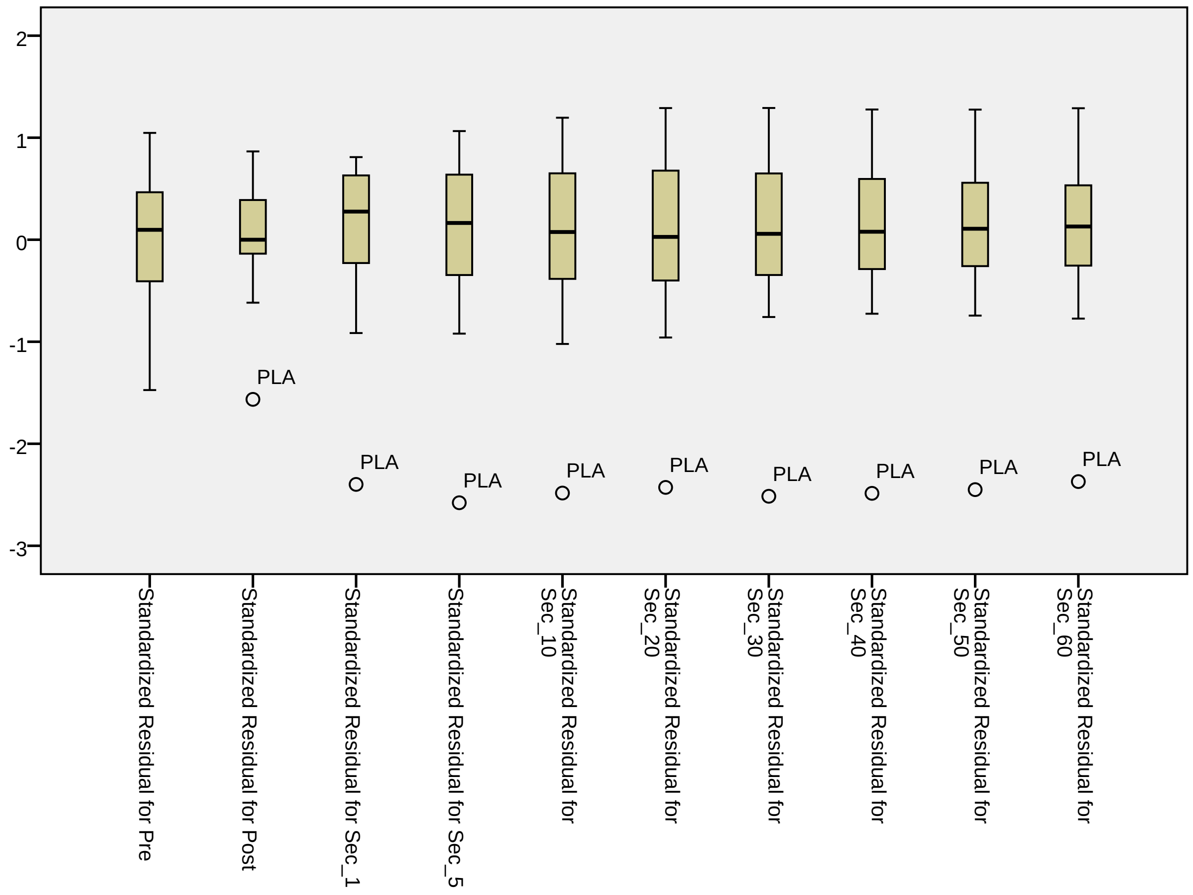

Normal Q-Q Plots of Standardized Residual for SmO2 During Recovery

Normal Q-Q Plot of Standardized Residual for Pre

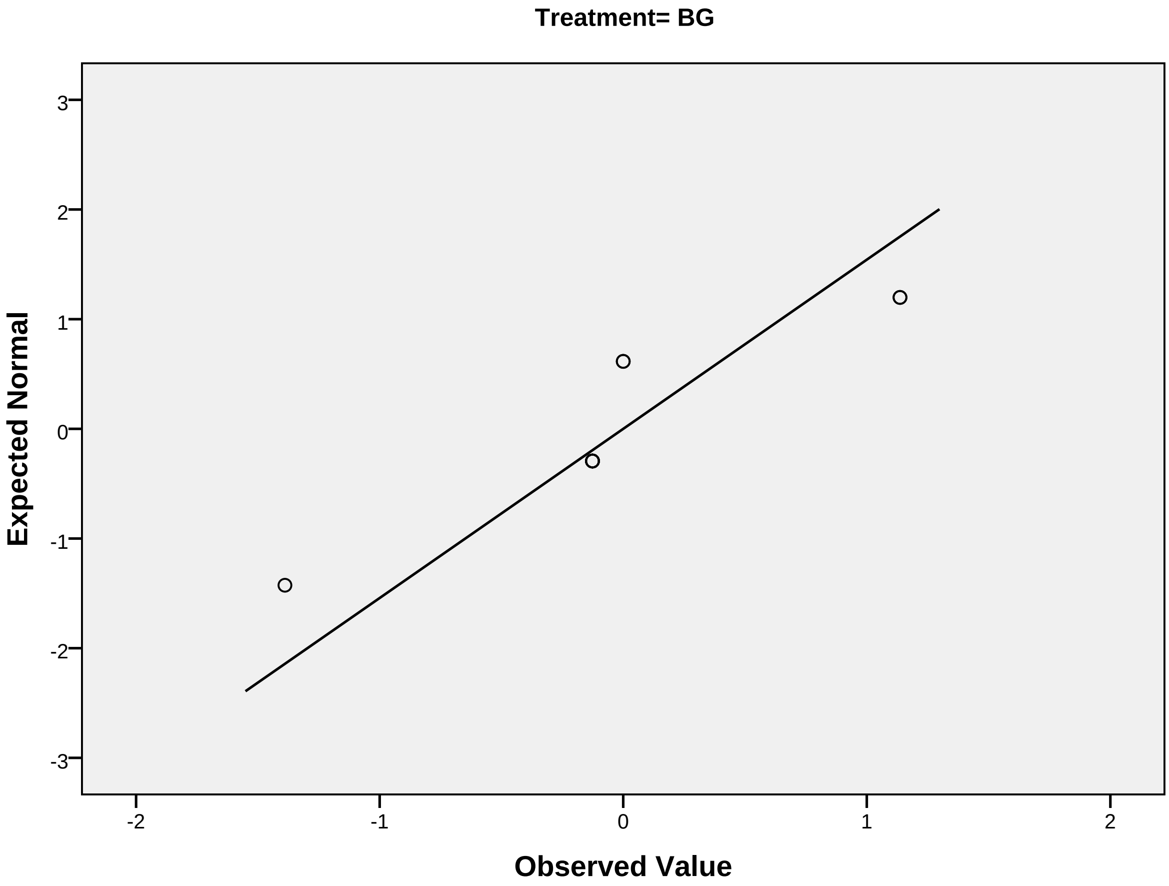

Normal Q-Q Plot of Standardized Residual for Sec_1

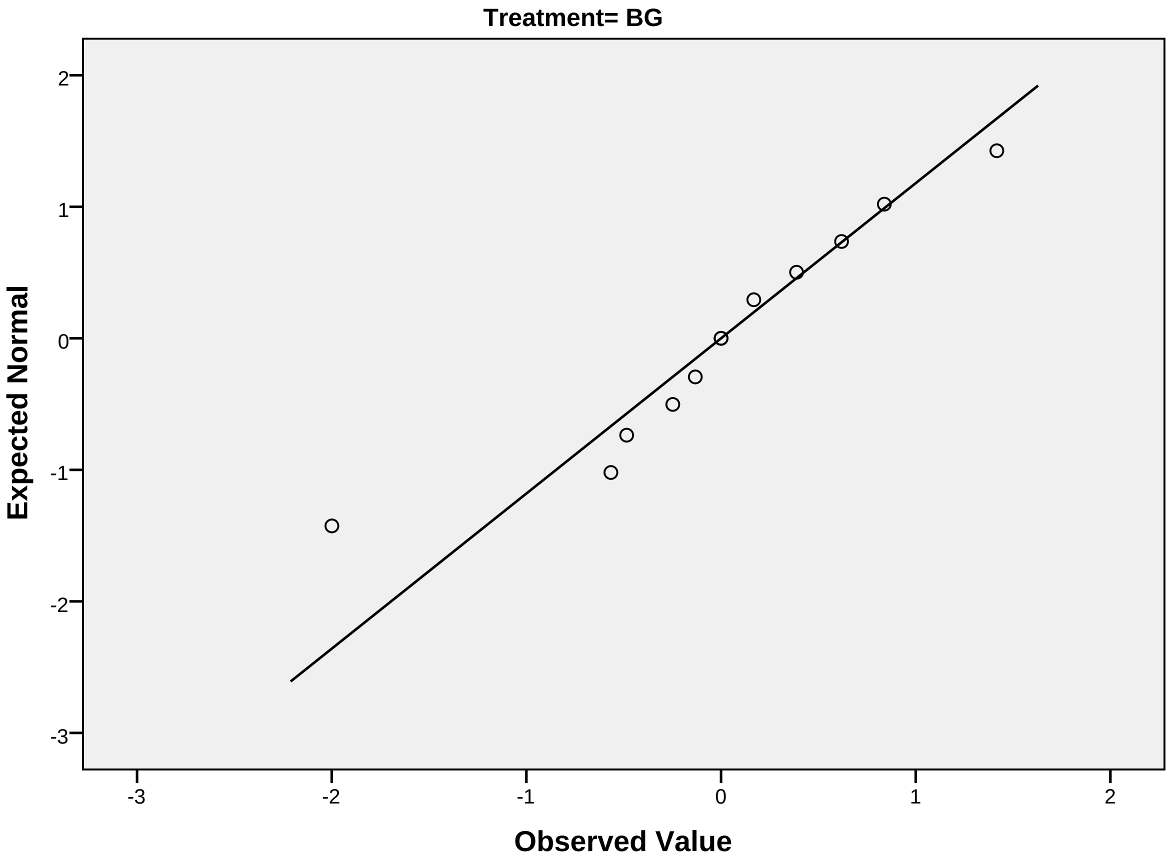

Normal Q-Q Plot of Standardized Residual for Sec_5

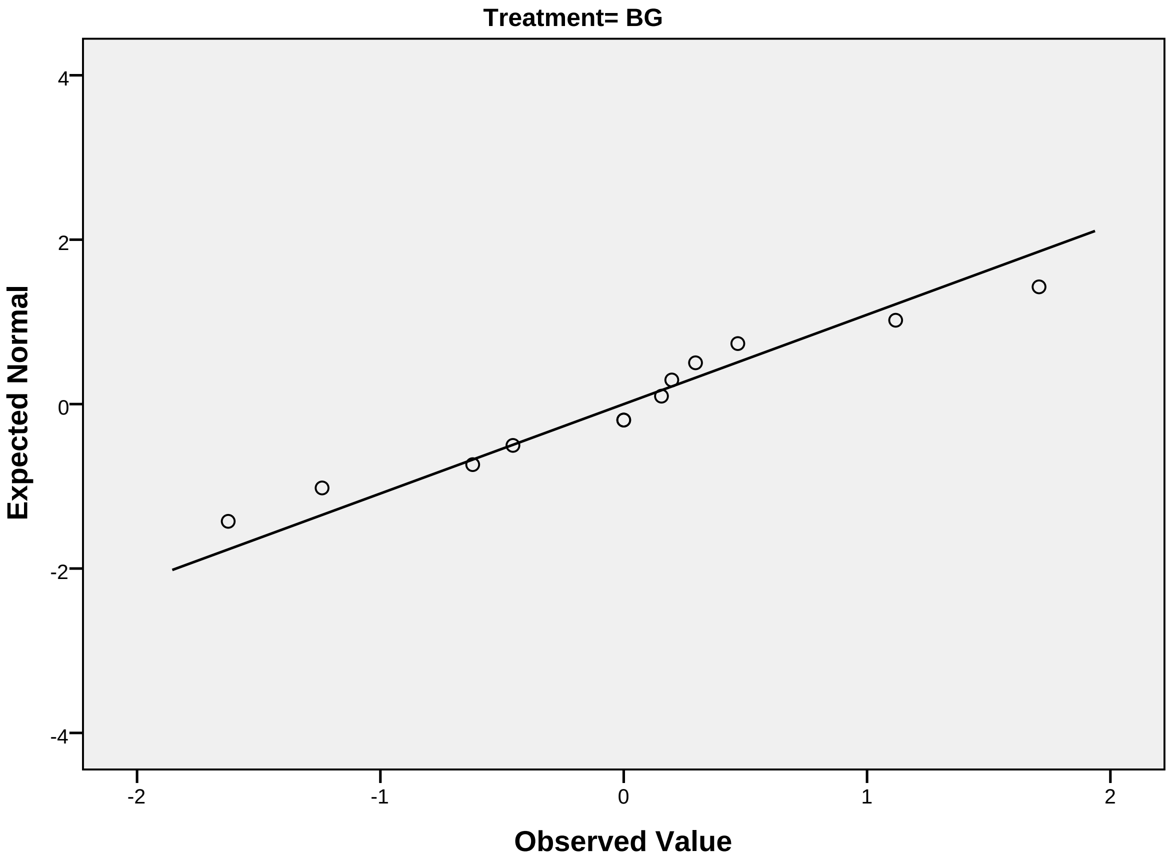

Normal Q-Q Plot of Standardized Residual for Sec_10

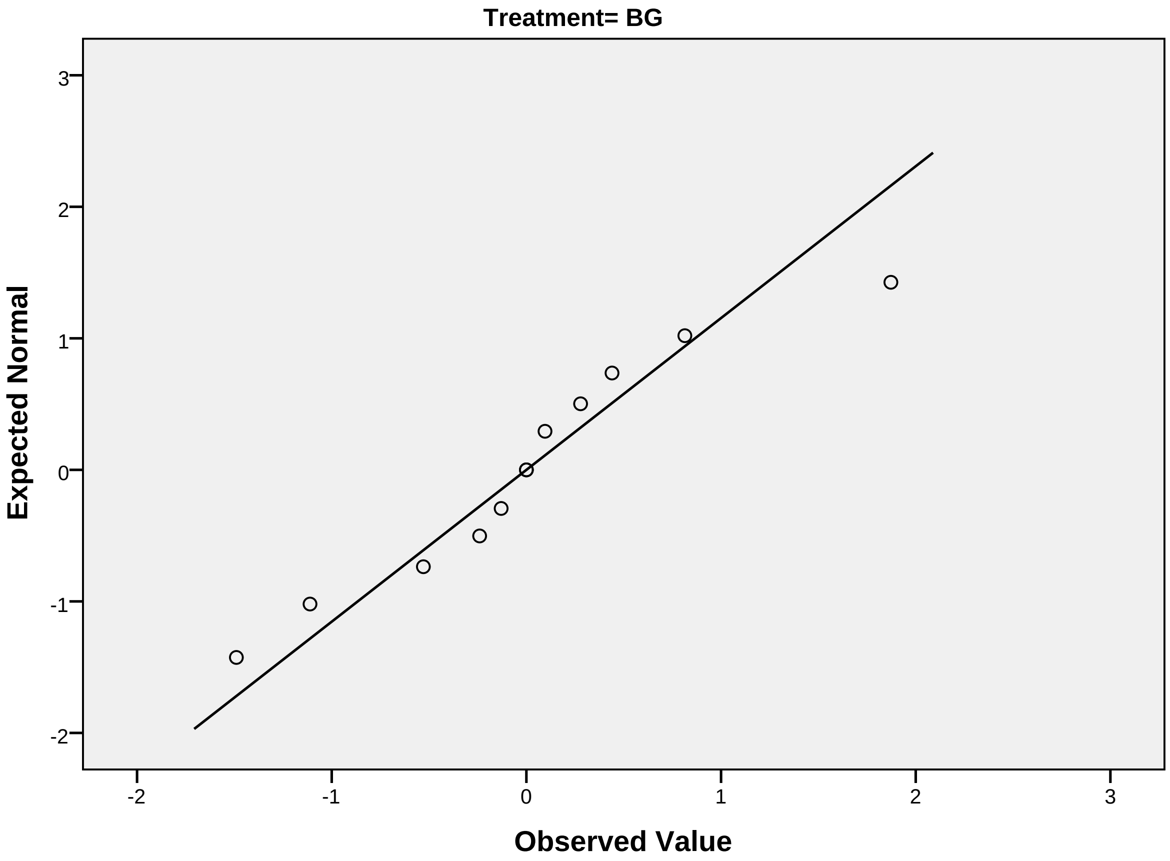

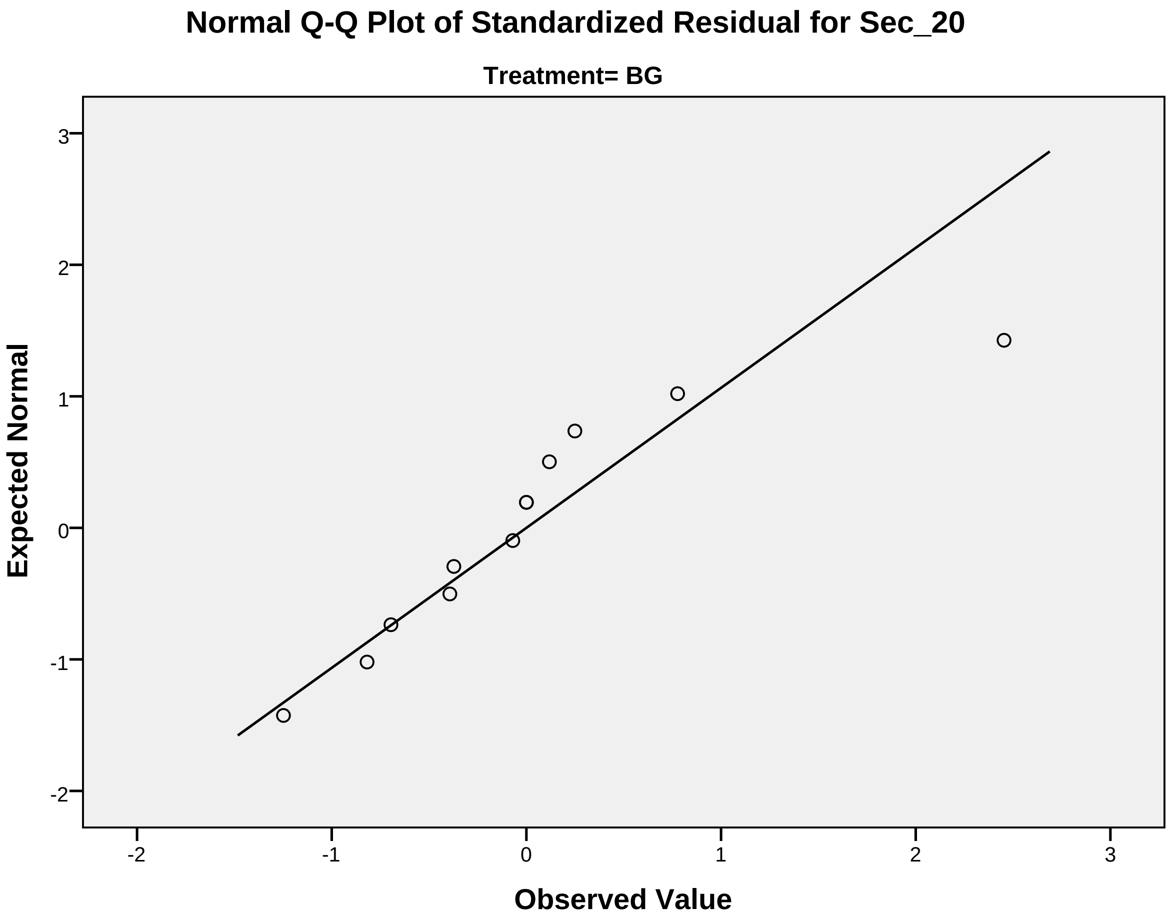

Normal Q-Q Plot of Standardized Residual for Sec_20
Treatment= BG
Expected Normal
3
2
1
0
-1
-2
-2
-1
0
1
2
3
Observed Value

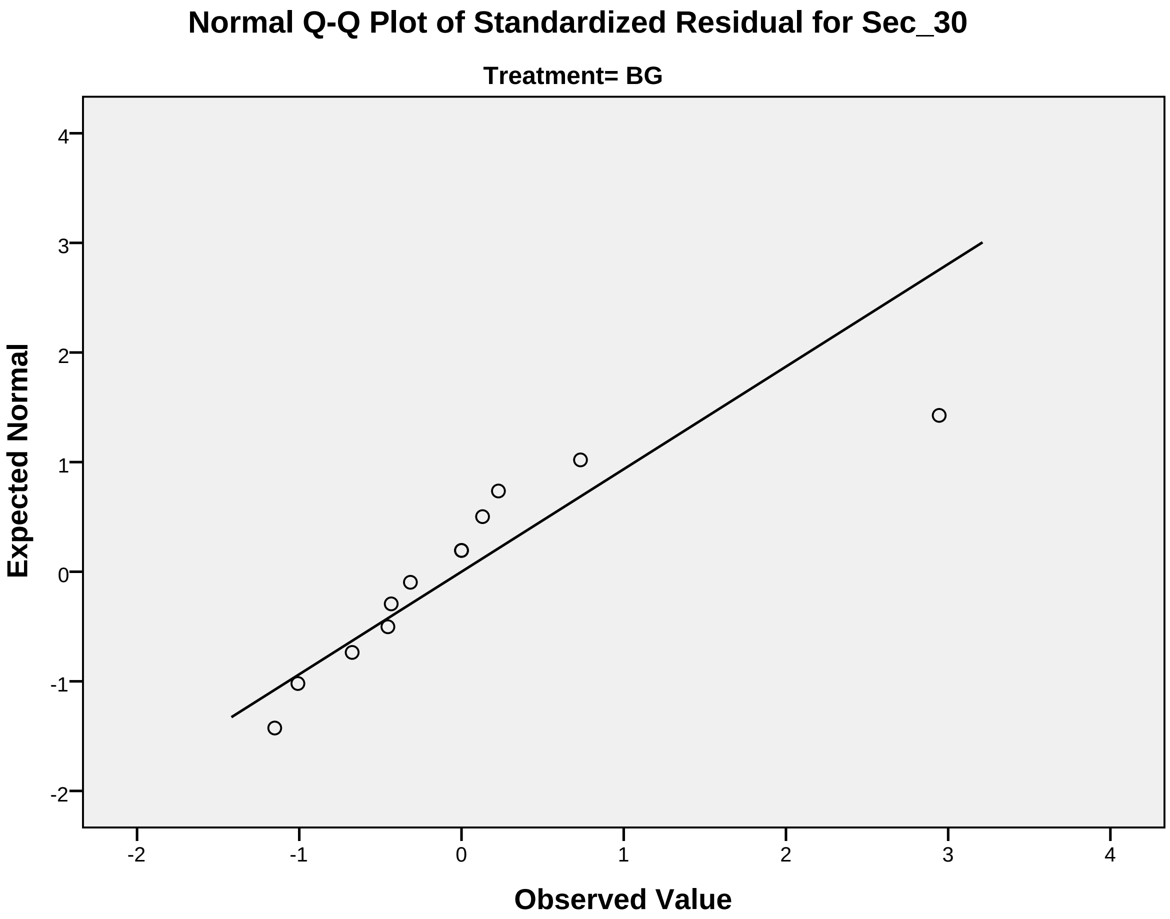
Normal Q-Q Plot of Standardized Residual for Sec_30
Treatment= BG
Expected Normal
Observed Value
4
3
2
1
0
-1
-2
-2
-1
0
1
2
3
4

Normal Q-Q Plot of Standardized Residual for Sec_40

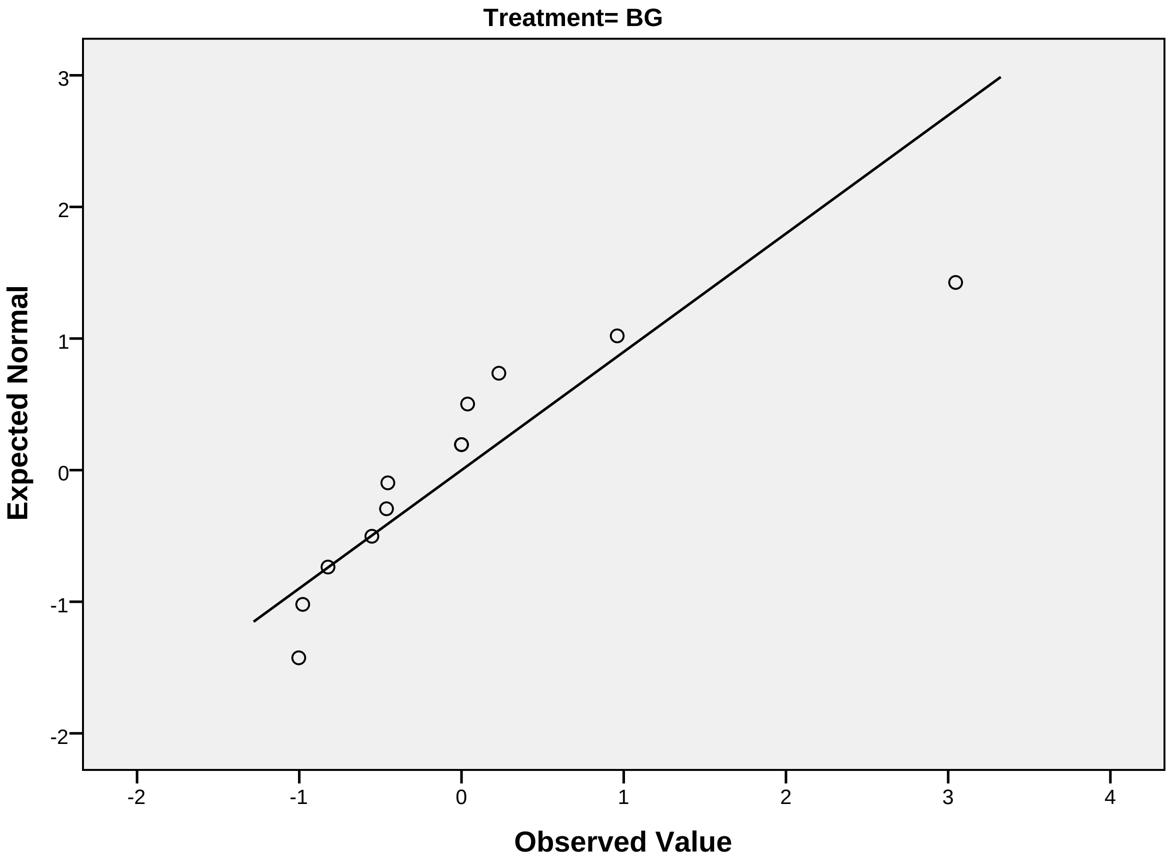

Normal Q-Q Plot of Standardized Residual for Sec_50

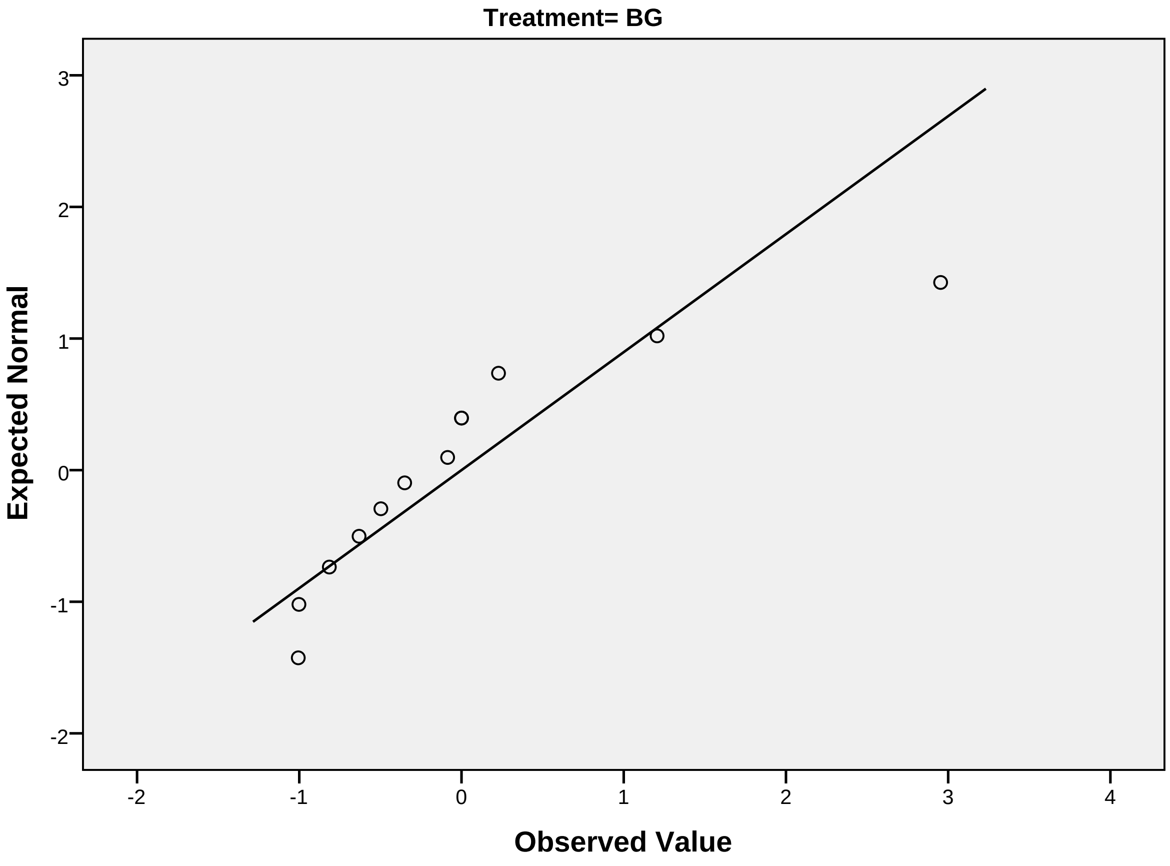

Normal Q-Q Plot of Standardized Residual for Sec_60

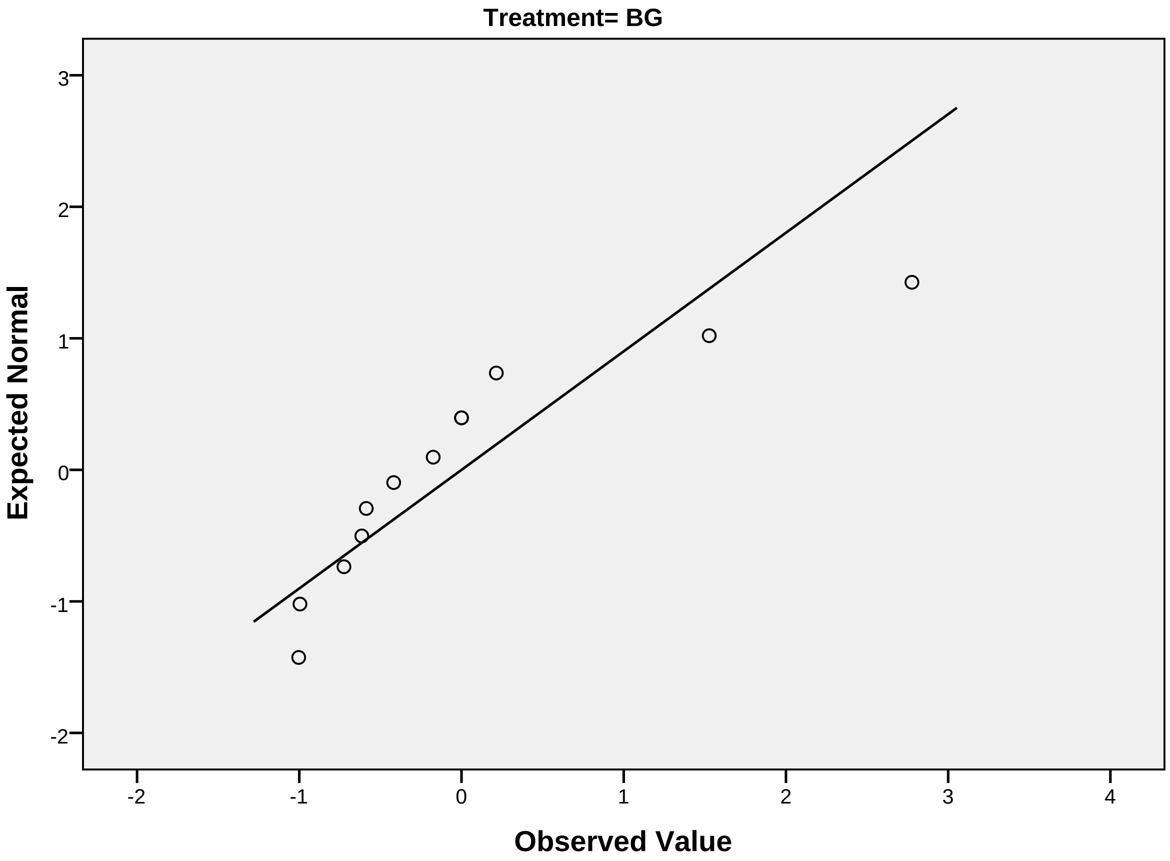

Detrended Normal Q-Q Plots of Standardized residual for tHb During Exercise

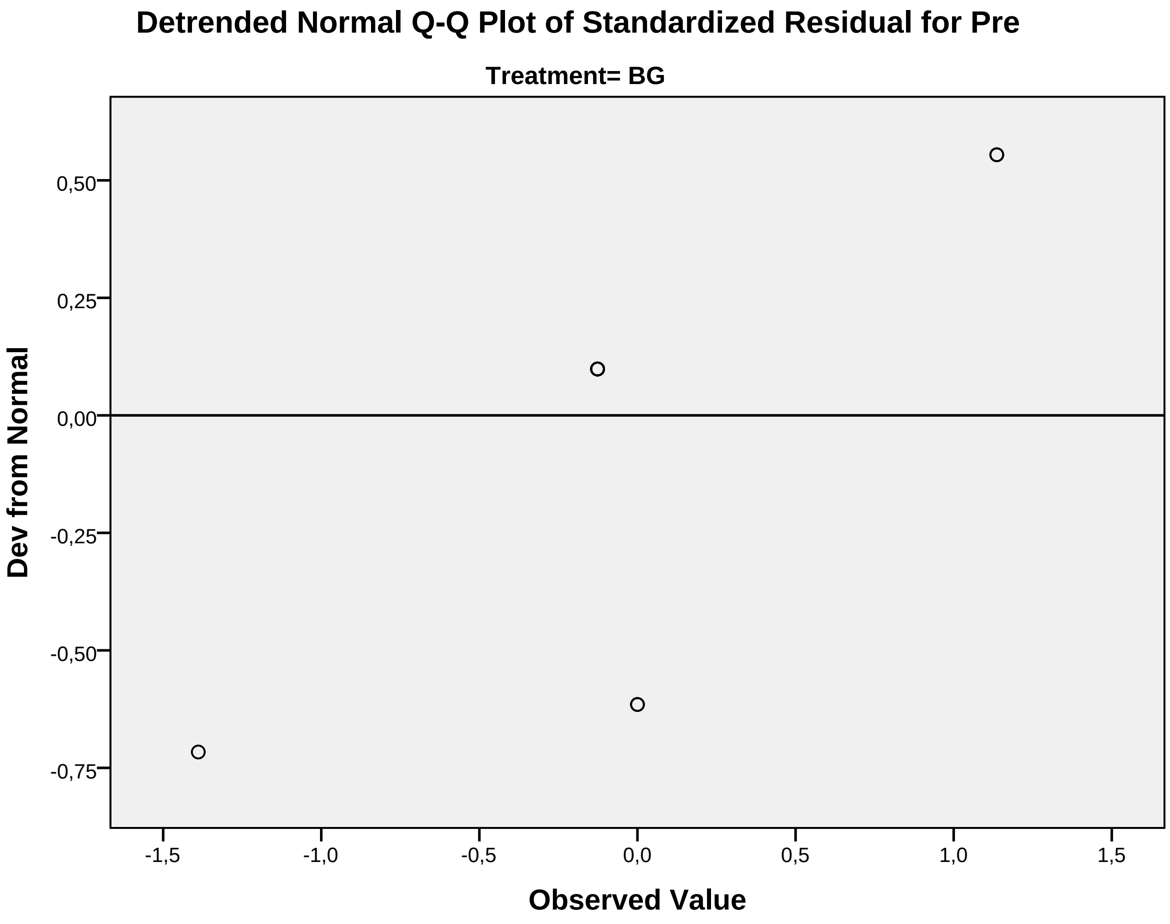

Detrended Normal Q-Q Plot of Standardized Residual for Pre
Treatment= BG
Dev from Normal
0,50
0,25
0,00
-0,25
-0,50
-0,75
-1,5
-1,0
-0,5
0,0
0,5
1,0
1,5
Observed Value

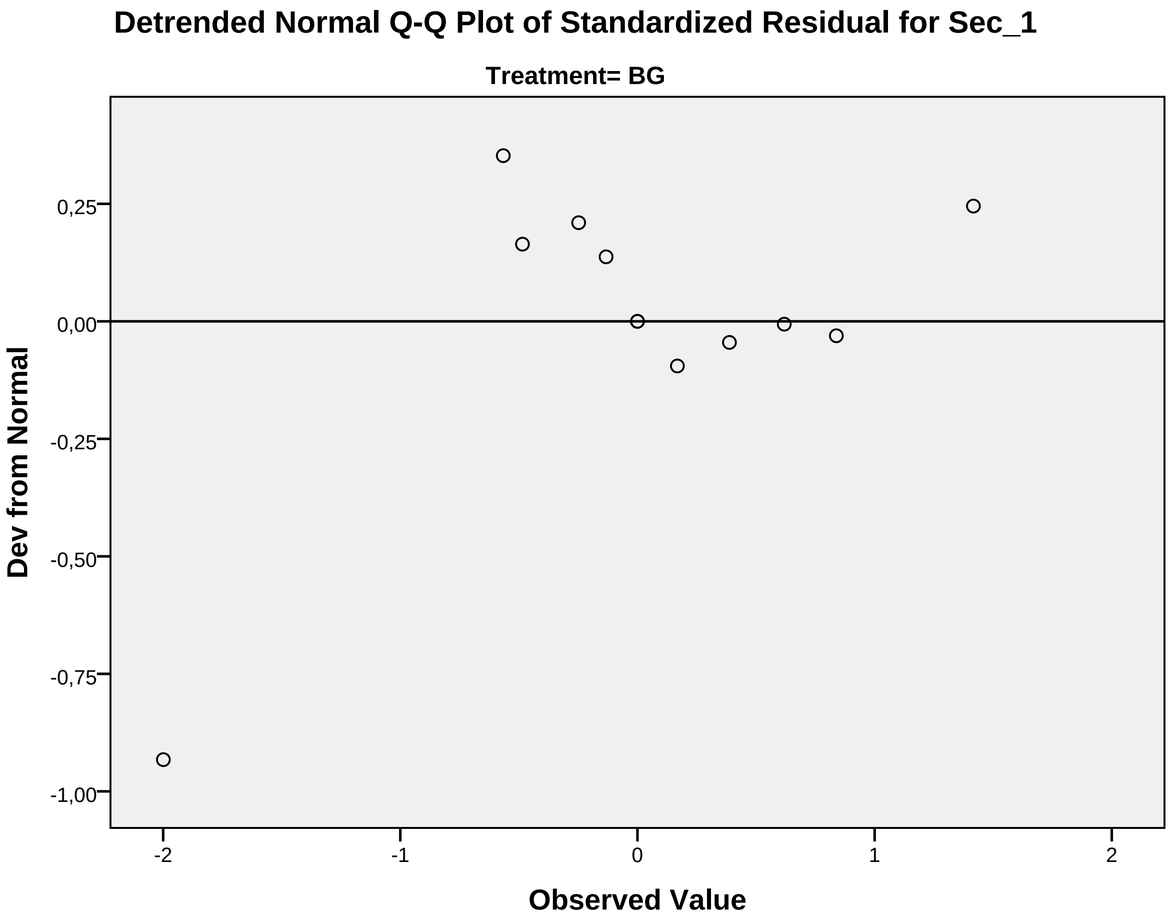
Detrended Normal Q-Q Plot of Standardized Residual for Sec_1
Treatment= BG
Dev from Normal
0,25
0,00
-0,25
-0,50
-0,75
-1,00
-2
-1
0
1
2
Observed Value

Detrended Normal Q-Q Plot of Standardized Residual for Sec_5

Treatment= BG

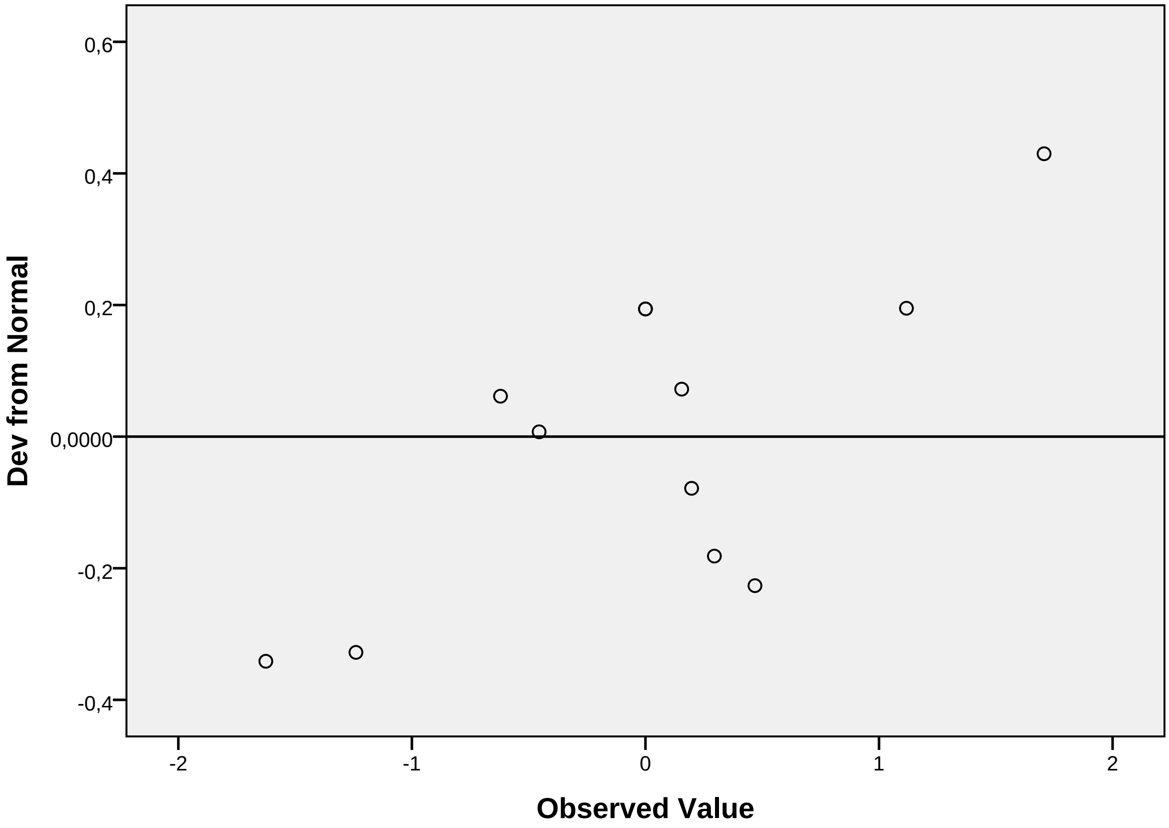

Detrended Normal Q-Q Plot of Standardized Residual for Sec_10

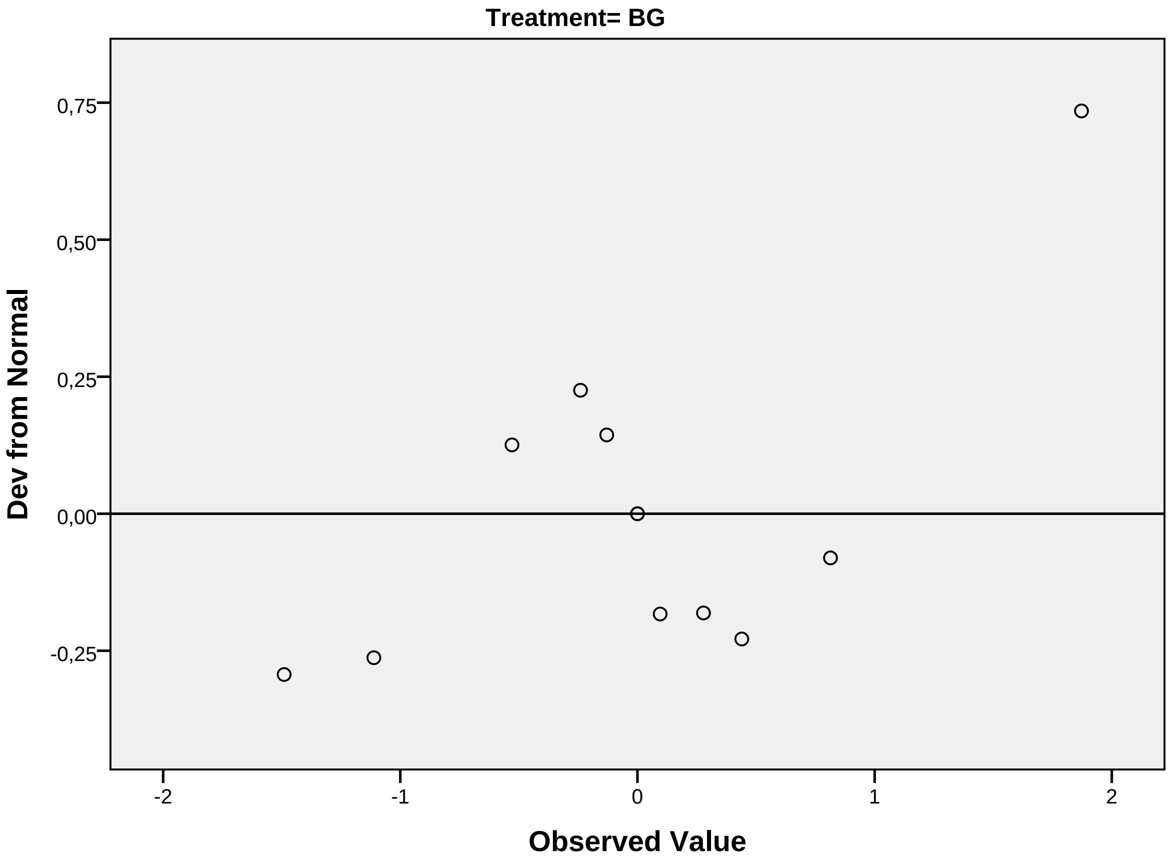

Detrended Normal Q-Q Plot of Standardized Residual for Sec_20

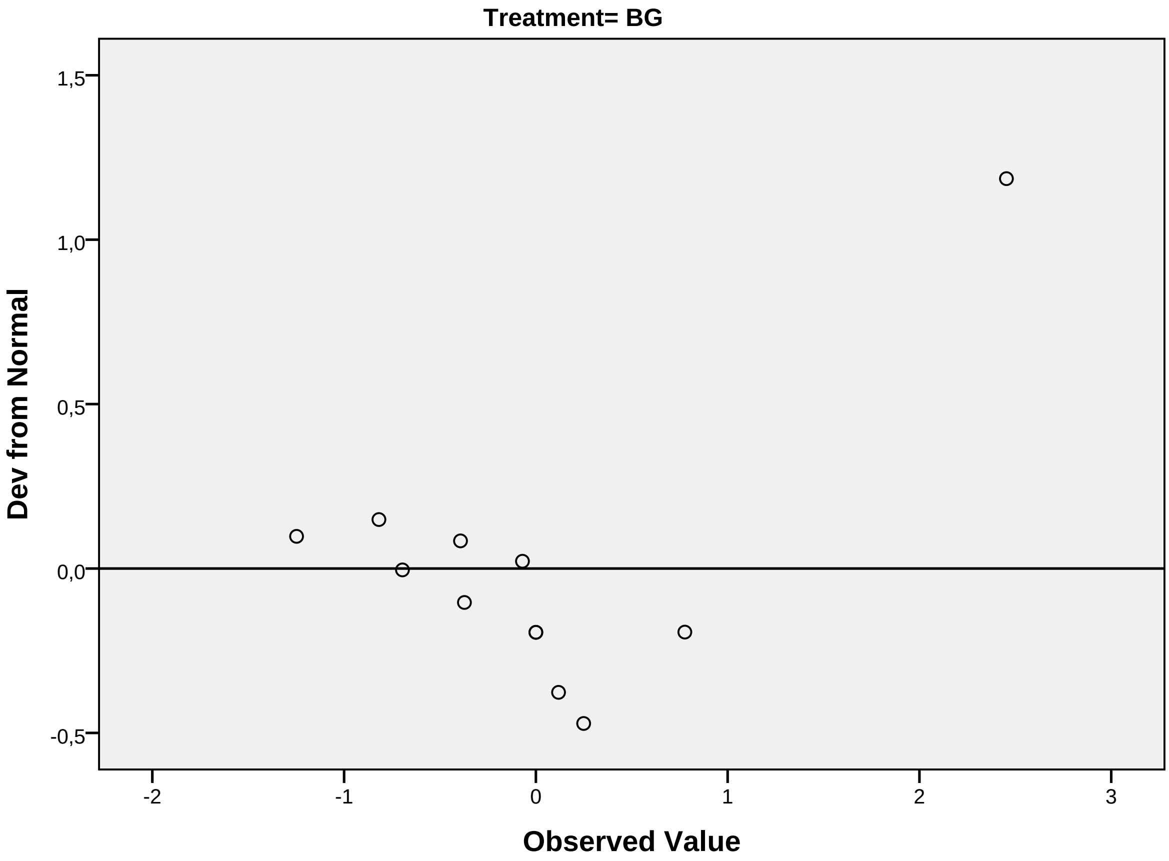

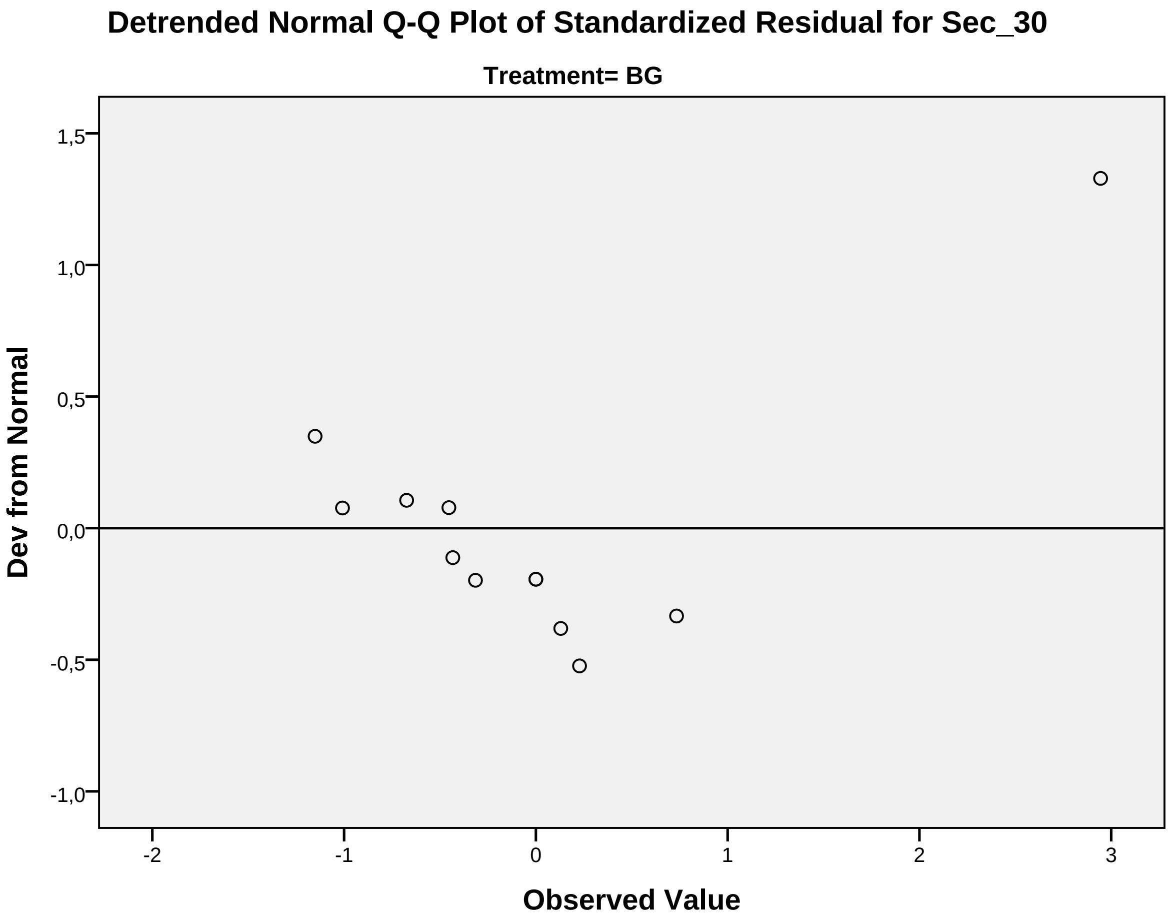

Detrended Normal Q-Q Plot of Standardized Residual for Sec_30
Treatment= BG
Dev from Normal
1,5
1,0
0,5
0,0
-0,5
-1,0
-2
-1
0
1
2
3
Observed Value

Detrended Normal Q-Q Plot of Standardized Residual for Sec_40

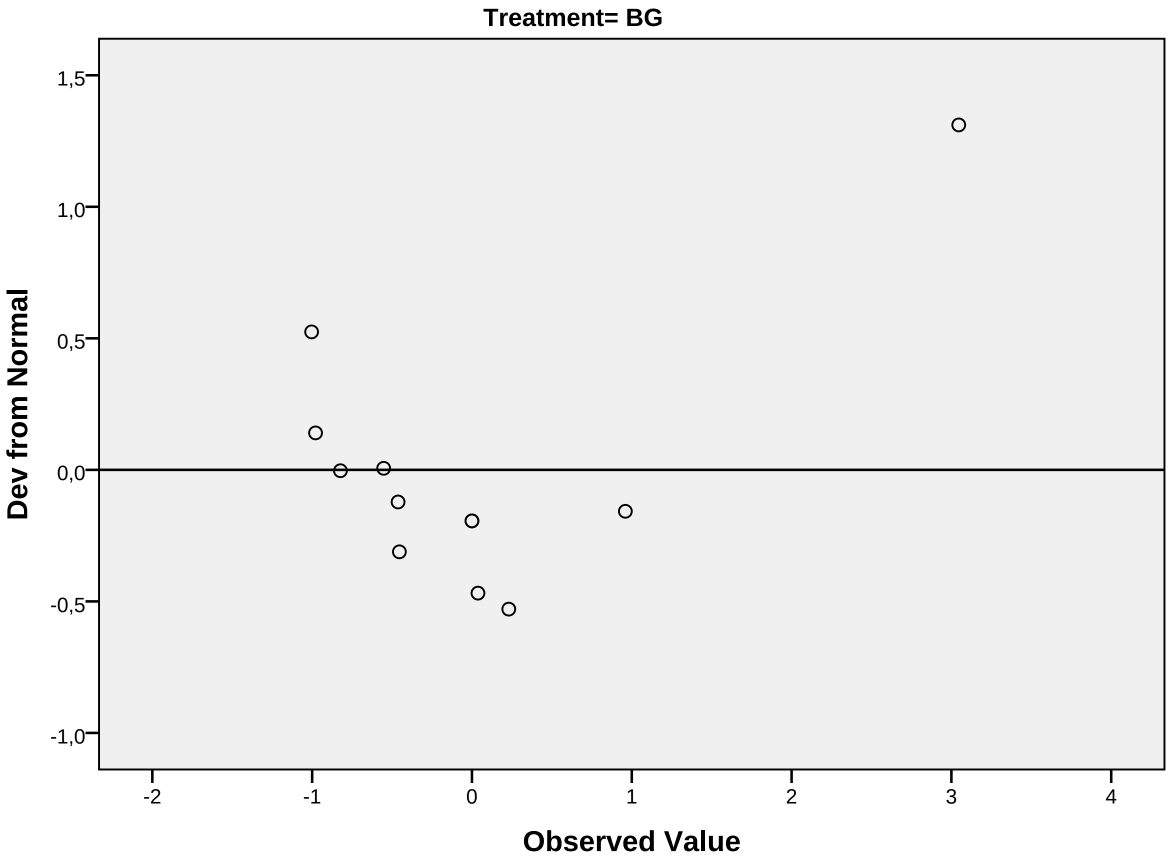

Detrended Normal Q-Q Plot of Standardized Residual for Sec_50

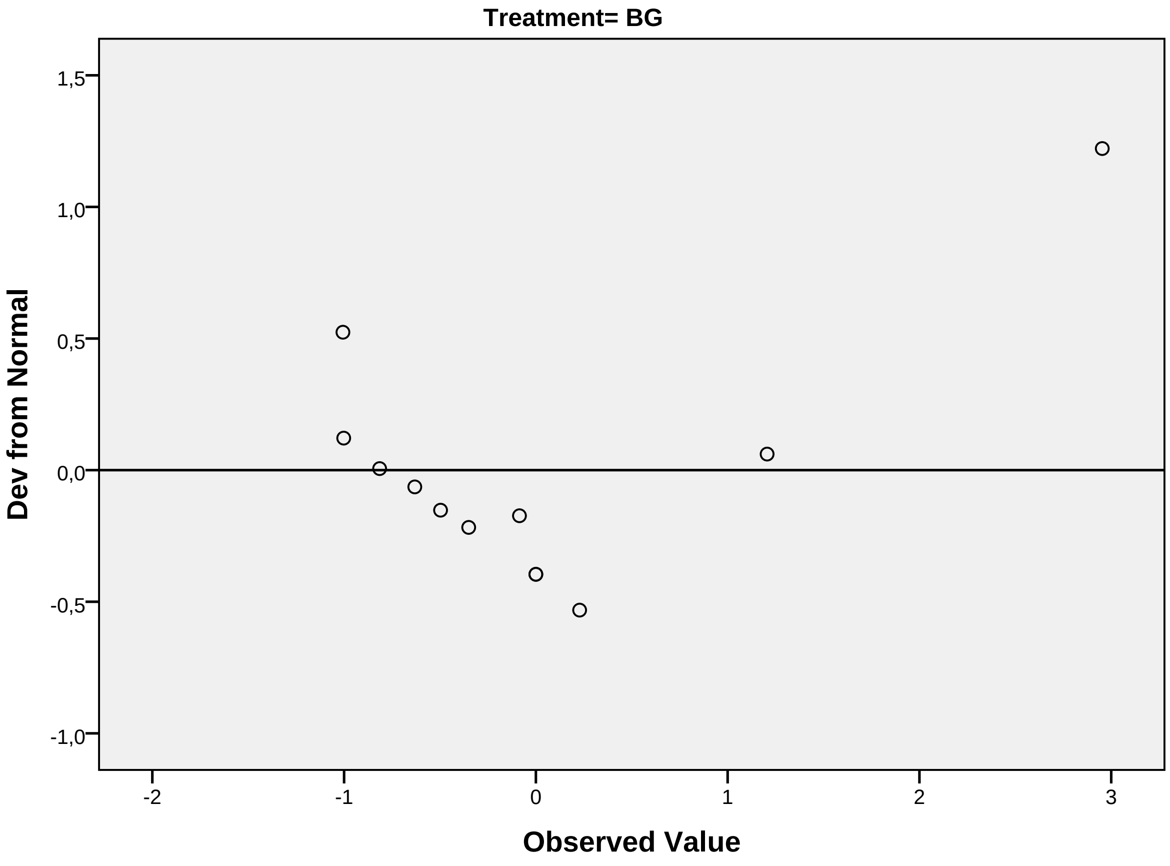

Detrended Normal Q-Q Plot of Standardized Residual for Sec_60

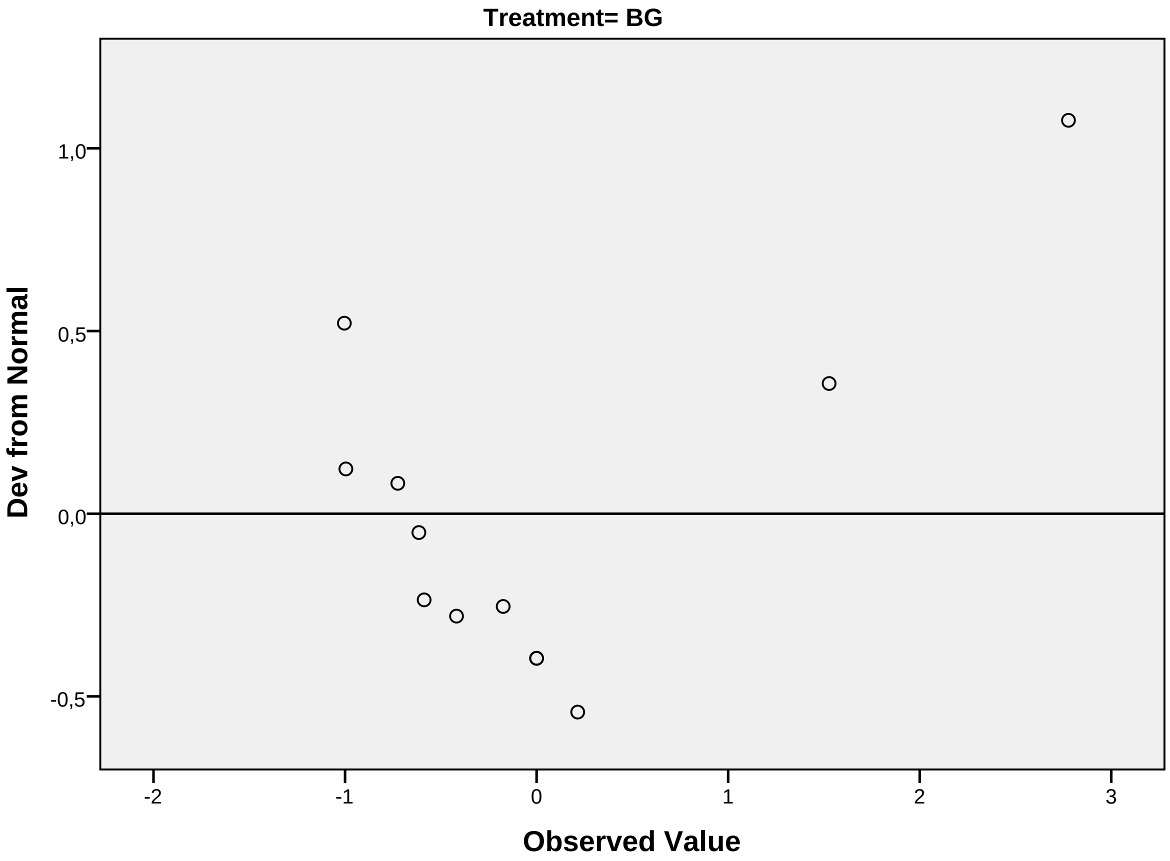

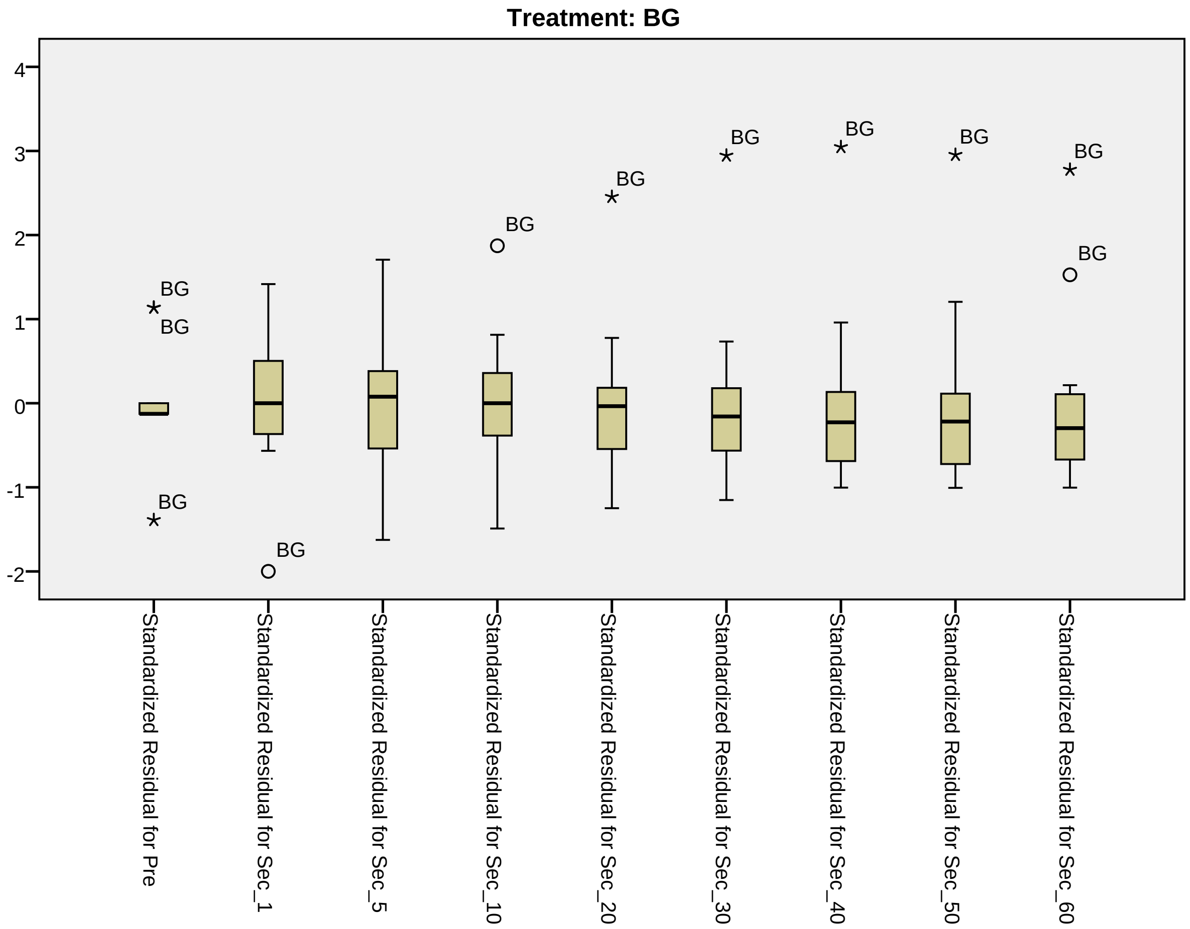

Normal Q-Q Plots of Standardized residual for tHb During Exercise

Normal Q-Q Plots of Standardized Residual for SmO2 During Recovery

Normal Q-Q Plot of Standardized Residual for Pre

Treatment= PLA

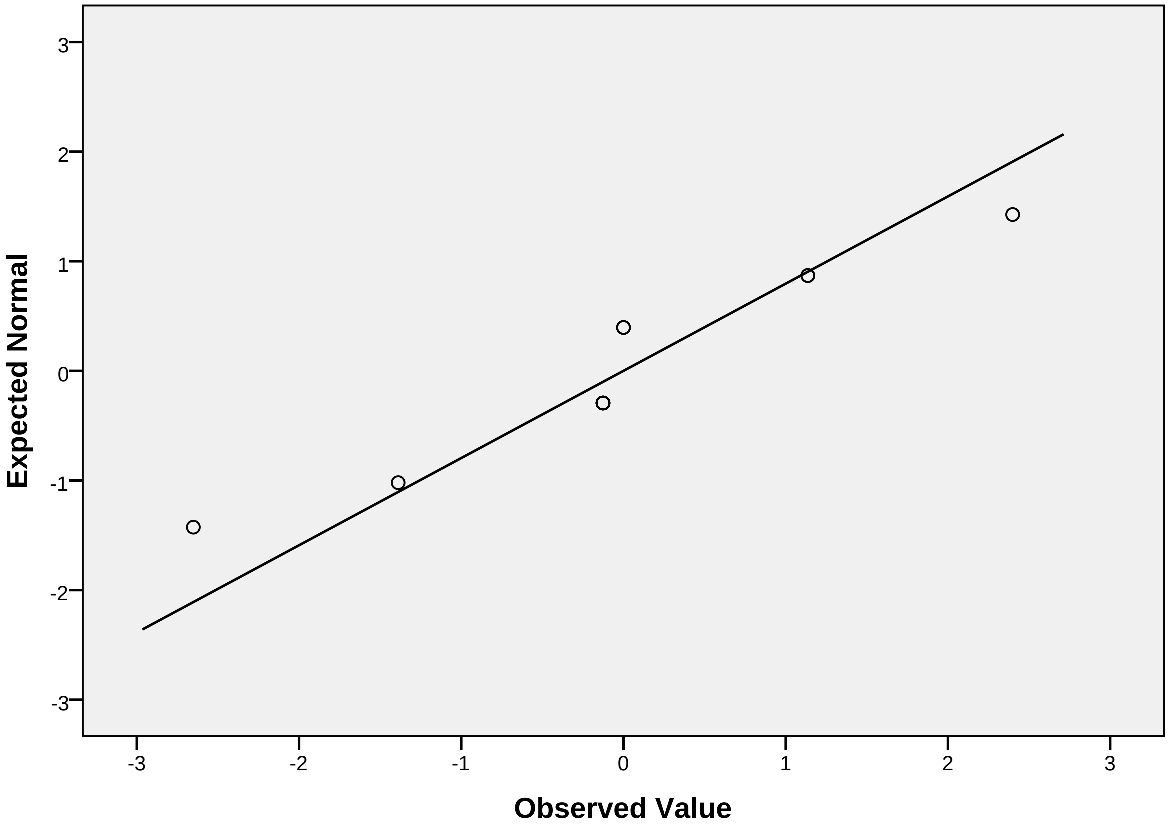

Normal Q-Q Plot of Standardized Residual for Sec_1

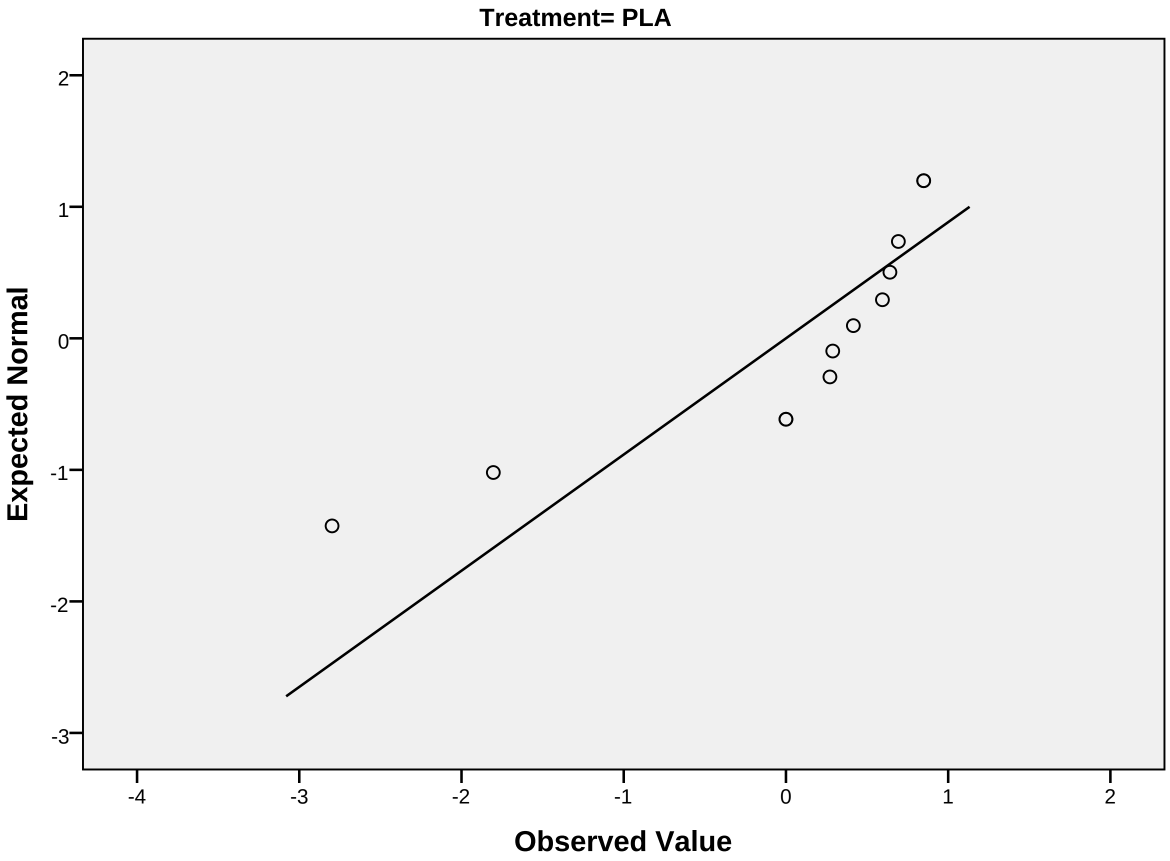

Normal Q-Q Plot of Standardized Residual for Sec_5

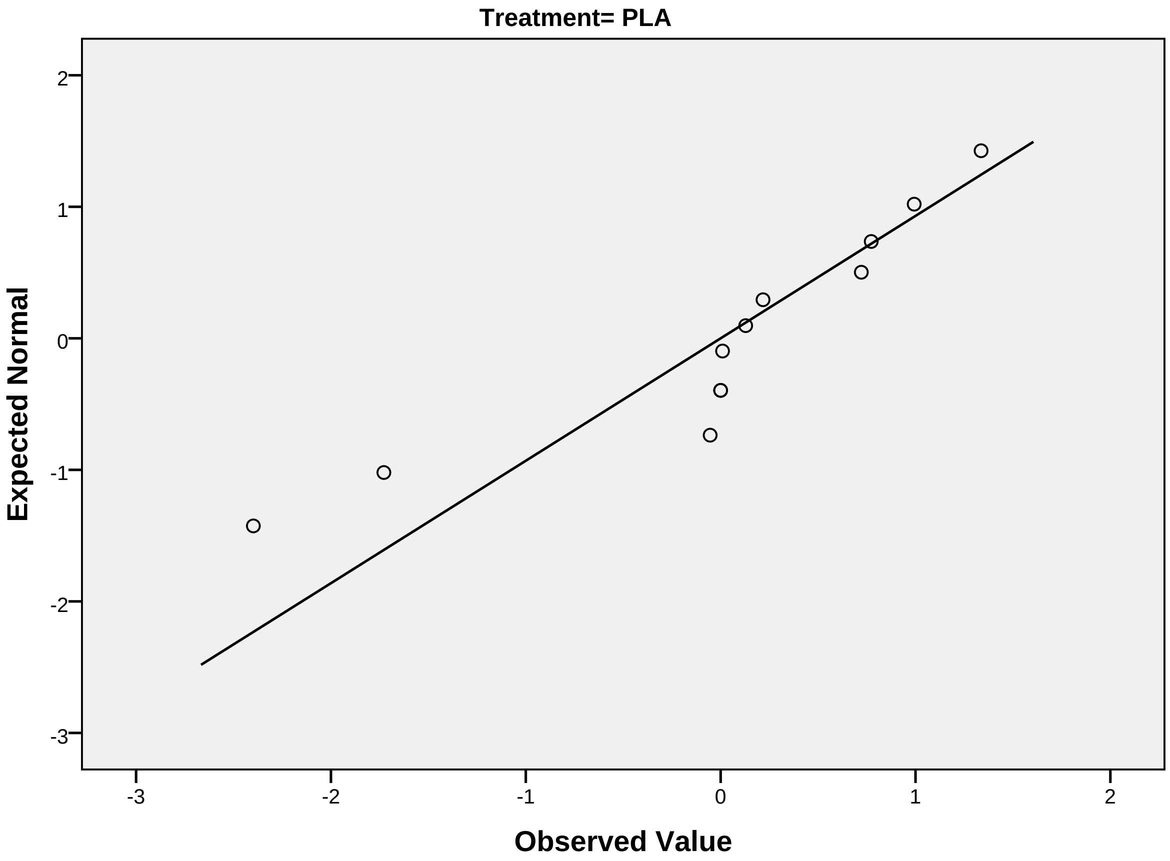

Normal Q-Q Plot of Standardized Residual for Sec_10

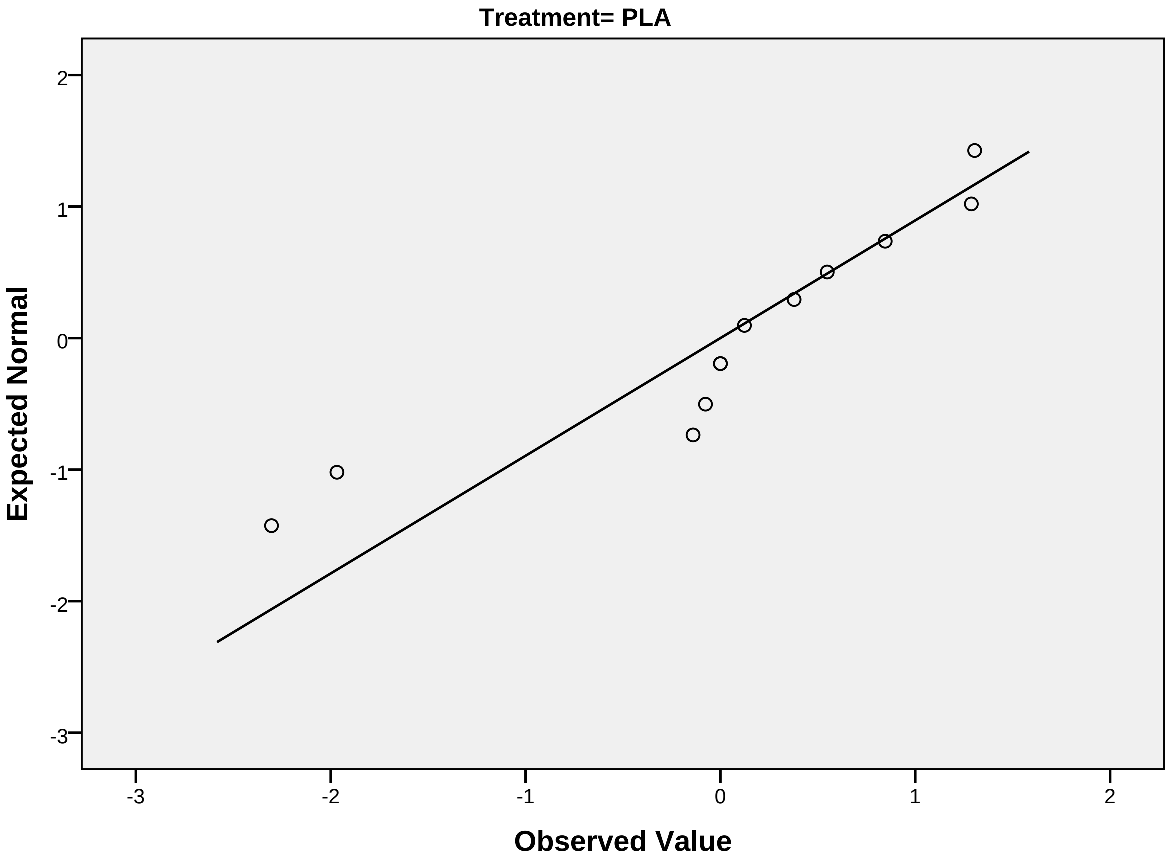

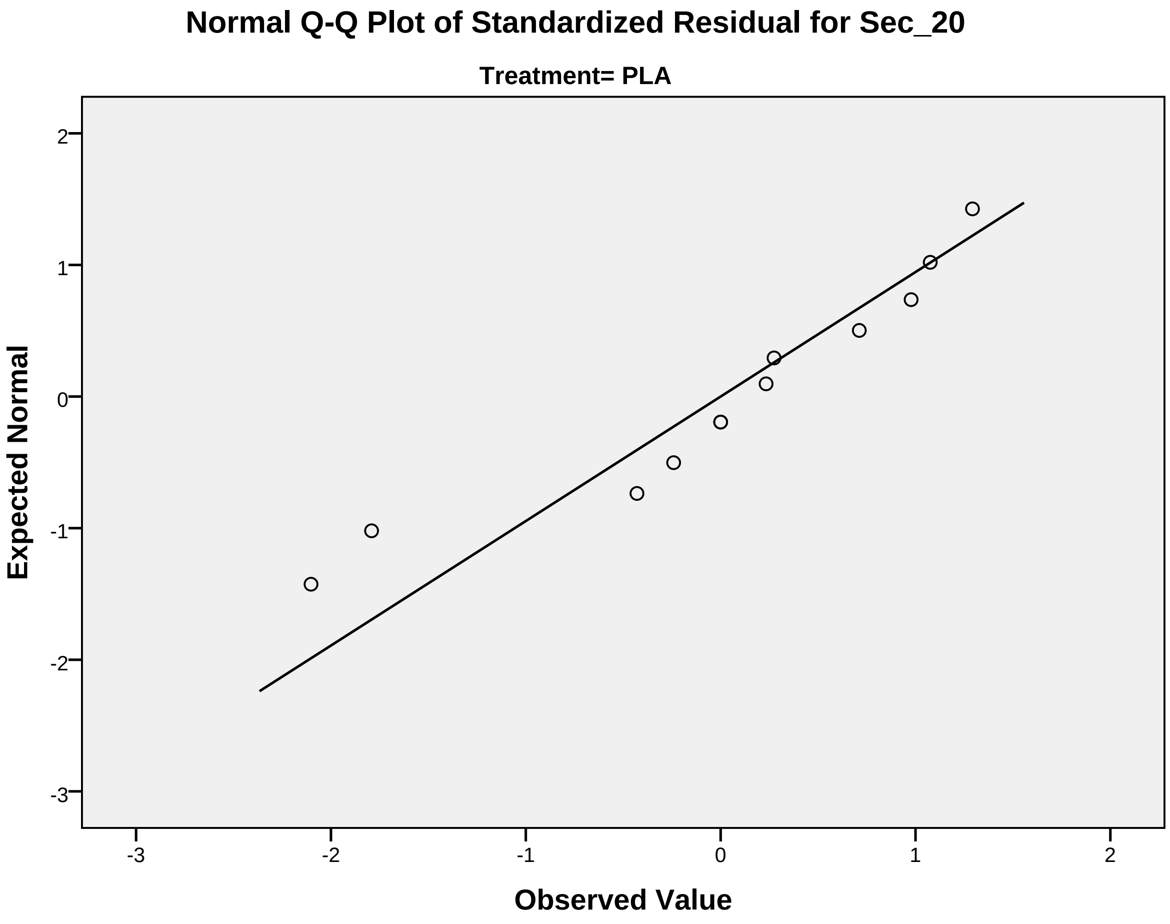
Normal Q-Q Plot of Standardized Residual for Sec_20
Treatment= PLA
Expected Normal
2
1
0
-1
-2
-3
-3
-2
-1
0
1
2
Observed Value

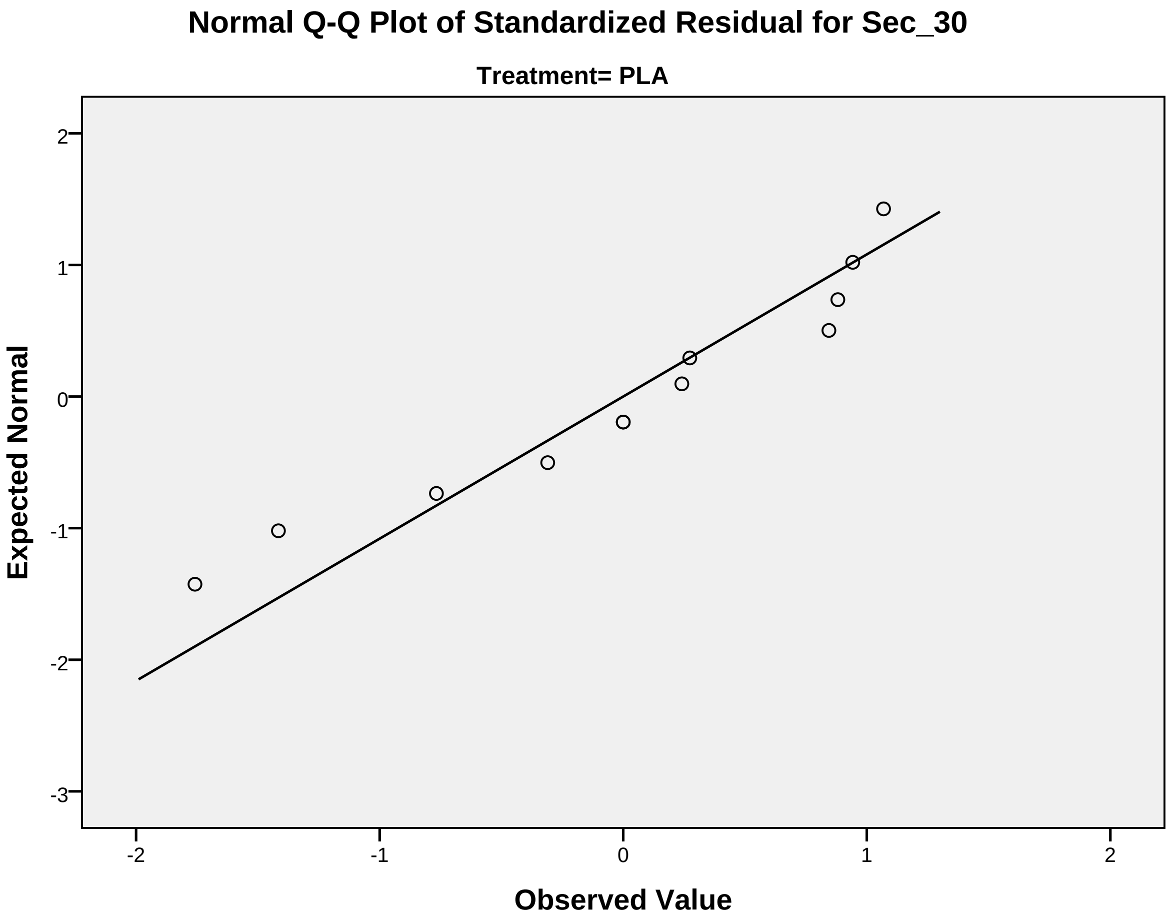
Normal Q-Q Plot of Standardized Residual for Sec_30
Treatment= PLA
Expected Normal
2
1
0
-1
-2
-3
-2
-1
0
1
2
Observed Value

Normal Q-Q Plot of Standardized Residual for Sec_40

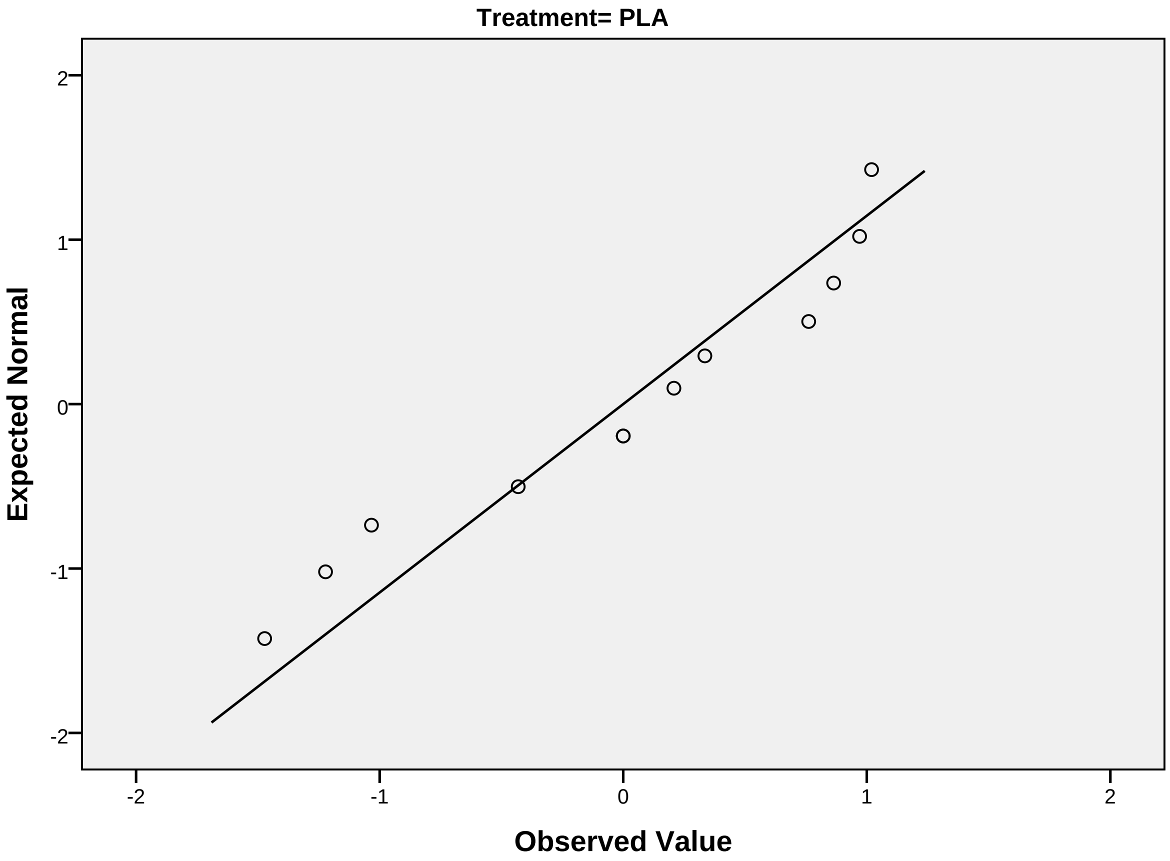

Normal Q-Q Plot of Standardized Residual for Sec_50

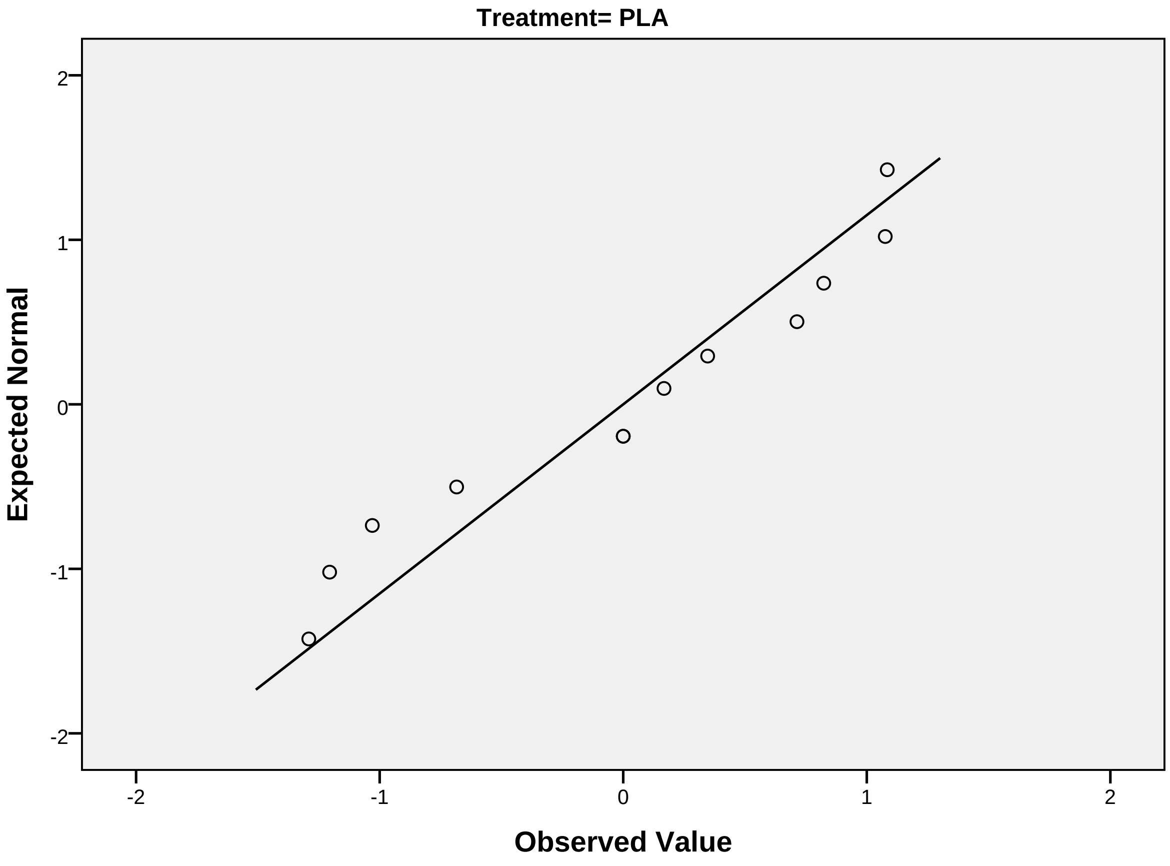

Normal Q-Q Plot of Standardized Residual for Sec_60

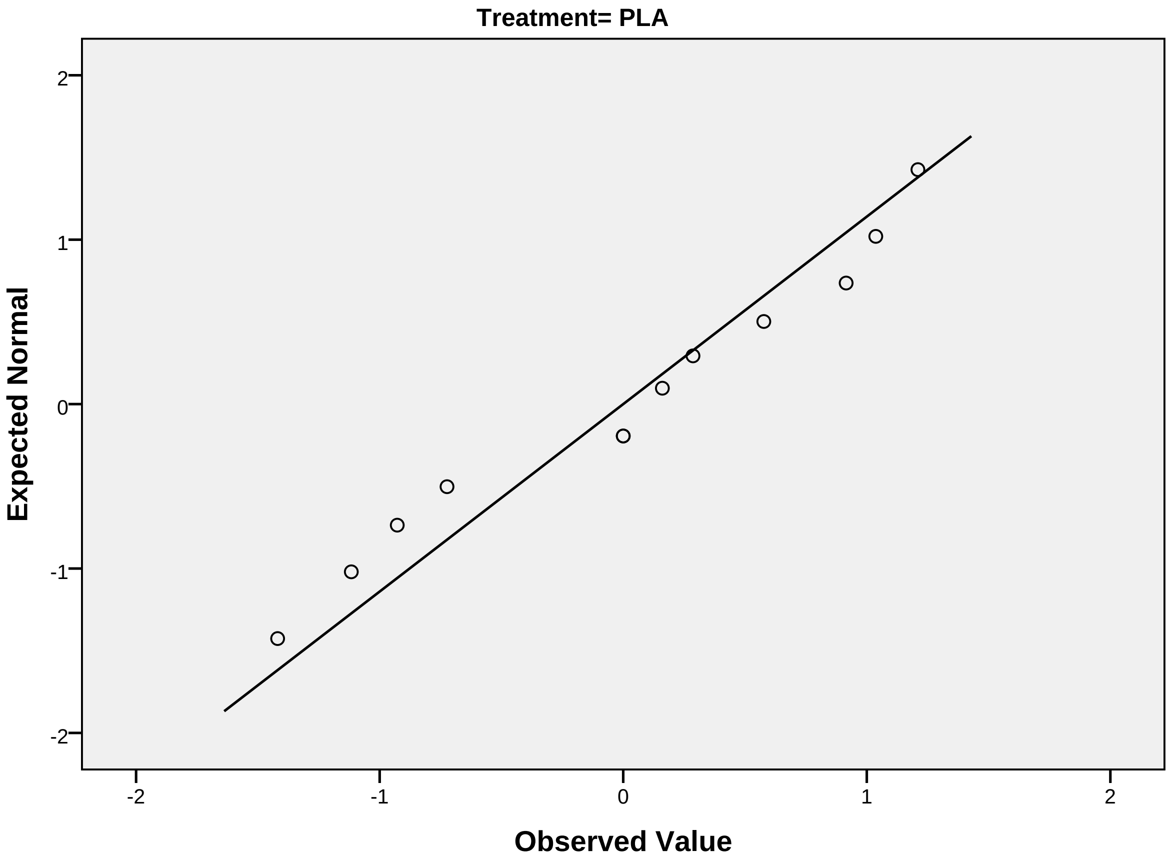

Detrended Normal Q-Q Plots of Standardized residual for tHb During Exercise

Detrended Normal Q-Q Plot of Standardized Residual for Pre

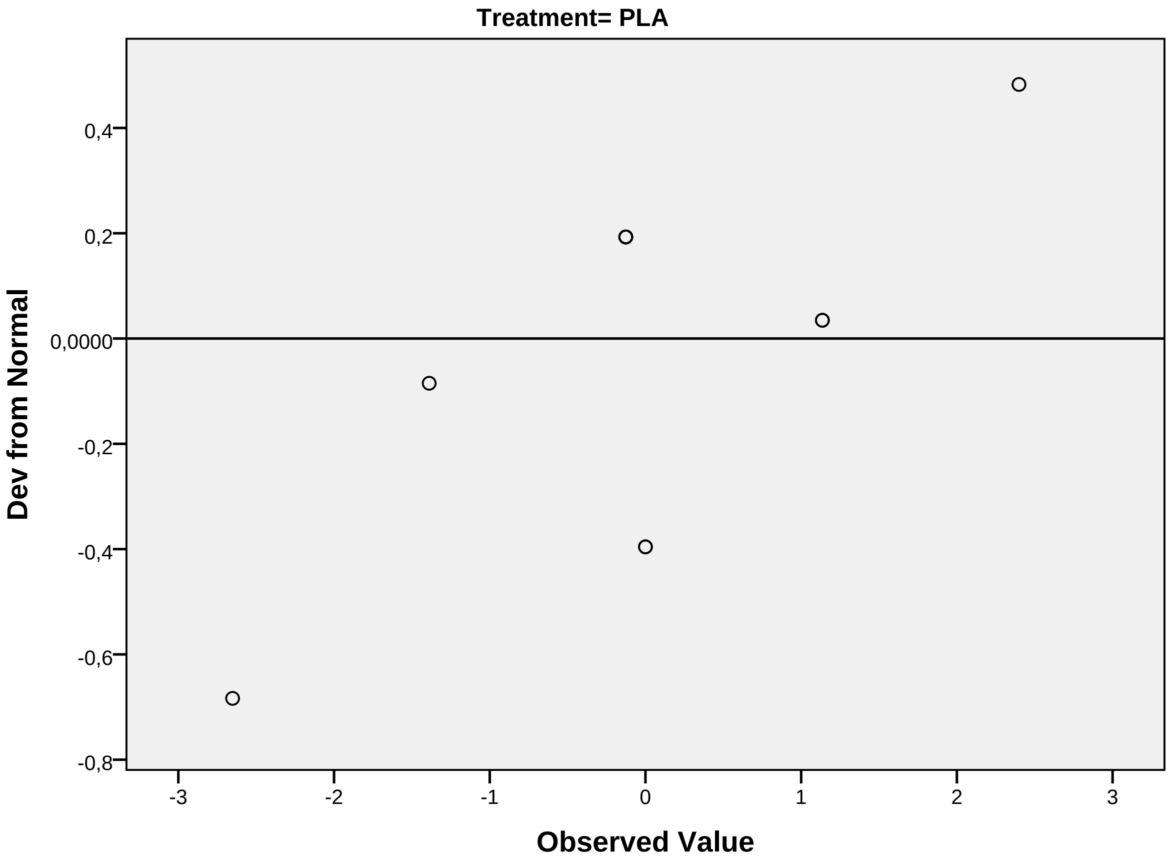

Detrended Normal Q-Q Plot of Standardized Residual for Sec_1

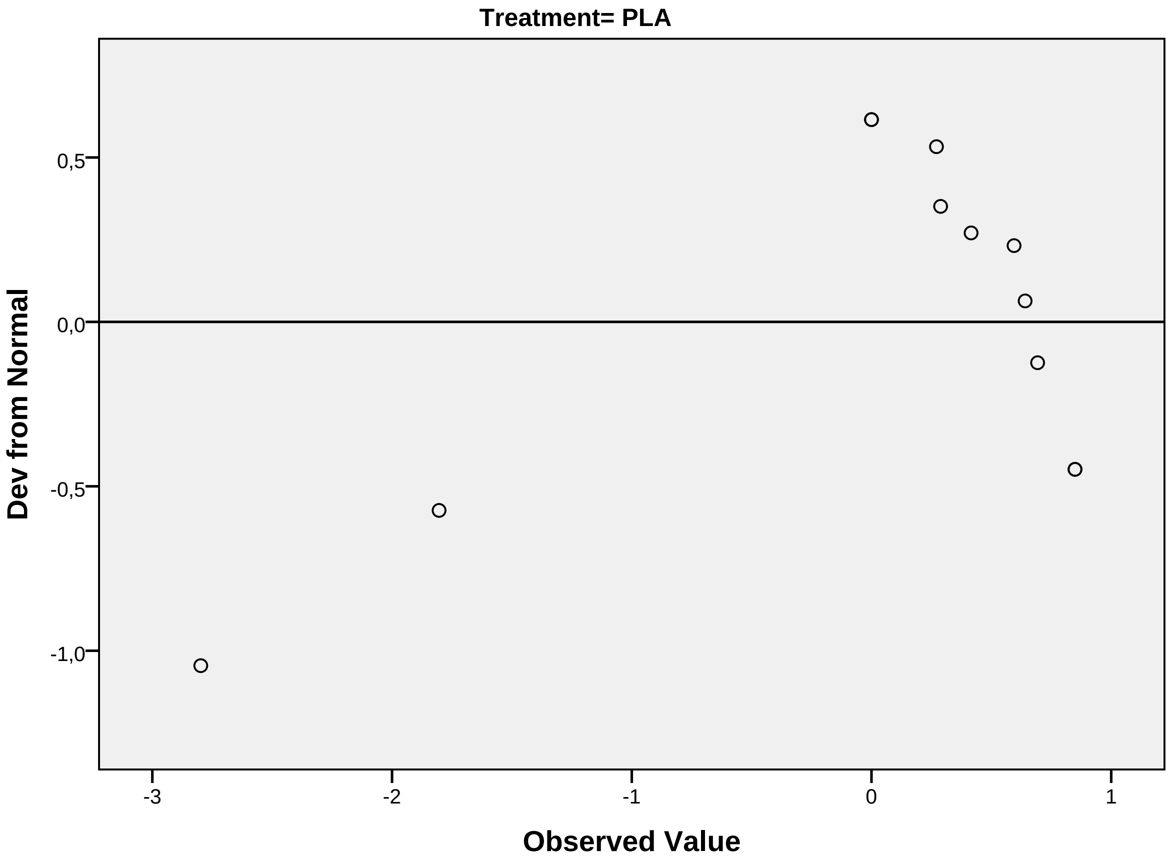

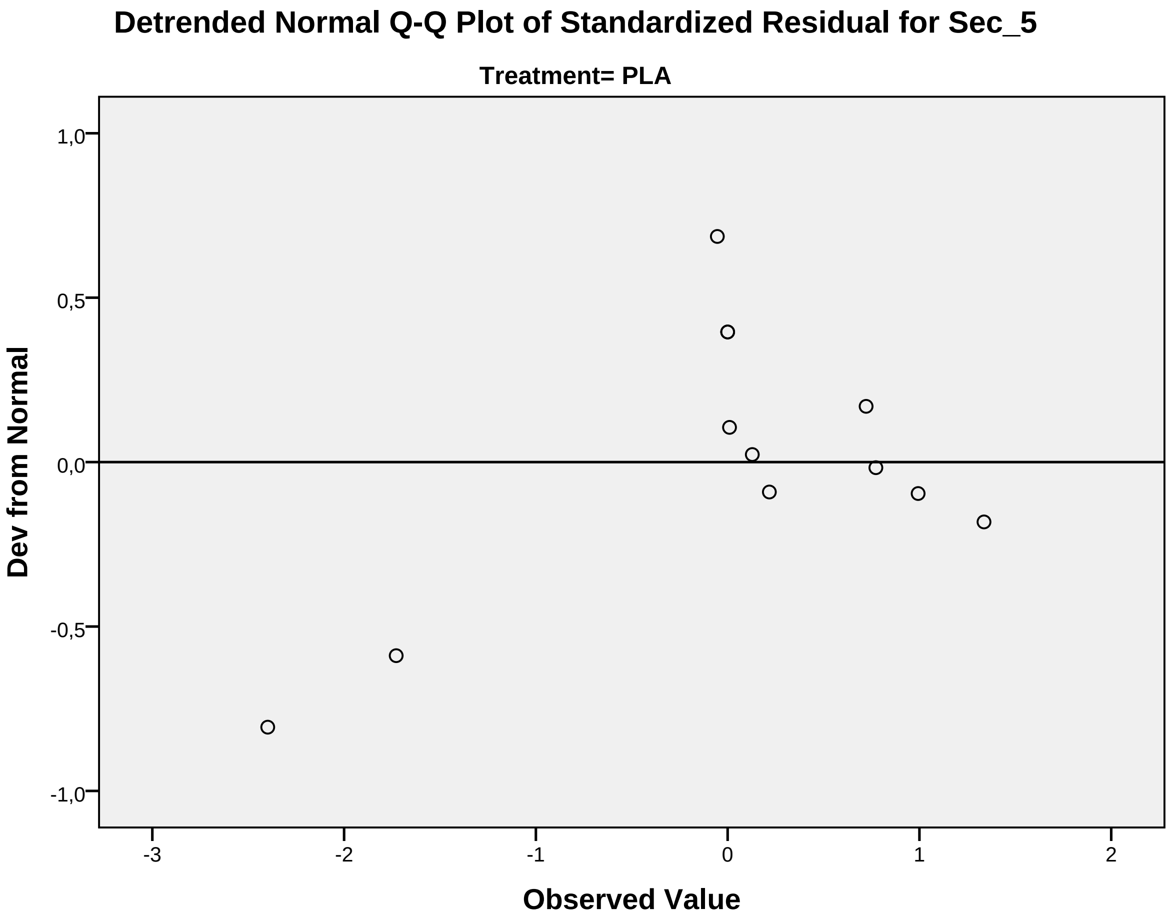
Detrended Normal Q-Q Plot of Standardized Residual for Sec_5
Treatment= PLA
Dev from Normal
1,0
0,5
0,0
-0,5
-1,0
-3
-2
-1
0
1
2
Observed Value

Detrended Normal Q-Q Plot of Standardized Residual for Sec_10

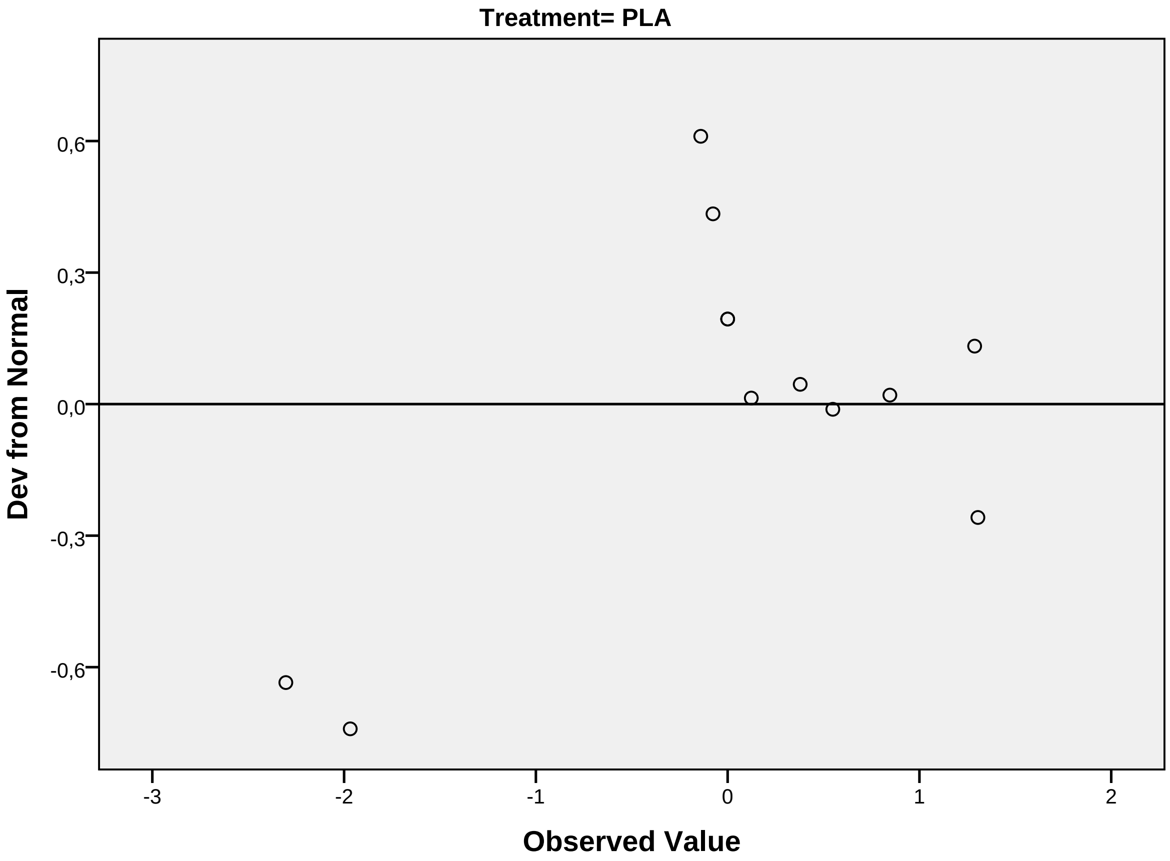

Detrended Normal Q-Q Plot of Standardized Residual for Sec_20

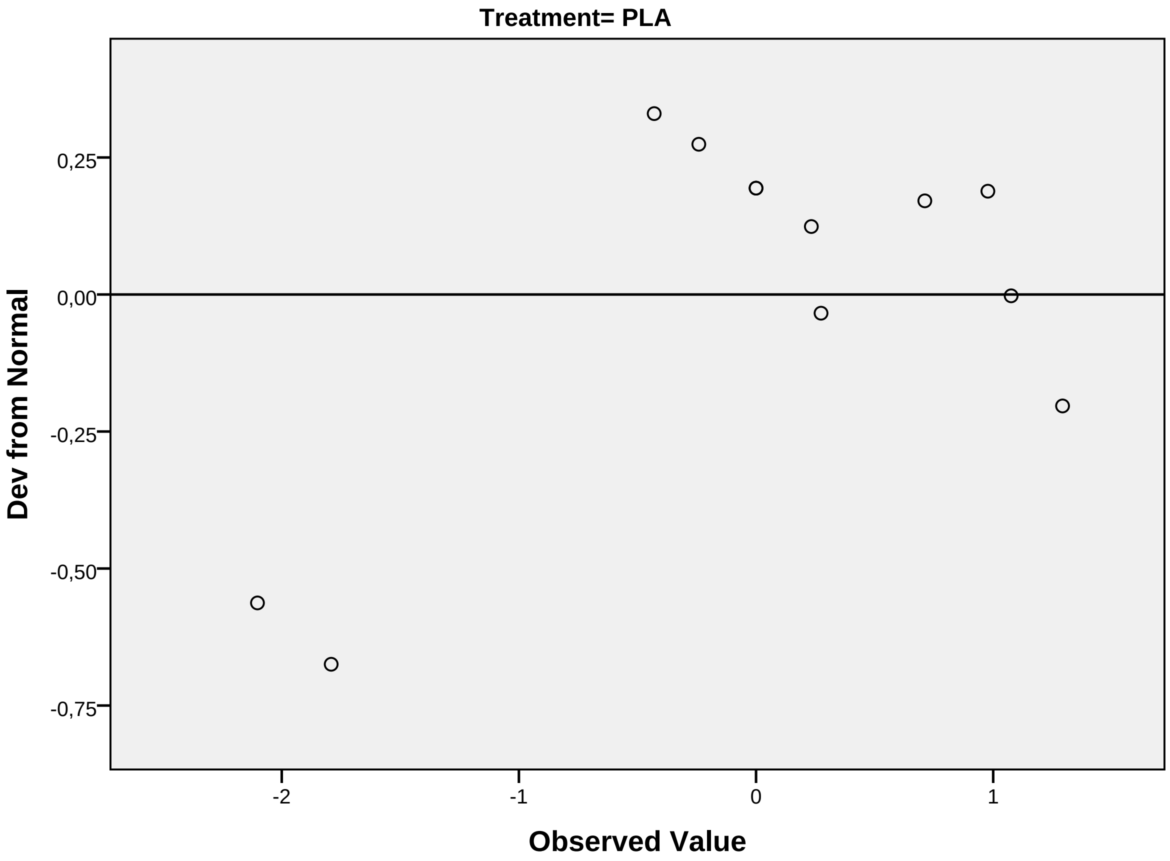

Detrended Normal Q-Q Plot of Standardized Residual for Sec_30

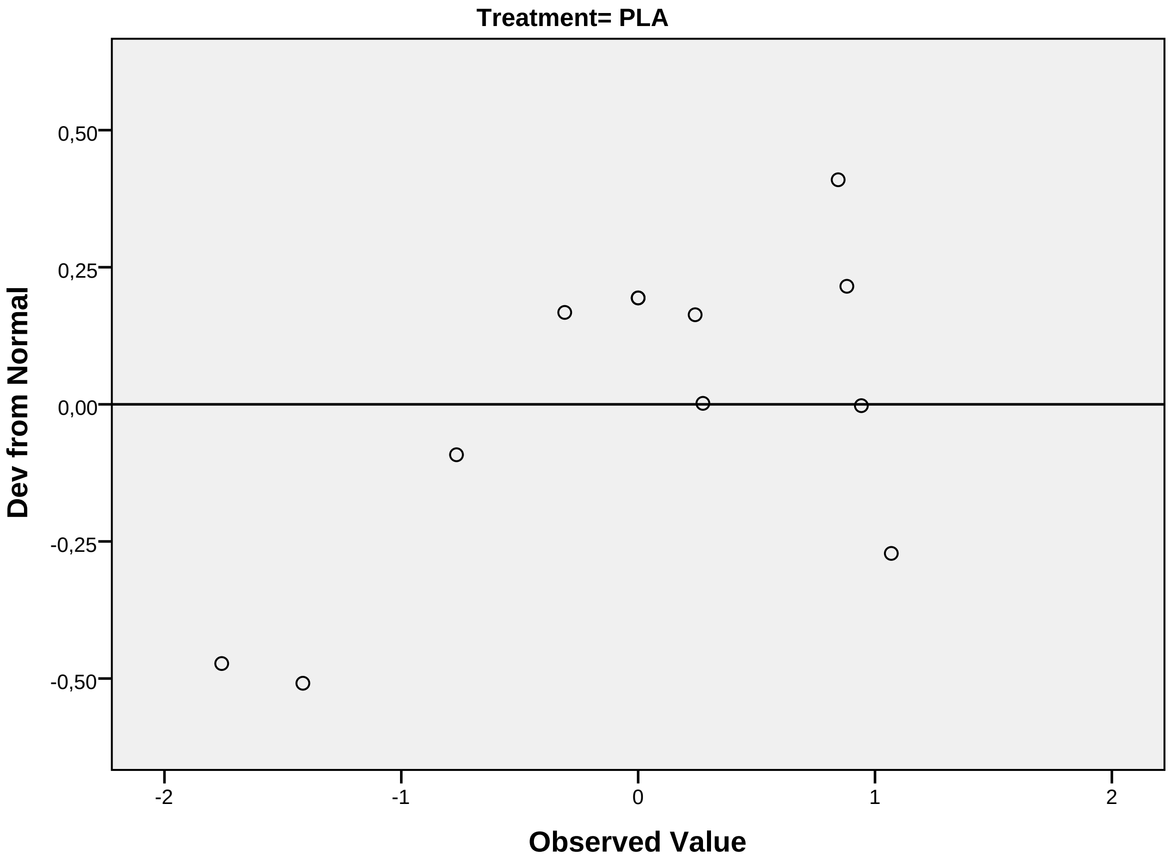

Detrended Normal Q-Q Plot of Standardized Residual for Sec_40

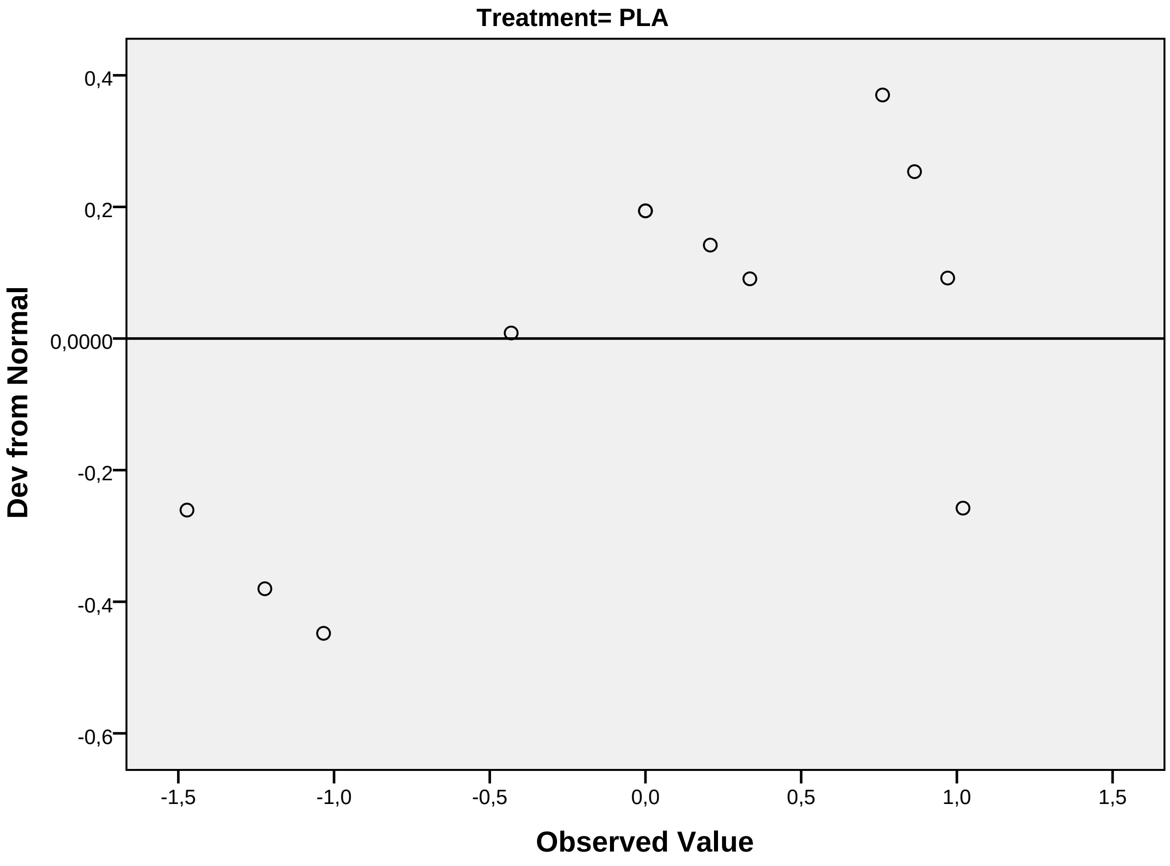

Detrended Normal Q-Q Plot of Standardized Residual for Sec_50

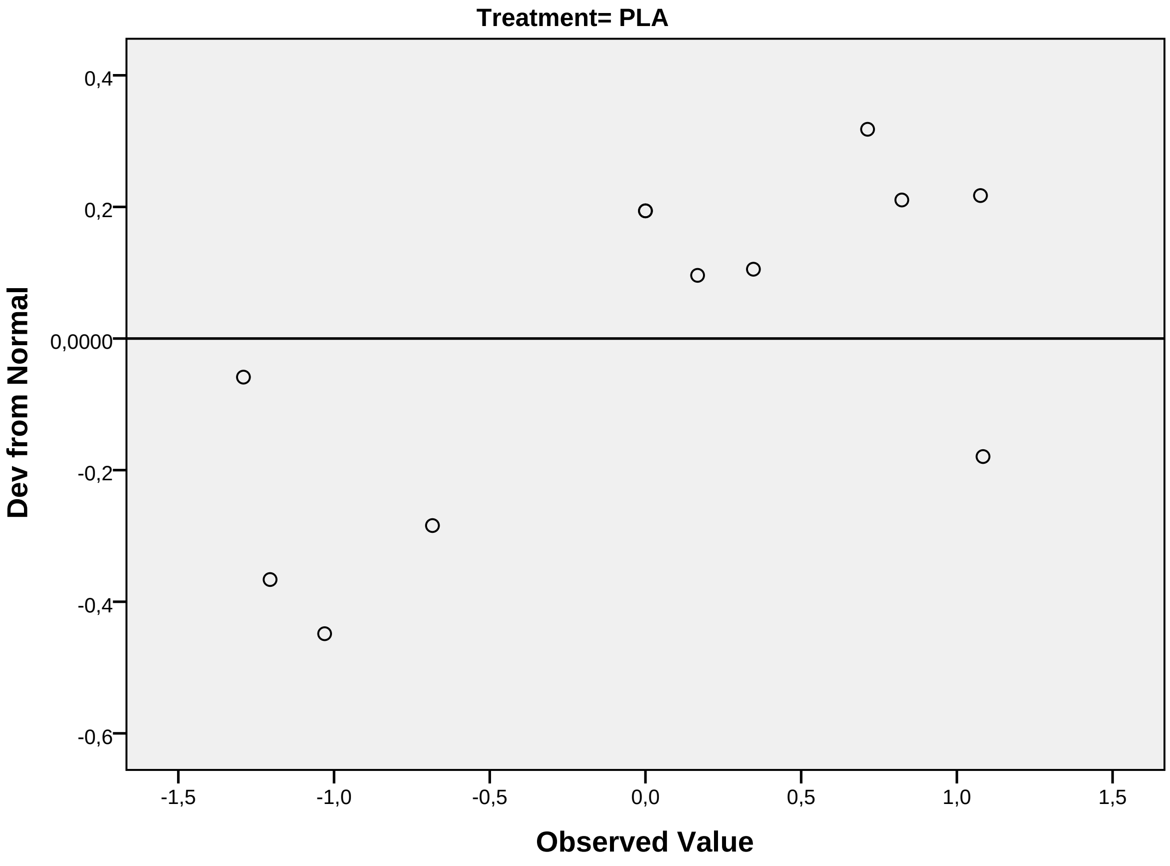

Detrended Normal Q-Q Plot of Standardized Residual for Sec_60

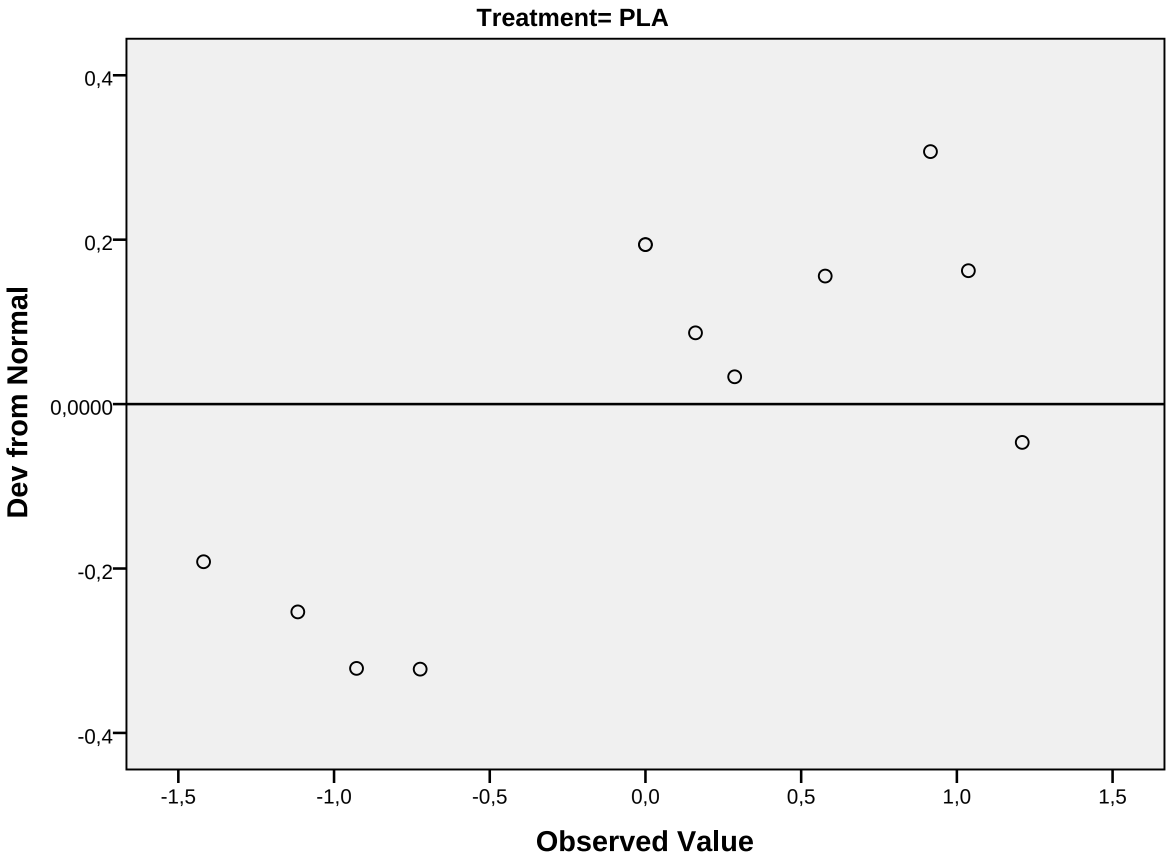

Treatment: PLA

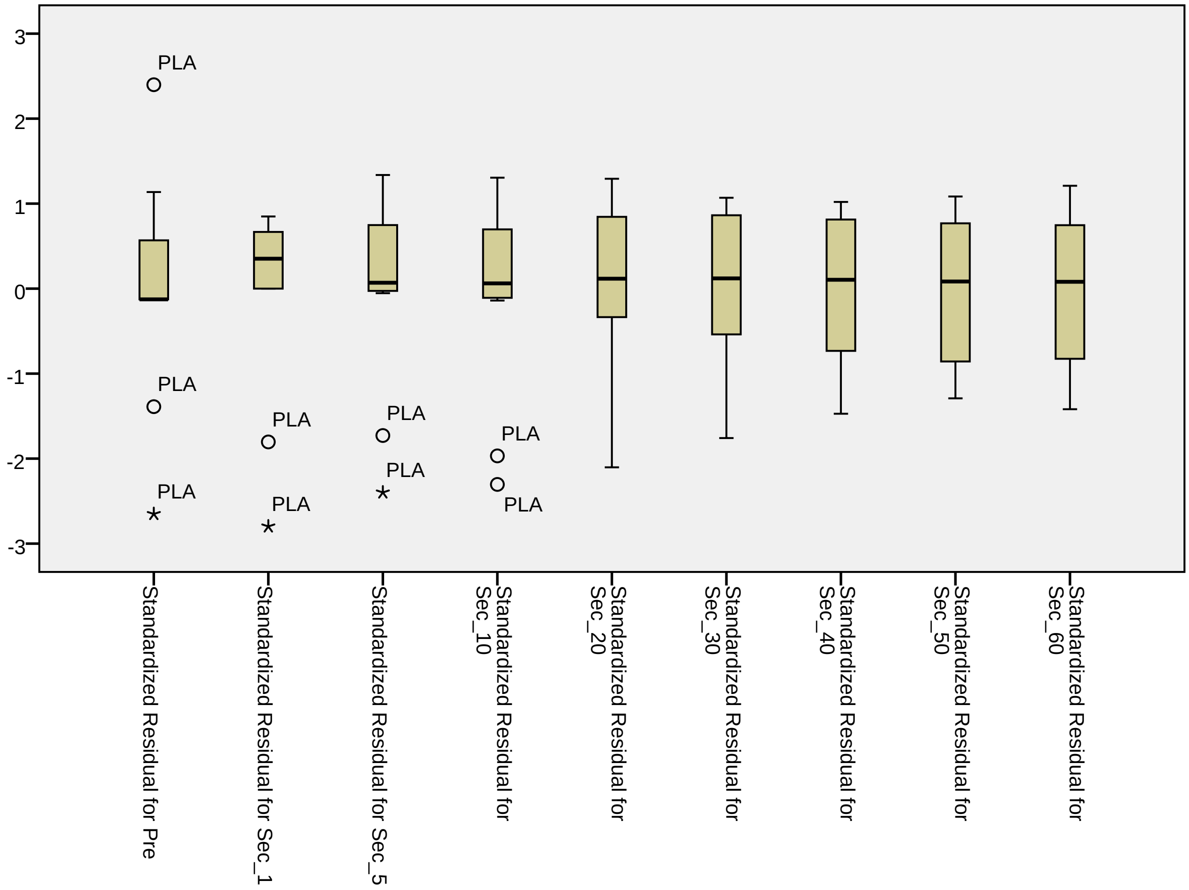

Normal Q-Q Plots of Standardized Residual for tHb During Recovery

Normal Q-Q Plot of Standardized Residual for Pre

Treatment= BG

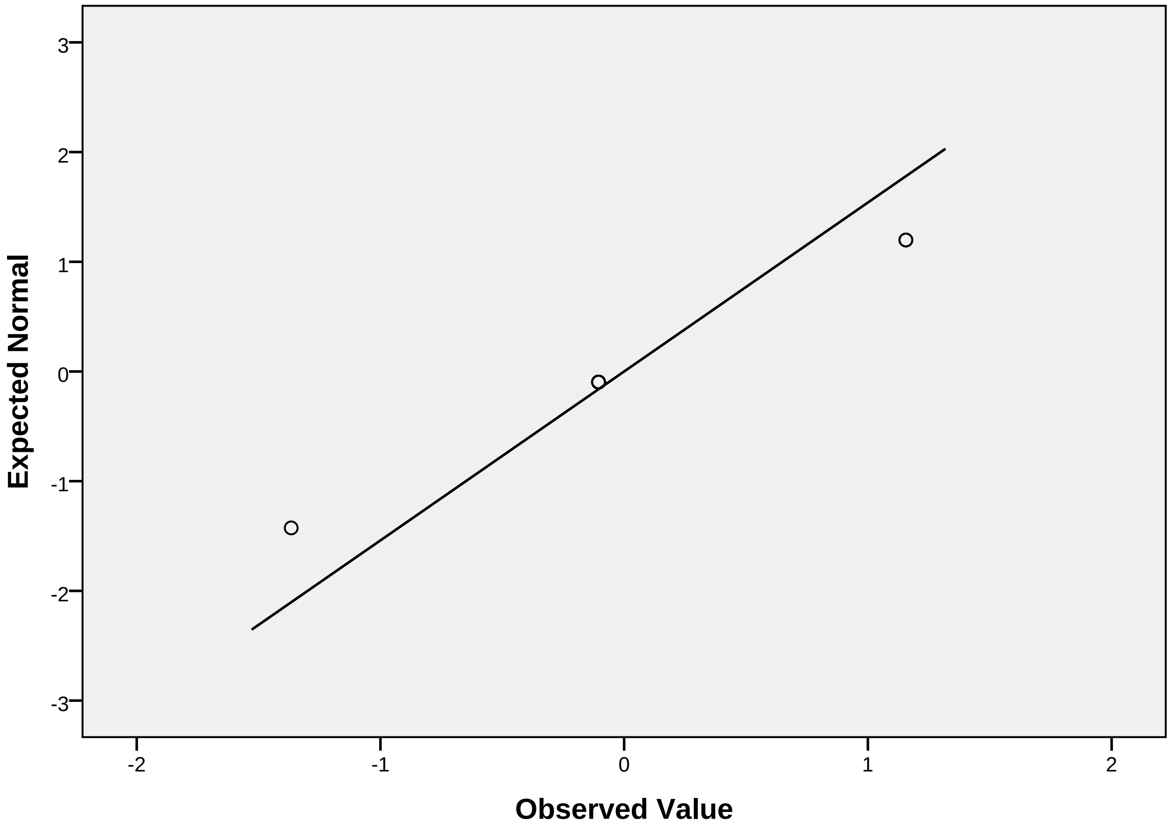

Normal Q-Q Plot of Standardized Residual for Post

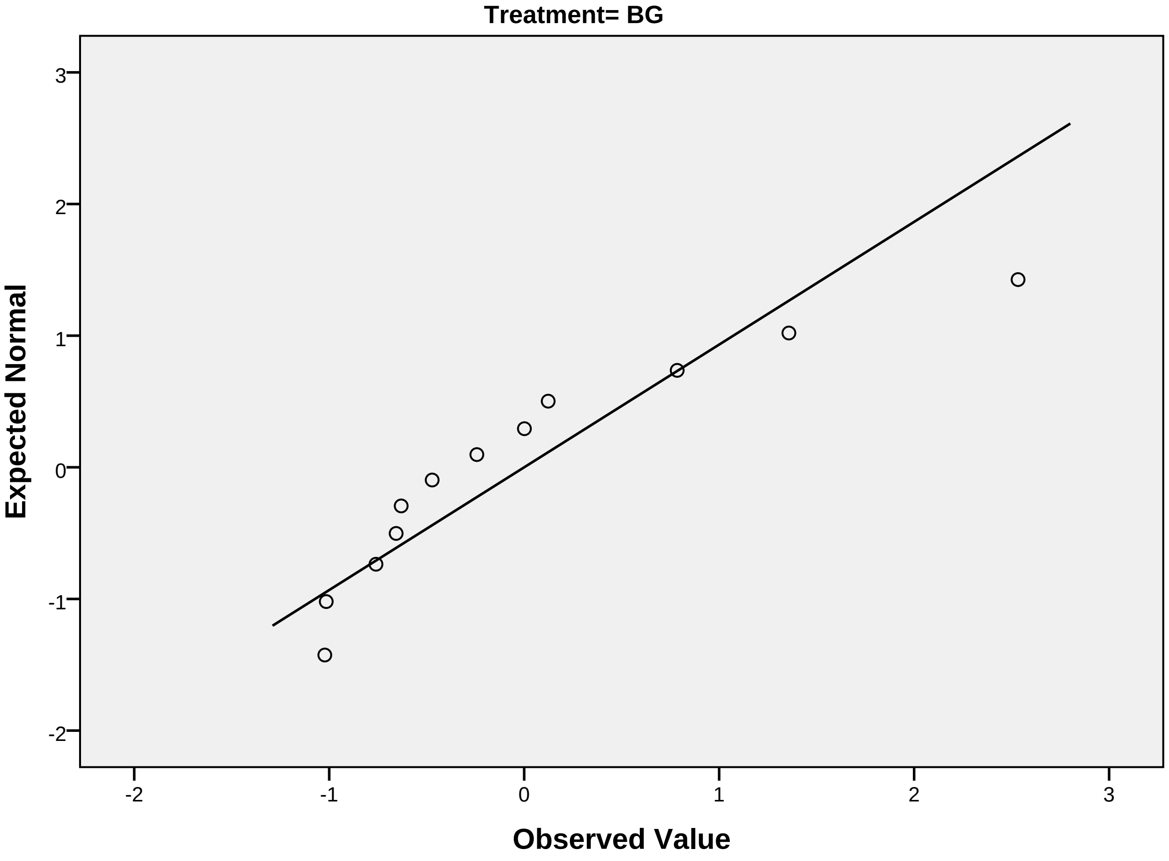

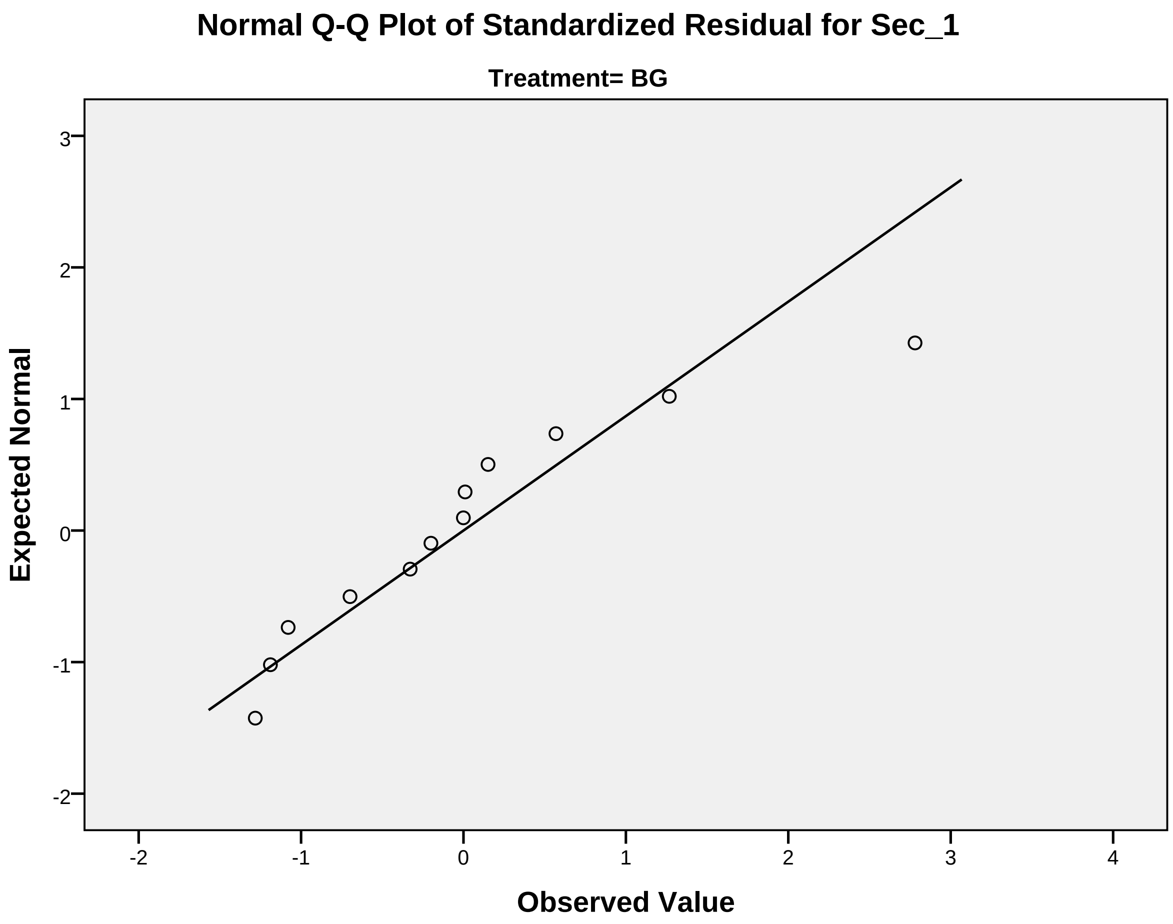

Normal Q-Q Plot of Standardized Residual for Sec_1
Treatment= BG
Expected Normal
Observed Value
3
2
1
0
-1
-2
-2
-1
0
1
2
3
4

Normal Q-Q Plot of Standardized Residual for Sec_5

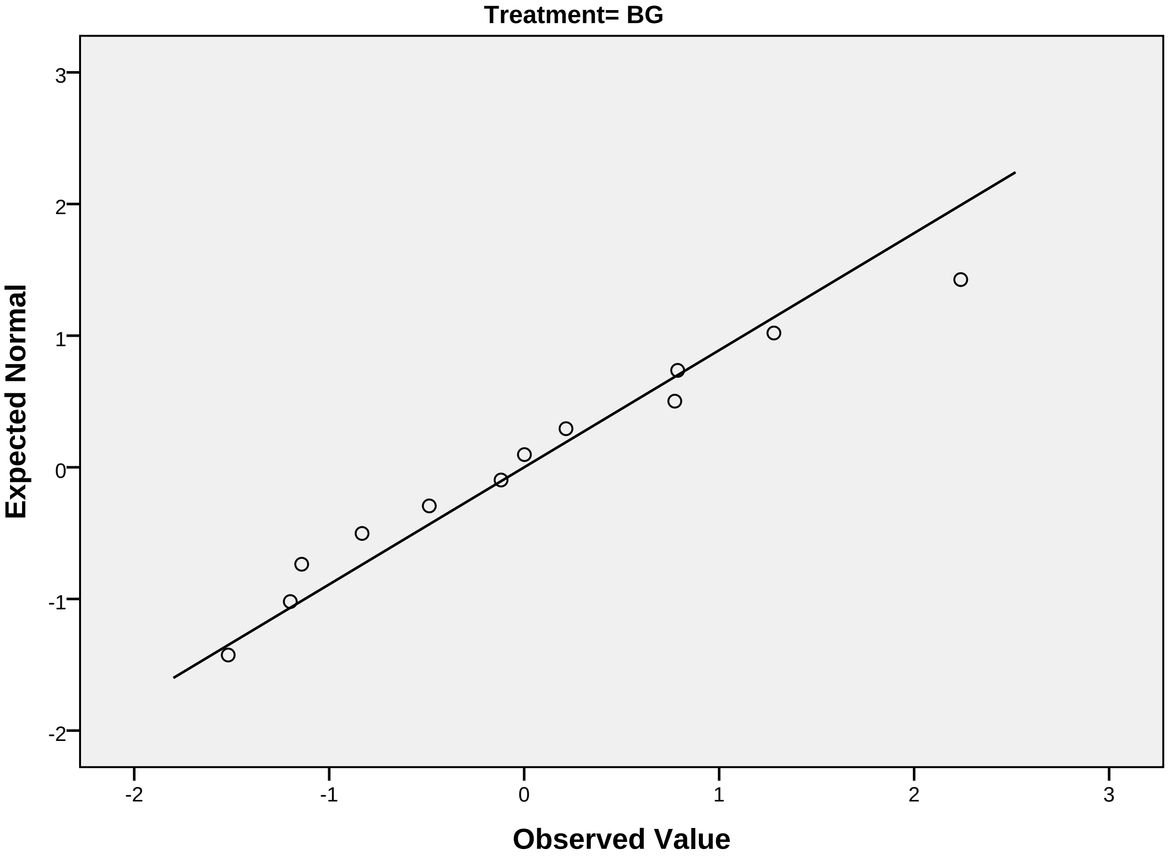

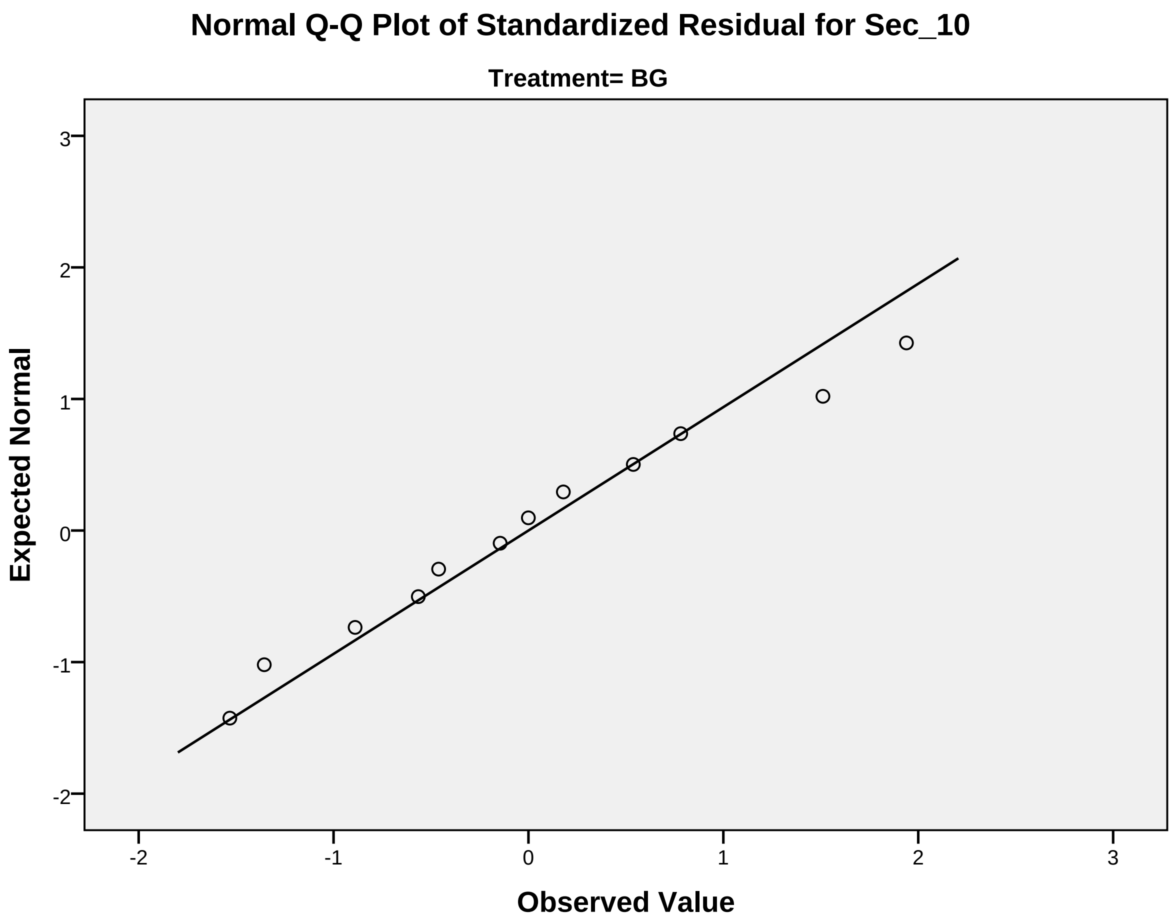
Normal Q-Q Plot of Standardized Residual for Sec_10
Treatment= BG
Expected Normal
3
2
1
0
-1
-2
-2
-1
0
1
2
3
Observed Value

Normal Q-Q Plot of Standardized Residual for Sec_20

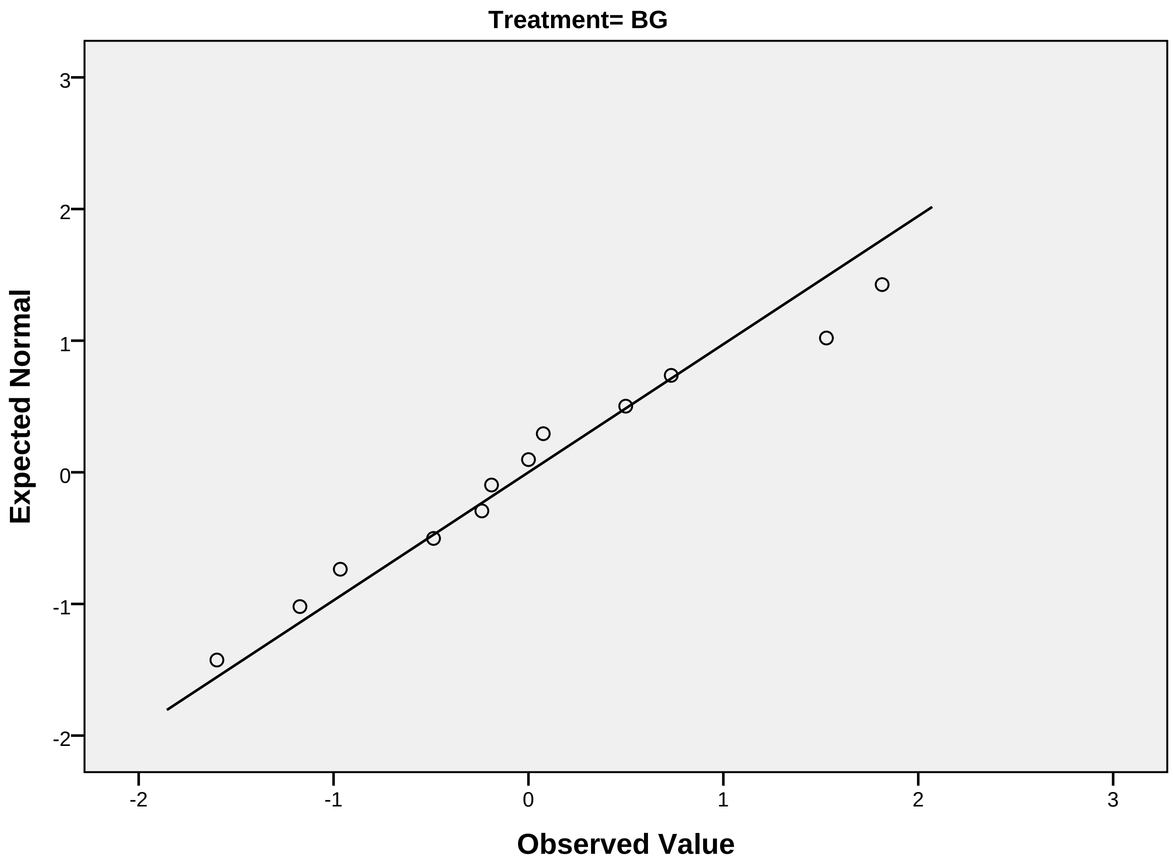

Normal Q-Q Plot of Standardized Residual for Sec_30

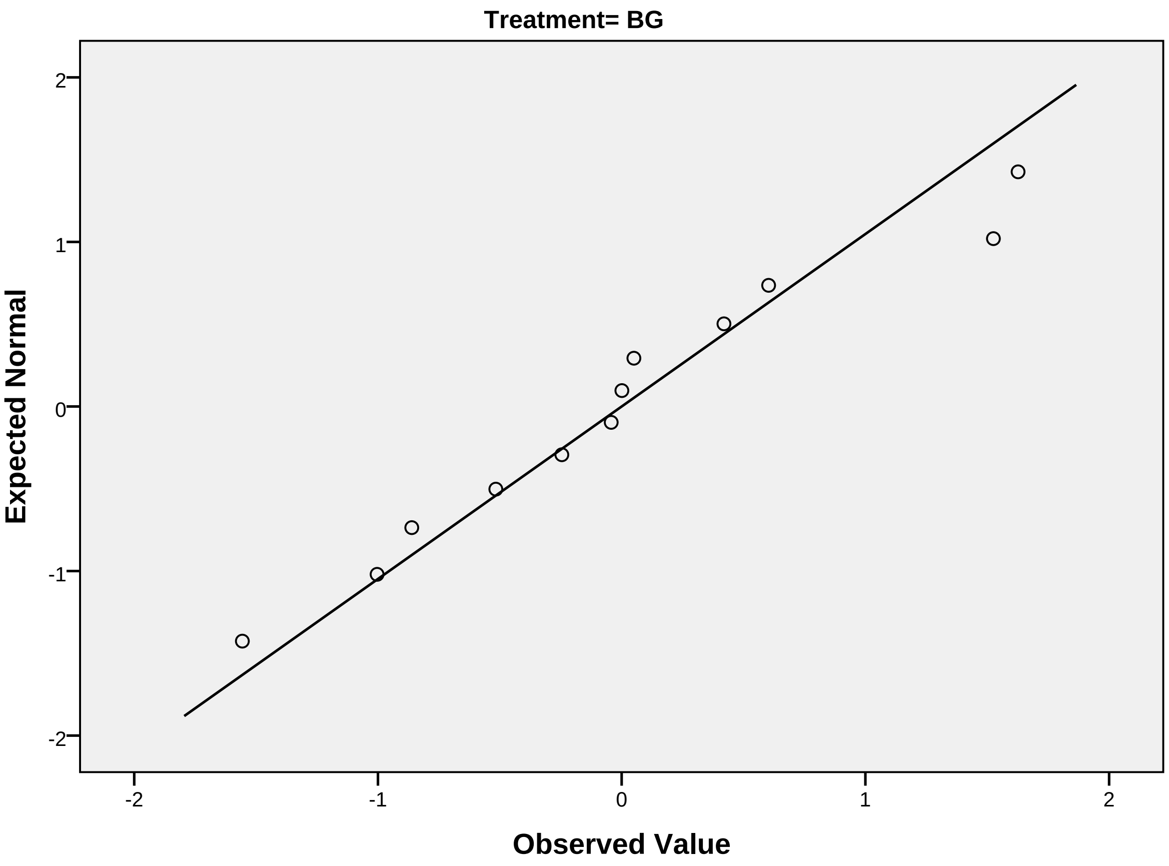

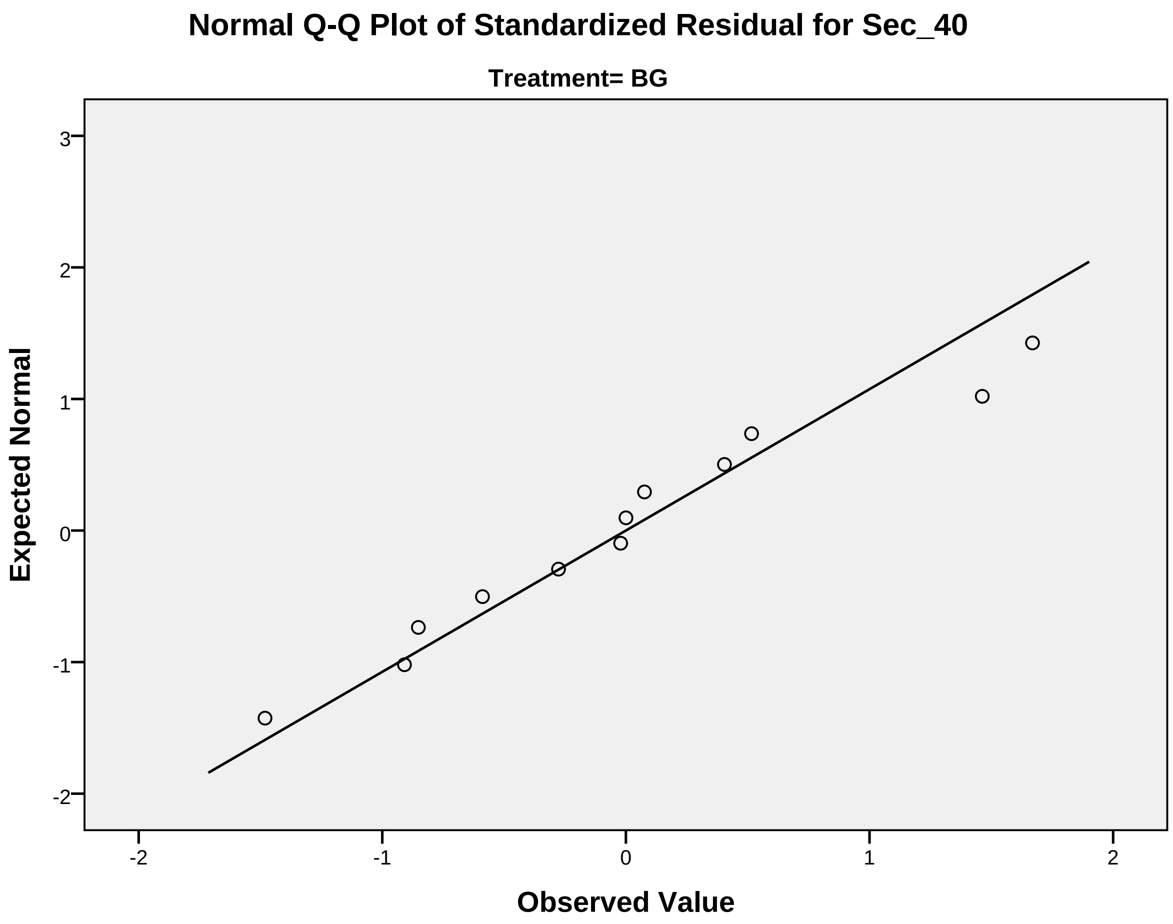
Normal Q-Q Plot of Standardized Residual for Sec_40
Treatment= BG
Expected Normal
3
2
1
0
-1
-2
-2
-1
0
1
2
Observed Value

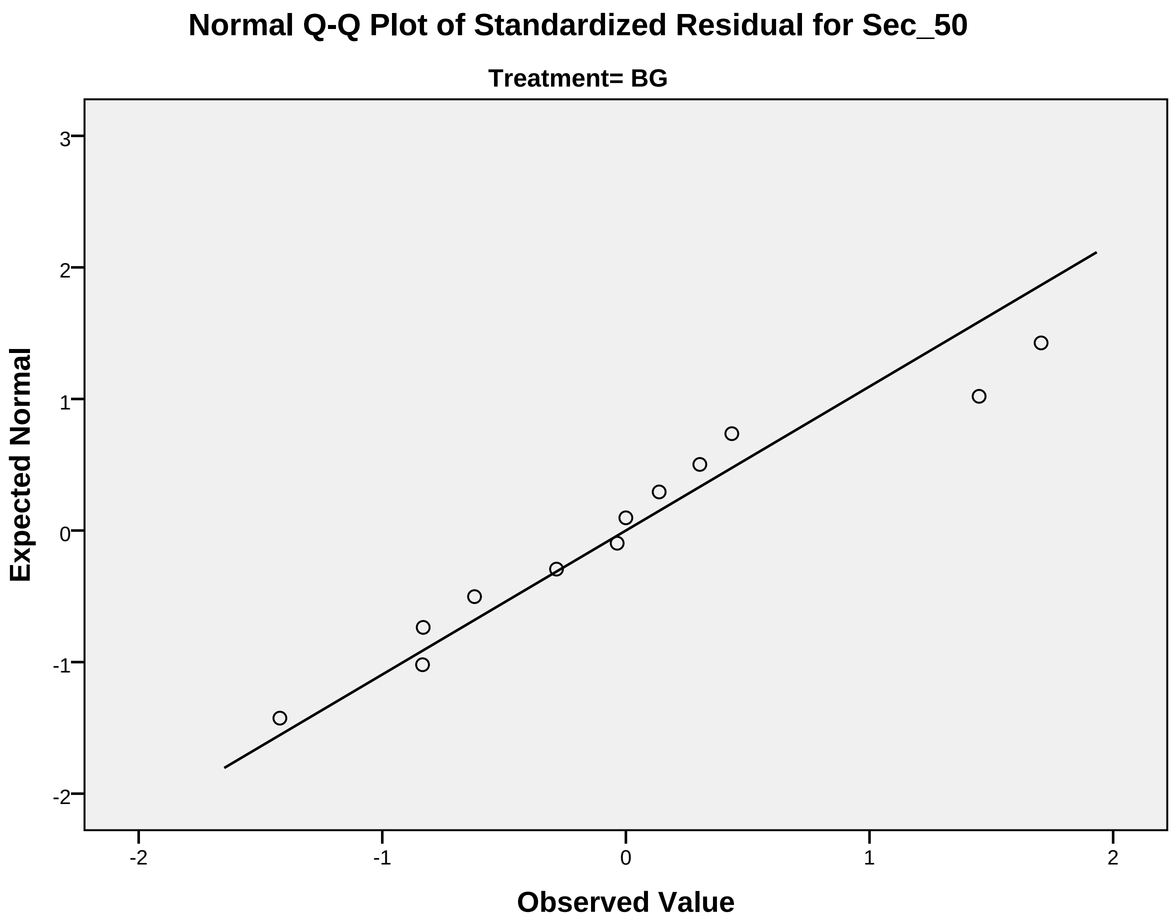
Normal Q-Q Plot of Standardized Residual for Sec_50
Treatment= BG
Expected Normal
3
2
1
0
-1
-2
-2
-1
0
1
2
Observed Value

Normal Q-Q Plot of Standardized Residual for Sec_60

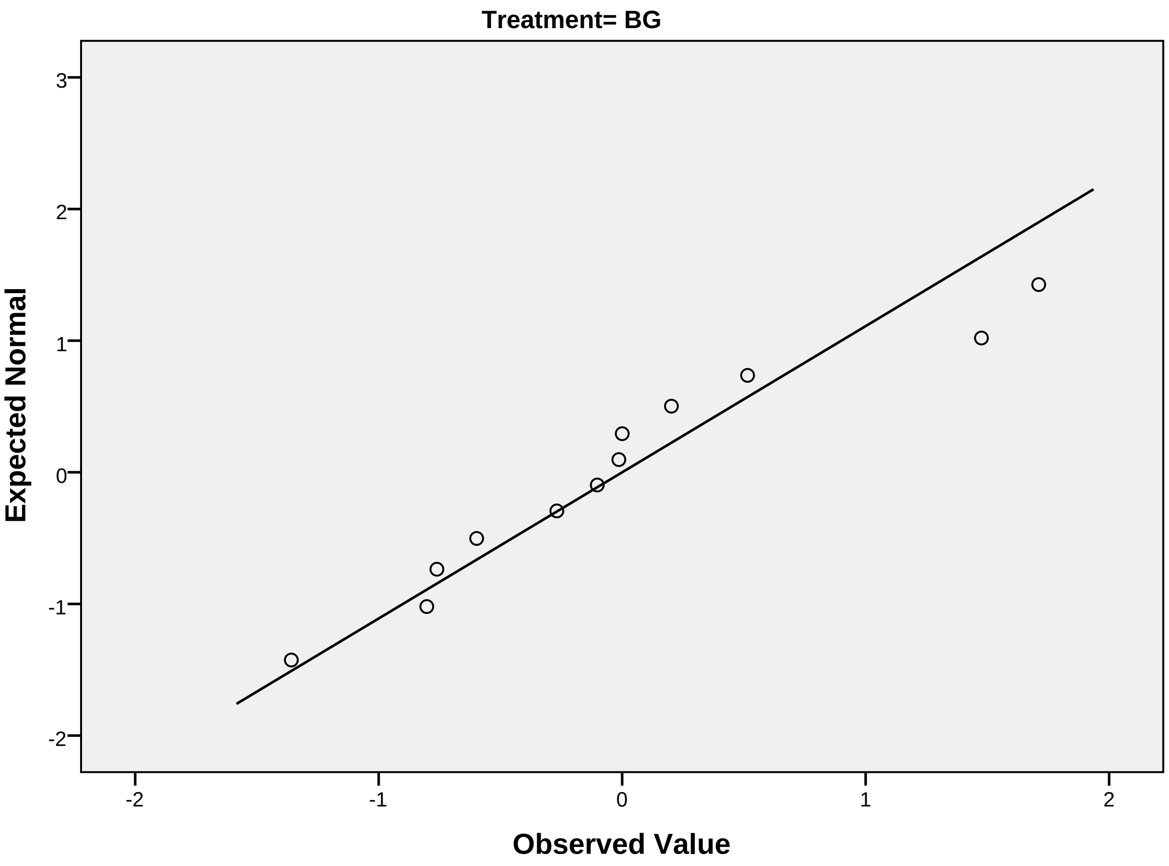

Detrended Normal Q-Q Plots of Standardized Residual for tHb During Recovery

Detrended Normal Q-Q Plot of Standardized Residual for Pre

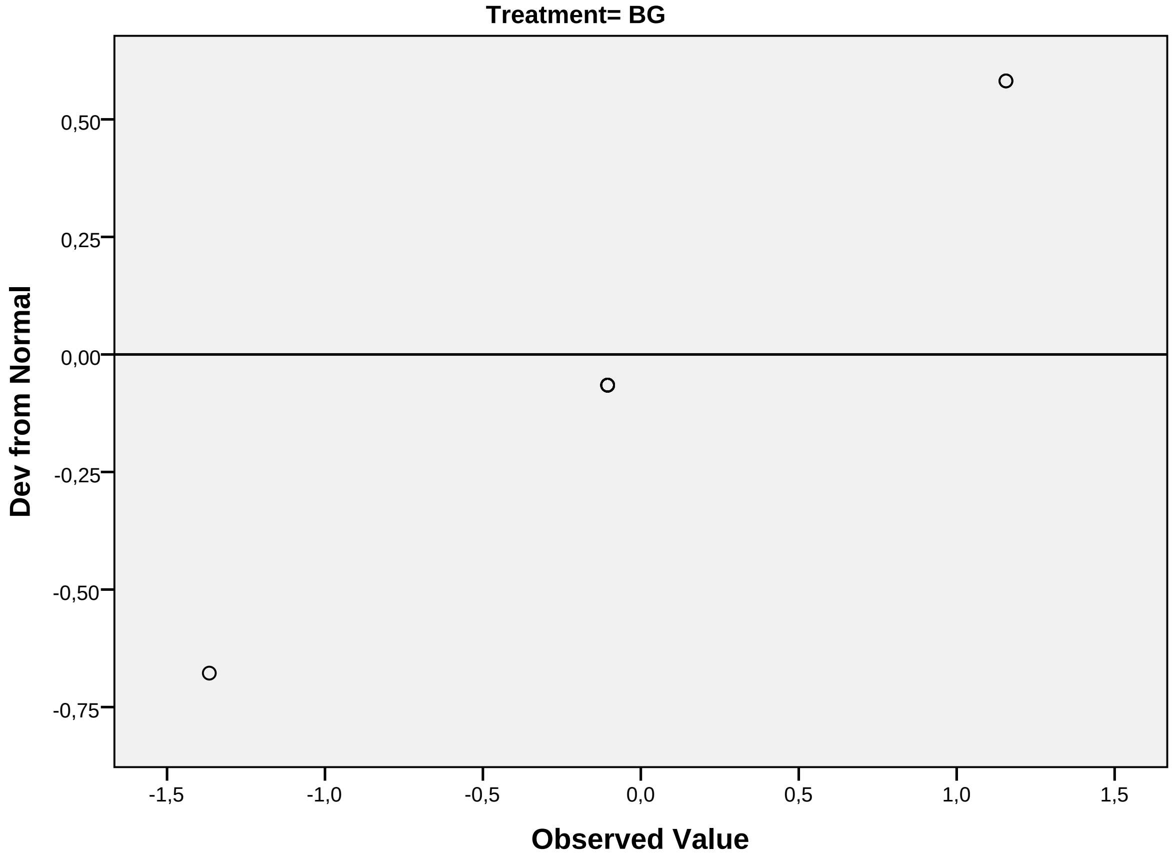

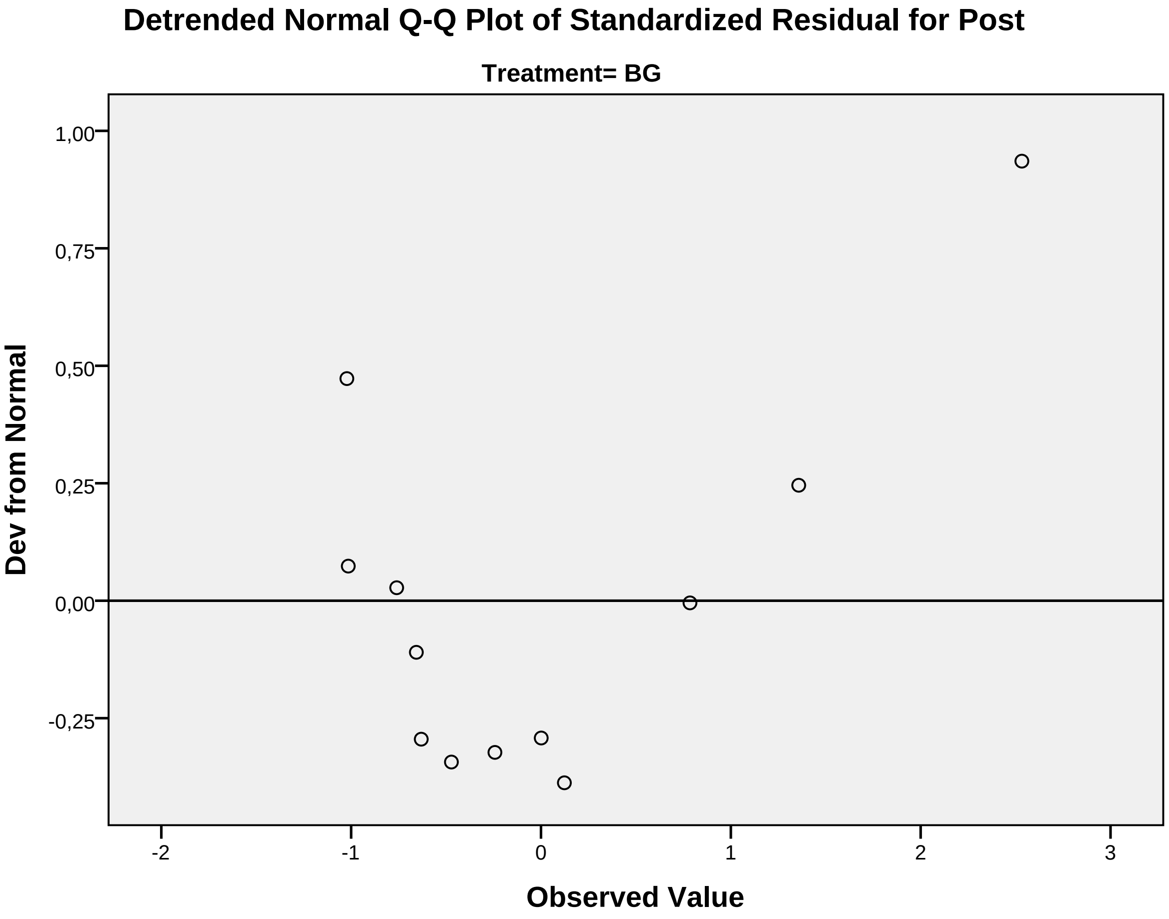
Detrended Normal Q-Q Plot of Standardized Residual for Post
Treatment= BG
Dev from Normal
1,00
0,75
0,50
0,25
0,00
-0,25
-2
-1
0
1
2
3
Observed Value

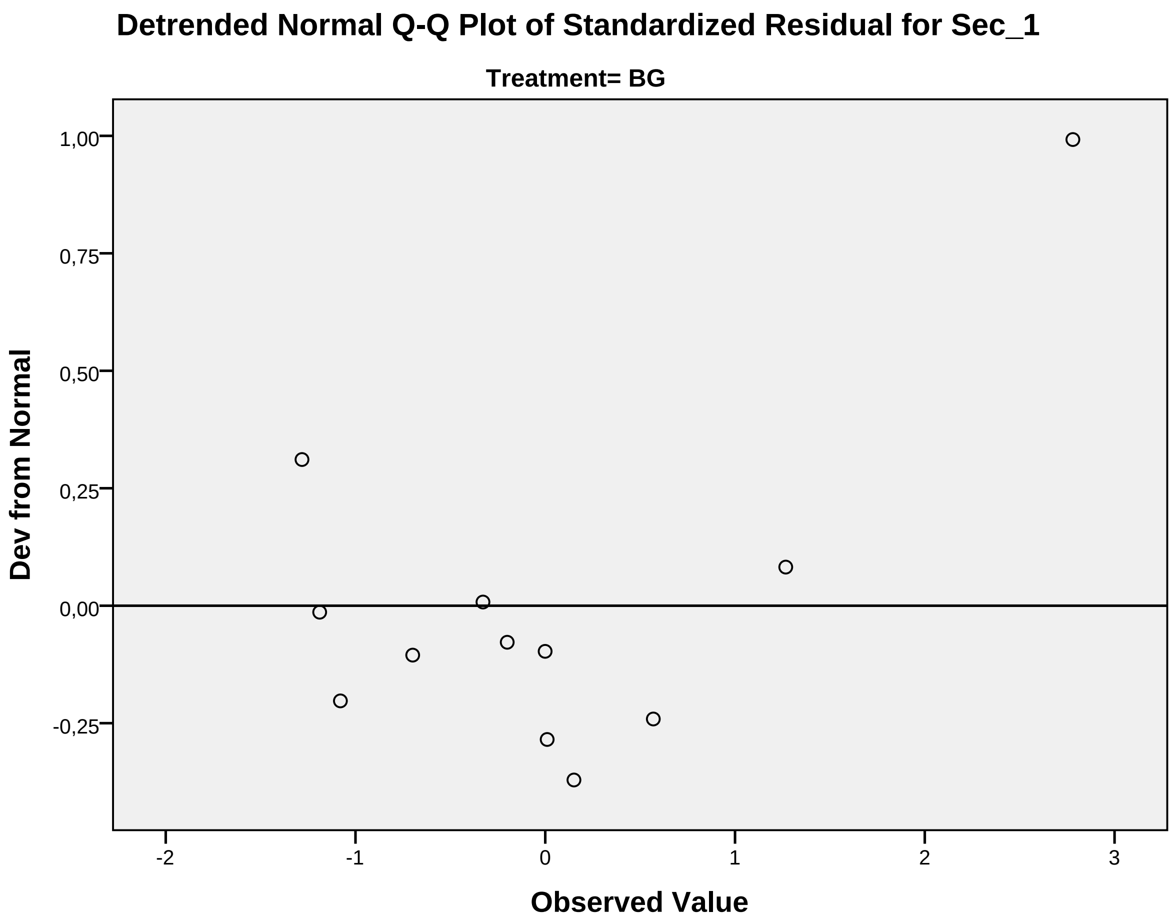
Detrended Normal Q-Q Plot of Standardized Residual for Sec_1
Treatment= BG
Dev from Normal
1,00
0,75
0,50
0,25
0,00
-0,25
-2
-1
0
1
2
3
Observed Value

Detrended Normal Q-Q Plot of Standardized Residual for Sec_5

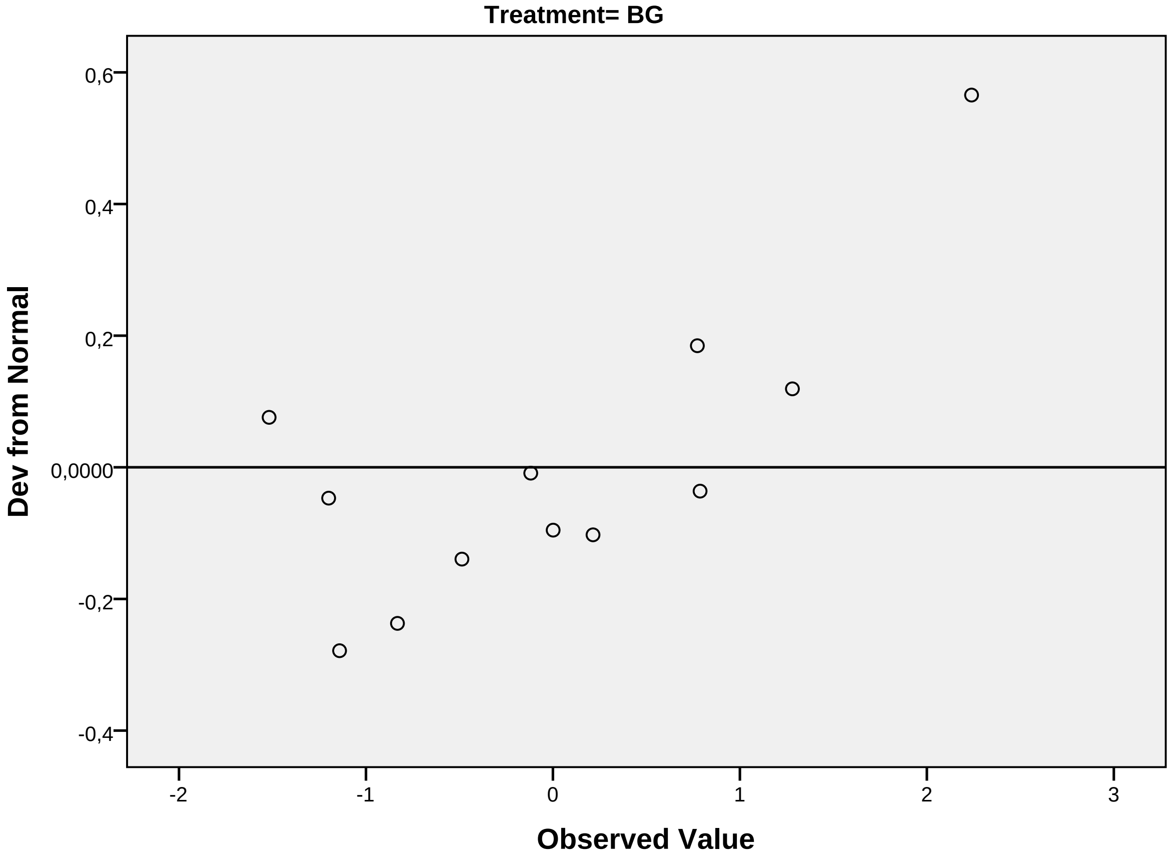

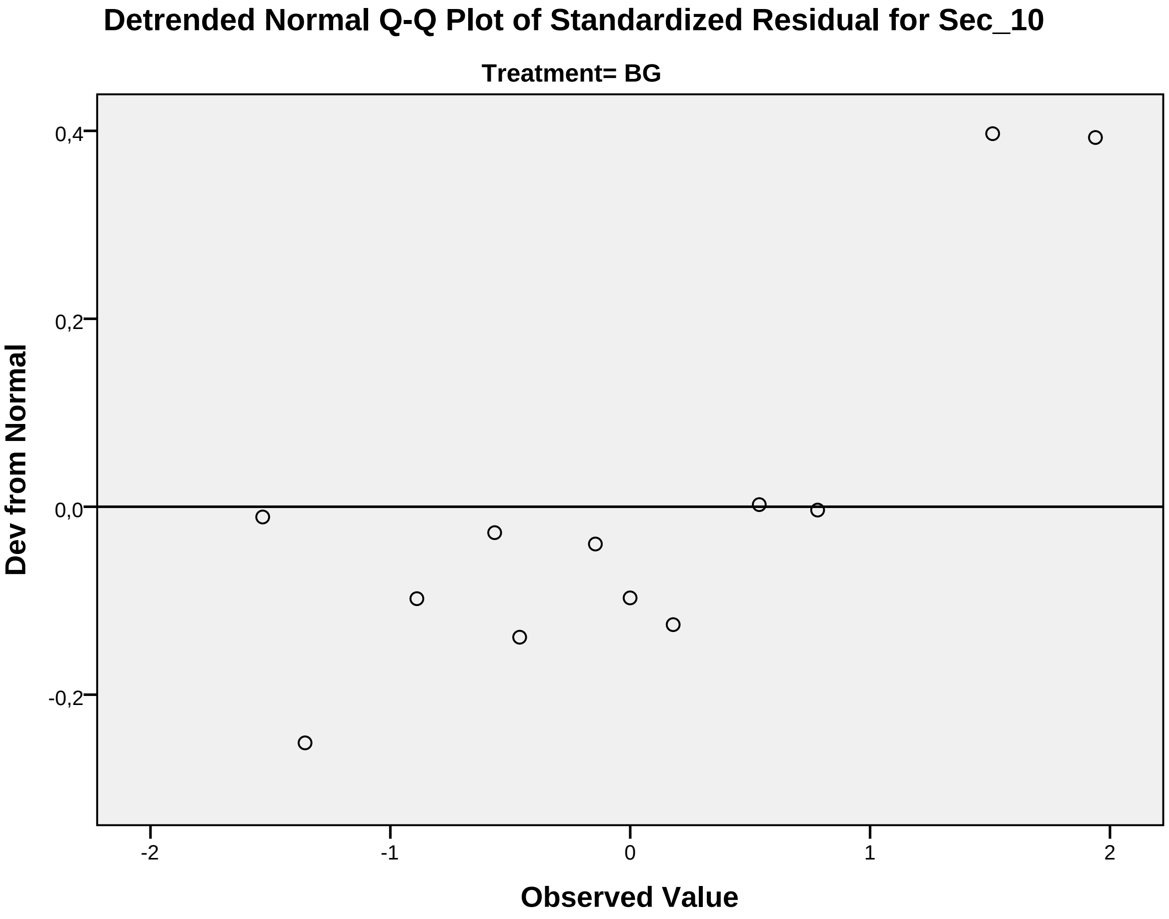

Detrended Normal Q-Q Plot of Standardized Residual for Sec_10
Treatment= BG
Dev from Normal
0,4
0,2
0,0
-0,2
-2
-1
0
1
2
Observed Value

Detrended Normal Q-Q Plot of Standardized Residual for Sec_20

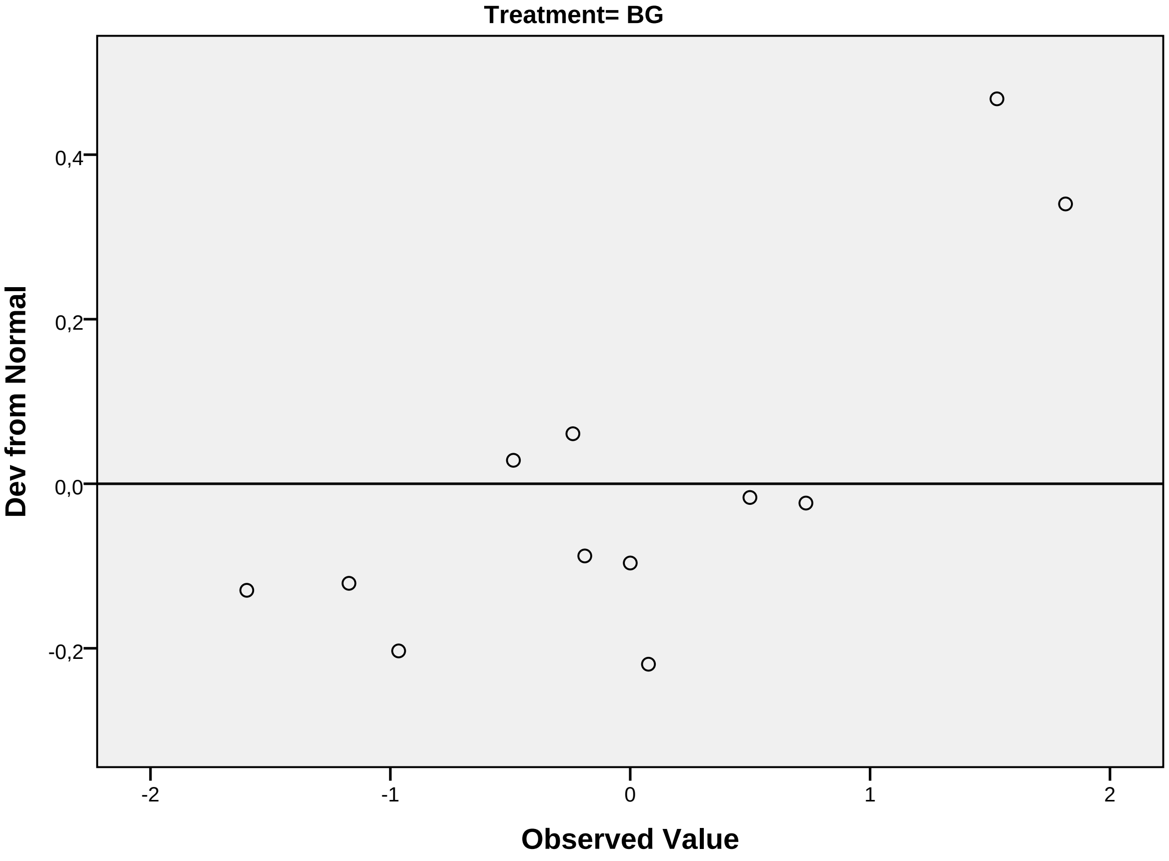

Detrended Normal Q-Q Plot of Standardized Residual for Sec_30

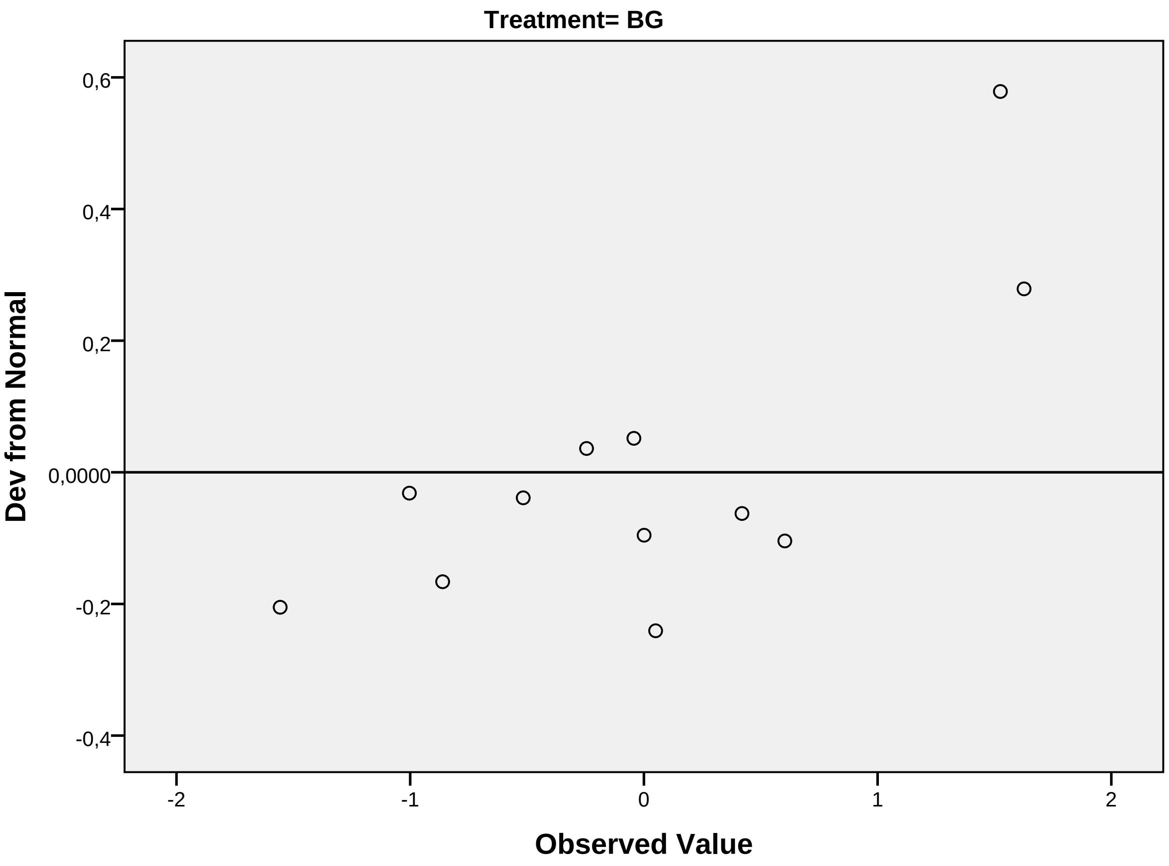

Detrended Normal Q-Q Plot of Standardized Residual for Sec_40

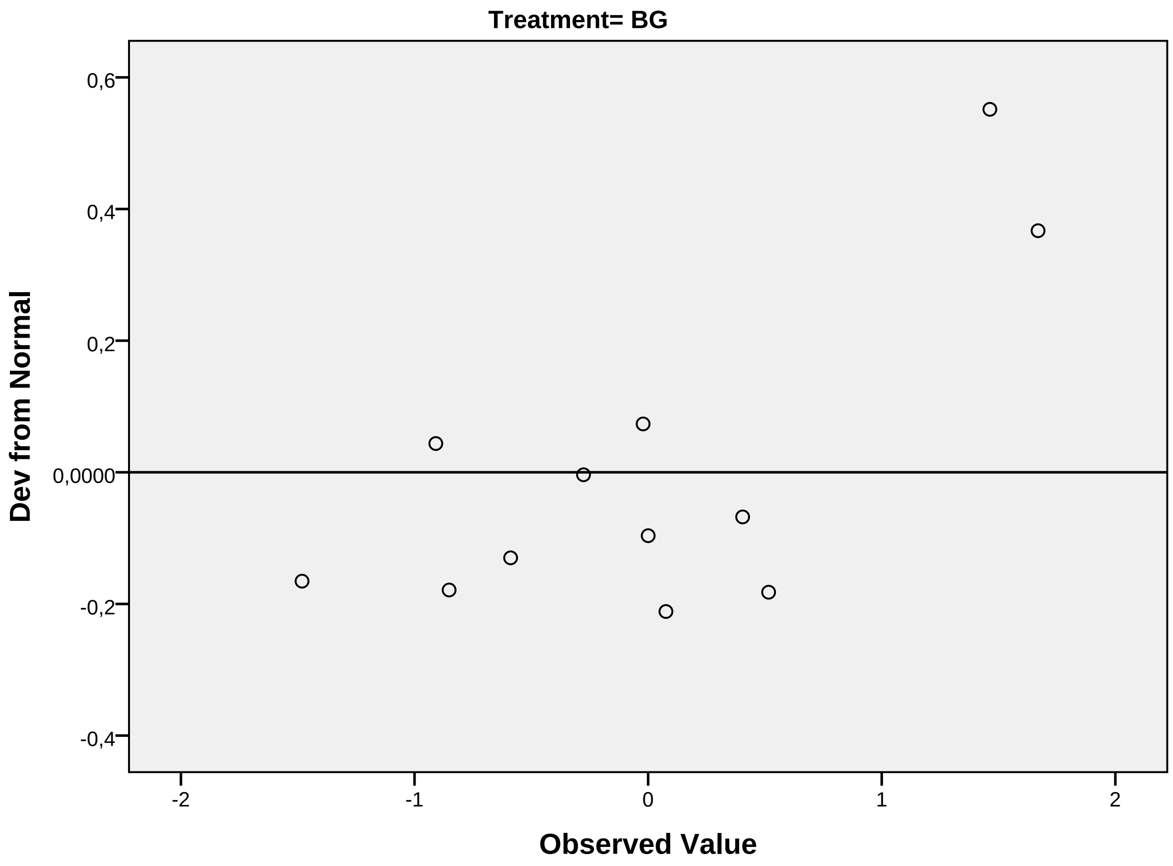

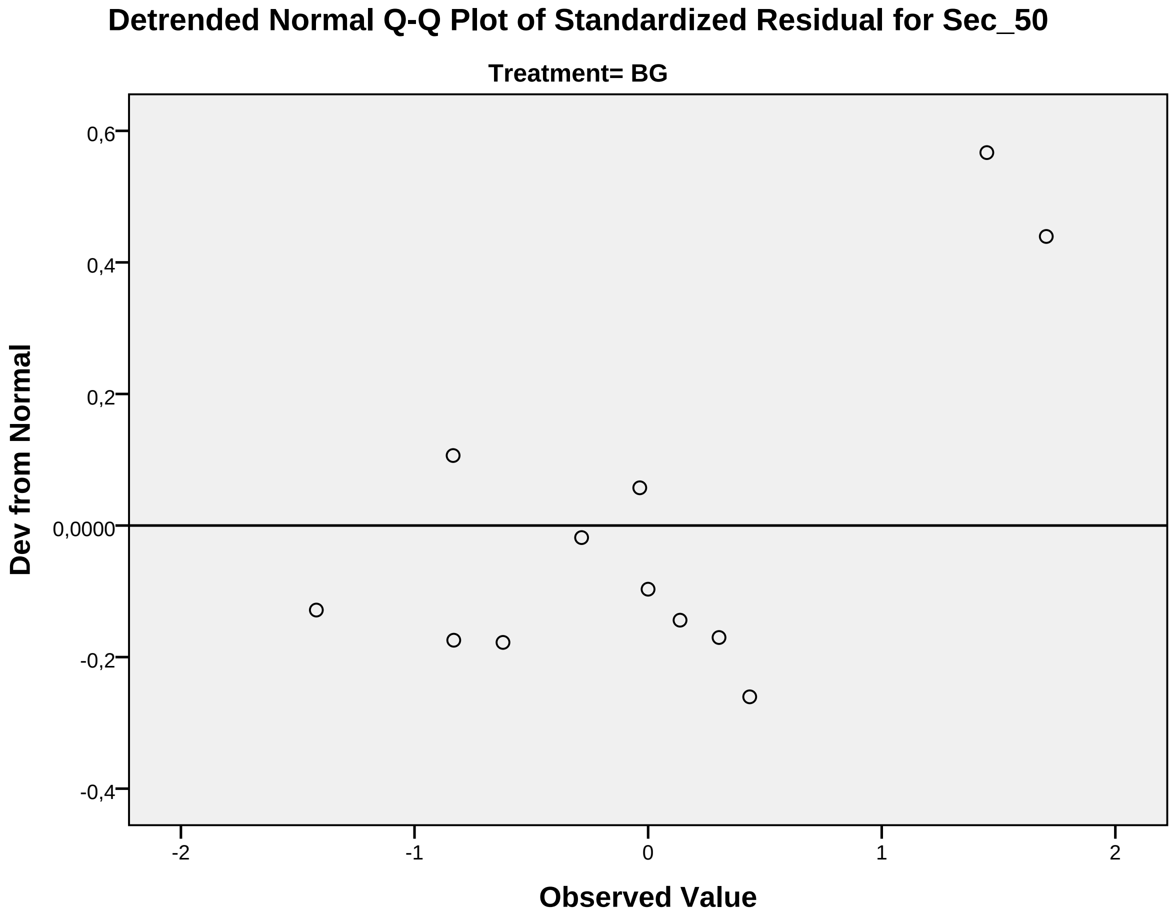
Detrended Normal Q-Q Plot of Standardized Residual for Sec_50
Treatment= BG
Dev from Normal
0,6
0,4
0,2
0,0000
-0,2
-0,4
-2
-1
0
1
2
Observed Value

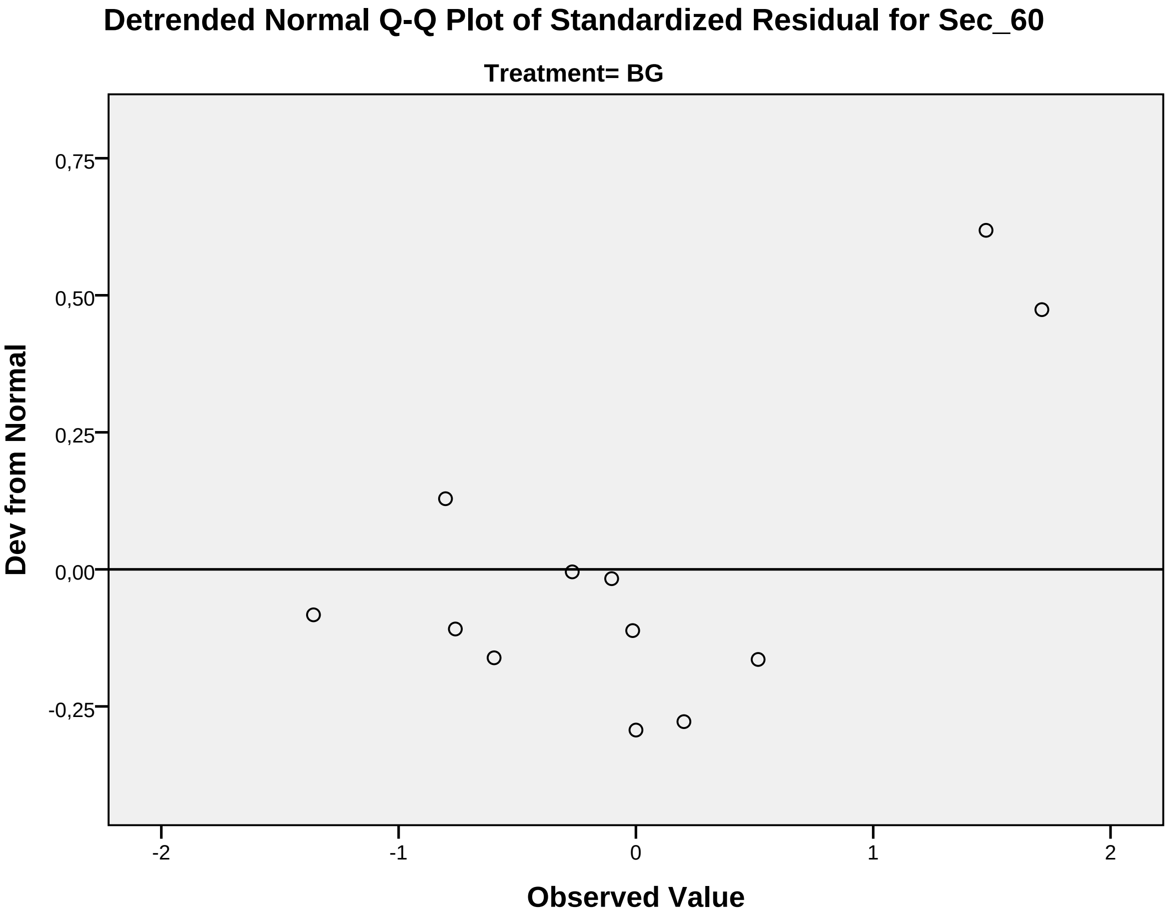

Detrended Normal Q-Q Plot of Standardized Residual for Sec_60
Treatment= BG
Dev from Normal
0,75
0,50
0,25
0,00
-0,25
-2
-1
0
1
2
Observed Value

Treatment: BG

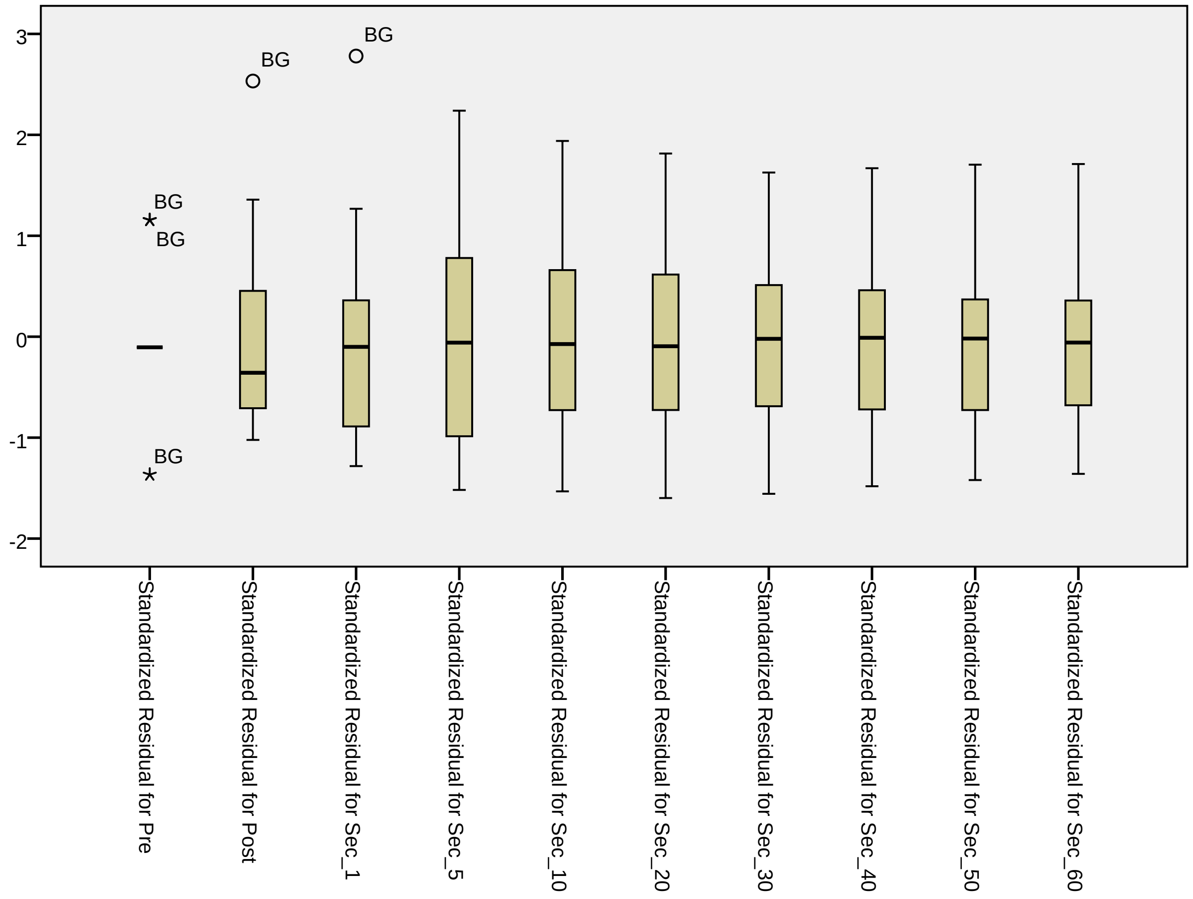

Normal Q-Q Plots of Standardized Residual for tHb During Recovery

Normal Q-Q Plots of Standardized Residual for tHb During Recovery

Normal Q-Q Plot of Standardized Residual for Pre

Treatment= PLA

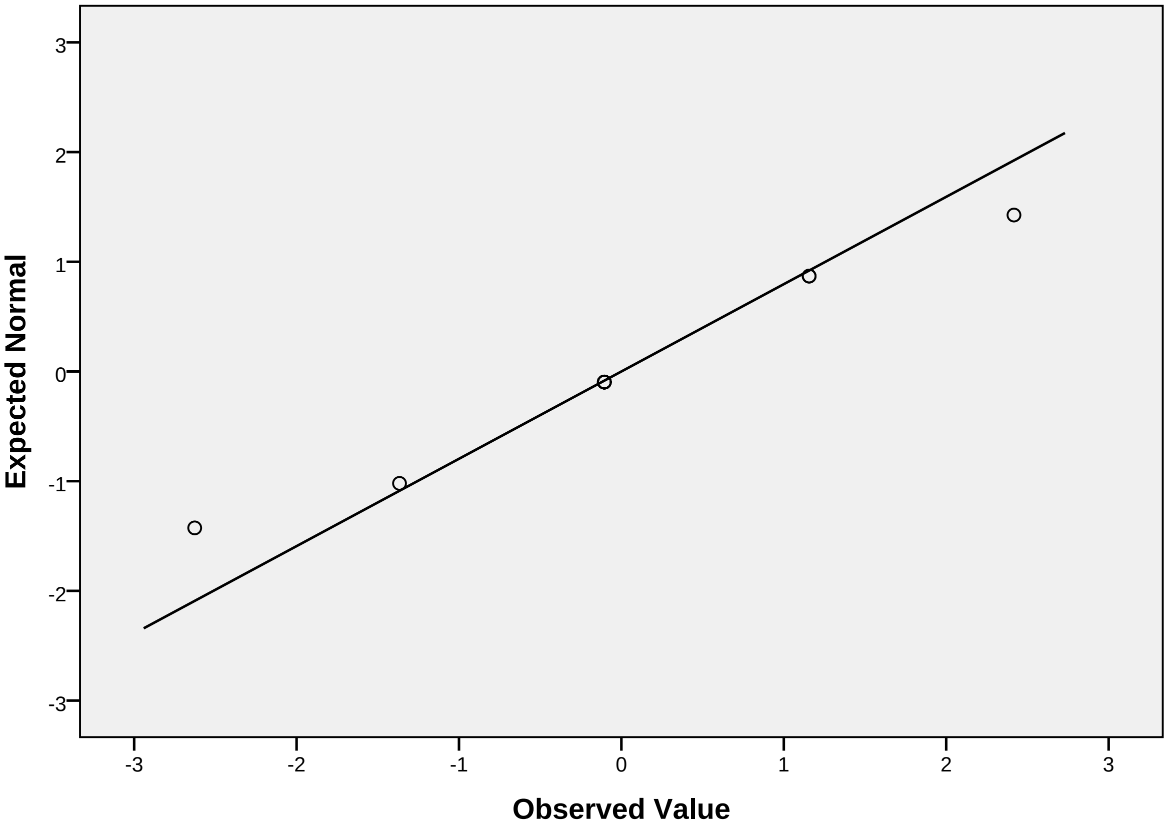

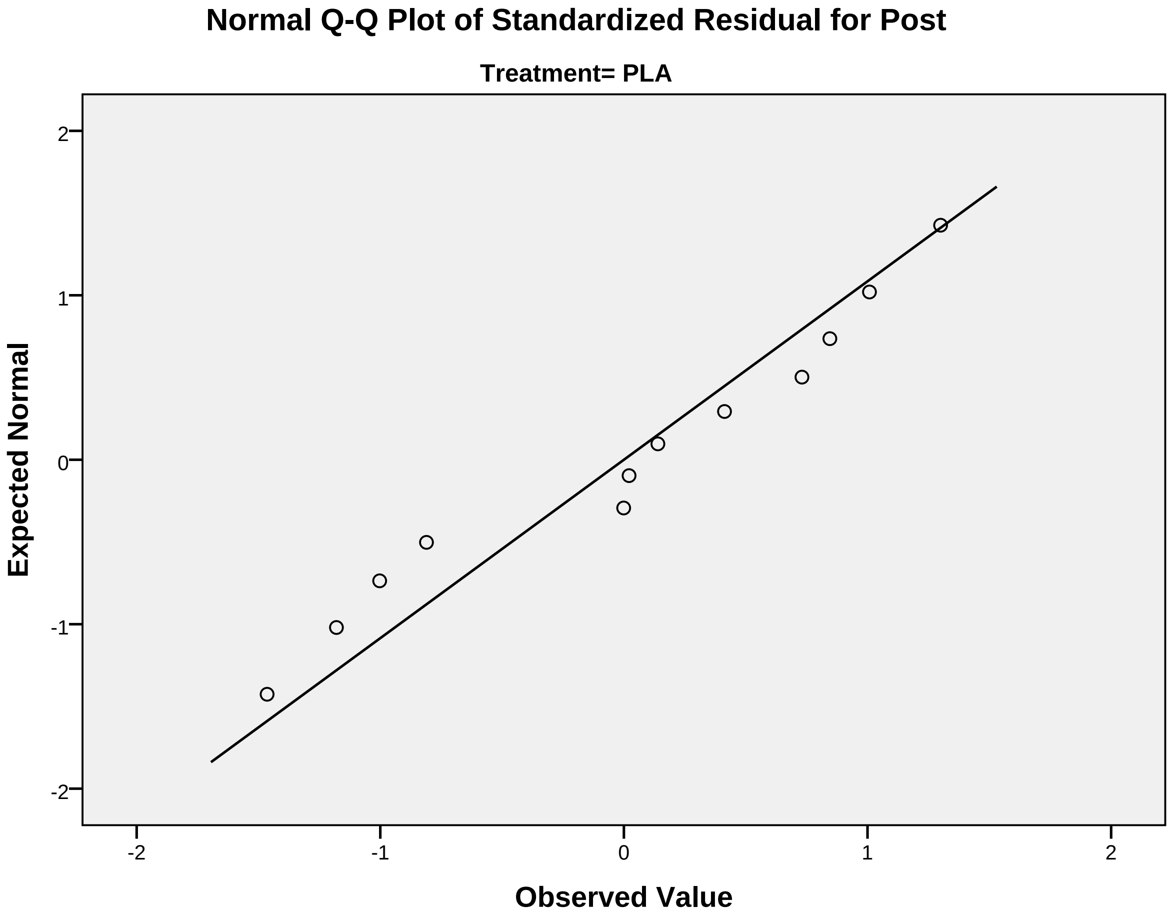

Normal Q-Q Plot of Standardized Residual for Post
Treatment= PLA
Expected Normal
Observed Value
2
1
0
-1
-2
-2
-1
0
1
2

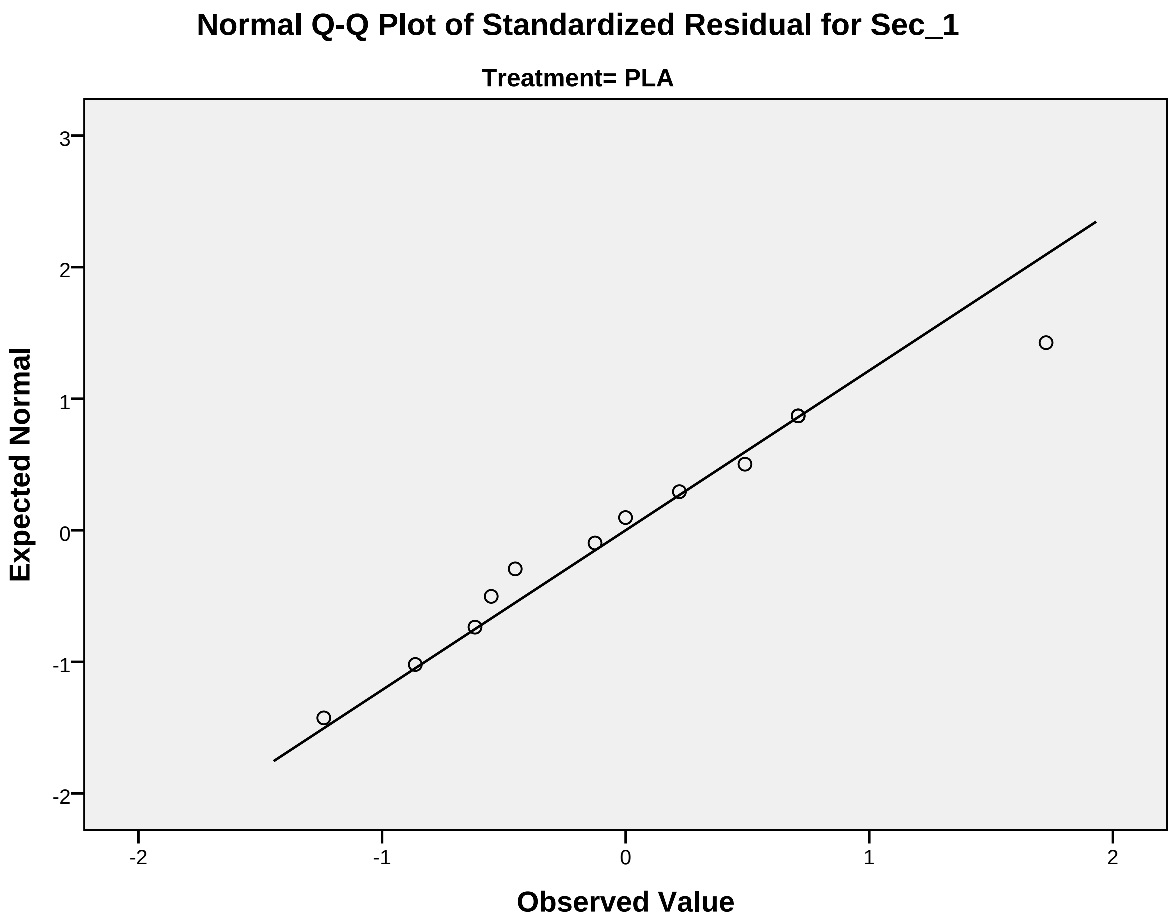
Normal Q-Q Plot of Standardized Residual for Sec_1
Treatment= PLA
Expected Normal
3
2
1
0
-1
-2
-2
-1
0
1
2
Observed Value

Normal Q-Q Plot of Standardized Residual for Sec_5

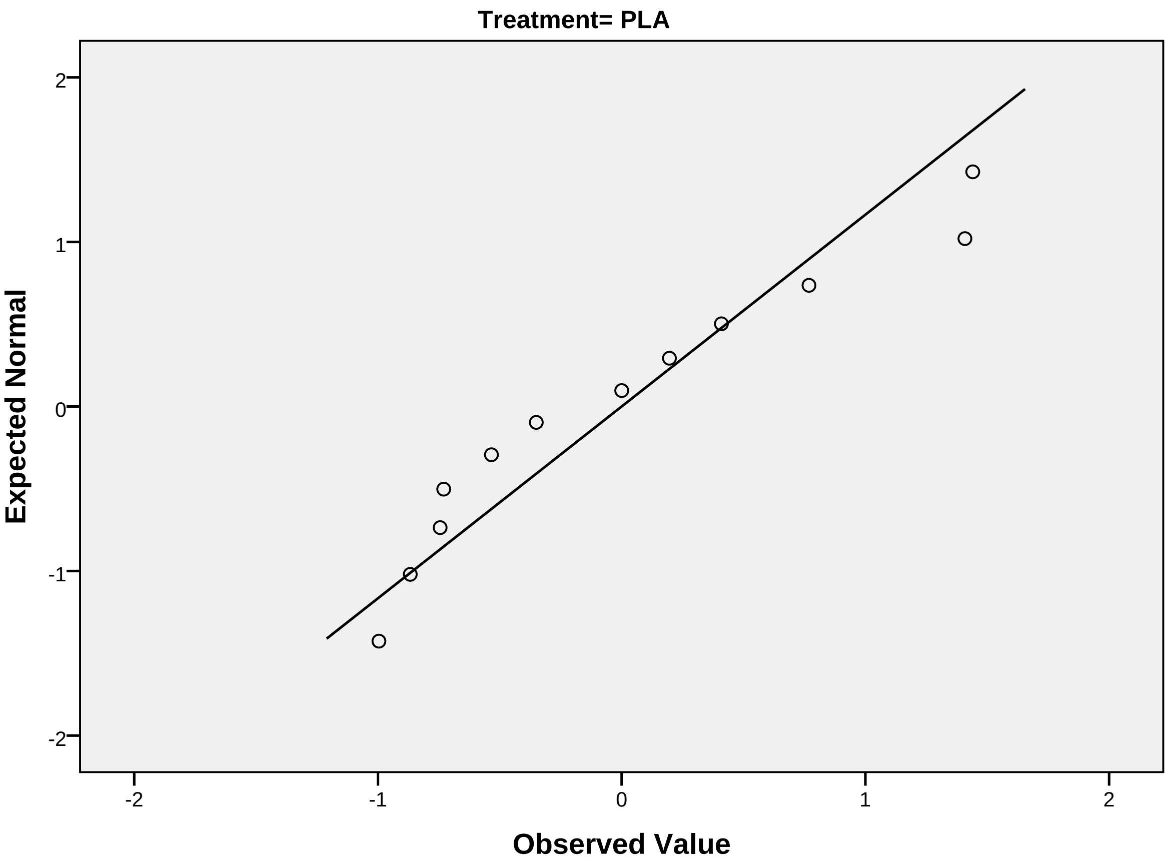

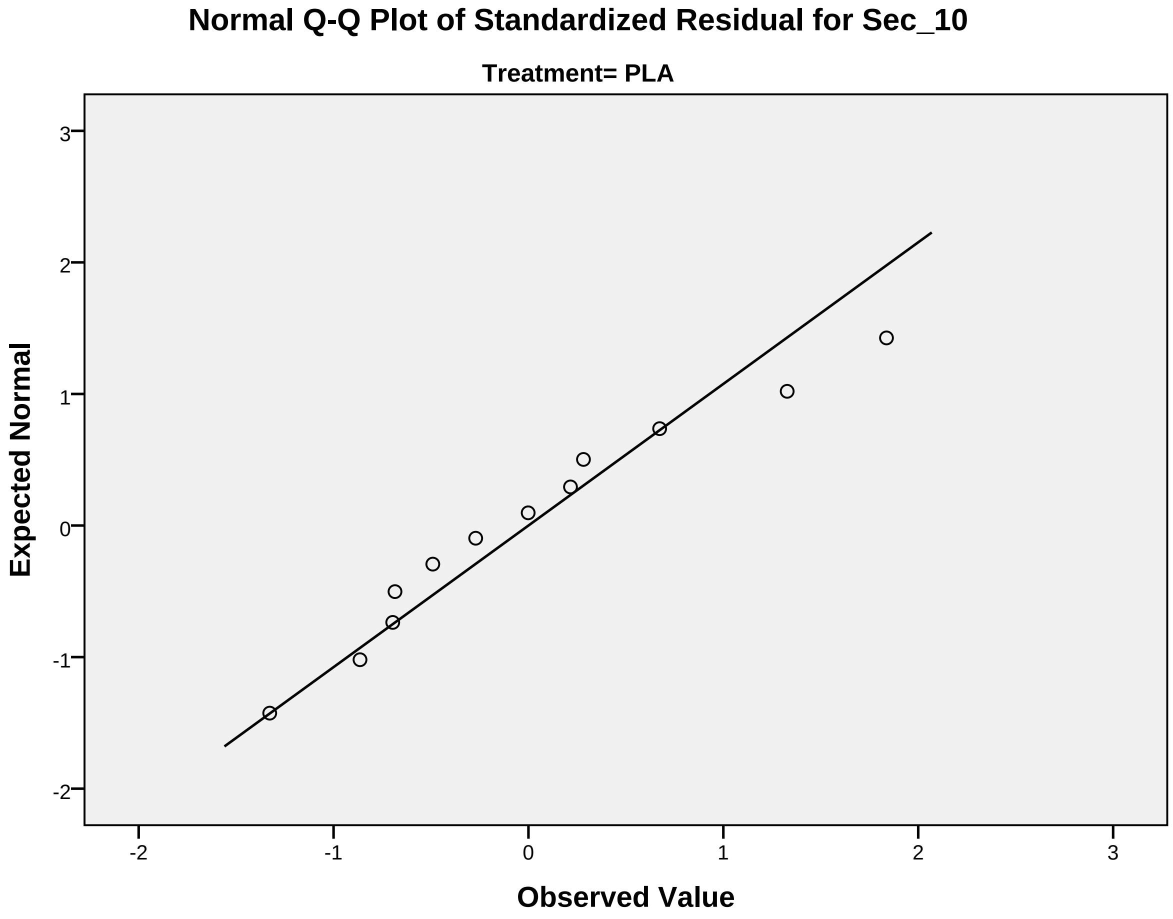
Normal Q-Q Plot of Standardized Residual for Sec_10
Treatment= PLA
Expected Normal
3
2
1
0
-1
-2
-2
-1
0
1
2
3
Observed Value

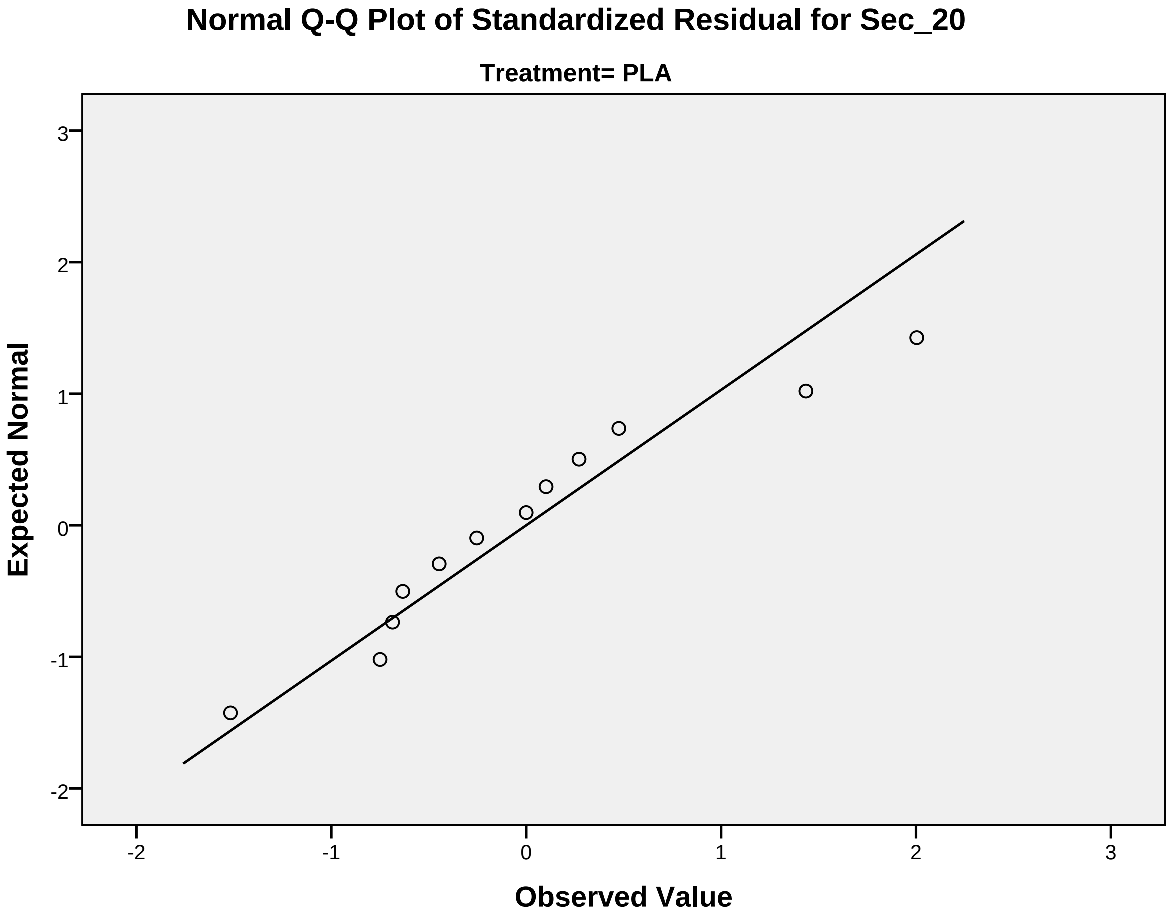

Normal Q-Q Plot of Standardized Residual for Sec_20
Treatment= PLA
Expected Normal
3
2
1
0
-1
-2
-2
-1
0
1
2
3
Observed Value

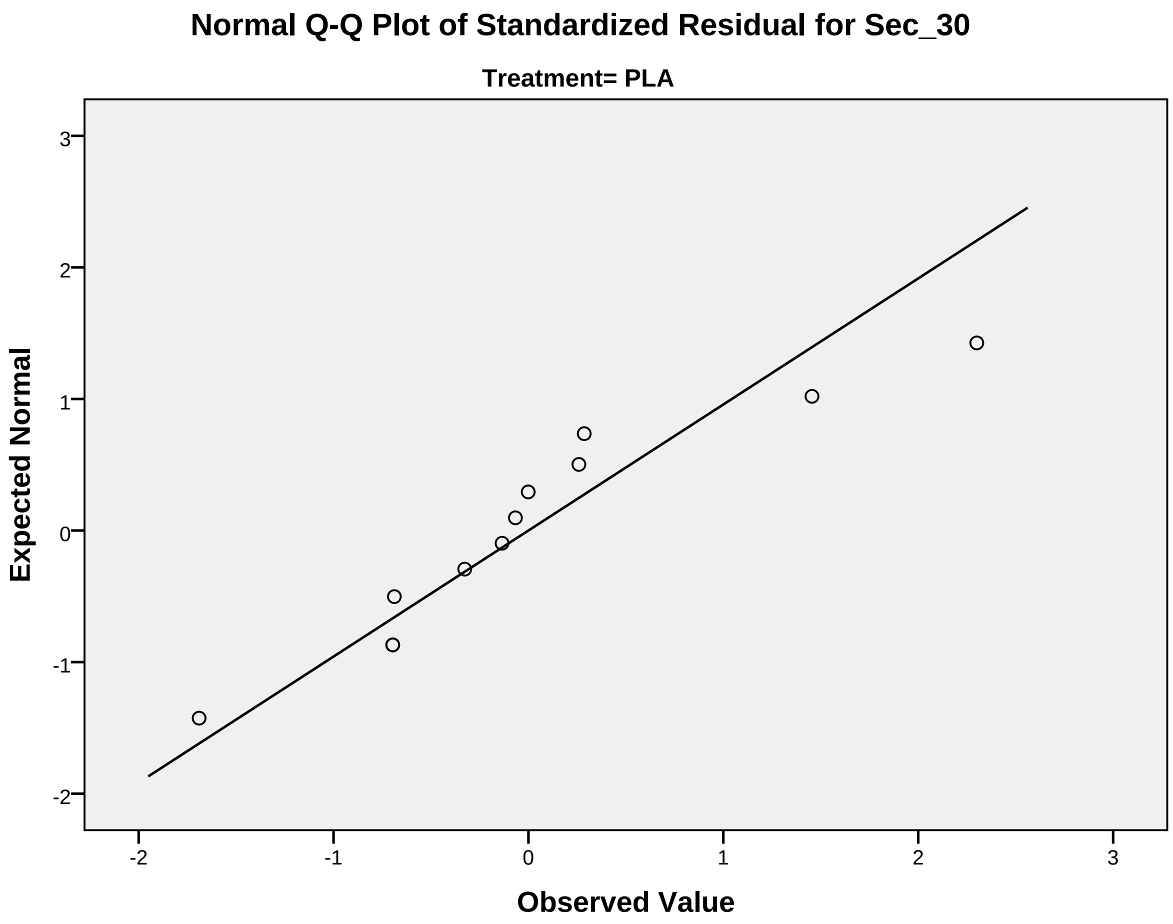
Normal Q-Q Plot of Standardized Residual for Sec_30
Treatment= PLA
Expected Normal
3
2
1
0
-1
-2
-2
-1
0
1
2
3
Observed Value

Normal Q-Q Plot of Standardized Residual for Sec_40

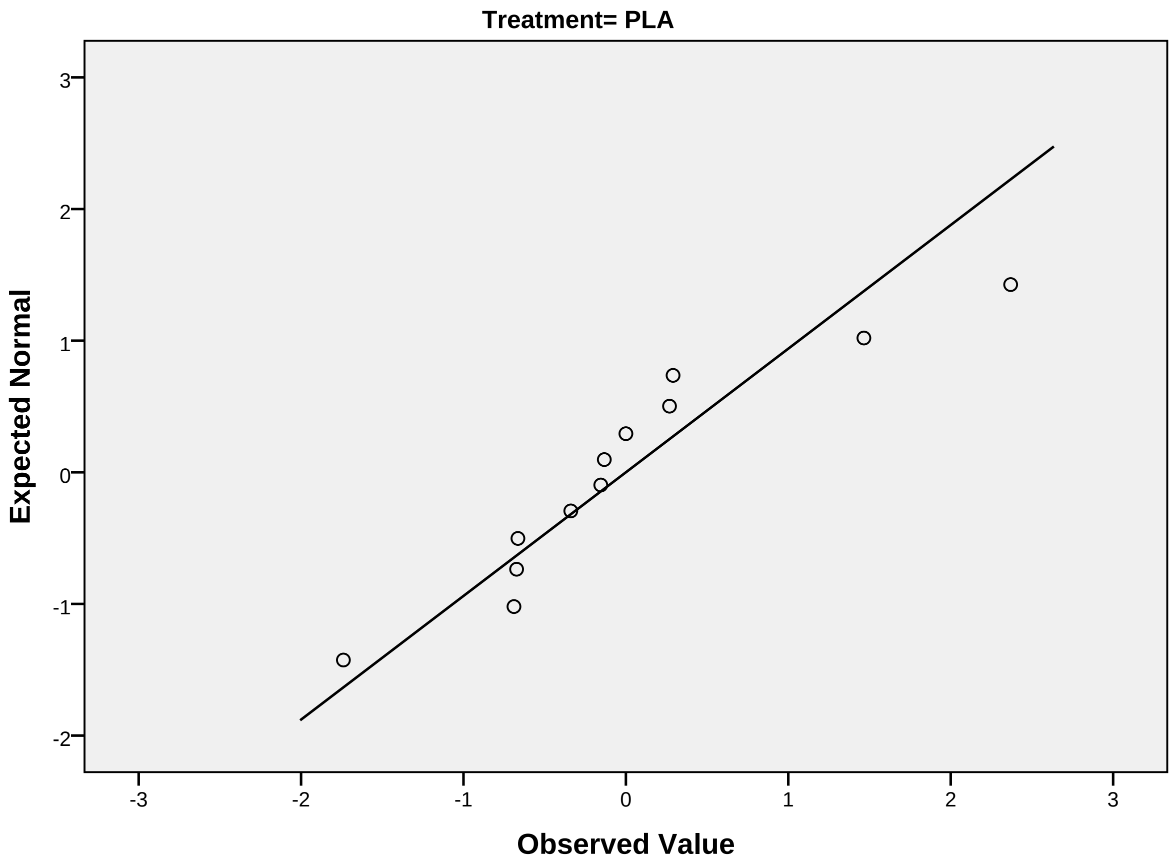

Normal Q-Q Plot of Standardized Residual for Sec_50

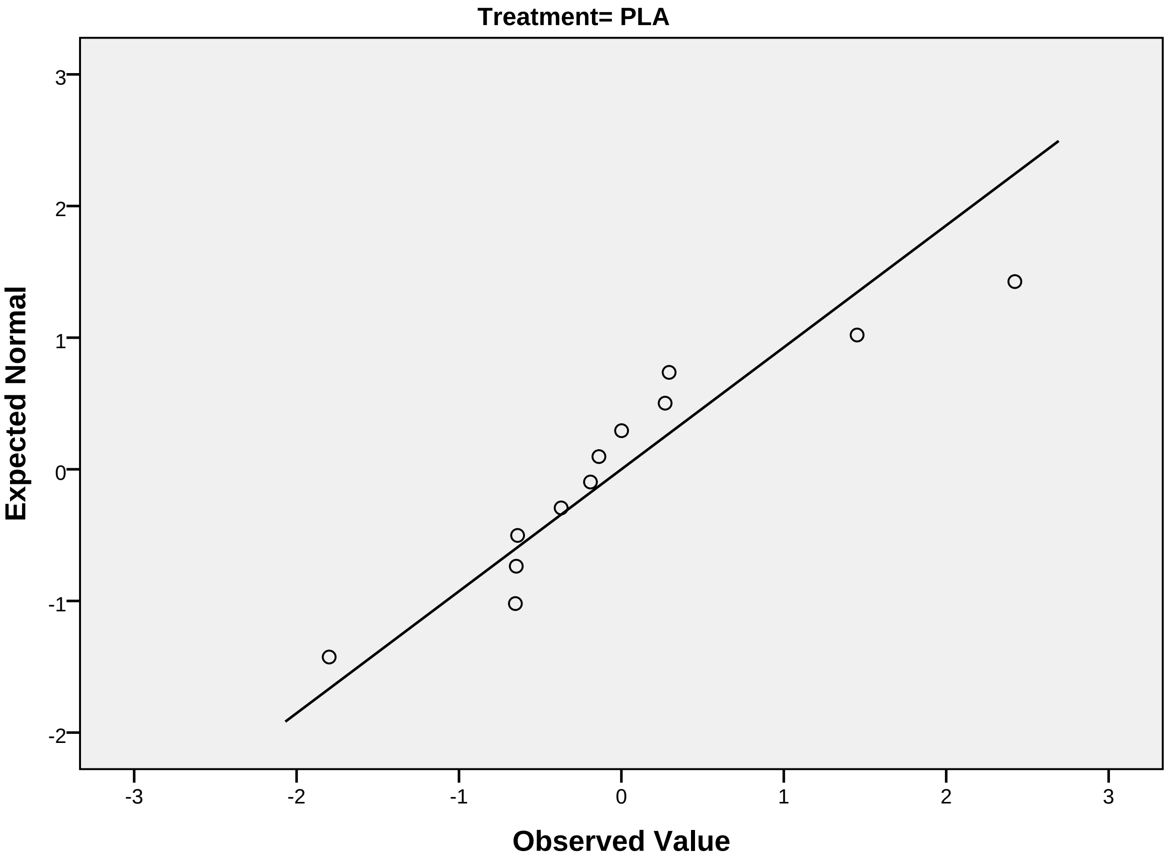

Normal Q-Q Plot of Standardized Residual for Sec_60

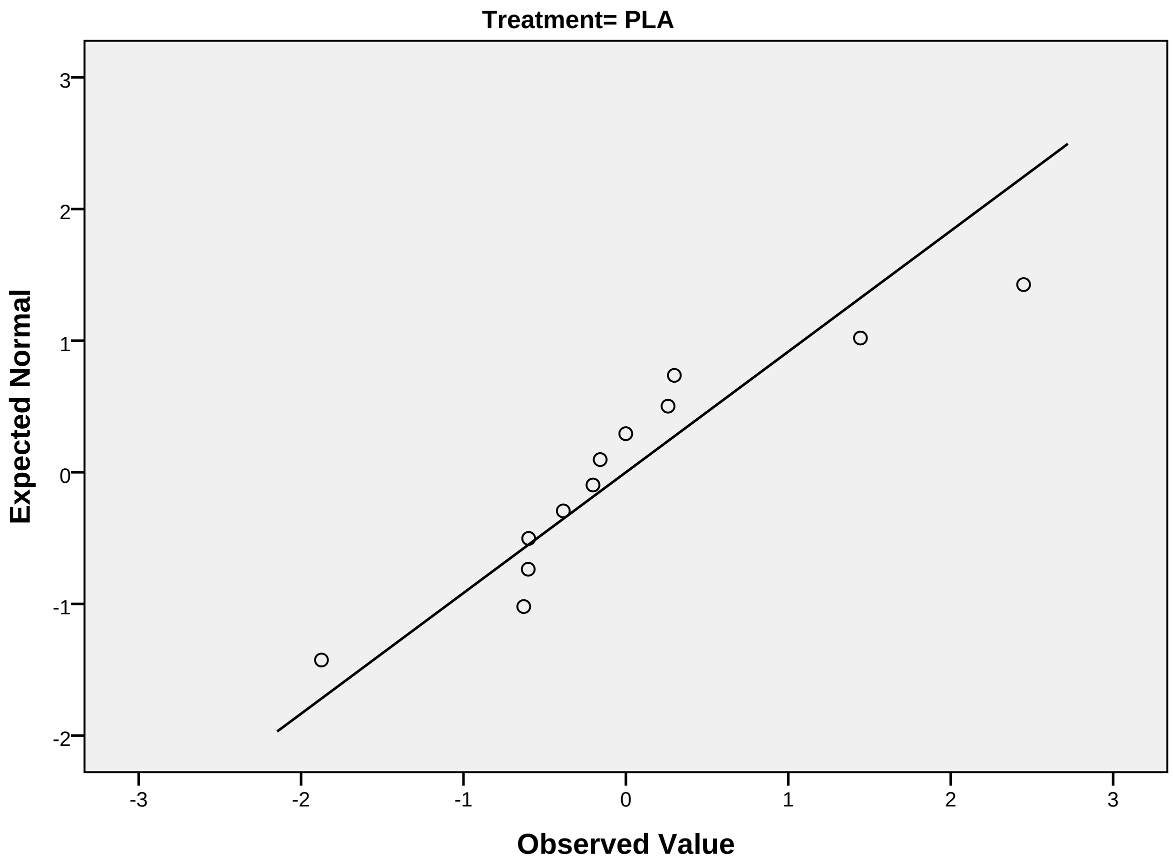

Detrended Normal Q-Q Plots of Standardized Residual for tHb During Recovery

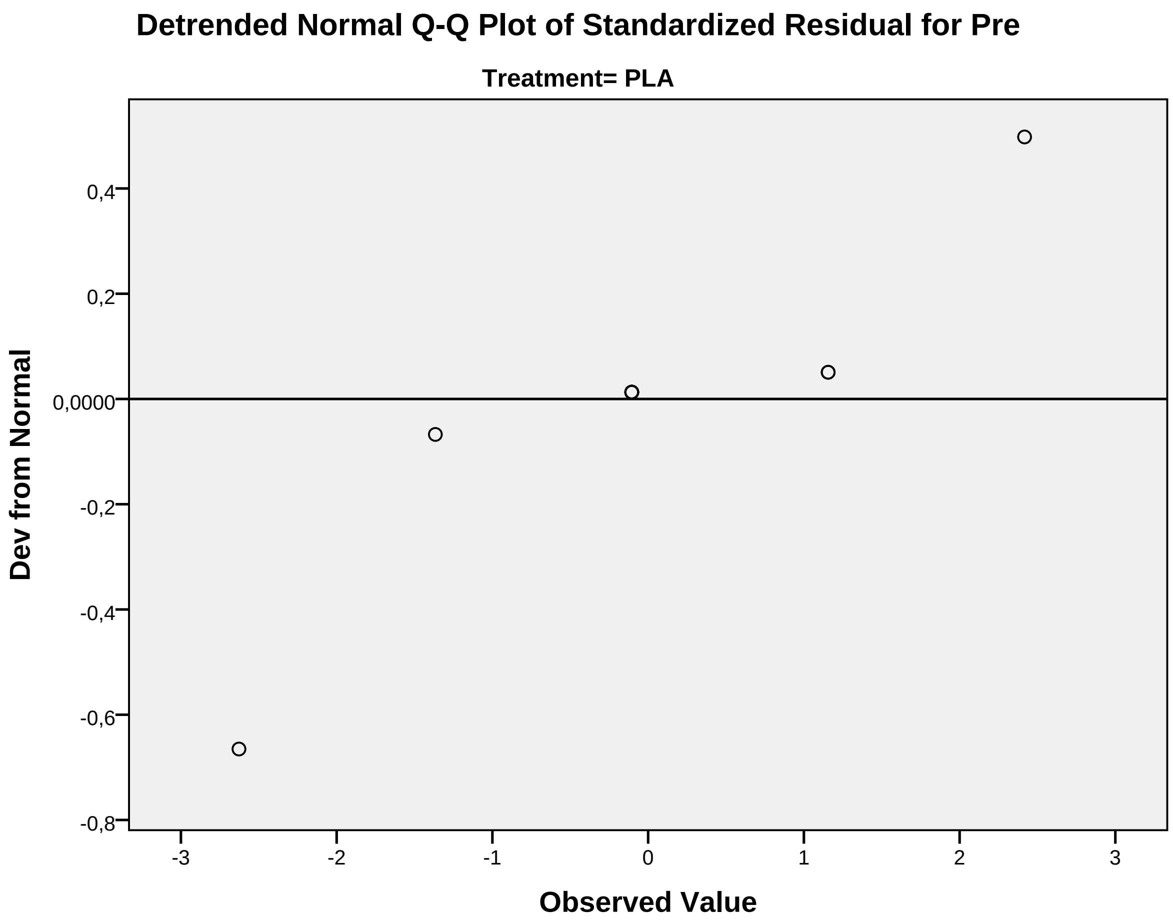
Detrended Normal Q-Q Plot of Standardized Residual for Pre
Treatment= PLA
Dev from Normal
0,4
0,2
0,0000
-0,2
-0,4
-0,6
-0,8
-3
-2
-1
0
1
2
3
Observed Value

Detrended Normal Q-Q Plot of Standardized Residual for Post

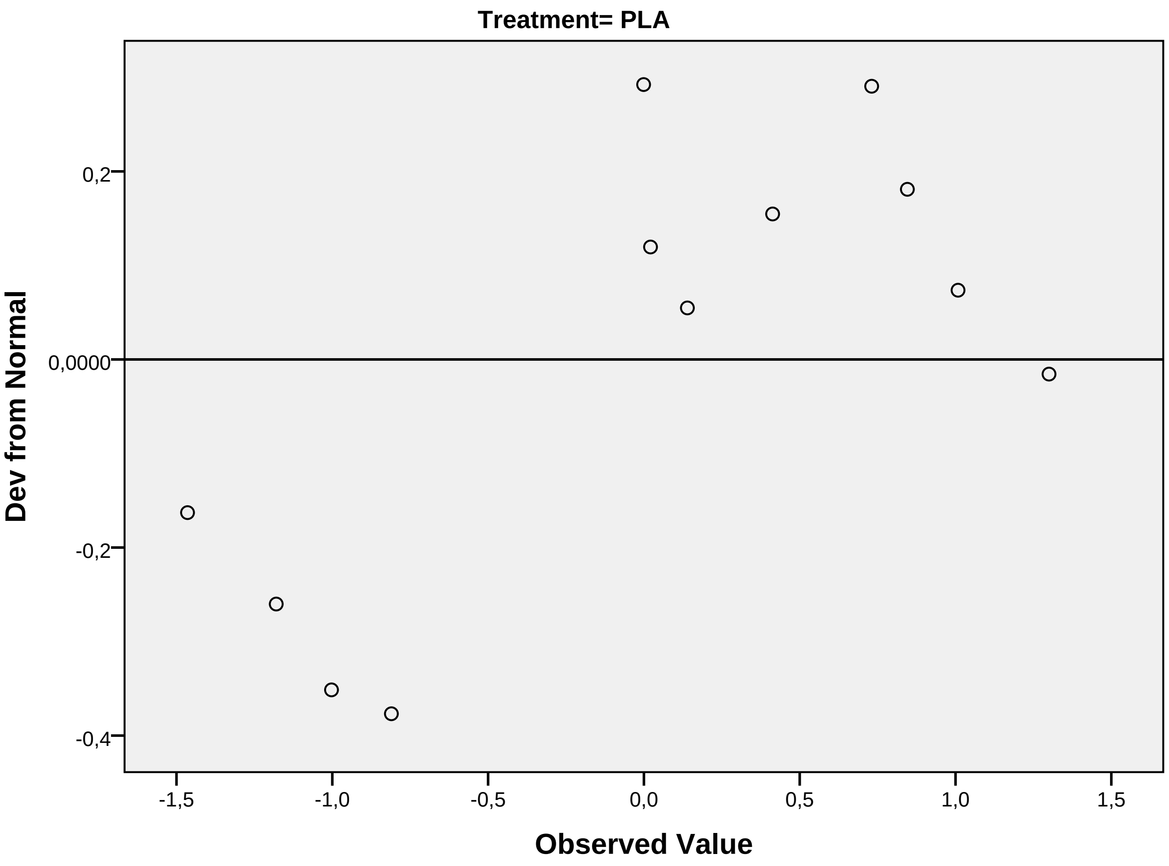

Detrended Normal Q-Q Plot of Standardized Residual for Sec_1

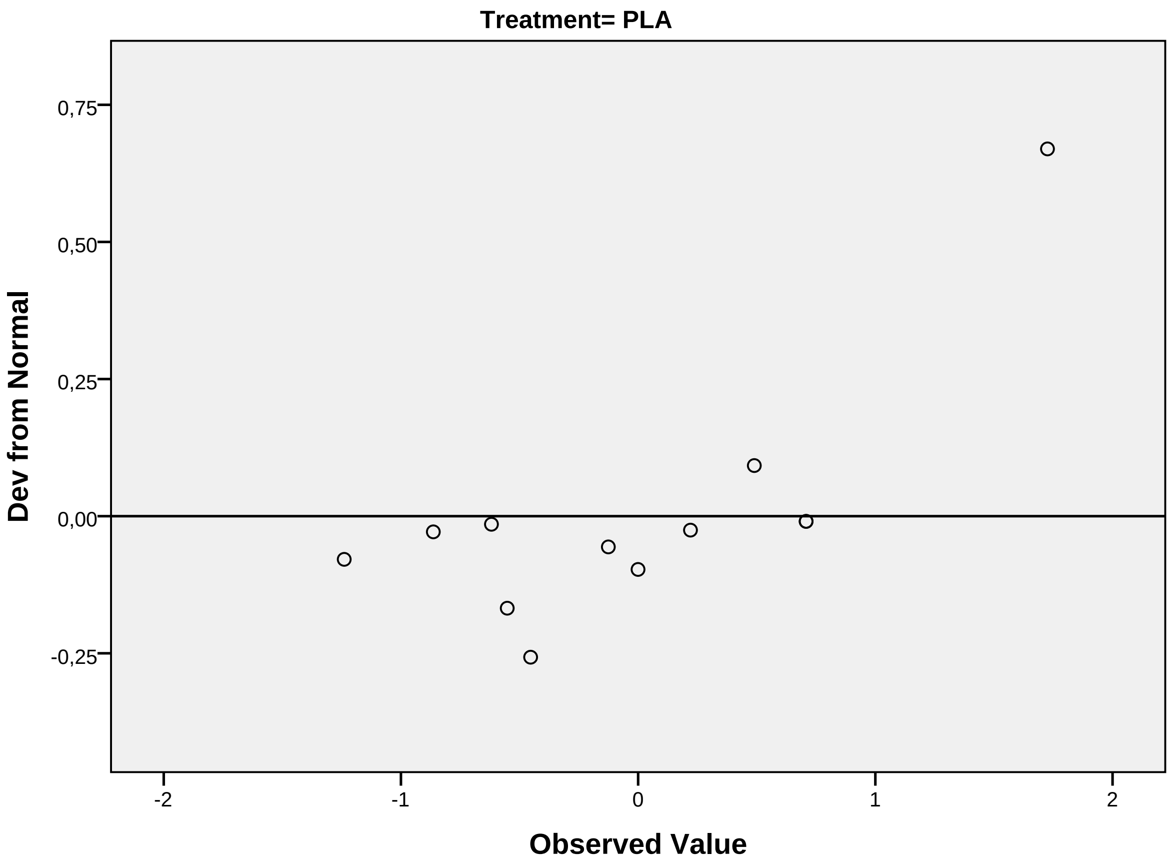

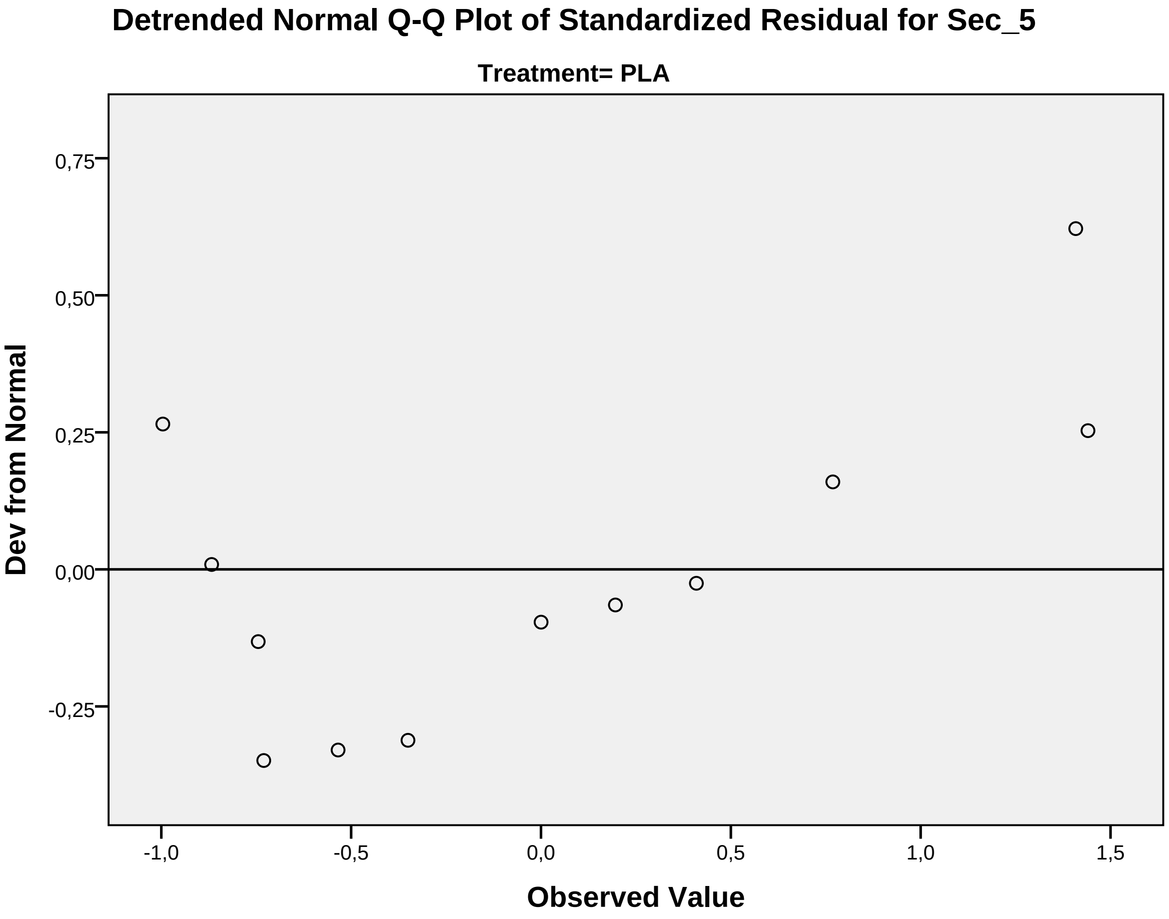
Detrended Normal Q-Q Plot of Standardized Residual for Sec_5
Treatment= PLA
Dev from Normal
0,75
0,50
0,25
0,00
-0,25
-1,0
-0,5
0,0
0,5
1,0
1,5
Observed Value

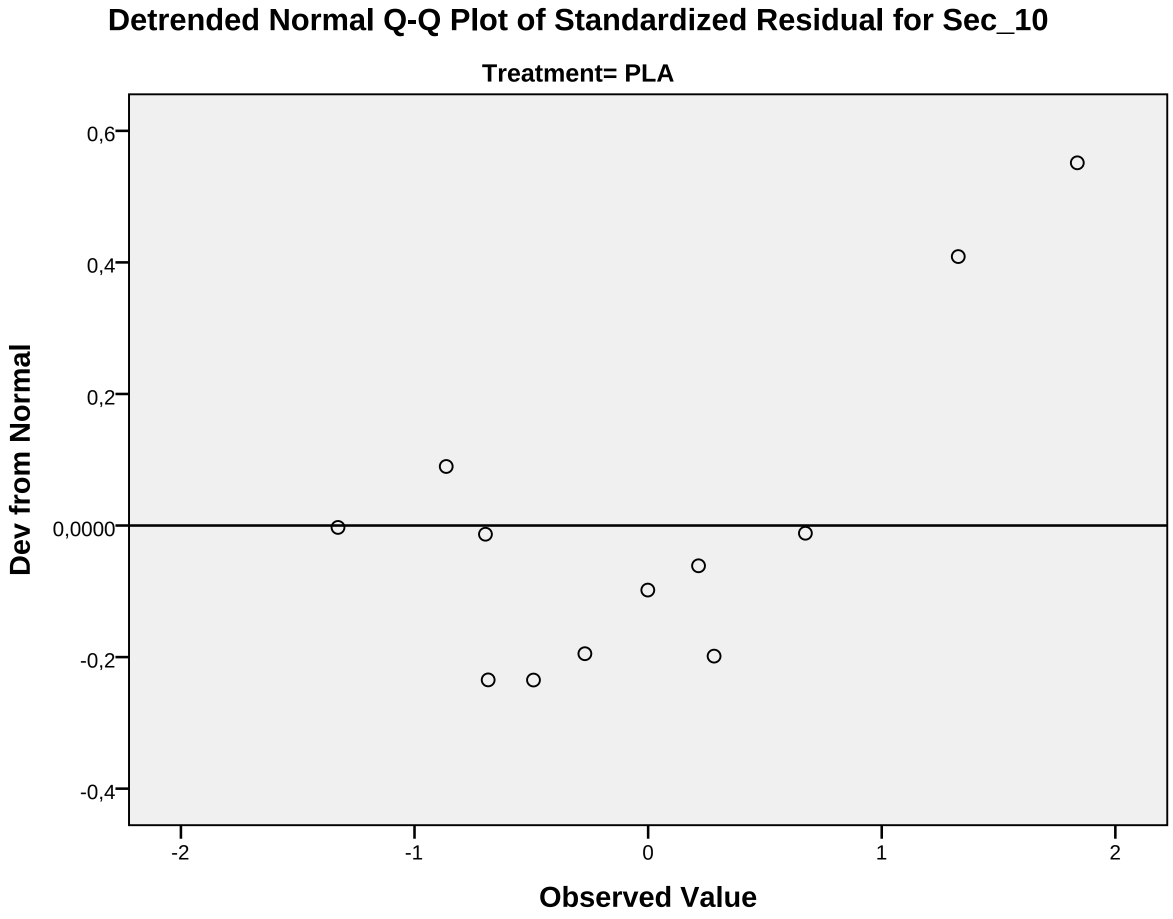
Detrended Normal Q-Q Plot of Standardized Residual for Sec_10
Treatment= PLA
Dev from Normal
0,6
0,4
0,2
0,0000
-0,2
-0,4
-2
-1
0
1
2
Observed Value

Detrended Normal Q-Q Plot of Standardized Residual for Sec_20

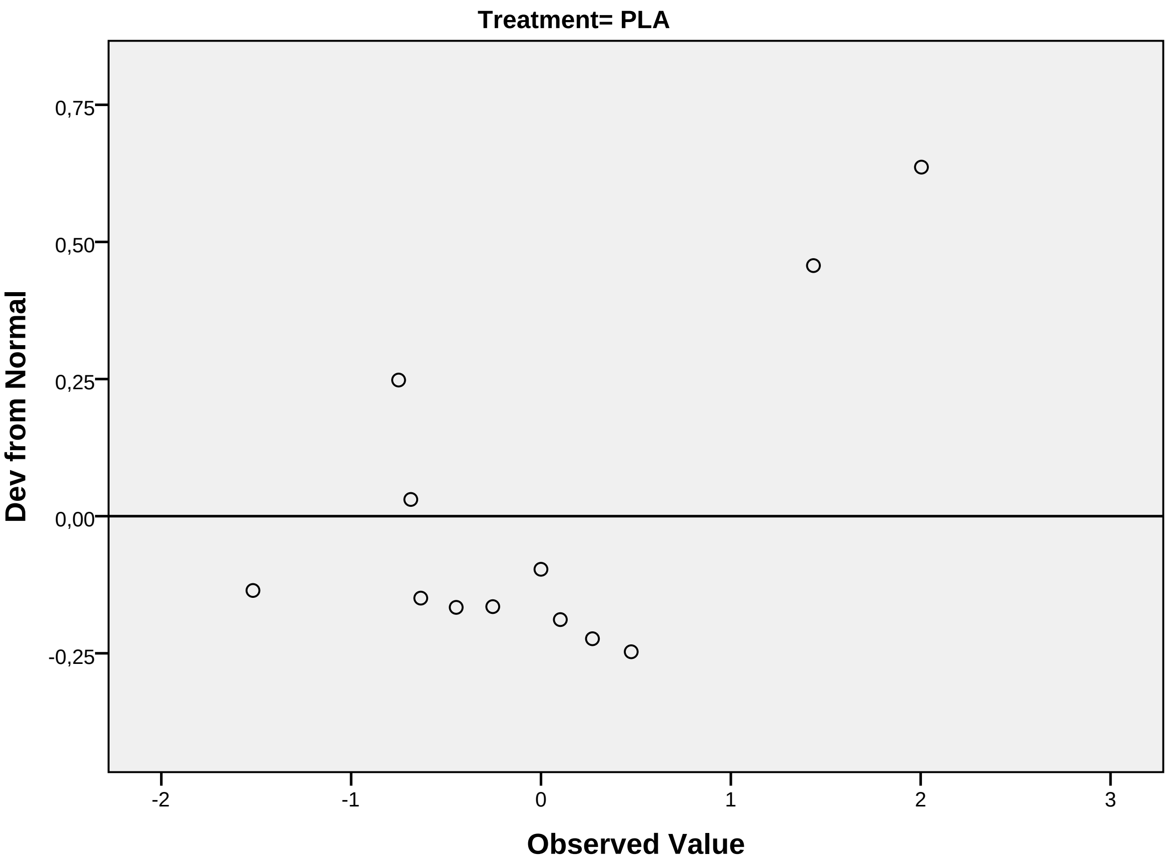

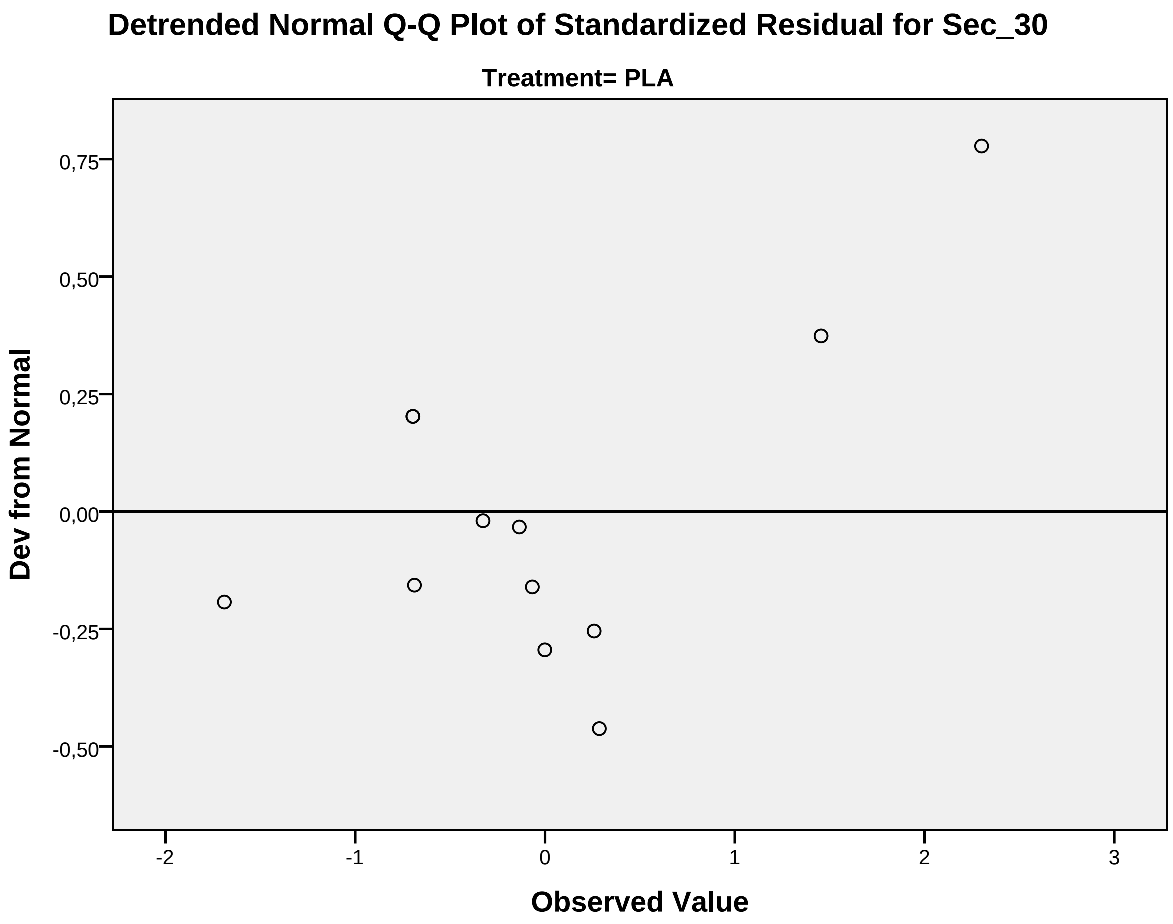
Detrended Normal Q-Q Plot of Standardized Residual for Sec_30
Treatment= PLA
Dev from Normal
0,75
0,50
0,25
0,00
-0,25
-0,50
-2
-1
0
1
2
3
Observed Value

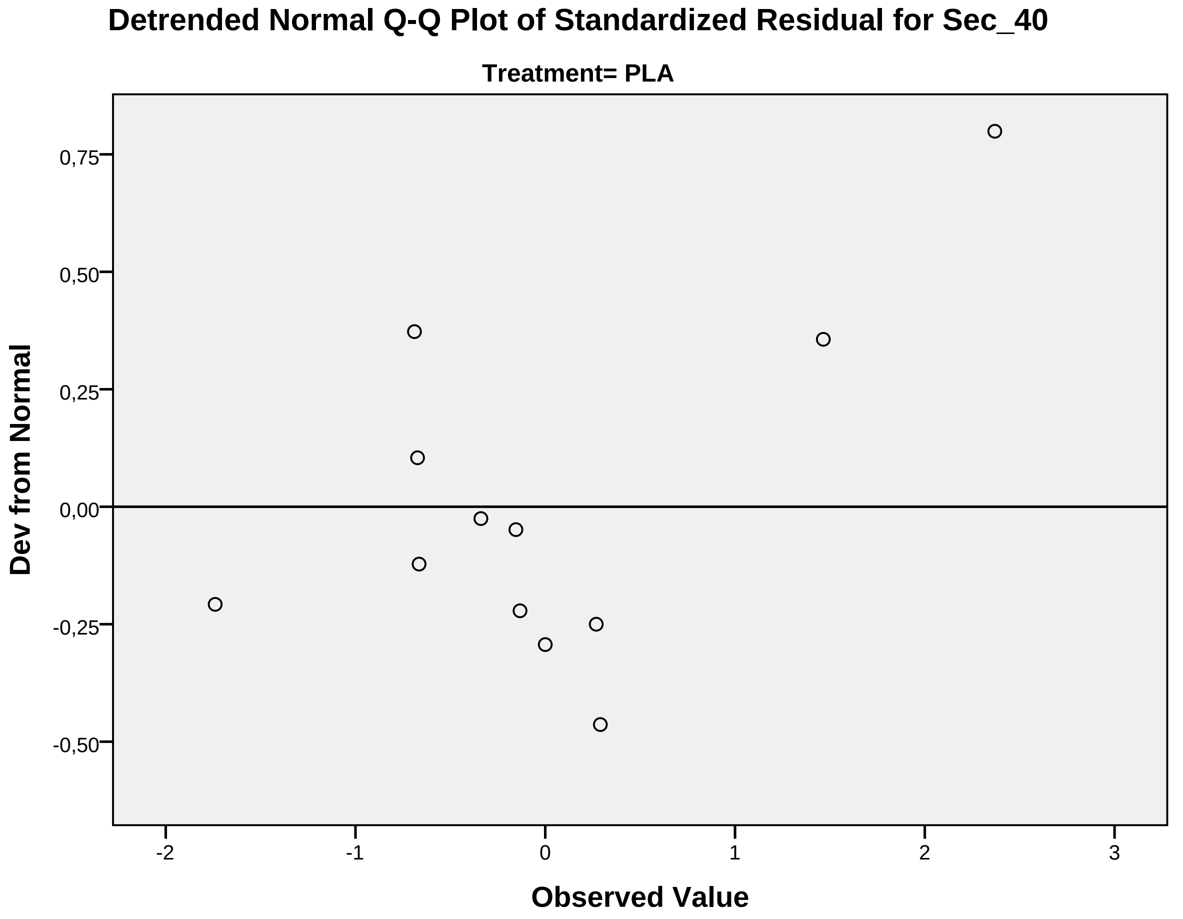
Detrended Normal Q-Q Plot of Standardized Residual for Sec_40
Treatment= PLA
Dev from Normal
0,75
0,50
0,25
0,00
-0,25
-0,50
-2
-1
0
1
2
3
Observed Value

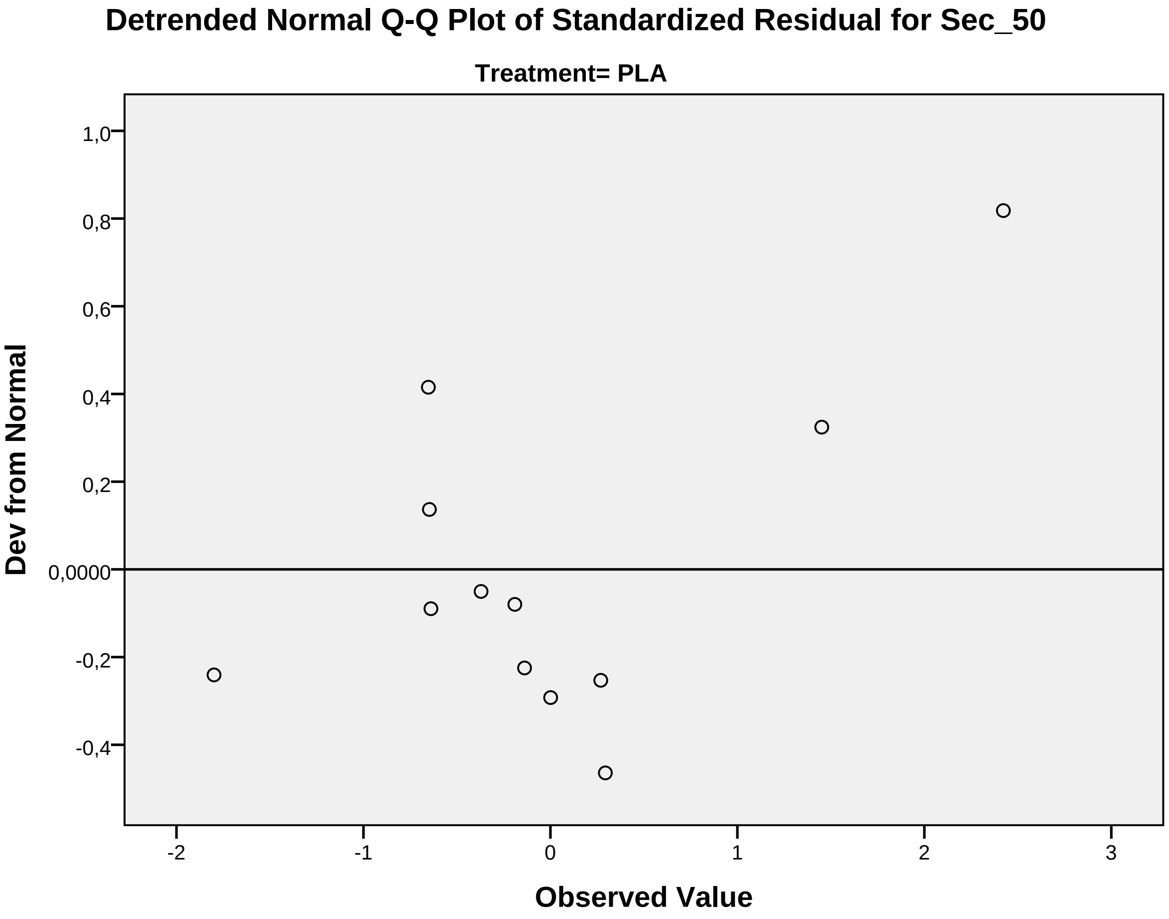
Detrended Normal Q-Q Plot of Standardized Residual for Sec_50
Treatment= PLA
Dev from Normal
1,0
0,8
0,6
0,4
0,2
0,0000
-0,2
-0,4
-2
-1
0
1
2
3
Observed Value

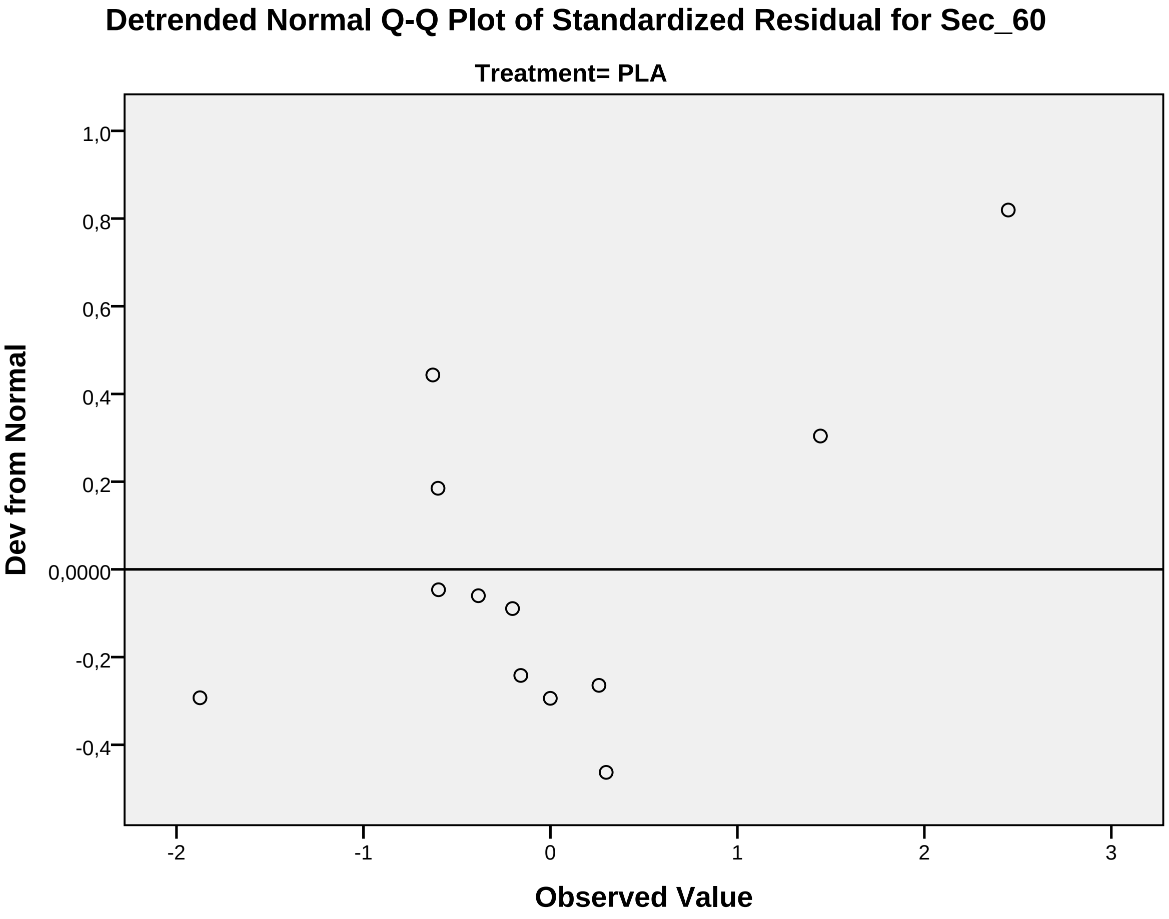

Detrended Normal Q-Q Plot of Standardized Residual for Sec_60
Treatment= PLA
Dev from Normal
1,0
0,8
0,6
0,4
0,2
0,0000
-0,2
-0,4
-2
-1
0
1
2
3
Observed Value

Treatment: PLA

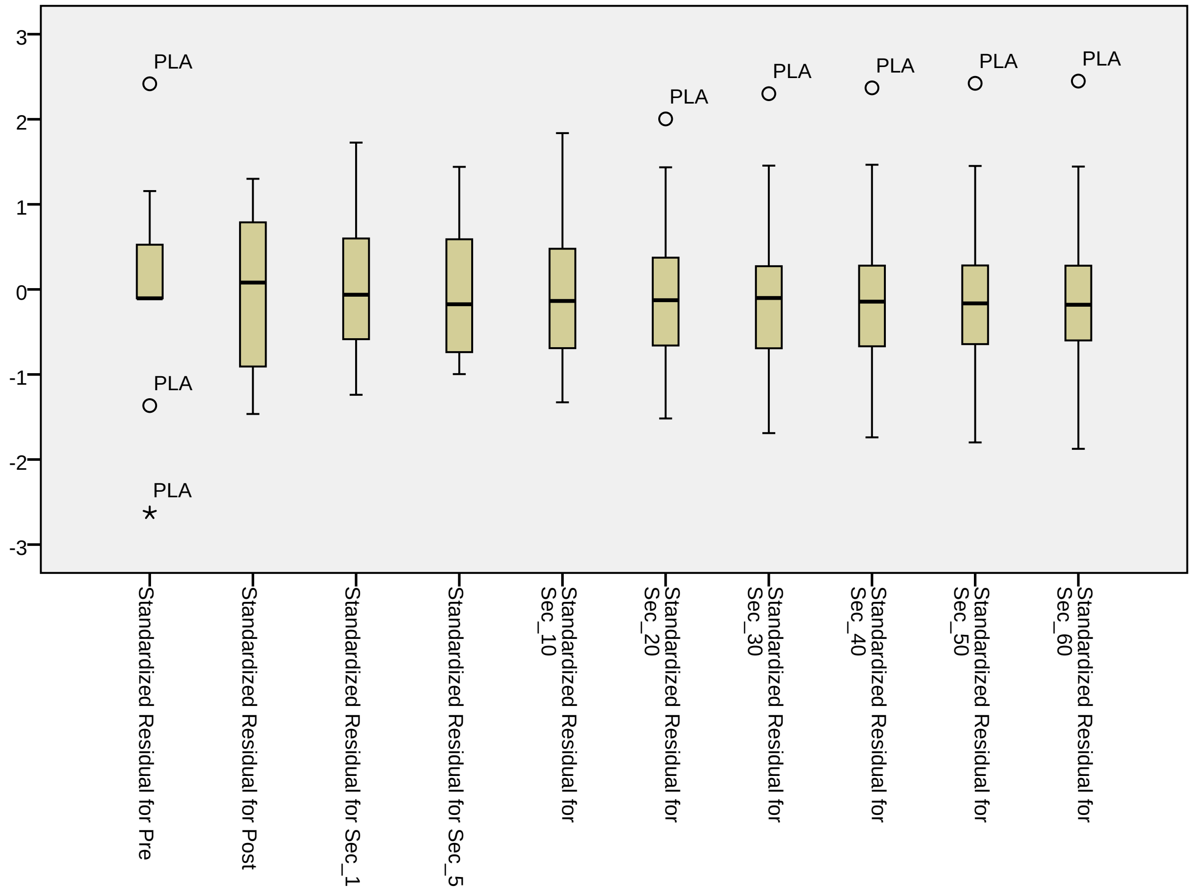

Supplement: S1 File — (PDF) [file pone.0188893.s001.pdf]
