# Supplementary material for: Acute effect of dietary nitrate on forearm muscle oxygenation, blood volume and strength in older adults: A randomized clinical trial
Source: PLoS One. 2017 Nov 30;12(11):e0188893. doi: 10.1371/journal.pone.0188893 (PMC5708833; doi:10.1371/journal.pone.0188893)
Supplement: S1 Table — Values are expressed as means ± SD. BG = beetroot-based nutritional gel, PLA = nitrate-depleted gel, O2Hb = muscle oxyhaemoglobin, HHb = muscle deoxyhaemoglobin. * Significantly different from PLA. a significantly different from Pre. (DOCX) [file pone.0188893.s002.docx]

**S2 Table.** Changes in muscle O_2_Hb and HHb during handgrip exercise and during exercise recovery.

| Time (sec) | Condition | O_2_Hb_Ex_ (µM) | HHb_Ex_ (µM) | O_2_Hb_Rec_ (µM) | HHb_Rec_ (µM) |
| --- | --- | --- | --- | --- | --- |
| Pre | BG | -0.08 ± 0.49 | 0.00 ± 0.00 | -0.08 ± 0.49 | 0.00 ± 0.00 |
|  | PLA | 0.00 ± 0.36 | 0.00 ± 0.00 | 0.00 ± 0.36 | 0.00 ± 0.00 |
| Post | BG | - | - | -7.40 ± 5.76*^,a^ | 6.87 ± 4.83^a^ |
|  | PLA | - | - | -3.01 ± 4.20 | 4.23 ± 2.05 |
| 1 | BG | -0.31 ± 1.99 | 0.04 ± 1.64 | -6.24 ± 5.30^a^ | 9.85 ± 5.02^a^ |
|  | PLA | 0.56 ± 2,23 | -0.14 ± 1.16 | -2.62 ± 6.63 | 7.82 ± 5.35 |
| 5 | BG | -1.57 ± 1.98 | -0.35 ± 1.58 | -3.55 ± 4.52 | 9.30 ± 5.56 |
|  | PLA | 0.15 ± 2.35 | -0.15 ± 1.18 | -1.17 ± 5.58 | 6.86 ± 6.01 |
| 10 | BG | -2.16 ± 2.4 | 0.25 ± 1.68 | -3.06 ± 4.41 | 9.04 ± 5.95 |
|  | PLA | 0.20 ± 2.61 | 0,07 ± 1.36 | -0.85 ± 5.13 | 6.30 ± 6.08 |
| 20 | BG | -3.41 ± 3.70 | 1.78 ± 2.75 | -2.62 ± 4.55 | 8.66 ± 6.04 |
|  | PLA | -0.73 ± 2,75 | 0.84 ± 1.83 | -0.62 ± 4.72 | 5.73 ± 5.81 |
| 30 | BG | -4.88 ± 4.57*^,a^ | 3.48 ± 3.55 | -1.17 ± 4.43 | 7.65 ± 6.12 |
|  | PLA | -1.45 ± 2.97 | 1.90 ± 1.91 | 0.30 ± 3.94 | 4.85 ± 5.36 |
| 40 | BG | -6.00 ± 5.16*^,a^ | 4.93 ± 4,12 | -0.37 ± 4.26 | 6.85 ± 5.94 |
|  | PLA | -2.04 ± 3.22 | 2.74 ± 1.84 | 0.77 ± 3.62 | 4.28 ± 5.07 |
| 50 | BG | -6.87 ± 5.55*^,a^ | 6.03 ± 4.53^a^ | 0.26 ± 4.03 | 6.15 ± 5.67 |
|  | PLA | -2.58± 3.68 | 3.53 ± 1.84 | 1.12 ± 3.31 | 3.77 ± 4.72 |
| 60 | BG | -7.40 ± 5.76*^,a^ | 6.87 ± 4.83^a^ | 0.79 ± 3.78 | 5.48 ± 5.30 |
|  | PLA | -3.01 ± 4.20 | 4.23 ± 2.05 | 1.41 ± 3.05 | 3.31 ± 4.36 |

Values are expressed as means ± SD. BG = beetroot-based nutritional gel, PLA = nitrate-depleted gel, O_2_Hb = muscle oxyhaemoglobin, HHb = muscle deoxyhaemoglobin. * Significantly different from PLA. ^a^ significantly different from Pre.
