# Supplementary material for: Acute effect of dietary nitrate on forearm muscle oxygenation, blood volume and strength in older adults: A randomized clinical trial
Source: PLoS One. 2017 Nov 30;12(11):e0188893. doi: 10.1371/journal.pone.0188893 (PMC5708833; doi:10.1371/journal.pone.0188893)
Supplement: S2 Table — Values are expressed as means ± SD. BG = beetroot-based nutritional gel, PLA = nitrate-depleted gel, O2Hb = muscle oxyhaemoglobin. * Significantly different from PLA. (DOCX) [file pone.0188893.s003.docx]

**S3 Table.** Changes in muscle O_2_Hb parameters during handgrip exercise and during exercise recovery.

|  | BG | PLA |
| --- | --- | --- |
| O_2_Hb_min_ (µM) | -13.39 ± 8.46* | -7.45 ± 7.43 |
| O_2_Hb_DR_ (%.s^-1^) | -1.65 ± -1.42 | -1.02 ± 1.30 |
| O_2_Hb_RR_ (%.s^-1^) | 1.34 ± 0.87* | 0.77 ± 0.91 |

Values are expressed as means ± SD. BG = beetroot-based nutritional gel, PLA = nitrate-depleted gel, O_2_Hb = muscle oxyhaemoglobin. * Significantly different from PLA.
